# Galectin-3 Induces a Pro-degradative/inflammatory Gene Signature in Human Chondrocytes, Teaming Up with Galectin-1 in Osteoarthritis Pathogenesis

**DOI:** 10.1038/srep39112

**Published:** 2016-12-16

**Authors:** Daniela Weinmann, Karin Schlangen, Sabine André, Sebastian Schmidt, Sonja M. Walzer, Bernd Kubista, Reinhard Windhager, Stefan Toegel, Hans-Joachim Gabius

**Affiliations:** 1 Karl Chiari Lab for Orthopaedic Biology, Department of Orthopaedics, Medical University of Vienna, Austria; 2 Center for Medical Statistics, Informatics, and Intelligent Systems, Medical University of Vienna, Austria; 3 Institute of Physiological Chemistry, Faculty of Veterinary Medicine, Ludwig-Maximilians-University Munich, Germany

## Abstract

Inflammatory chemo- and cytokines and matrix-degrading proteases underlie the progression of osteoarthritis (OA). Aiming to define upstream regulators for these disease markers, we pursued initial evidence for an upregulation of members of the adhesion/growth-regulatory galectin family. Immunohistochemical localization of galectin-3 (Gal-3) in sections of human cartilage with increasing levels of degeneration revealed a linear correlation reaching a chondrocyte positivity of 60%. Presence *in situ* was cytoplasmic, the lectin was secreted from OA chondrocytes in culture and binding of Gal-3 yielded lactose-inhibitable surface staining. Exposure of cells to the lectin led to enhanced gene expression and secretion of functional disease markers. Genome-wide transcriptomic analysis broadened this result to reveal a pro-degradative/inflammatory gene signature under the control of NF-κB. Fittingly, targeting this route of activation by inhibitors impaired the unfavourable response to Gal-3 binding, as also seen by shortening the lectin’s collagen-like repeat region. Gal-3’s activation profile overlaps with that of homodimeric galectin-1 (Gal-1) and also has distinctive (supplementing) features. Tested at subsaturating concentrations in a mixture, we found cooperation between the two galectins, apparently able to team up to promote OA pathogenesis. In summary, our results suggest that a network of endogenous lectins is relevant for initiating this process cascade.

The clinical course of osteoarthritis (OA), the most prevalent joint disorder in the ageing population, leads to cartilage destruction, thereby causing an enormous human and economic toll^1^,^2^. Whereas the molecular mechanisms of tissue degradation are well explored and functional disease markers have been defined, the identification of effectors active in early steps of OA pathogenesis remains a conspicuous challenge. Toward this end, three lines of reasoning account for the interest to study the family of endogenous β-galactoside-binding proteins with β-sandwich fold (i.e., galectins): i) in principle, galectins are capable to specifically bind distinct glycans of cell surface glycoconjugates and to translate their information into effects with broad relevance for diverse processes such as cell growth, inflammation and tissue remodelling, and also exert activities intracellularly via respective binding partners^3^,^4^,^5^, ii) the tight control of their expression by central factors such as tumour suppressors (p53, p16^INK4a^) or corticosteroids that activate cell-type-specific cell death programs via galectins as mediators illustrates their capacity to act as molecular switches in (patho)physiological homeostasis^5^,^6^,^7^, and iii) their presence can markedly alter the course of disease in rheumatoid arthritis (RA) in animal models^8^,^9^,^10^,^11^,^12^.

Having first revealed an increase of positivity for galectin presence in severely degenerated cartilage in OA^13^ and then the upregulation of the levels of galectins-1, -3, -4 and -8 (Gal-1, -3, -4 and -8) in OA chondrocytes *in vitro* and in clinical specimens^14^, our previous studies have shaped the concept of participation of a network of galectins in OA pathogenesis. Since this disease hereby offers the attractive possibility to initiate to study the functional cooperation between galectins *in situ*, a so far not yet explored area, characterizing the individual activity profiles alone and in combination is the route to be taken for respective studies. Having started with Gal-1, we recently discovered that this galectin, whose presence in OA cartilage is associated with increasing degeneration (p < 0.0001, n = 29), represents an inducer of a set of disease markers via NF-κB activation^15^. In contrast to its strong anti-inflammatory role in RA and other autoimmune diseases^5^,^12^,^16^, Gal-1 thus upregulates clinically relevant pro-degradative/inflammatory activities in OA chondrocytes^17^. Obviously, the clinical context critically matters for the activity of Gal-1. This salient insight precludes extrapolations and makes case-by-case studies for the galectins necessary, on the levels of the disease and the protein. As the second piece of the galectin puzzle in OA, we here present the data of respective work on Gal-3 in clinical material and OA chondrocytes *in vitro*. Equally important, as consequence of the network concept, the analysis of the impact of co-incubation with both galectins, mimicking the pathophysiological condition, was initiated to take the first step in testing the hypothesis for functional cooperation.

Gal-3 has a trimodular design with a non-triple helical collagen-like stalk, fundamentally different from homodimeric Gal-1^18^. *In situ*, its interaction with and cross-linking of the glycoprotein lubricin contributes to cartilage lubrication^19^. Serum and synovial fluid of OA patients showed no significant increase in their Gal-3 levels, in contrast to specimens from RA^20^. Adding to a role of Gal-3 in chondrocyte survival delineated in a mouse model^21^,^22^, this galectin yet caused OA-like lesions by intra-articular injection in knee joints in mice and favoured conditions for proteoglycan degradation by human OA chondrocytes^23^,^24^. Of relevance for a pro-inflammatory potential, Gal-3 of synovial fibroblasts of OA and RA patients, in a paracrine manner, appears to be a co-mediator in lipopolysaccharide (LPS)-induced interleukin (IL)-6 secretion using Toll-like pattern-recognition receptors (TLRs) as sensors^25^. Such a role had also been reported for microglia, where TLR4 is a Gal-3 counterreceptor^26^, and macrophages, where TLR2 and Gal-3 work together to raise cytokine production in response to *Candida albicans*^27^.

In this report, we underscore the clinical association of Gal-3 expression with disease progression, illustrate localization in and secretion by OA chondrocytes and determine their response to carbohydrate-dependent cell surface binding of Gal-3, always in relation to Gal-1. This comparison to Gal-1 was extended to transcriptomic analysis and to the subsequent investigation on involvement of NF-κB activation in enhanced transcription of OA marker genes. Finally, by performing functional assays with mixtures of Gal-1 and -3, we provide first information on the functional cooperation between galectins in OA.

## Results

### Gal-3 Localization in Clinical Specimens and OA Chondrocytes

**Figure 1:**
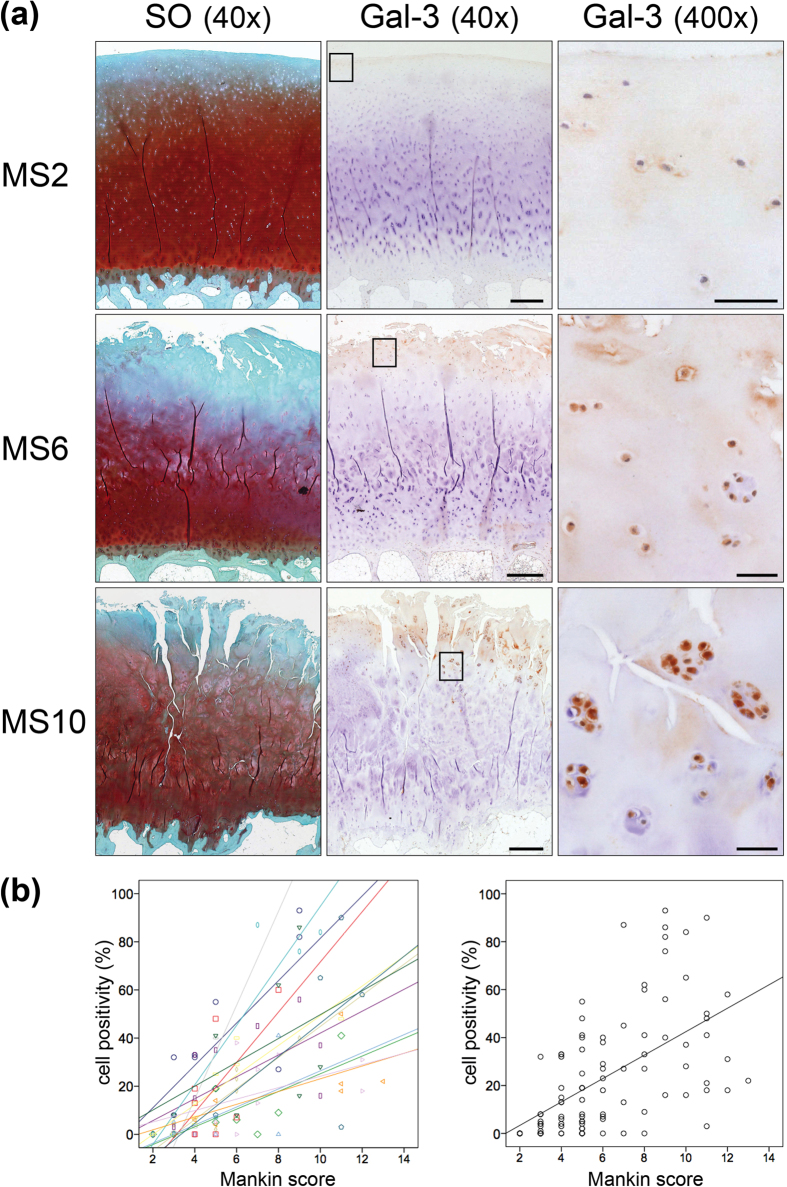
Gal-3 presence in articular chondrocytes correlates with cartilage degeneration. Histological sections of OA articular cartilage and subchondral bone (n = 13 patients) were stained with Safranin O (SO). From each patient, 3–9 regions of interest were graded using the Mankin score (MS). Consecutive sections were processed immunohistochemically and the percentages of Gal-3-positive chondrocytes were assessed at 40× and 400× magnification. Overview images (40×) were photomerged using Adobe Photoshop from single photographs. (**a**) Shown are exemplary series from mildly (MS2), moderately (MS6) and severely (MS10) degenerated cartilage regions stained with SO (left) and using an antibody against Gal-3 (middle, scale bar: 500 μm, squares indicate the regions illustrated at 400× magnification; right, scale bar: 50 μm). (**b**) Shown are scatterplots of Mankin scores versus percentages of Gal-3-positive chondrocytes with regression lines for each patient (left panel) and over all patients (right panel). Pearson correlation coefficients ranged from 0.43 to 0.94 (mean: 0.69 ± 0.18).

Having collected clinical material of OA articular cartilage and subchondral bone that covers the range of the Mankin score (MS), the measurement of cellular positivity at different levels of degeneration was possible. As exemplarily shown in Fig. 1a for mildly (MS2), moderately (MS6) and severely (MS10) degenerated regions, the percentage of Gal-3 positivity in the chondrocyte population increased steadily. As presented in scatterplots for each patient (Fig. 1b, left) and for the entire group (Fig. 1b, right), onset and progression of the disease were positively correlated with this parameter (p < 0.001, Wilcoxon signed rank test). In comparison, positivity for Gal-3 reached a maximum level of about 60% in linear regression, whereas a higher slope of the regression line and a maximum level of 100% chondrocyte positivity were seen for Gal-1^14^,^15^. Overall, both proteins appear to contribute to the previously reported increase of reactivity to the pan-galectin sensor asialofetuin^13^. Considering the role of the extracellular matrix (ECM) as bioactive scaffold to present galectins in a mode suited for potent activity^28^, we gave special attention to this aspect. Positivity for Gal-3 was found in the ECM of OA cartilage (Fig. 1 and Supplementary Fig. S1a). It was confined to zones with marked degradation, as indicated by the loss of Safranin O staining. Of note, the staining intensity for Gal-3 in the ECM was significantly increased in regions with high MS (Supplementary Fig. S1b), in line with the situation in OA chondrocytes.

**Figure 2:**
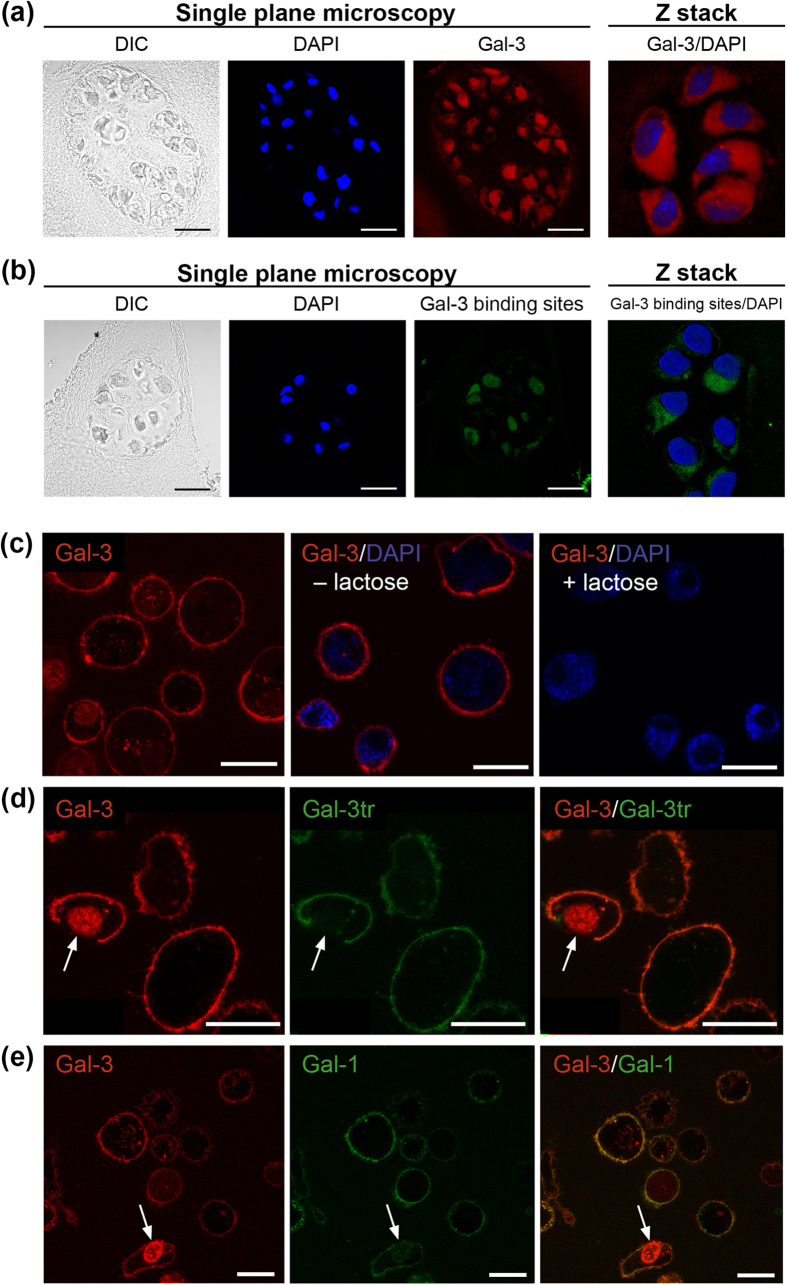
Localization of Gal-3 and its binding sites in OA chondrons and lactose-inhibitable binding of galectins to chondrocytes *in vitro*. (**a**) OA cartilage sections were processed with antibodies against Gal-3 (red) – followed by immunofluorescence detection using AlexaFluor555-labelled second-step antibodies – together with DAPI (blue) prior to analysis using laser scanning microscopy. Differential interference contrast (DIC) imaging was included. Scale bar: 20 μm. A series of eight images was recorded at 1 μm intervals to create a stack in the Z axis. Shown is the projection from the Z stack generated using ZEN software. (**b**) OA cartilage sections were processed with Gal-3-FITC (green) together with DAPI (blue) prior to analysis using laser scanning microscopy. DIC imaging was included. Scale bar: 20 μm. A stack in the Z axis was created, based on eight serial microphotographs. Shown is the projection from the Z stack established using ZEN software. (**c**) Cultured OA chondrocytes were trypsinised and resuspended prior to labelling with Alexa-Fluor555-labelled Gal-3 (Gal-3-555) (red) and DAPI (blue) at 4 °C for 10 minutes in presence or absence of 0.1 M β-lactose. After 10 minutes of incubation, cells were washed and analysed using laser-scanning microscopy, with the focus plane set to the centre of cells. Scale bars: 20 μm. (**d,e**) Cultured OA chondrocytes were trypsinised and resuspended prior to labelling with (**d**) Gal-3-555 (red) and AlexaFluor488-labelled (Gal-3tr-488) (green) or (**e**) Gal-3-555 (red) and Gal-1-488 (green) at 4 °C for 10 minutes. After 10 minutes of incubation, cells were washed and analysed using laser-scanning microscopy, with the focus plane set to the centre of cells. Co-localization is presented in separate and overlay images. Arrows mark the staining of cell nuclei of chondrocytes, whose cell membranes have lost their integrity, by (**d,e**) Gal-3-555 (red), but not by (**d**) Gal-3tr-488 (green) or (**e**) Gal-1-488 (green). Shown are the results from chondrocytes of one patient, representative for three independent experiments (n = 3 patients). Scale bars: 20 μm.

Extending our findings from immunohistochemical experiments, Gal-3 presence was visualized in OA chondrons using immunofluorescence and single plane microscopy (Fig. 2a). Z Stack projection identified Gal-3 to be consistently present throughout the cytoplasm of chondrocytes (Fig. 2a, right). When applying FITC-labelled Gal-3 to the fixed material as sensor for accessible binding sites, staining was obtained (Fig. 2b). It was less homogeneously distributed within the cytoplasm than the signal for Gal-3 presence. In fact, intracellular sites with reactivity for Gal-3 appeared to be restricted to defined cytoplasmic regions closely associated to the nucleus (Fig. 2b, right). Evidently, Gal-3 is present in chondrocytes *in situ*, and the detected correlation to the level of degeneration suggests that the lectin qualifies as effector in OA progression. To enable its activity in an auto-/paracrine manner, secretion of Gal-3 by chondrocytes is a prerequisite.

Tested in medium of OA chondrocytes, 0.11 ± 0.17 ng/ml (n = 7 patients) were detected in supernatants after 24 h of monolayer culture. This value tends to be smaller (p = 0.08, paired t-test) than the 1.11 ± 1.74 ng/ml (n = 7) found in cell cultures from the same patients under identical conditions for Gal-1. Comparable to the situation for Gal-1^15^, Gal-3 secretion was not altered by adding the cytokines IL-1β, IL-8 and tumour necrosis factor-α (TNF-α) as well as an IL-1β/TNF-α mixture (Supplementary Fig. S2). Also, their presence in the medium did not affect the level of LGALS3 mRNA in OA and non-OA chondrocytes, which excludes an influence of these cytokines on transcription of the Gal-3 gene (Supplementary Fig. S2). Because these key constituents of a pro-inflammatory environment fail to affect Gal-3 expression and secretion, we hypothesized that the lectin is an upstream effector, via carbohydrate-dependent cell surface binding. Indeed, fluorescent Gal-3 binds to viable chondrocyte surfaces in a lactose-inhibitable manner (Fig. 2c). When having access to the cell interior, the lectin is also reactive with nuclei (Fig. 2d, left). Removing the N-terminal tail of Gal-3 results in a product called truncated Gal-3 (Gal-3tr), which lacks this reactivity (Fig. 2d, center and right). Taken together, Gal-3 shares the capacity for lactose-inhibitable cell surface binding with Gal-1, as shown by two-colour staining in Fig. 2e. This series of results prompted us to examine whether addition of Gal-3 to the medium of cultured OA chondrocytes engenders changes in the level of mRNA or secretion of disease markers.

### Gal-3 Enhances Expression of Functional Disease Markers

**Figure 3:**
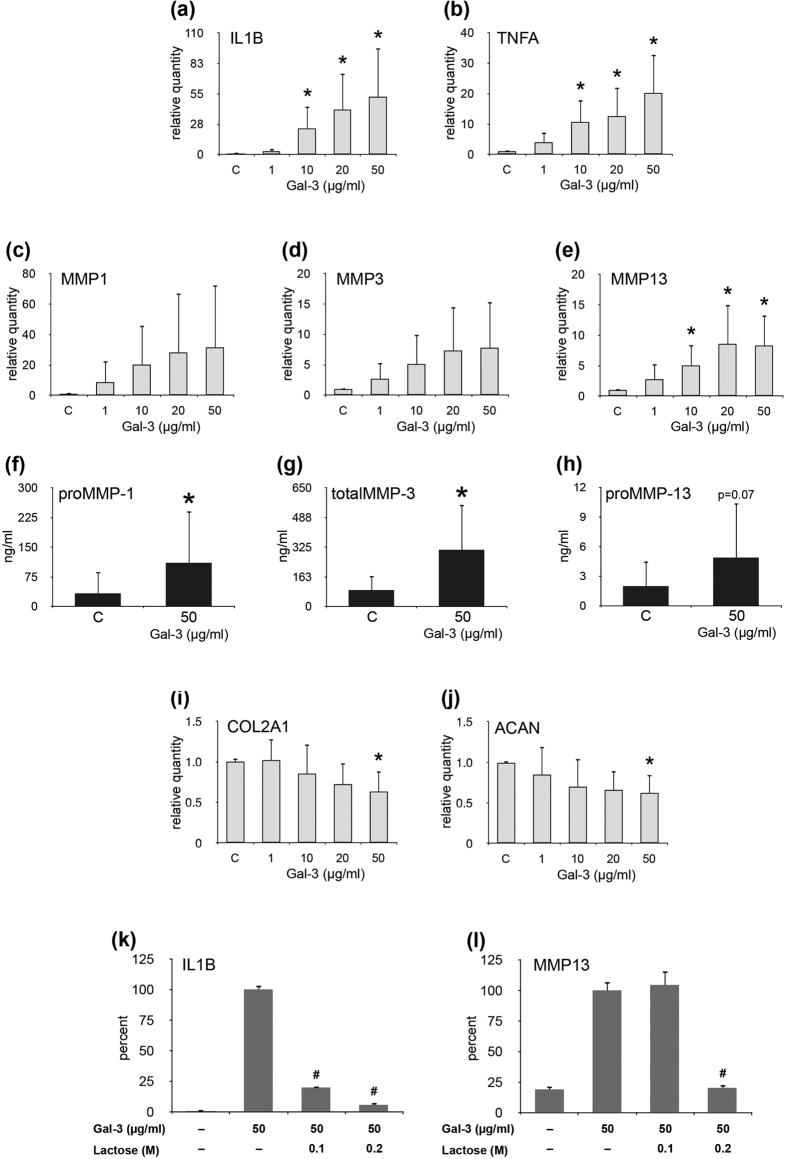
Gal-3 regulates functional OA markers via lactose-inhibitable binding. Chondrocytes of five OA patients were starved overnight prior to treatment with 1–50 μg/ml Gal-3 for 24h. Total RNA was isolated and relative mRNA levels of IL1B (**a**), TNFA (**b**), MMP1 (**c**), MMP3 (**d**), MMP13 (**e**), COL2A1 (**i**), and ACAN (**j**) were determined using RT-qPCR. Results are expressed as relative quantities (mean ± SD) with respect to untreated controls set to 1 (^*^p < 0.05, paired t-test vs control). (**f–h**) Concentrations (mean ± SD; ng/ml) of proMMP-1 (**f**), total MMP-3 (**g**), and proMMP-13 (**h**) were measured in cell culture supernatants of chondrocytes (n = 5 patients) treated with 50 μg/ml Gal-3 for 24 h using respective ELISAs (^*^p < 0.05, paired t-test vs control), mean ± SD; ng/ml). (**k,l**) Chondrocytes of three OA patients were starved overnight prior to treatment with 50 μg/ml Gal-3 or 50 μg/ml Gal-3 together with 0.1 M or 0.2 M lactose for 24 h. Total RNA was isolated and mRNA levels of IL1B (**k**) and MMP13 (**l**) were determined using RT-qPCR. Results of one representative patient are shown and data are expressed as percent (mean ± SD) with respect to Gal-3-treated cells set to 100% (^#^p < 0.05, unpaired t-test vs Gal-3-treated cells).

**Figure 4:**
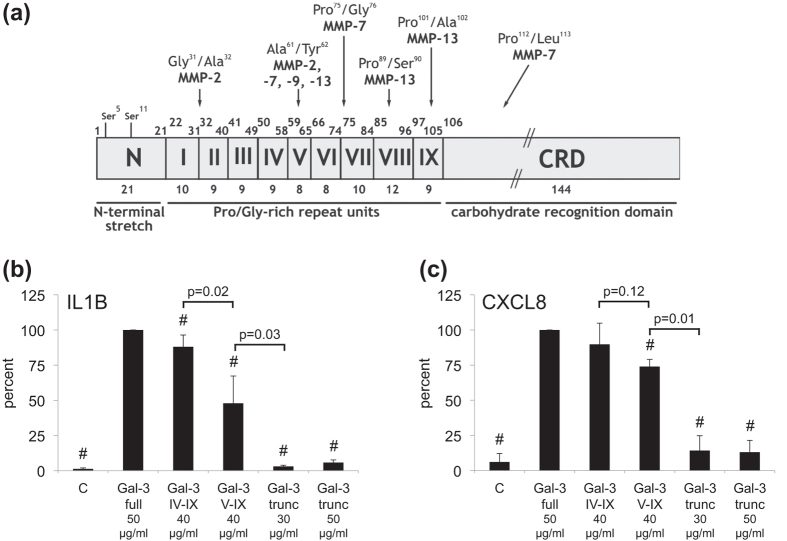
Structure-function relationship between Gal-3 and its activity in chondrocytes. (**a**) Structural organization of the trimodular design of human Gal-3, together with cleavage sites for MMPs, from ref. 45 with modifications. (**b,c**) Primary OA chondrocytes (n = 3 patients) were treated with full-length Gal-3, Gal-3 IV-IX, Gal-3 V-IX or Gal-3tr for 24 h. The concentrations of the variants were set to obtain equimolarity (with respect to CRD) for stimulation experiments. In addition, Gal-3tr was also applied at 50 μg/ml to examine dose-response effects. RT-qPCR assays for IL1B (**b**) and IL8 for CXCL8 (**c**) were performed. Results are expressed as percent (mean ± SD) of the activity of full-length Gal-3 (^#^p < 0.05 paired t test vs full-length Gal-3).

Supplementation of medium with LPS-free Gal-3 led to a concentration-dependent effect on gene expression (upregulation of IL1B, TNFA, MMP1, MMP3 and MMP13; downregulation of COL2A1 and ACAN; Fig. 3a–e and i,j, respectively) and on the level of secretion in the cases of matrix metalloproteinases (MMPs) (Fig. 3f–h). This effect was sensitive to the presence of the cognate sugar (Fig. 3k,l) which also serves as an additional control for the absence of LPS activity. Interestingly, despite its difference in protein design to homodimeric Gal-1, Gal-3 was also effective to elicit enhanced expression of disease markers. In order to pinpoint structural properties relevant for this activity, we engineered and tested variants of Gal-3 by reducing the length of the N-terminal tail, shown in Fig. 4a. Physiologically, cleavage sites for MMPs predestine the tail for proteolytic shortening, if not protected by aggregation on cell surfaces (Fig. 4a). The fully truncated form has apparently lost its elicitor activity, and removal of one repeat in the central region can be critical (Fig. 4b,c). The integrity of the second half of this repeat region appears necessary to modulate chondrocyte gene expression for the tested disease markers (IL1B, CXCL8). The results for these two selected cases encouraged us to answer the question on the dimension of Gal-3’s impact on regulation of gene expression by genome-wide microarray in combination with molecular analyses.

### Gal-3 Reprograms Gene Expression

**Figure 5:**
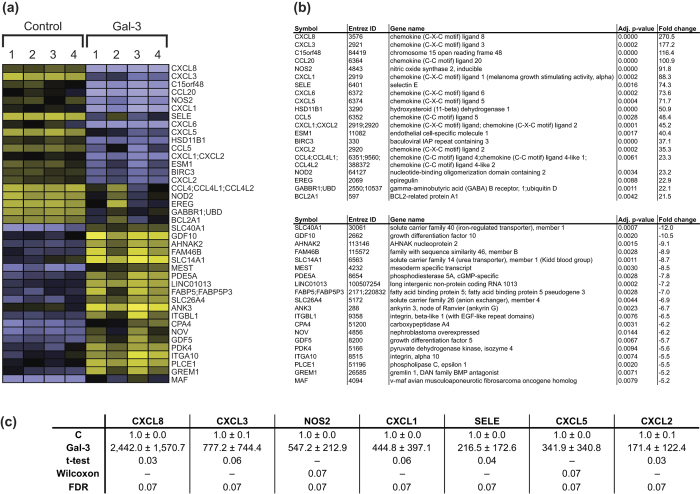
The most up- and downregulated transcripts in microarray analysis of Gal-3-stimulated chondrocytes. (**a**) OA chondrocytes (n = 4 patients; numbered with “1–4”) were starved overnight prior to treatment with 50 μg/ml Gal-3 for 24 h. Following microarray analysis, heat maps of the 20 most upregulated genes and the 20 most downregulated genes were plotted. (**b**) For these genes, the fold-changes of mRNA levels in Gal-3-treated vs untreated chondrocytes across all four patients were calculated. The p-values are also given. (**c**) The results of the microarray experiments were ascertained using RT-qPCR in the same RNA samples as used in the microarray analysis. Values are given as fold-changes with respect to untreated controls set to 1. Means and SDs were computed, normality of the data was analysed using Shapiro-Wilk test, and statistical significance of the data was examined using paired t test or Wilcoxon signed-rank test. In addition, multiple testing correction based on false discovery rate (FDR) was applied to adjust the raw p-values.

**Figure 6:**
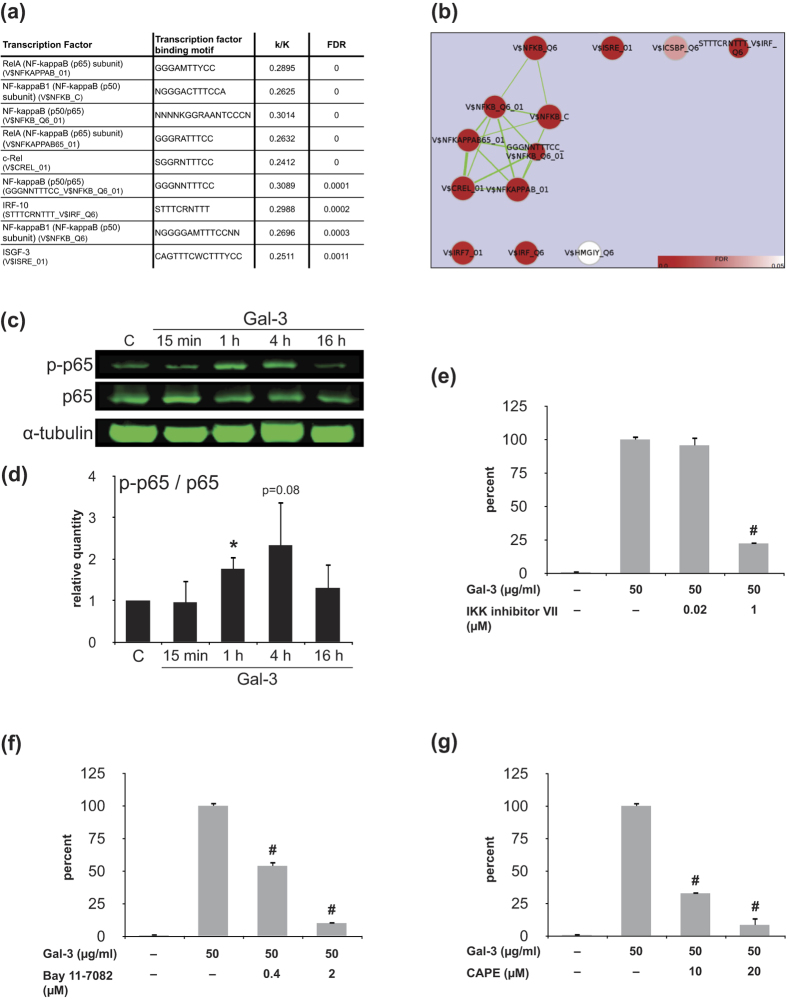
The activity of Gal-3 in chondrocytes is mediated via NF-κB signalling. (**a**) Top 10 results of GSEA analysis against the C3 (transcription factor target genes (TFT)) database in order of significance. K: number of genes harbouring the respective TFBS motif. k: number of overlapping genes induced by Gal-3. (**b**) Cytoscape and Enrichment Map visualization of significant C3-GSEA TFT results (FDR < 0.05). Enriched TFT gene sets are represented by nodes, which are grouped according to the gene similarity within each gene set. Nodes are coloured by FDR values (see calibration scaling, bottom right), where deeper red colours indicate increasing degree of significant enrichment. The size of the nodes is proportional to the total number of genes within each gene set, while the width of the edges is proportional to the gene overlap between the gene sets (number of shared genes), calculated using the overlap coefficient (cutoff: 0.5). (**c,d**) Data of quantitative Western blot analyses of p65 and phosphorylated p65 (p-p65) of extracts of OA chondrocytes (n = 3 patients), which were starved overnight and then treated with 50 μg/ml Gal-3 for 15 min, 1 h, 4 h, and 16 h. α-Tubulin was used as loading control. (**c**) Shown are the blots of one representative patient. (**d**) Shown are the ratios between p-p65 and p65 (normalized for α-tubulin) over time. Data are expressed as relative quantity in comparison to the untreated control set to 1 (n = 3 patients; *p < 0.05, paired t test vs untreated control). (**e–g**) OA chondrocytes were starved overnight and treated with 50 μg/ml Gal-3 in the presence or absence of inhibitors for 24 h prior to RT-qPCR analyses of IL1B mRNA levels. The graphs show the percent of IL1B expression relative to cells treated with Gal-3 (100%) in the presence of the NF-κB inhibitors IKK inhibitor VII (**e**), Bay 11-7082 (**f**), or CAPE (**g**). The experiment was repeated three times with cells from three different patients. Shown are the mean and SD (two technical replicates) from one representative experiment. Statistics were performed in comparison with Gal-3 activity in the absence of inhibitors. (^#^p < 0.05, unpaired t test vs Gal-3-treated cells).

Cell surface binding of Gal-3 to human OA chondrocytes caused a broad-scale change in their profile of gene expression. This change was fairly consistent in the four tested cell populations, as shown in Fig. 5a and b for the most up- and downregulated genes. Marked increases concerned a group of chemokine ligands (most notably CXCL8), the pro-inflammatory inducible nitric oxide synthase 2 (NOS2) and E-selectin (SELE, the docking site for leukocytes on inflamed endothelium). Independent RT-qPCR experiments for these mRNAs confirmed the array data (Fig. 5c). In addition, Supplementary Table S1 provides selected subsets of OA-related genes and glycogenes regulated by Gal-3. Gene Set Enrichment Analysis (GSEA) against C2 molecular signatures databases was performed to disclose functional implications. It revealed solid evidence for enhanced inflammation (Supplementary Fig. S3a–d). Metacore analysis of the Gal-3-upregulated gene sets disclosed connections with immune response and inflammation as well as with connective tissue, as well as autoimmune and arthritic joint diseases (Supplementary Fig. S3e–g). Using the C3 transcription factor targets (TFT) database for GSEA to delineate regulation of genes with common binding sites for transcription factors, an enrichment of genes harbouring NF-κB binding sites was unveiled (Fig. 6a). As graphically depicted in Fig. 6b, a significant proportion of the genes under the control of Gal-3 harbour such sites. Taken together, the findings of these bioinformatic analyses intimated an active involvement of NF-κB in the Gal-3-mediated upregulation of gene expression.

The emerging concept of NF-κB activation and participation was next experimentally verified. Upon stimulation of OA chondrocytes with Gal-3, phosphorylation of p65 was transiently increased (Fig. 6c,d). When determining the level of mRNA from IL1B gene transcription in the presence of three site-specific NF-κB inhibitors (IKK inhibitor VII, Bay-117082, CAPE), marked and dose-dependent blocking of the positive response by more than 75% was seen in each case (Fig. 6e–g). These data support the participation of NF-κB in the upregulation of transcription of Gal-3-responsive genes. Hereby, they establish a mechanistic similarity to the activity of Gal-1 in OA chondrocytes^15^. This finding prompted us to compare the responses to Gal-1 and -3.

### Gal-3 Cooperates with Gal-1 to Enhance Effector Expression for Degradation

**Figure 7:**
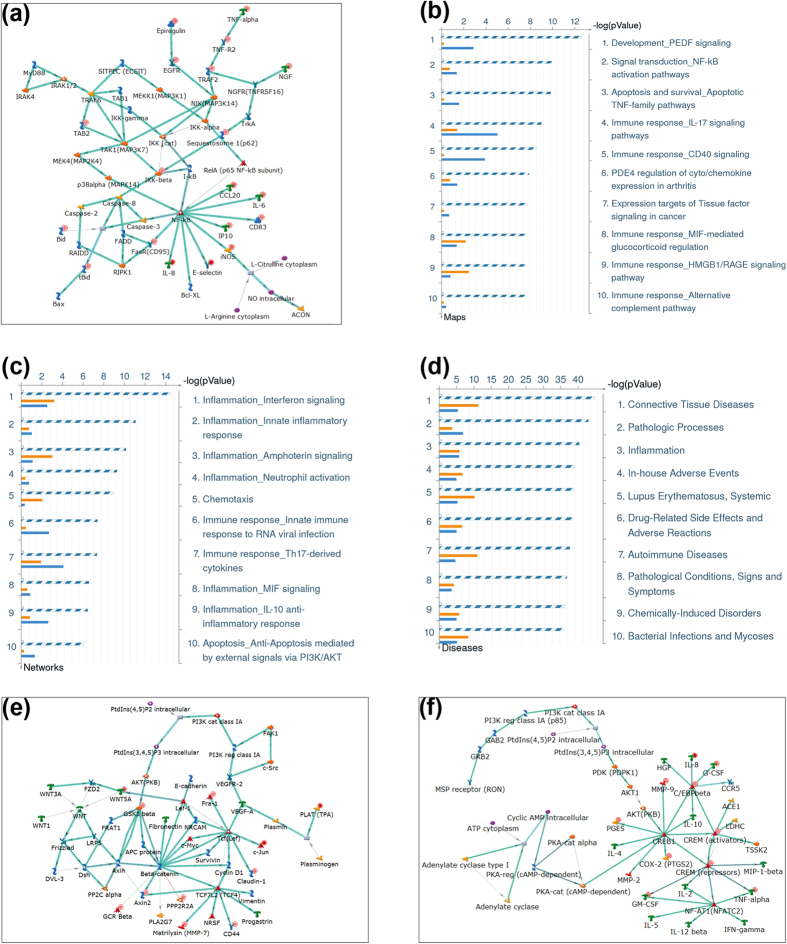
Bioinformatic comparison of the gene sets regulated by Gal-3 and Gal-1. (**a**–**d**) Pathway enrichment results using MetaCore’s “Compare Experiments” workflow obtained with genes consistently upregulated in both Gal-3-treated chondrocytes and Gal-1-treated chondrocytes (≥2-fold, p < 0.05). (**a**) Top scored network. Thick cyan lines indicate sections of canonical pathways. Upregulated genes are marked with red circles. (**b–d**) Blue/white striped bars: genes upregulated in both experiments. Orange bars: genes uniquely upregulated in Gal-3-treated chondrocytes. Blue bars: genes uniquely upregulated in Gal-1-treated chondrocytes. (**b**) Top10 scored Canonical Pathway maps. (**c**) Top10 scored Process Networks. (**d**) Top10 scored Diseases. (**e**,**f**) Pathway enrichment results using MetaCore’s “Compare Experiments” workflow obtained with genes uniquely upregulated in (**e**) Gal-3-treated chondrocytes or (**f**) Gal-1-treated chondrocytes (≥2-fold, p < 0.05). Presented are the top-scored networks using genes uniquely upregulated in chondrocytes treated with Gal-3 (**e**) or Gal-1 (**f**).

Comparing the gene sets, which were upregulated (≥2-fold, p < 0.05) in Gal-3- and in Gal-1-treated chondrocytes, revealed a high level of congruence. In particular, many NF-κB target genes were upregulated by both galectins (Fig. 7a). In terms of canonical pathways, an overlap by inducing immune response and NF-κB signalling was found (Fig. 7b). As consequence, inflammation was the most prominent commonly upregulated process network (Fig. 7c). Thus, it did not come as a surprise that a pronounced cooperation of Gal-1 and Gal-3 in triggering pathologic processes, with a particular focus on autoimmune and connective tissue diseases, was detected (Fig. 7d). Two areas of non-overlapping activity were identified: Gal-3 – but not Gal-1 – affected Wnt/β-catenin signalling (Fig. 7e). In contrast, genes uniquely induced by Gal-1 are under the control of cAMP response element-binding protein (CREB)-related signalling (Fig. 7f). When especially looking at glycogenes, transcription of the sialyltransferase gene for α2,3-sialylation of the core of *O*-glycan disaccharide (Thomsen-Friedenreich antigen), a potent Gal-3 ligand of mucins, is upregulated in concert, as are potential binding partners for galectins, i.e. α_5_β_1_-integrin (Supplementary Table S1). In view of an orchestrated action of galectins, the observed positive effect of Gal-3 on expression of Gal-8 is of interest for further studies (Supplementary Table S1).

**Figure 8:**
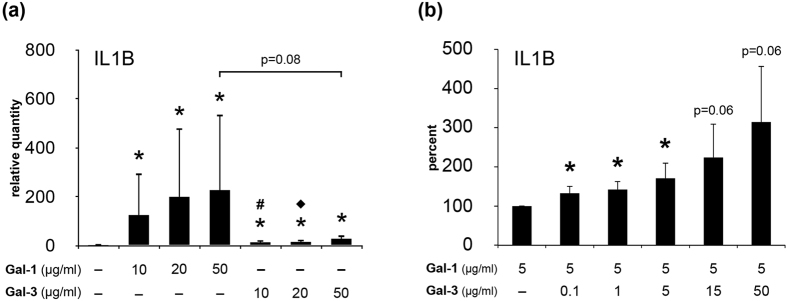
Functional cooperation between Gal-3 and Gal-1 activities in chondrocytes. (**a**) Chondrocytes of five OA patients were starved overnight prior to treatment with 10–50 μg/ml Gal-3 or 10–50 μg/ml Gal-1 for 24 h. Total RNA was isolated and mRNA levels of IL1B were determined using RT-qPCR. Results are expressed as relative quantities (mean ± SD) with respect to untreated controls set to 1 (^*^p < 0.05 vs Control; ^#^p < 0.05 vs 10 μg/ml Gal-1; ^♦^p < 0.05 vs 20 μg/ml Gal-1; Wilcoxon signed rank test). (**b**) Chondrocytes of three OA patients were starved overnight prior to treatment with mixtures of 5 μg/ml Gal-1 with Gal-3 for 24 h. Total RNA was isolated and mRNA levels of IL1B were determined using RT-qPCR. Results are expressed as percent (mean ± SD) with respect to cells treated with 5 μg/ml Gal-1 set to 100% (^*^p < 0.05 vs cells treated with 5 μg/ml Gal-1; paired t test).

Having described these similar response profiles on the level of transcriptional regulation, it was tempting to test the hypothesis of a functional cooperation. As illustrated in Fig. 8a for IL1B gene expression, Gal-1 was more active as elicitor than Gal-3, when assayed in parallel in OA chondrocyte cultures. The same finding was further extended for MMP13 (data not shown). Considering the differences in slope in regression analysis for the correlation of chondrocyte positivity and cartilage degradation in clinical specimens, Gal-1 is present at a higher concentration *in situ* than is the case for Gal-3 (as shown in ref. 15 and Fig. 1, respectively). In the course of degeneration, these concentrations likely are at a sub-saturating level. Thus, we set the value of Gal-1 at 5 μg/ml and added Gal-3 in different quantities, especially at low Gal-3 concentrations of 0.1 and 1 μg/ml. Thereby, the assays were designed to reflect pathophysiological conditions. As shown in Fig. 8b, a significant increase by the addition of Gal-3 occurred in both cases. When tested at a saturating Gal-1 concentration (50 μg/ml), no enhancement by Gal-3 was expectedly seen (not shown). Notably, we have herewith discovered a functional cooperation between these two endogenous lectins under conditions of progressing cartilage degeneration.

## Discussion

This report establishes the functional significance of Gal-3 as a broad-spectrum upstream effector in OA. As measured immunohistochemically in clinical specimens, immunocytochemically in OA chondrocyte cultures^14^ and ELISA-wise in culture supernatants, the lectin is present intracellularly, as it is secreted and can bind to the cell surface in a lactose-inhibitable manner. This cell association is the prerequisite for auto- and paracrine routes of cell regulation. Fittingly, when monitoring the course of the disease, a positive correlation between cartilage degeneration and Gal-3 positivity in chondrocytes and the ECM was found. In principle, the lectin can thus bind counterreceptor(s) within the cell and at the cell surface, what we demonstrated by using fluorescent protein as the optimal probe. Carbohydrate epitopes such as Lacdi*N*Ac on *N*-glycans presented by OA chondrocytes^13^ or clustered core 1 disaccharide/core 2 structures on *O*-glycans can serve as docking sites, whereas intracellular proteins such as the anti-apoptotic protein bcl-2 are further binding partners^29^,^30^,^31^,^32^,^33^. Cytoplasmic Gal-3 could thus maintain OA chondrocyte survival^22^,^34^, hereby sustaining the source for a pro-degradative/inflammatory microenvironment. Already at low concentrations in the 1–10 μg/ml range, the lectin was active as inducer of pro-inflammatory cytokines and MMPs. Of note, work on Gal-1 as molecular trigger of T cell death via galectin-dependent surface binding had disclosed that presentation by the ECM can increase the galectin’s potency^28^. Tested at such concentrations and lower, gene regulation in OA chondrocytes for the major aggrecanase in proteoglycan degradation, i.e. ADAMTS5^35^, had been reported to be highly sensitive to Gal-3 presence with peaks of increase (p < 0.0002) at 0.25 and 5 μg/ml^24^. The concentration of 5 μg/ml Gal-3 was also active to synergize with TNF-α in pro-inflammatory induction in human notochordal nucleus pulposus tissue specimen^36^. Additionally examined by transcriptomics at the whole-genome level, Gal-3 proved to be a potent elicitor of a pro-degradative/inflammatory signature. Systematic bioinformatic analyses and the use of site-specific inhibitors disclosed activation by NF-κB. Because cellular positivity *in situ* reached a calculated mean of about 60% at highest MS, an auto- and paracrine effector mechanism is likely that is simulated *in vitro* by adding Gal-3 to cultures of OA chondrocytes.

In addition to chemokines and MMPs, inducible NO synthase (iNOS) is high on the list of regulated proteins. NO as effector will promote cartilage breakdown and cause pain, also involved in oxidative damage and assisting cytokines in driving chondrocytes into apoptosis and altering cell surface glycosylation^37^,^38^. In this case, the Gal-3-induced increase of IL-1β may even lead to secondary enhancements. In view of the natural complexity of downstream pathways, activation of the NF-κB pathway has also been shown to reduce presence of the microRNA-26a-5p^39^. This result suggests the possibility that this regulatory route can be added to the effector branches of Gal-3. The expression of the lectin itself was not sensitive to pro-inflammatory cytokines in our assays. Such a responsiveness is known for other cell types, e.g. chronic wound edge (but not non-involved) fibroblasts, in which TNF-α even reduces LGALS3 expression (p = 0.032)^40^. In the tested OA chondrocytes, it was also not subject to auto-regulation, despite the presence of two putative binding sites for NF-κB in the promoter of the human gene for Gal-3^41^. However, exposure to sodium nitroprusside, which impairs NF-κB activation in human OA chondrocytes, led to a decrease in Gal-3, further exacerbated by three types of NF-κB-targeting inhibitors^34^. When looking at other cell types, Gal-3 is an upstream regulator of the NF-κB pathway in acute lymphoblastic leukemia^42^ and ovarian cancer cells^43^, also critically responsible for stimulation of IL-6 and CCL5 production/secretion from synovial fibroblasts of RA and – to a lesser extent – of OA patients^44^. Its truncated form appears to be able to reduce phosphorylation of p65/IκB in multiple myeloma and ovarian cancer cells^43^. In our assays, this natural variant was not active as elicitor and did not interfere with galectin activity (not shown). As previously seen in the case of regulating neuroblastoma cell (SK-N-MC) growth^45^, shortening the collagen-like repeat section around the cleavage site for MMPs-2, 7, -9, and -13 can be critical for the activity of Gal-3 on cells, likely by impairing self-association on a polyvalent surface. Thus, Gal-3 is an effector of disease progression sui generis for OA via NF-κB activation.

Importantly, our report teaches a second salient lesson by initiating a functional study of the galectin network. The apparent upregulation of galectins within OA pathogenesis, which we detected for Gal-1 and Gal-3^14^,^15^, encouraged to list the transcription factors, which are reactive to shared sequences as putative binding sites in both promoters and in introns (Supplementary Table S2). This compilation provides the basis for inferring the underlying mechanisms of increased transcription (to complement the previous documentation for Gal-1^15^, we also add the computationally detected TFBS of the Gal-3 promoter in Supplementary Table S3). (Patho)physiologically, different galectins can therefore be present at the same time in a tissue. However, the analysis of the functional significance of galectin expression is currently mostly confined to single members of the family. In the cases of Gal-1 and -3, the chimera-type protein is up to now viewed as antagonist of the negative growth regulator Gal-1 in neuroblastoma (SK-N-MC) and pancreas carcinoma (Capan-1) cells *in vitro*, in the latter case under the control of the p16^INK4a^ tumour suppressor^46^,^47^. In the context of OA chondrocytes, however, we here discovered a functional cooperation between galectins. As elicitors, they have a broad overlap in the gene-activation signature, in stark contrast to their opposite actions in RA. Additive effects are tangible in OA regarding the expression of various chemo- and cytokines, iNOS, E-selectin (favouring extravasation of leukocytes together with the chemoattractant capacity of Gal-3, hereby counteracting a respective anti-inflammatory activity of Gal-1^48^), TLR2 and also glycogenes such as α2,3-sialyltransferase I. This enzyme works on the core 1 *O*-glycan disaccharide and is so far reported to be upregulated by pro-inflammatory cytokines^49^,^50^.

Additionally, cases where genes are uniquely induced by either Gal-3 or Gal-1 (Supplementary Table S1) may well be interpreted as an extension of the combined profile of activities. The complementary upregulation of integrins, for example, could be pro-apoptotic, via caspase-8 activation^47^, or anti-apoptotic, sustaining marker release by Gal-3 by enhanced cell adhesion^51^. Complementary aspects of activity of both galectins were also detected by bioinformatic pathway analysis, indicating the independent induction of Wnt/β-catenin and CREB signalling, respectively.

Providing direction for further work on the galectin network in OA, the documented upregulation of Gal-8 and also galectin-related protein, a gene with unusually strong positive selection^52^, warrants further analysis. This conclusion is further supported by the previously reported immunohistochemical data on tandem-repeat-type Gal-4 and Gal-8 in OA specimens^14^. Future studies should also focus on the contribution of galectins to OA-related pathomechanisms besides inflammation and on the role of the cartilage ECM. Since the ECM can serve as bioactive scaffold for galectin (and also cytokine) presentation^28^, its structural status of integrity may also contribute to galectin activities. Similarly of interest is the molecular identification of the counterreceptor(s) and their signalling routes, on the levels of the scaffold (protein/lipid) and the glycosylation, in OA and non-OA (normal) cells. Considering glycosylation, the possibility to keep local NF-κB activation at bay by involving inhibitory receptors dampening inflammatory responses, as seen in bacterial infections^53^, can open a promising route for intervention with disease progression. Such a beneficial outcome may also be envisioned by using bifunctional compounds targeting and blocking galectins and MMPs^54^, their local efficiency most properly assessed histochemically^55^.

## Materials and Methods

### Galectins

Gal-1 and -3 as well as Gal-3 variants with shortened N-terminal tail were produced and purified as described previously^15^,^45^,^56^. Fluorescent galectins were prepared under activity-preserving conditions and rigorously checked for maintained activity as described^57^.

### Clinical specimens

Human articular cartilage specimens were obtained from OA patients who underwent total knee replacement surgery. Non-OA cartilage specimens were obtained post-mortem from individuals without signs of arthritic cartilage degeneration. The experimental protocols of this study were approved by the ‘Ethics Committee of the Medical University of Vienna’ (EK-No: 1065/2011, 203/2003). Written informed consent was obtained from all study participants. All methods were carried out in accordance with the approved guidelines and the Declaration of Helsinki.

### Histological assessment

Tissue specimens from femoral condyles of 13 OA patients (8 female, 5 male; age range 50–81 years) were collected to provide a wide range of cartilage degeneration and were processed for histological grading and immunohistochemistry as recently outlined^15^. In brief, paraffin sections were stained with Safranin O and 3–9 regions per patient (91 in total) were graded according to the MS. Consecutive sections were assessed by two independent observers for the percentages of Gal-3-positive chondrocytes in the regions of interest after immunohistochemical staining (as described below).

### Immunohistochemistry

Immunohistochemical staining for Gal-3 was performed according to standardised protocols^14^. In brief, deparaffinised cartilage sections were incubated with rabbit polyclonal antibody against human Gal-3, which was detected by a commercial second-step reagent with horseradish peroxidase as signal-generating part. Staining was developed using 3,3′-diaminobenzidine tetrahydrochloride hydrate and H_2_O_2_ as substrates. Counterstaining was performed using Mayer’s hemalum solution. Negative controls were performed by omission of primary antibody and by the use of irrelevant antibodies.

### Chondrocyte culture

Articular chondrocytes were isolated from femoral condyles and tibial plateaus and kept in culture following established protocols^50^. Only freshly isolated and seeded chondrocytes without subculturing were used for all assays. Upon 90% confluency, chondrocytes were serum-starved overnight. To induce pro-inflammatory conditions, chondrocytes were stimulated with IL-1β, TNF-α, and/or IL-8 (all from Biolegend). Further treatments occurred in the presence or absence of lactose with human recombinant Gal-1, Gal-3, its proteolytically truncated form or variants of Gal-3 in which the number of collagen-like repeats has been reduced, alone or in combination, for the indicated time periods. For the inhibition of NF-κB pathway components, three inhibitors (Bay 11-7082, IKK inhibitor VII, CAPE; all from Merck) acting at distinct sites were added to the chondrocyte cultures one hour prior to the stimulation with recombinant Gal-3.

### Immunofluorescence staining of cartilage tissue

Following tissue processing, the slides were washed twice with Tris-buffered saline (TBS) for five minutes and once in 3% H_2_O_2_ in TBS for three minutes and again twice with TBS. Blocking was performed using donkey serum (1:10 diluted with TBS) for 30 minutes at room temperature. The slides were then incubated overnight at 4 °C with the anti-Gal-3-specific antibody (1:25 diluted with TBS), which is non-cross-reactive to other human galectins. The next day, the slides were washed twice with TBS and incubated for one hour at room temperature with the second-step antibody (anti-rabbit Alexa Fluor 555 made in donkey; 1:500 diluted with TBS; Life Technologies) together with 3 μg/ml DAPI. Afterwards, the slides were washed twice with TBS and incubated overnight at 4 °C with FITC-conjugated Gal-3 (1:100 diluted with TBS). On the following day, slides were washed twice with TBS and finally mounted for microscopy using Fluorsave Reagent (Merck). Images were obtained using laser scanning microscopy at an original magnification of 630× (LSM700; Carl Zeiss).

### Immunofluorescence staining of galectin binding sites

Cell suspension of 3 × 10^5^ cells in 50 μl PBS was prepared by trypsinization and centrifugation of monolayer chondrocyte culture. Cells were incubated with 50 μl PBS containing 5 μg Alexa488-labelled Gal-1, Alexa555-labelled Gal-3, and/or Alexa488-labelled Gal-3tr (and 10 ng DAPI) in the presence and absence of 0.1 M lactose for 10 minutes at 4 °C, to limit internalization of cell-bound galectins. Cells were thoroughly washed three times with PBS to remove unbound galectins. Cells were then immediately mounted for laser scanning microscopy without fixation.

### RT-qPCR

Isolation of total RNA, cDNA synthesis, and SYBR-green-based RT-qPCR experiments were performed as described^50^. In brief, total RNA was extracted from OA chondrocytes cultivated in 12-well plates using the innuPREP RNA Mini Kit (Analytic Jena), and each sample was examined for quantity and quality on NanoDrop 1000 and Agilent 2100 Bioanalyzer Nano LabChip (Agilent Technologies) prior to reverse transcription into cDNA. RNA integrity numbers were between 9.5 and 10. The protocols strictly followed the minimal guidelines for the design and documentation of qPCR experiments. A detailed qPCR checklist containing all relevant information is provided by the authors upon request. mRNA levels were calculated as quantities relative to the untreated control group, considering amplification efficiencies and normalization to succinate dehydrogenase complex, subunit A (SDHA), which had been identified as stable reference gene under the experimental conditions of this study.

### ELISA

Cell culture supernatants were collected from Gal-3-treated or untreated OA chondrocytes, centrifuged and stored at −80 °C. The levels of ADAMTS-4, proMMP-1, proMMP-13, and totalMMP-3 were determined by ELISA assays (ADAMTS-4: BosterBio; MMPs: R&D Systems) following the protocols provided by the manufacturers. Gal-3 levels were assessed in cell culture supernatants of pro-inflammatory cytokine-treated chondrocytes (R&D). ELISA standard curve ranges were 0.625–40 ng/ml (ADAMTS-4), 0.313–10 ng/ml (Gal-3), 0.156–10 ng/ml (proMMP-1 and total MMP-3), and 78–5,000 pg/ml (proMMP-13).

### Microarray

Articular chondrocytes were isolated from four female osteoarthritis patients (59–68 years). Cells were cultivated in 25 cm^2^ flasks, serum-starved overnight and then incubated with 50 μg/ml recombinant Gal-3 for 24 h. Following isolation and quality control of total RNA, GeneChip analysis was performed using 200 ng total RNA per sample as described^15^. The microarray data discussed in this publication have been deposited in the GEO database and are publicly accessible through GEO Series accession number GSE85254.

### Bioinformatic analyses

Gene set enrichment analysis (GSEA, v2.2.0,^58^) was performed using the normalized and filtered expression data against the Molecular Signatures Databases v5.0 (http://software.broadinstitute.org/gsea/msigdb) C2: CGP, KEGG, BIOCARTA, REACTOME and C3: TFT (transcription factor targets). Analysis was performed with 1,000 permutations of gene sets using the Signal2noise ranking metric. Enrichment results of the GSEA analysis against transcription factor targets (C3: TFT) were visualized in Cytoscape 3.2.1^59^ using the Enrichment Map plugin^60^. For comparison, the Gal-1 data set was downloaded from GEO (accession number: GSE68760) and processed in the same way as the results obtained for the Gal-3 dataset.

Pathway analysis was performed using the MetaCore (version 6.26) analytical software. Up- or downregulated genes (≥2-fold in Gal-3-treated and Gal-1-treated^15^ chondrocytes, respectively, adjusted p-value (FDR) < 0.05) were imported into MetaCore. Enrichment analyses (hypergeometric test) were run on four ontologies: pathway maps, GO processes, process networks, and diseases (by biomarkers). These ontologies were ordered according to their p-value, based on hypergeometric distribution. In addition, the “Analyze Network” algorithm was employed to build network modules associated with the upregulated genes (≥2-fold, p < 0.05) in Gal-3-and/or Gal-1-treated chondrocytes. Furthermore, a comparison between upregulated genes in Gal-1- and Gal-3-treated chondrocytes, respectively, was done using MetaCore’s “Compare Experiments” workflow in order to identify enrichments using genes consistently expressed in both experiments as well as those uniquely expressed in the two experiments.

### Promoter analysis

The proximal promoter region and the five introns of the gene for human Gal-3 were processed by the MatInspector software (Matrix Library 9.4) with settings to exclude low-quality hits, as performed for Gal-1 previously, to allow direct comparison^15^.

### Protein isolation, SDS-PAGE and Western blot

Proteins were extracted from OA chondrocytes cultured in 6-well plates and prepared for SDS-PAGE and subsequent Western blot analyses following previously described protocols^15^. In brief, cells were lysed with RIPA lysis buffer, supplemented with phenylmethylsulfonyl fluoride, sodium orthovanadate, and a protease inhibitor cocktail.

Proteins were separated using 5% stacking and 10% separating gels and were transferred to a nitrocellulose membrane (Amersham Protran; GE Healthcare Europe). After blocking, membranes were probed with anti-NF-κB p65 (1:1,000; rabbit monoclonal; Cell Signaling) and anti-phospho-NF-κB p65 (Ser536; 1:1,000; rabbit monoclonal; Cell Signaling), together with anti-α-tubulin (1:1,000; mouse monoclonal; Cell Signaling). Thereafter, membranes were incubated with DyLight 800 nm-labelled goat anti-rabbit IgG (1:15,000; Thermo Scientific). Finally, the immunoreactive protein bands were detected and quantified using the Odyssey Imager CLx (Licor). The ratios between phospho-p65 and p65 (normalized for α-tubulin) were calculated as relative quantities in comparison to the untreated controls set at 1.

### Statistical analyses

Correlation analysis between immunopositivity for Gal-3 and the MS was performed using SPSS 20.0. Pearson’s correlation coefficients were calculated for each patient separately, and Wilcoxon signed-rank test was performed to test whether the median correlation coefficient was different from 0. Statistical comparison of the microarray data was performed using the limma algorithm, and multiple testing correction based on false discovery rate (FDR) was applied to adjust the raw p-values. Statistical analyses of RT-qPCR, ELISA and quantitative Western blot data were performed using SPSS 20.0. Normality of the data was analysed using the Shapiro-Wilk test, and statistical significance of the data was delineated using paired or unpaired t-test or Wilcoxon signed-rank test. All analysis units (n) given in figure and table legends – unless stated otherwise – refer to the number of independent observations (biological replicates) underlying the respective descriptive statistics and statistical tests.

## Additional Information

**How to cite this article:** Weinmann, D. *et al*. Galectin-3 Induces a Pro-degradative/inflammatory Gene Signature in Human Chondrocytes, Teaming Up with Galectin-1 in Osteoarthritis Pathogenesis. *Sci. Rep.* **6**, 39112; doi: 10.1038/srep39112 (2016).

**Publisher's note:** Springer Nature remains neutral with regard to jurisdictional claims in published maps and institutional affiliations.

## Supplementary Material

Supplementary Information
