# Supplementary material for: Galectin-3 Induces a Pro-degradative/inflammatory Gene Signature in Human Chondrocytes, Teaming Up with Galectin-1 in Osteoarthritis Pathogenesis
Source: Sci Rep. 2016 Dec 16;6:39112. doi: 10.1038/srep39112 (PMC5159921; doi:10.1038/srep39112)
Supplement: Supplementary Information [file srep39112-s1.pdf]

Galectin-3 Induces a Pro-degradative/inflammatory Gene Signature in Human Chondrocytes, Teaming Up with Galectin-1 in Osteoarthritis Pathogenesis

Authors

Daniela Weinmann, Karin Schlangen, Sabine André, Sebastian Schmidt, Sonja M. Walzer, Bernd Kubista, Reinhard Windhager, Stefan Toegel, Hans-Joachim Gabius

Supplementary Figure S1.

The staining intensity of Gal-3 in the ECM of OA cartilage increases with tissue degeneration.

The histological regions (91 in total) used for the evaluation of cellular Gal-3 staining (Figure 1) were grouped into 3 categories based on the Mankin score (i.e., MS≤4, MS5-8, MS≥9). The staining intensity of Gal-3 in the ECM of these regions was evaluated microscopically by two observers and categorised into gradual stages, ranging from SI = 1 (negative) to SI = 4 (strong staining). (**a**) Shown are representative images of the 3 Mankin score categories presenting the respective median staining intensities (left image: SI=2.0; middle image: SI=2.5; right image: SI=3.5). Scale bars: 50 µm. (**b**) Shown is a dot plot of the Gal-3 staining intensities in the ECM of OA cartilage. Each dot represents the staining intensity of one evaluated histological region and the bars indicate the respective median values. Statistical analyses were performed using SPSS. Data were not normally distributed as determined by Shapiro-Wilk tests. Mann-Whitney U tests were performed to identify statistically significant differences between the study groups (*, $p<0.05$).

(a)

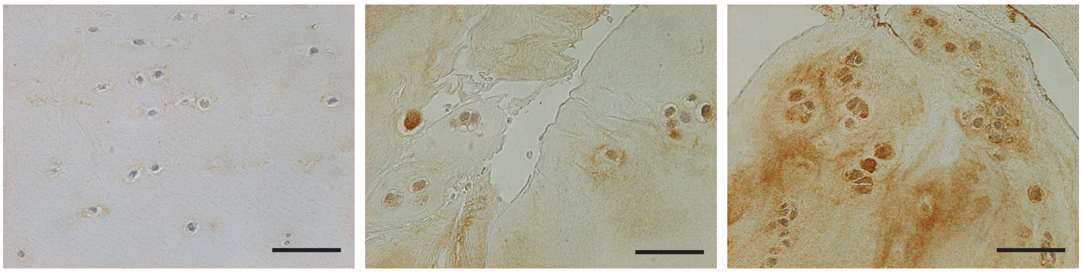

(b)

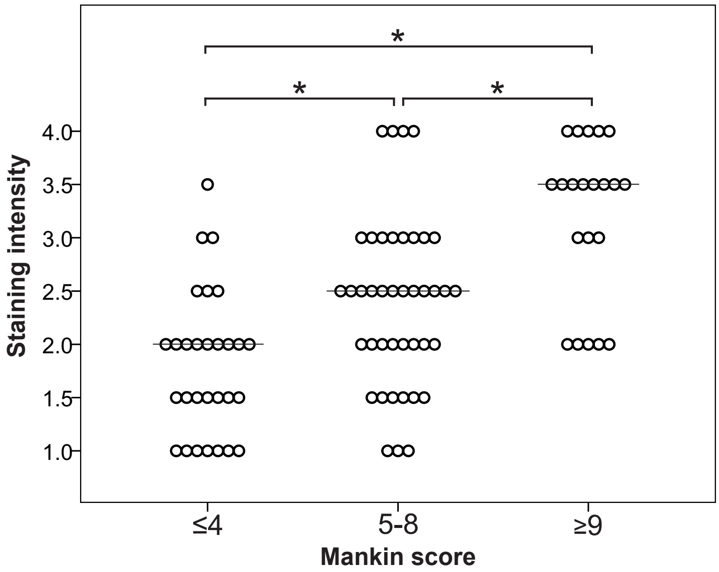

Supplementary Figure S2.

Gal-3 is expressed and secreted by cultured chondrocytes, but not induced by cytokines.

(**a**) Gal-3 secretion to cell culture medium was determined using ELISA. OA chondrocytes (n=4–11 patients) were starved overnight prior to treatment with 10 ng/ml IL-1β, 10 ng/ml TNF-α, 100 ng/ml IL-8, or a combination of 10 ng/ml IL-1β with 10 ng/ml TNF-α for 24 h and preparation of cell culture supernatants. Levels of secreted Gal-3 (ng/ml) are shown as mean ± SD. (**b**) LGALS3 mRNA levels of OA chondrocytes of five patients were determined using RT-qPCR. Cells were starved overnight prior to treatment with 10 ng/ml IL-1β, 10 ng/ml TNF-α, 100 ng/ml IL-8, or a combination of 10 ng/ml IL-1β with 10 ng/ml TNF-α for 24 h. Results are expressed as relative quantities (mean ± SD) with respect to untreated controls set to 1.
(**c**) LGALS3 mRNA levels of non-OA chondrocytes (n=4 individuals) were determined using RT-qPCR. Cells were starved overnight prior to treatment with 10 ng/ml IL-1β or 10 ng/ml TNF-α for 24 h. Results are expressed as relative quantities (mean ± SD) with respect to untreated controls set to 1.

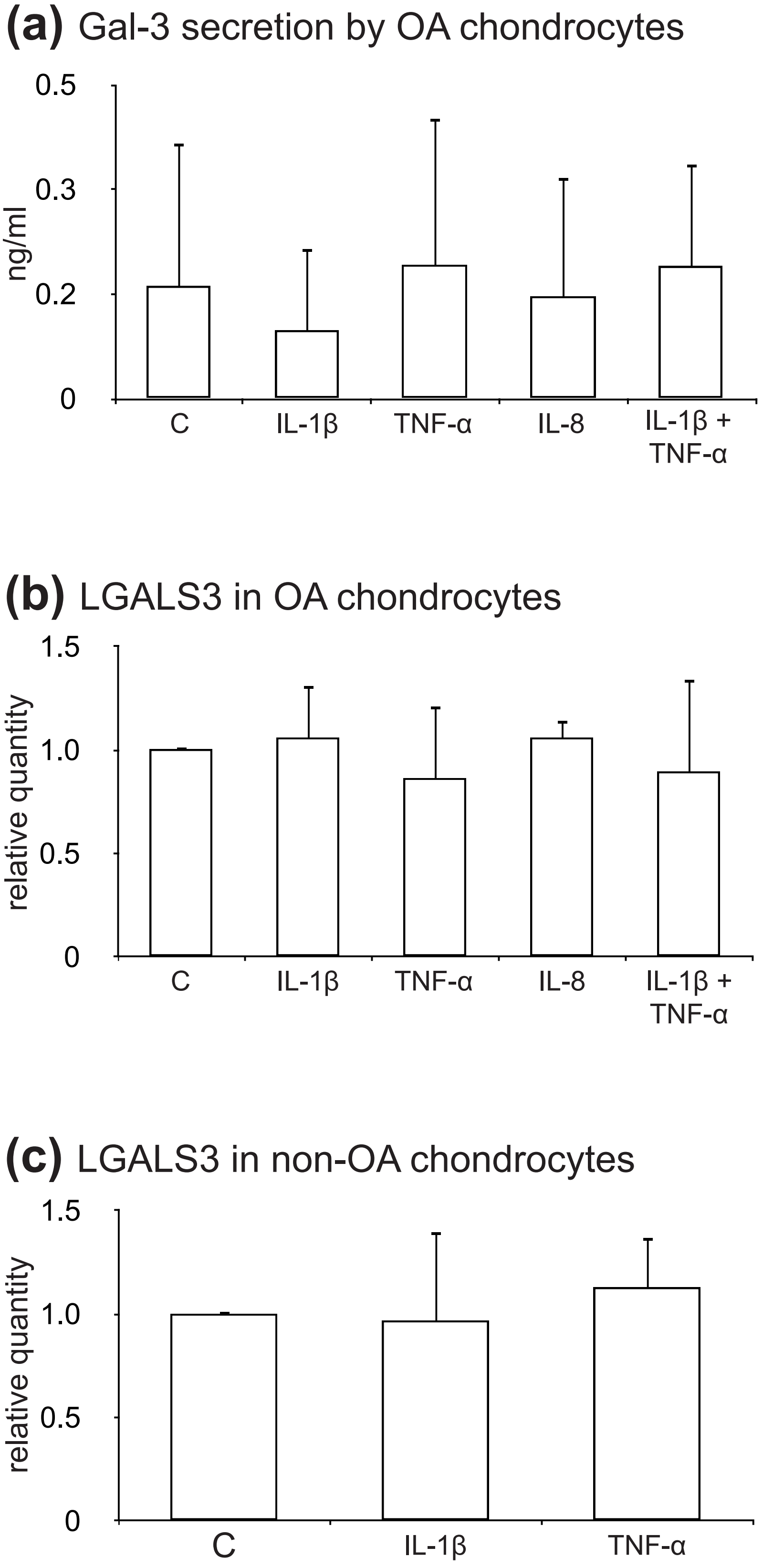
(a) Gal-3 secretion by OA chondrocytes
ng/ml
0.5
0.3
0.2
0
C
IL-1β
TNF-α
IL-8
IL-1β +
TNF-α
(b) LGALS3 in OA chondrocytes
relative quantity
1.5
1.0
0.5
0
C
IL-1β
TNF-α
IL-8
IL-1β +
TNF-α
(c) LGALS3 in non-OA chondrocytes
relative quantity
1.5
1.0
0.5
0
C
IL-1β
TNF-α

Supplementary Figure S3.

C2-GSEA and Metacore pathway enrichment analysis of genes induced in chondrocytes treated with Gal-3.

(**a**) C2-GSEA (REACTOME pathways; top 10 significant results in order of significance). K: number of genes included in REACTOME pathway. k: number of overlapping genes induced by Gal-3. (**b**) C2-GSEA (KEGG pathways; top 10 significant results in order of significance). K: number of genes included in KEGG pathway. k: number of overlapping genes induced by Gal-3. (**c**) C2-GSEA (perturbation signatures (CGP); top 10 significant results in order of significance). K: number of genes included in CGP signatures. k: number of overlapping genes induced by Gal-3. (**d**) C2-GSEA (BIOCARTA pathways; top 10 significant results in order of significance). K: number of genes included in BIOCARTA pathway. k: number of overlapping genes induced by Gal-3. (**e-g**) Results of the MetaCore pathway enrichment analysis using the upregulated genes in Gal-3-treated chondrocytes (≥2-fold, $p<0.05$). Presented are the top scored Canonical Pathway maps (**e**), Process Networks (**f**), and Diseases (**g**). (**h-j**) Results of the MetaCore pathway enrichment analysis using the downregulated genes in Gal-3-treated chondrocytes (≥2-fold, $p<0.05$). Presented are the top scored Canonical Pathway maps (**h**), Process Networks (**i**), and Diseases (**j**).

(a)

Reactome Pathway	k	K	k/K	FDR
PEPTIDE_LIGAND_BINDING_RECEPTORS	23	138	0.166	0
CHEMOKINE_RECEPTORS_BIND_CHEMOKINES	14	40	0.350	0
INTERFERON_ALPHA_BETA_SIGNALING	36	51	0.706	0
CYTOKINE_SIGNALING_IN_IMMUNE_SYSTEM	80	233	0.343	0
INTERFERON_SIGNALING	59	132	0.447	0
INTERFERON_GAMMA_SIGNALING	33	52	0.635	0
RIG_I_MDA5_MEDIATED_INDUCTION_OF_IFN_ALPHA_BETA_PATHWAYS	29	61	0.475	0
CLASS_A1_RHODOPSIN_LIKE_RECEPTORS	35	225	0.156	0
G_ALPHA_I_SIGNALLING_EVENTS	18	153	0.118	0
SIGNALING_BY_ILS	35	98	0.357	0

(b)

KEGG Pathway	k	K	k/K	FDR
CYTOKINE_CYTOKINE_RECEPTOR_INTERACTION	76	211	0.360	0
NOD_LIKE_RECEPTOR_SIGNALING_PATHWAY	19	55	0.346	0
CYTOSOLIC_DNA_SENSING_PATHWAY	18	38	0.474	0
RIG_I_LIKE_RECEPTOR_SIGNALING_PATHWAY	27	58	0.466	0
JAK_STAT_SIGNALING_PATHWAY	45	125	0.360	0
APOPTOSIS	35	83	0.422	0
CHEMOKINE_SIGNALING_PATHWAY	39	164	0.238	0
TOLL_LIKE_RECEPTOR_SIGNALING_PATHWAY	39	84	0.464	0
HEMATOPOIETIC_CELL_LINEAGE	21	75	0.280	0.002
EPITHELIAL_CELL_SIGNALING_IN_HELICOBACTER_PYLORI_INFECTION	26	64	0.406	0.003

(c)

CGP (perturbation) Signatures	k	K	k/K	FDR
ZHANG_RESPONSE_TO_IKK_INHIBITOR_AND_TNF_UP	119	199	0.598	0
GHANDHI_BYSTANDER_IRRADIATION_UP	51	71	0.718	0
GHANDHI_DIRECT_IRRADIATION_UP	60	93	0.645	0
HINATA_NFKB_TARGETS_KERATINOCYTE_UP	51	83	0.615	0
SANA_TNF_SIGNALING_UP	54	77	0.701	0
SEKI_INFLAMMATORY_RESPONSE_LPS_UP	50	70	0.714	0
ALTEMEIER_RESPONSE_TO_LPS_WITH_MECHANICAL_VENTILATION	56	109	0.514	0
ZHOU_INFLAMMATORY_RESPONSE_LIVE_UP	147	392	0.375	0
BROWNE_INTERFERON_RESPONSIVE_GENES	45	64	0.703	0
HINATA_NFKB_TARGETS_FIBROBLAST_UP	35	80	0.438	0

(d)

BIOCARTA Pathway	k	K	k/K	FDR
TNFR2_PATHWAY	12	18	0.667	0.001
HIVNEF_PATHWAY	16	57	0.281	0.010
41BB_PATHWAY	10	16	0.625	0.011
TID_PATHWAY	9	16	0.563	0.011
CD40_PATHWAY	10	15	0.667	0.011
RELA_PATHWAY	8	16	0.500	0.012
NFKB_PATHWAY	12	23	0.522	0.012
IL10_PATHWAY	6	17	0.353	0.015
INFLAM_PATHWAY	11	23	0.478	0.019
IL1R_PATHWAY	17	30	0.567	0.019

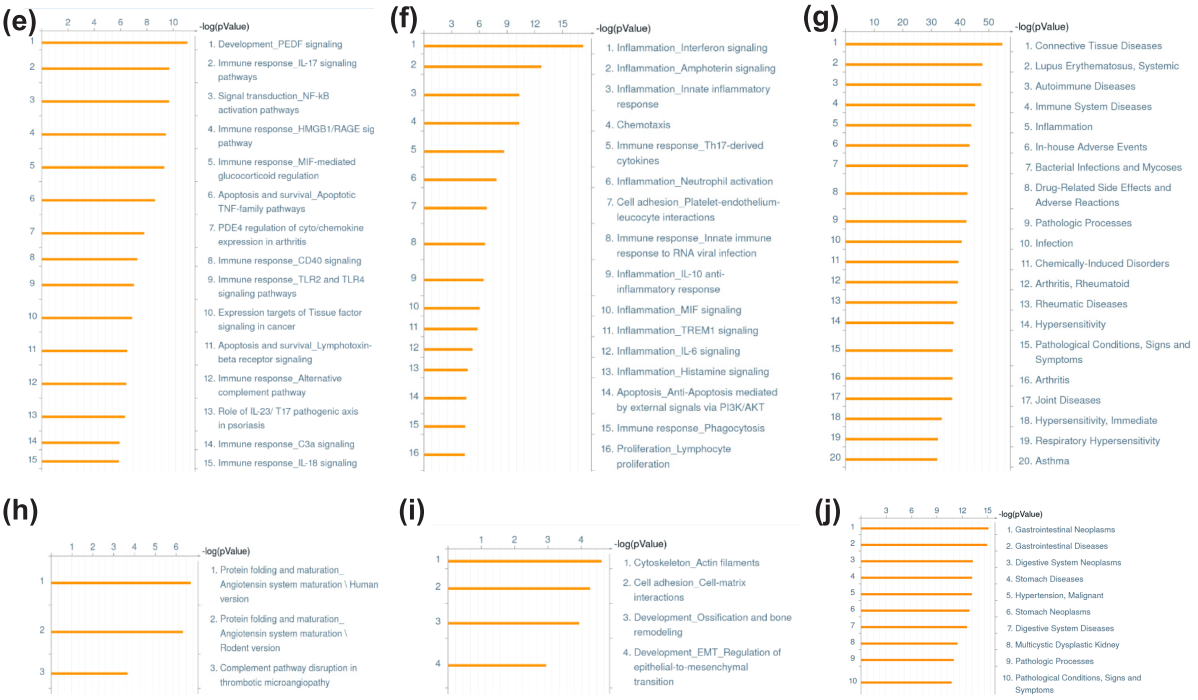

Supplementary Table S1.

Comparison of genes regulated by Gal-3 and Gal-1 as determined by microarray analysis (see Figure 4 for details).

The ratio between mRNA levels in Gal-3- or Gal-1-treated versus untreated chondrocytes as well as p-values, corrected for multiple hypothesis testing by the Benjamini-Hochberg method, are given. Data from Gal-1-treated chondrocytes were reproduced from GEO (accession number: GSE68760).

Cartilage- and OA-related genes

Symbol	Entrez ID	Gene name	Gal-3 BH	Gal-3 Ratio treated/untreated	Gal-1 BH	Gal-1 Ratio treated/untreated
COL7A1	1294	collagen, type VII, alpha 1	1E-03	**3.7**	1E-03	**4.73**
COL5A3	50509	collagen, type V, alpha 3	-	-	5E-02	**1.94**
COL22A1	169044	collagen, type XXII, alpha 1	-	-	6E-03	**1.83**
COL21A1	81578	collagen, type XXI, alpha 1	-	-	2E-02	**0.53**
COL1A1	1277	collagen, type I, alpha 1	-	-	1E-02	**0.51**
PCOLCE	5118	procollagen C-endopeptidase enhancer	-	-	3E-03	**0.47**
COL6A1	1291	collagen, type VI, alpha 1	-	-	1E-02	**0.42**
COL8A1	1295	collagen, type VIII, alpha 1	-	-	1E-02	**0.38**
PCOLCE2	26577	procollagen C-endopeptidase enhancer 2	-	-	2E-02	**0.37**
COL5A1	1289	collagen, type V, alpha 1	-	-	1E-02	**0.33**
COL5A2	1290	collagen, type V, alpha 2	-	-	2E-02	**0.29**
COL6A3	1293	collagen, type VI, alpha 3	-	-	2E-02	**0.25**
COL15A1	1306	collagen, type XV, alpha 1	-	-	2E-02	**0.24**
COL8A2	1296	collagen, type VIII, alpha 2	-	-	3E-03	**0.23**
COL12A1	1303	Collagen, type XII, alpha 1	-	-	2E-03	**0.19**
COL10A1	1300	collagen, type X, alpha 1	-	-	2E-02	**0.19**
COL14A1	7373	collagen, type XIV, alpha 1	-	-	1E-02	**0.18**
COL1A2	1278	collagen, type I, alpha 2	-	-	8E-03	**0.17**
COL11A1	1301	collagen, type XI, alpha 1	-	-	8E-03	**0.15**
ACAN	176	aggrecan	-	-	5E-02	**0.54**
LAMB3	3914	laminin, beta 3	2E-04	**19.0**	3E-04	**23.61**
LAMC2	3918	laminin, gamma 2	3E-02	**1.8**	3E-02	**3.48**
LAMA3	3909	laminin, alpha 3	-	-	3E-02	**2.36**
LAMB2	3913	laminin, beta 2 (laminin S)	-	-	3E-02	**0.64**
LAMC1	3915	laminin, gamma 1 (formerly LAMB2)	-	-	2E-02	**0.50**
LAMA4	3910	laminin, alpha 4	-	-	5E-03	**0.40**
LAMB1	3912	laminin, beta 1	-	-	5E-03	**0.17**
FNDC3B	64778	fibronectin type III domain containing 3B	1E-03	**2.8**	6E-04	**2.84**
FSD1L	83856	fibronectin type III and SPRY domain containing 1-like	3E-02	**1.5**	6E-03	**8.91**
DCN	1634	decorin	-	-	2E-02	**0.21**
MMP12	4321	matrix metallopeptidase 12 (macrophage elastase)	7E-04	**8.3**	7E-05	**15.85**
MMP10	4319	matrix metallopeptidase 10 (stromelysin 2)	1E-03	**8.0**	5E-04	**10.61**
MMP1	4312	matrix metallopeptidase 1 (interstitial collagenase)	5E-03	**5.0**	-	-
MMP13	4322	matrix metallopeptidase 13 (collagenase 3)	2E-04	**4.7**	-	-
MMP7	4316	matrix metallopeptidase 7 (matrilysin, uterine)	3E-02	**2.6**	5E-02	**1.45**
MMP9	4318	matrix metallopeptidase 9 (gelatinase B, 92kDa gelatinase, 92kDa type IV collagenase)	-	-	3E-02	**3.72**
MMP19	4327	matrix metallopeptidase 19	-		3E-02	**2.41**
MMP25	64386	matrix metallopeptidase 25	-	-	4E-02	**2.11**
ADAMTS4	9507	ADAM metallopeptidase with thrombospondin type 1 motif, 4	4E-02	**3.2**	4E-03	**6.37**
ADAMTS5	11096	ADAM metallopeptidase with thrombospondin type 1 motif, 5	-	-	1E-02	**0.19**
BMP2	650	bone morphogenetic protein 2	2E-04	**6.0**	2E-03	**5.72**
BMP6	654	bone morphogenetic protein 6	2E-02	**2.9**	2E-03	**4.32**
BMP5	653	bone morphogenetic protein 5	2E-02	**1.5**	1E-02	**2.90**
BMPR1B	658	bone morphogenetic protein receptor, type IB	-	-	4E-04	**3.40**
BMP1	649	bone morphogenetic protein 1	-	-	8E-03	**1.79**
BMP8A	353500	bone morphogenetic protein 8a	-	-	2E-02	**1.66**
BMP7	655	bone morphogenetic protein 7	-	-	2E-02	**1.57**
BMPR1A	657	bone morphogenetic protein receptor, type IA	-	-	2E-02	**0.24**
BMP4	652	bone morphogenetic protein 4	-	-	5E-03	**0.16**
TGFA	7039	transforming growth factor, alpha	-	-	3E-02	**1.64**
TGFB3	7043	transforming growth factor, beta 3	-	-	3E-02	**0.53**
TGFB2	7042	transforming growth factor, beta 2	-	-	1E-02	**0.46**
IGFBP7	3490	insulin-like growth factor binding protein 7	3E-02	**2.1**	-	-
IGFBP1	3484	insulin-like growth factor binding protein 1	5E-03	**1.9**	-	-
IGFBP6	3489	insulin-like growth factor binding protein 6	-	-	3E-02	**0.61**
IGF1R	3480	insulin-like growth factor 1 receptor	-	-	3E-02	**0.37**
IGFBP5	3488	insulin-like growth factor binding protein 5	-	-	1E-02	**0.29**
IGF2;INS-IGF2	3481;723961	insulin-like growth factor 2 (somatomedin A) /// INS-IGF2 readthrough transcript	-	-	4E-02	**0.28**
TNF	7124	tumor necrosis factor	-	-	7E-03	**23.56**
TLR2	7097	toll-like receptor 2	3E-04	**20.3**	3E-02	**5.94**
TLR1	7096	toll-like receptor 1	8E-03	**2.1**	-	-
TLR3	7098	toll-like receptor 3	3E-03	**2.0**	-	-
ITGB8	3696	integrin, beta 8	3E-03	**1.8**	-	-
ITGA5	3678	integrin, alpha 5 (fibronectin receptor, alpha polypeptide)	1E-02	**1.7**	1E-02	**1.8**
ITGB3	3690	integrin, beta 3 (platelet glycoprotein IIIa, antigen CD61)	2E-02	**1.6**		-
ITGA1	3672	integrin, alpha 1	1E-02	**1.5**	1E-02	**2.0**
ITGAM	3684	integrin, alpha M (complement component 3 receptor 3 subunit)	-	-	1E-02	**8.4**
CIB2	10518	calcium and integrin binding family member 2	-	-	2E-02	**0.6**
ITGA11	22801	integrin, alpha 11	-	-	2E-02	**0.5**
ITGB1	3688	integrin, beta 1 (fibronectin receptor, beta polypeptide, antigen CD29 includes MDF2, MSK12)	-	-	5E-02	**0.5**
ITGA3	3675	integrin, alpha 3 (antigen CD49C, alpha 3 subunit of VLA-3 receptor)	-	-	9E-03	**0.5**
ITGB5	3693	integrin, beta 5	-	-	5E-03	**0.4**
ITFG1	81533	integrin alpha FG-GAP repeat containing 1	-	-	4E-02	**0.4**
ITGAE	3682	integrin, alpha E (antigen CD103, human mucosal lymphocyte antigen 1; alpha polypeptide)	-	-	2E-02	**0.4**
ITGB1BP1	9270	integrin beta 1 binding protein 1	-	-	3E-02	**0.4**
ITGB3BP	23421	integrin beta 3 binding protein (beta3-endonexin)	-	-	8E-03	**0.3**
ITGA10	8515	integrin, alpha 10	-	-	1E-04	**0.1**
ITGBL1	9358	integrin, beta-like 1 (with EGF-like repeat domains)	-	-	1E-03	**0.1**

N-glycosylation

Symbol	Entrez ID	Gene name	Gal-3 BH	Gal-3 Ratio treated/untreated	Gal-1 BH	Gal-1 Ratio treated/untreated
FUT4	2526	fucosyltransferase 4 (alpha (1,3) fucosyltransferase, myeloid-specific)	3E-03	**2.5**	-	-
B4GALT1	2683	UDP-Gal:betaGlcNAc beta 1,4- galactosyltransferase, polypeptide 1	8E-03	**2.4**	-	-
ST3GAL4	6484	ST3 b-galactoside a2,3-sialyltransferase 4	1E-02	**1.8**	4E-02	1.5
B3GNT2	10678	UDP-GlcNAc:betaGal beta-1,3-N-acetylglucosaminyltransferase 2	2E-02	**1.6**	-	-
ALG13	79868	ALG13, UDP-N-acetylglucosaminyltransferase subunit	3E-02	**1.5**	-	-
B4GALT3	8703	UDP-Gal:bGlcNAc b1,4-galactosyltransferase, polypeptide 3	1E-02	**1.5**	4E-03	1.9
B3GNT7	93010	UDP-GlcNAc:betaGal beta-1,3-N-acetylglucosaminyltransferase 7	4E-02	**1.5**	-	-
B4GALT5	9334	UDP-Gal:bGlcNAc b1,4-galactosyltransferase, polypeptide 5	-	-	1E-02	1.9
FUT10	84750	fucosyltransferase 10 (a1,3-fucosyltransferase)	-	-	5E-02	0.7
ALG10;ALG10B	144245;84920	asparagine-linked glycosylation 10, a1,2-glucosyltransferase homolog (*S. pombe*); asparagine-linked glycosylation 10, a1,2-glucosyltransferase homolog B (yeast)	-	-	4E-02	0.7
FUT11	170384	fucosyltransferase 11 (a1,3-fucosyltransferase)	-	-	5E-02	0.6
ALG9	79796	asparagine-linked glycosylation 9, a1,2-mannosyltransferase homolog (*S. cerevisiae*)	-	-	3E-02	0.4
FUT8	2530	fucosyltransferase 8 (a1,6-fucosyltransferase)	-	-	1E-02	0.4
ALG10B	144245	asparagine-linked glycosylation 10, a1,2-glucosyltransferase homolog B (yeast)	-	-	2E-02	0.4
B4GALT2	8704	UDP-Gal:bGlcNAc b1,4- galactosyltransferase, polypeptide 2	-	-	2E-02	0.3
ALG6	29929	asparagine-linked glycosylation 6, a1,3-glucosyltransferase homolog (*S. cerevisiae*)	-	-	2E-02	0.3
ST6GAL1	6480	ST6 b-galactosamide a2,6-sialyltranferase 1	-	-	2E-02	0.1

O-glycosylation

Symbol	Entrez ID	Gene name	Gal-3 BH	Gal-3 Ratio treated/untreated	Gal-1 BH	Gal-1 Ratio treated/untreated
ST3GAL1	6482	ST3 b-galactoside a2,3-sialyltransferase 1	1E-03	**4.1**	2E-04	4.7
POMGNT1	55624	protein O-linked mannose b1,2-N-acetylglucosaminyltransferase	4E-03	**1.7**	1E-02	1.8
B3GAT1	27087	b1,3-glucuronyltransferase 1 (glucuronosyltransferase P)	-	-	4E-02	1.8
B3GAT2	135152	b1,3-glucuronyltransferase 2 (glucuronosyltransferase S)	-	-	4E-02	0.6
B3GNT9	84752	UDP-GlcNAc:bGal b1,3-N-acetylglucosaminyltransferase 9	-	-	1E-02	0.4
B3GALTL	145173	b1,3-galactosyltransferase-like	-	-	3E-02	0.4
B3GNT1	11041	UDP-GlcNAc:bGal b1,3-N-acetylglucosaminyltransferase 1	-	-	2E-02	0.3

Glycolipids

Symbol	Entrez ID	Gene name	Gal-3 BH	Gal-3 Ratio treated/untreated	Gal-1 BH	Gal-1 Ratio treated/untreated
A4GALT	53947	a1,4-galactosyltransferase	3E-03	**1.9**	2E-02	2.0
B3GNT5	84002	UDP-GlcNAc:bGal b1,3-N-acetylglucosaminyltransferase 5	-	-	2E-02	2.8
ST6GALNAC5	81849	ST6 (a-N-acetyl-neuraminyl-2,3-b-galactosyl-1,3)-N-acetylgalactosaminide a-2,6-sialyltransferase 5	-	-	3E-02	0.7
GBGT1	26301	globoside a1,3-N-acetylgalactosaminyltransferase 1	-	-	2E-02	0.7
ST6GALNAC6	30815	ST6 (a-N-acetyl-neuraminyl-2,3-b-galactosyl-1,3)-N-acetylgalactosaminide a-2,6-sialyltransferase 6	-	-	1E-02	0.6
B3GALNT1	8706	b1,3-N-acetylgalactosaminyltransferase 1 (globoside blood group)	-	-	1E-02	0.1

Mannosidases

Symbol	Entrez ID	Gene name	Gal-3 BH	Gal-3 Ratio treated/untreated	Gal-1 BH	Gal-1 Ratio treated/untreated
MAN1A1	4121	mannosidase, alpha, class 1A, member 1	2E-03	**2.6**	-	-
MAN1C1	57134	a-mannosidase, class 1C, member 1	-	-	3E-04	0.2
MANSC1	54682	MANSC domain containing 1	-	-	2E-02	0.2
MAN2B2	23324	a-mannosidase, class 2B, member 2	-	-	8E-03	0.4
MANBA	4126	a-mannosidase, b A, lysosomal	-	-	1E-02	0.4
MAN1B1	11253	a-mannosidase, class 1B, member 1	-	-	1E-02	0.4
MAN2A2	4122	a-mannosidase, class 2A, member 2	-	-	9E-03	0.4
MAN1A2	10905	a-mannosidase, class 1A, member 2	-	-	2E-02	0.5

Sialic acid (donor) synthesis/processing

Symbol	Entrez ID	Gene name	Gal-3 BH	Gal-3 Ratio treated/untreated	Gal-1 BH	Gal-1 Ratio treated/untreated
NANS	54187	N-acetylneuraminic acid synthase	-	-	4E-02	1.8
CMAS	55907	cytidine monophosphate N-acetylneuraminic acid synthetase	-	-	2E-02	0.6
SIAE	54414	sialic acid acetylesterase	-	-	1E-02	0.4

Lectins

Symbol	Entrez ID	Gene name	Gal-3 BH	Gal-3 Ratio treated/untreated	Gal-1 BH	Gal-1 Ratio treated/untreated
SELE	6401	E-selectin (CD62E)	2E-03	**74.3**	3E-07	323.3
LGALS8	3964	lectin, galactoside-binding, soluble, 8	1E-02	**1.8**	-	-
LMAN1	3998	lectin, mannose-binding, 1	2E-03	**1.7**	-	-
CLEC2D	29121	C-type lectin domain family 2, member D	3E-02	**1.5**	-	-
OLR1	4973	oxidized low density lipoprotein (lectin-like) receptor 1	-	-	2E-02	5.5
HSPC159	29094	galectin-related protein (GRP)	-	-	2E-02	3.2
KLRD1	3824	killer cell lectin-like receptor subfamily D, member 1	-	-	1E-02	2.1
CLEC4E	26253	C-type lectin domain family 4, member E	-	-	7E-03	1.9
KLRK1	22914	killer cell lectin-like receptor subfamily K, member 1	-	-	5E-02	1.6
LGALS3	3958	lectin, galactoside-binding, soluble, 3 (galectin-3)	-	-	3E-02	1.5
KLRC1	3821	killer cell lectin-like receptor subfamily C, member 1	-	-	4E-02	1.5
KLRG1	10219	killer cell lectin-like receptor subfamily G, member 1	-	-	3E-02	0.7
SELP	6403	P-selectin (granule membrane protein 140kDa, antigen CD62P)	-	-	1E-02	0.4
MLEC	9761	malectin	-	-	3E-02	0.4
CLEC3B	7123	C-type lectin domain family 3, member B	-	-	2E-03	0.3
CLEC3A	10143	C-type lectin domain family 3, member A	-	-	9E-04	0.2
COLEC12	81035	collectin sub-family member 12	-	-	2E-03	0.2

Supplementary Table S2.

Comparison of the computationally detected putative binding sites for transcription factors (TF) in the promoter regions and introns of the genes for human Gal-1 and Gal-3.

Regions analyzed were -2000 to +97 for Gal-1 and -2500 for Gal-3.

	**common TF for Gal-1 and Gal-3**
**promoter**	ACAAT, AHR_ARNT, AP1, ARE, ATBF1, ATF6, BARBIE, BCL6, BKLF, BRIGHT, BRN2, BRN3, BRN5, BSX, CDP, CDX1, CHOP, CHR, CP2, CREB2, CREL, CRX, CSRNP1, CTCF, δEF1, DLX1, DLX3, DREAM, E2F, E2F1, E2F3, E2F4, EBF1, EGR1, EKLF, EN1, FAST1, FREAC7, GATA1, GATA3, GC, GCM1, GFI1, GKLF, GSH1, GSH2, HDBP1_2, HFH1, HHEX, HIC1, HMGIY, HMX3, HNF1, HOXA5, HOXB8, HOXB9, HOXC13, HOXC4, HOXC9, HSF1, HSF2, IK3, INSM1, IR2_NGRE, IRF2, ISL2, JUNB, KAISO, KKLF, KLF6, KLF7, LEF1, LHX1, LHX2, LHX3, LRRFIP1, LTSM, MAFA, MAZR, MEF2, MEL1, MESP1_2, MIZ1, MSX, MTBF, MYBL1, MYOD, MZF1, NANOG, NBRE, NF1, NFAT, NFAT5, NF-κB65, NFY, NKX11, NKX12, NKX25, NKX31, NKX61, NMP4, OLF1, P53, PAX3, PAX6, PCE1, PHOX2, PIT1, PLAG1, PLAGL1, PPARG, PRDM1, PREB, PROP1, PSE, PURα, S8, SMAD3, SMARCA3, SOX3, SP1, SPI1, SPZ1, SREBP, SRF, STAT, STAT3, STAT5A, STAT6, TCF7L1, TCFAP2A, TLX1, VDR_RXR, VMYB, XBOX, YB1, ZBED4, ZBP89, ZBTB3, ZBTB7, ZF5, ZFP410, ZFX, ZNF282, ZNF300
**(introns)**	(AARE, AHRARNT, AIRE, AML1, AML2, AP1, AP2, AP4, ARID5A, ARNTL, ATBF1, ATF, ATOH1, BARHL1, BCL6, BKLF, BLIMP1, BNC, BRIGHT, BRN2, BRN3, BRN4, BRN5, BSX, BTEB3, CAAT, CAR_RXR, CARF, CART1, CDP, CDX1, CDX2, CEBP, CEBPB, CEBPE_ATF4, CHOP, CHR, CLEAR, CMYB, COUP, CP2, CREB, CREB3L2, CREL, CRX, CSRNP1, CTCF, DBP, DEC1, DEC2, δEF1, DLX2, DLX3, DLX4, DMP1, DMRT3, DREAM, DUX4, E2F, E2F3, E2F4, E2F7, E4F, EBF1, EGR1, EGR2, EGR3, EKLF, ELK1, ER, ESRRA, ESRRB, ETV1, EVI1, EVX1, FAST1, FHXB, FOSL1, FOXO1, FOXP1_ES, FREAC7, FTF, FXRE, GABP, GATA1, GATA3, GBX1, GC, GCM1, GFI1, GKLF, GLIS2, GLIS3, GRHL1, GRHL2, GRHL3, GSH1, GSH2, HAND2_E12, HAS, HBP1, HDBP1_2, HEN1, HESX1, HIC1, HMBOX, HMGIY, HMX2, HMX3, HNF1, HNF3, HNF3B, HNF6, HOMEZ, HOX1-3, HOXA3, HOXB3, HOXB4, HOXB5, HOXC13, HOXC8, HOXC9, HOXD10, HSF1, HSF2, IK1, IK2, IK3, INSM1, IPF1, IR1_NGRE, IRF2, IRF4, ISL1, ISL2, JARID2, JUNB, KKLF, KLF12, KLF2, KLF6, KLF7, LACTOFERRIN, LBX2, LEF1, LHX2, LHX4, LHX9, LRRFIP1, LTSM, LYF1, LYL1_E12, MARE, MAZ, MAZR, MEIS1, MEL1, MESP1_2, MIT, MIXL1, MIZ1, MIZF, MOK2, MSX2, MSX3, MTBF, MYBL1, MYOD, MYOGENIN, MYRF, MYT1, MZF1, NACA1, NANOG, NBRE, NEUROG, NF1, NFAT5, NFATC1, NF-κB, NF-κB50, NFY, NGN_NEUROD, NKX11, NKX12, NKX25, NKX26, NKX29, NKX31, NKX61, NKX63, NM23, NMP4, NOBOX, NR2F6, NRF1, NRL, NRSF, NXF_ARNT, OC2, OCT1, OCT3_4, OLF1, OLIG2, OTX2, OVOL1, P53, PARAXIS, PAX3, PAX4, PAX4_PD, PAX5, PAX6, PAX6_HD, PBX1_MEIS1, PBX3, PCE1, PDEF, PDX1, PEA3, PHOX2, PIT1, PLAG1, PLAGL1, PLZF, PNR, POU2F3, POU6F2, PPARG, PRDM1, PRDM14, PROP1, PTF1, PURα, RAR_RXR, RAX, RBPJK, REV-ERBA, RFX4, RORA, RP58, RREB1, S8, SALL1, SALL2, SATB1, SF1, SIX2, SIX3, SMAD3, SMAD4, SMARCA, SOX1, SOX10, SOX15, SOX5, SOX7, SOX9, SP1, SP2, SP4, SPI1, SPZ1, SREBP, SRF, SRY, STAT3, STAT5A, STAT5B, SZF1, TAL1_E2A, TAL1αE47, TCF2, TCFAP2A, TCFAP2B, TCFCP2L1, TCFE2A, TEF, TH1E47, THRB, TLX1, TST1, VAX2, VBP, VDR_RXR, VERBA, VMYB, WHN, WT1, XBOX, XFD2, YB1, YY1, YY2, ZBED1, ZBED4, ZBP89, ZBTB3, ZBTB7, ZF5, ZFP410, ZFP57, ZFP652, ZFX, ZIC2, ZIC3, ZKSCAN3, ZNF217, ZNF219, ZNF263, ZNF282, ZNF300, ZNF35, ZNF354C, ZSCAN10)

	specific TF for Gal-1	specific TF for Gal-3
**promoter**	AARE, AML1, AP2, AP4, AREB6, ARNTL, BTEB3, CART1, CHREBP_MLX, CJUN_ATF2, CKROX, CREB1, CTCFL, DLX5, DMRT1, E47, EGR2, EGR3, ELK1, ER, ETV4, FOXP1, GABPA, GC_SBE, GLIS2, GLI3, GRE, GSC, GZF1, HAS, HDGF, HFH3, HIF1, HNF4, HOMEZ, HOXD13, ISGF3G, KLF2, KLF12, LACTOFERRIN, LBX2, LHX4, LHX8, MAZ, MEF3, MEIS1, MIT, MIZF, MOK2, MSX2, MTF-1, MYF5, MYOGENIN, MYT1L, NACA1, NEUROD1, NF-κB, NKX29, NM22, NRF1, NRL, NXF_ARNT, OLIG2, PAX2, PAX4, PBX1, PEG3, PLU1_JARID1B, PNR, PRDM5, PXR_RXR, RARA, RAX, RREB1, RU49, RXR_RXR, SALL1, SIX2, SOX10, SOX5, SP4, SPT, SZF1, TAL1_E2A, TCFAP2E, TGIF, THR, VERBA, WHN, WT1XBP1, YY1, ZIC2, ZIC3, ZKSCAN3, ZNF219, ZNF263, ZNF354C	AIRE, AML3, BACH2, BARHL2, BARX1, BARX2, BATF, BHLHA15, BRN4, CAAT, CDPCR3HD, CDX2, CEBPB, CEBPE_ATF4, CPHX, DBP, DICE, DLX2, DMP1, DMRT3, DUX4, E2F7, E4BP4, EMX2, ERG, ESR2, ESRRA, ETS2, ETV1, EVI1, FAC1, FHXB, FOSL2, FREAC2, FTF, GAGA, GATA2, GATA4, GBX1, GFI1B, GLIS3, GRHL2, GRHL3, HBP1, HLF, HMGA, HMX2, HNF3B, HNF4G, HNF6, HOXA10, HOXA3, HOXA9, HOXB13, HOXB3, HOXB4, HOXB7, HOXC10, HOXC8, HOXC9, HOXD8, IK1, IK2, IPF1, IRF3, ISRE, ISX, JARID2, LHX5, LHX9, MASH1, MEIS1, MEOX1, MIXL1, MSX1, MSX3, MYCMAX, MYT1, NKX63, NOBOX, NUDR, OC2, OCT1, OCT3_4, OSR1, OVOL1, PARAXIS, PAX6_HD, PAX7, PAX8, PEA3, PHOX2A, PLZF, POU3F3, POU6F2, PTX1, RARG, RBPJK, REX1, RFX1, RFX4, RFX5, RORA, RP58, SATB1, SCX, SF1, SIX1, SL1, SOX1, SOX17, SOX21, SOX4, SOX6, SOX8, SOX9, SPIB, SRY, STAT1, TAL1βHEB, TCF11, TCF12, TCFCP2L1, TEAD4, TEF, TEF_HLF, THRA, TIEG, TST1, VBP, VSX1, ZFP652, ZNF217, ZTRE
**(introns)**	(ACAAT, ARE, BACH2, BRACH, CHREBP_MLX, CJUN_ATF2, CLOX, ELF2, ESR2, ESRRG, ETS2, FLI, GABPA, GATA2, GLI3, GLIS1, GMEB2, GTF3R4, HDG, HES1, HFH3, HLXB9, HOXA5, HOXB7, HOXD1, HPF1, LMX1B, MASH1, MEF3, MEIS1A_HOXA9 ,MIF1, MRG1, NEUROD1, NF-κB65, NUDR, PRRX1, PRRX2, PSE, RARA, RFX1, RFX3, RXR_RXR ,SCRT2 ,SOX2 ,TAXCREB, TCF12, TCF21, TCFAP2C, ZEC, ZNF143, ZNF202, ZNF76_143)	(ALX3, ALX4, AML3, AREB6, ATF6, BAPX1, BARBIE, BARX1, BARX2, BATF, BATF3, BHLHA15, CABL, CDE, CEBPA, CEBPD, CEBPE, CEBPG, CKROX, CMYC, CPHX, CREB2, DLX1, DLX5, DMBX1, DMRT1, DMRT2, DMRT4, DMRT5, DMRT7, E2F2, E2F6, E47, E4BP4, ELF5, EMX2, EN1, EN2, EOMES, ERG, ERR, ESX1, ETS1, ETV4, EVX2, FAC1, FOXA1, FOXJ1, FOXP1, FOXP2, FREAC2, FREAC3, GATA4, GBX2, GC_SBE, GFI1B, GLI1, GRE, HELT, HES7, HFH1, HHEX, HIF1, HIVEP1, HLF, HMGA, HMX1, HNF4, HNF4A, HNF4G, HOX_PBX, HOXA1, HOXA10, HOXA13, HOXA2, HOXA4, HOXA9, HOXB4, HOXB6, HOXB8, HOXB9, HOXC4, HOXC6, HOXC10, HOXD3, HOXD8, HOXD13, HRE, IRF1, IRF3, IRF7, IRX2, IRX3, IRX5, IRX6, ISGF3G, ISRE, ISX, JUNDM2, KAISO, LHX1, LHX3, LHX6, LMX1A, MAFA, MAFB, MAFF, MAFK, MEF2, MEF2C, MEF3, MEIS1, MEIS1B_HOXA9, MEOX1, MGA, MNT, MRF2, MSX, MTF-1, MYBL2, MYCMAX, MYF5, MYF6, NFAT, NFE2, NKX32, NMYC, NRF2, OSNT, OSR1, OTX1, PAX2, PAX7, PAX8, PBX1, PEG3, PEGASUS, PKNOX2, PLU1_JARID1B, PRE, PREB, PROX1, PTX1, PXR_RXR, RARG, REX1, RFX5, RHOX6, RSRFC4, RTR, RU49, SCX, SIP1, SIX1, SIX3, SL1, SOX18, SOX21, SOX3, SOX30, SOX4, SOX6, SPIB, SPIC, SPT, STAF, STAT1, STAT5, STAT6, TAL1βE47, TBOX, TBX20, TCF11, TCF11MAFG, TCF7, TCF7L1, TCFAP2E, TEAD, TEF_HLF, TGIF, THAP1, THR, THRA, TIEG, TLX2, TP63, TWIST, USF, VAX1, VMAF, XBP1, XFD1, XVENT2, ZBTB7, ZID, ZTRE)

Names of transcription factors: AARE: amino acid response element, ATF4 binding site; ACAAT: avian C-type LTR CCAAT box; AHRARNT: aryl hydrocarbon/Arnt heterodimers, fixed core; AIRE: autoimmune regulator; AML1: acute myeloid leukemia 1 protein, RUNX1 (runt-related transcription factor 1); AML3: acute myeloid leukemia 3 protein, RUNX2 (runt-related transcription factor 2)/CBFA1 (core-binding factor, runt domain, alpha subunit 1); AP1: Activator protein 1; AP2: Activator protein 2 alpha; AP4: Activator protein 4; ARE: androgene receptor binding site, IR3 sites; AREB6: AREB6 (Atp1a1 regulatory element binding factor 6); ARNTL: aryl hydrocarbon receptor nuclear translocator-like, homodimer; ATBF1: AT-binding transcription factor 1; ATF6: activating transcription factor 6, member of b-zip family, induced by ER damage/stress, binds to the ERSE in association with NF-Y; BACH2: BTB and CNC homology 1, basic leucine zipper transcription factor 2; BARBIE: barbiturate-inducible element; BARHL2: BarH-like homeobox 2; BARX1: BARX homeobox 1; BARX2: Barx2, homeobox transcription factor that preferentially binds to paired TAAT motifs; BATF: basic leucine zipper transcription factor, ATF-like; BCL6: B-cell lymphoma 6 protein, POZ/zinc finger protein, transcriptional repressor, translocations observed in diffuse large cell lymphoma; BHLHA15: basic helix-loop-helix family member A15, also known as MIST1 (muscle, intestine and stomach expression 1); BKLF: basic Krueppel-like factor (KLF3); BRIGHT: Bright, B cell regulator of IgH transcription; BRN2: POU-III protein class; BRN3: POU class 4 homeobox 3 (POU4F3), BRN3C; BRN4: POU domain transcription factor brain 4; BRN5: POU class 6 homeobox 1 (POU6F1); BSX: brain specific homeobox; BTEB3: basic transcription element (BTE) binding protein, BTEB3, FKLF-2; CAAT: cellular and viral CCAAT box; CART1: cartilage homeoprotein 1; CDP: transcriptional repressor CDP; CDPCR3HD: cut-like homeodomain protein (Cut Repeat III / homeodomain); CDX1: caudal type homeobox 1; CDX2: caudal type homeobox 2; CEBPB: CCAAT/enhancer binding protein beta; CEBPE_ATF4: heterodimer of CEBP epsilon and ATF4; CHOP: C/EBP homologous protein, heterodimers of CHOP and C/EBPalpha; CHR: cell cycle gene homology region (CDE/CHR tandem elements regulate cell cycle dependent repression); CHREBP_MLX: carbohydrate response element binding protein (CHREBP) and Max-like protein X (Mlx) bind as heterodimers to glucose-responsive promoters; CJUN_ATF2: c-Jun/ATF2 heterodimers; CKROX: collagen krox protein (zinc finger protein 67 - ZFP67); CP2: transcription factor CP2; CPHX: cytoplasmic polyadenylated homeobox; CREB1: cAMP-responsive element binding protein 1; CREB2: cAMP-responsive element binding protein 2; CREL: proto-oncogene c-Rel, subunit of NF-κB; CRX: cone-rod homeobox-containing transcription factor; CSRNP1: cysteine-serine-rich nuclear protein 1 (AXUD1, AXIN1 up-regulated 1); CTCF: insulator protein, transcriptional repressor CTCF (CCCTC-binding factor); CTCFL: CCCTC-binding factor (zinc finger protein)-like (BORIS); DBP: albumin D-box binding protein; δEF1: δEF1, also known as zinc finger E-box binding homeobox 1 (ZEB1); DICE: downstream immunoglobulin control element, interacting factor: BEN (also termed Mus-TRD1 and WBSCR11); DLX1: distal-less homeobox 1; DLX2: distal-less homeobox 2; DLX3: distal-less homeobox 3; DLX5: distal-less homeobox 5; DMP1: dentin matrix acidic phosphoprotein 1, cyclin D binding myb-like transcription factor; DMRT1: doublesex and mab-3 related transcription factor 1; DMRT3: doublesex and mab-3 related transcription factor 3; DREAM: downstream regulatory element-antagonist modulator, Ca^{2+}-binding protein of the neuronal calcium sensors family that binds DRE (downstream regulatory element) sites as a tetramer; DUX4: double homeobox protein 4; E2F: E2F, involved in cell cycle regulation, interacts with Rbp107 protein; E2F1: E2F transcription factor 1; E2F3: E2F transcription factor 3; E2F4: E2F transcription factor 4; E2F7: E2F transcription factor 7; E47: E47 homodimer; E4BP4: E4BP4, bZIP domain, transcriptional repressor; EBF1: early B-cell factor 1; EGR1: early growth response 1; EGR2: early growth response 2; EGR3: early growth response 3; EKLF: erythroid Krueppel like factor; ELK1: ETS domain-containing protein Elk-1; EMX2: empty spiracles homeobox 2; EN1: engrailed homeobox 1; ER: estrogen response elements (ER alpha), IR3 sites; ERG: ETS-related gene; ESR2: estrogen receptor 2 (ER beta); ESRRA: estrogen-related receptor alpha; ETS2: ETS proto-oncogene 2; ETV1: Ets variant 1; ETV4: Ets variant 4; EVI1: ecotropic viral integration site 1 encoded factor, amino-terminal zinc finger domain; FAC1: fetal Alz-50 clone 1; FAST1: forkhead box H1; FHXB: fork head homologous X binds DNA with a dual sequence specificity (FHXA and FHXB); FOSL2: FOS-like antigen 2, AP-1 transcription factor subunit; FOXP1: sites bound by FOXP1 and an alternative splicing variant FOXP1_ES, activated in ESCs; FREAC2: fork head related activator-2; FREAC7: fork head related activator-7; FTF: Alpha (1)-fetoprotein transcription factor (FTF), liver receptor homologue-1 (LRH-1), Nr5a2; GABPA: GA binding protein transcription factor, alpha; GAGA: GAGA-Box; GATA1: GATA-binding factor 1; GATA2: GATA-binding factor 2; GATA3: GATA-binding factor 3; GATA4: GATA binding protein 4; GBX1: gastrulation brain homeobox 1; GC: GC box elements; GC_SBE: GC-rich Smad1/5 binding element; GCM1: glial cells missing homolog 1 (secondary DNA binding preference); GFI1: growth factor independence 1; GFI1B: growth factor independence 1 zinc finger protein Gfi-1B; GKLF: gut-enriched Krueppel-like factor; GLI3: GLI-Kruppel family member; GLIS2: GLIS family zinc finger 2; GLIS3: GLIS family zinc finger 3, Gli-similar 3; GRE: glucocorticoid receptor, IR3 sites; GRHL2: grainyhead-like 2 (BOM, TFCP2L3); GRHL3: grainyhead-like 3 (sister-of-mammalian grainyhead - SOM); GSC: goosecoid homeobox; GSH1: homeobox transcription factor Gsh-1; GSH2: homeodomain transcription factor Gsh-2; GZF1: GDNF-inducible zinc finger protein 1 (ZNF336); HAS: HIF-1 ancillary sequence; HBP1: HMG box-containing protein 1; HDBP1_2: Huntington's disease gene regulatory region-binding protein 1 and 2 (SLC2A4 regulator and papillomavirus binding factor); HDGF: hepatoma-derived growth factor; HFH1: HNF-3/Fkh Homolog 1 (FOXQ1); HFH3: HNF-3/Fkh Homolog 3 (FOXI1, Freac-6); HHEX: hematopoietically expressed homeobox, proline-rich homeodomain protein; HIC1: hypermethylated in cancer 1; HIF1: hypoxia inducible factor, bHLH/PAS protein family; HLF: hepatic leukemia factor; HMGA: HMGA family of architectural transcription factors (HMGA1, HMGA2); HMGIY: HMGI(Y) high-mobility-group protein I (Y), architectural transcription factor organizing the framework of a nuclear protein-DNA transcriptional complex; HMX2: H6 family

homeobox 2; HMX3: H6 family homeobox 3; HNF1: hepatocyte nuclear factor 1 alpha (Tcf-1); HNF3B: hepatocyte nuclear factor 3beta (FOXA2); HNF4: hepatic nuclear factor 4, DR1 sites; HNF4G: hepatocyte nuclear factor 4 gamma (NR2A2), DR1 sites; HNF6: onecut homeodomain factor HNF6; HOMEZ: homeobox and leucine zipper encoding transcription factor; HOXA3: homeobox A3; HOXA5: homeobox A5/Hox-1.3; HOXA9: homeobox A9; HOXA10: homeobox A10/Hox1.8; HOXB3: homeobox B3/Hox 2-gamma; HOXB4: homeobox B4; HOXB7: homeobox B7/Hox2c; HOXB8: homeobox B8/Hox-2delta; HOXB9: Abd-B-like homeodomain protein Hoxb-9; HOXB13: homeobox B13; HOXC4: homeobox C4/Hox-3epsilon; HOXC8: homeobox C8/Hox-3alpha; HOXC9: homeobox C9; HOXC10: homeobox C10/Hox-3iota; HOXC13: homeobox C13; HOXD8: homeobox D8; HOXD10: homeobox D10; HOXD13: homeobox D13/Hox-4I; HSF1: heat shock factor 1; HSF2: heat shock factor 2; IK1: Ikaros 1; IK2: Ikaros 2; IK3: Ikaros 3; INSM1: zinc finger protein insulinoma-associated 1 (IA-1) functions as a transcriptional repressor; IPF1: insulin promoter factor 1, pancreatic and duodenal homeobox 1 (Pdx1); IR2_NGRE: repressive binding sites for glucocorticoid receptor (IR2); IRF2: interferon regulatory factor 2; IRF3: Interferon regulatory factor 3; ISGF3G: interferon-stimulated transcription factor 3, gamma (IRF9); ISL2: ISL LIM homeobox 2; ISRE: interferon-stimulated response element; ISX: intestine-specific homeobox; JARID2: Jumonji, AT rich interactive domain 2 (JMJ); JUNB: transcription factor Jun-B; KAISO: transcription factor Kaiso, ZBTB33; KKLF: kidney-enriched Krueppel-like factor, KLF15; KLF2: Krueppel-like factor 2 (lung) (LKLF); KLF6: core promoter-binding protein (CPBP) with 3 Krueppel-type zinc fingers (KLF6, ZF9); KLF7: Krueppel-like factor 7; KLF12: Krueppel-like factor 12 (AP-2rep); LACTOFERRIN: lactotransferrin and delta-lactoferrin, growth-inhibiting protein 12; LBX2: ladybird homeobox 2; LEF1: TCF/LEF-1, involved in the Wnt signal transduction pathway; LHX1: LIM homeobox 1; LHX2: LIM homeobox 2; LHX3: LIM homeobox 3; LHX4: LIM homeobox 4; LHX5: LIM homeobox 5; LHX8: LIM homeobox 8; LHX9: LIM homeobox 9; LRRFIP1: leucine rich repeat (in FLII) interacting protein 1; LTSM: LTSM elements with 8 bp spacer; MAFA: lens-specific Maf/MafA-sites; MASH1: mammalian achaete scute homolog 1 (Ascl1); MAZ: myc associated zinc finger protein; MAZR: myc-associated zinc finger protein related transcription factor; MEF2: myocyte-specific enhancer factor 2; MEF3: myocyte-specific enhancer factor 3; MEIS1: monomeric Meis1 homeodomain protein; MEL1: MEL1 (MDS1/EVI1-like gene 1) DNA-binding domain 2; MEOX1: mesenchyme homeobox 1; MESP1_2: mesoderm posterior 1 and 2; MIT: microphthalmia transcription factor, MIXL1: Mix1 homeobox-like 1; MIZ1: myc-interacting Zn finger protein 1, zinc finger and BTB domain containing 17 (ZBTB17); MIZF: MBD2 (methyl-CpG-binding protein)-interacting zinc finger protein, histone nuclear factor P (HiNF-P); MOK2: ribonucleoprotein associated zinc finger protein MOK-2 (human); MSX: homeodomain proteins MSX-1 and MSX-2; MSX1: muscle-segment homeobox 1; MSX2: muscle segment homeobox 2, homologue of *Drosophila* (HOX 8); MSX3: muscle segment homeobox 3; MTBF: muscle-specific Mt binding site; MTF-1: metal transcription factor 1, MRE; MYBL1: v-myb avian myeloblastosis viral oncogene homolog-like 1 (AMYB) (secondary DNA binding preference); MYCMAX: MYC-MAX binding sites; MYF5: myogenic factor 5; MYOD: myogenic differentiation; MYOGENIN: myogenic bHLH protein; MYT1: myelin transcription factor 1; MYT1L: myelin transcription factor 1 like; MZF1: myeloid zinc finger 1; NACA1: nascent polypeptide-associated complex subunit alpha 1; NANOG: homeobox transcription factor Nanog; NBRE: monomers of the nur subfamily of nuclear receptors (nur77, nurr1, nor-1); NEUROD1: neurogenic differentiation 1; NF1: nuclear factor 1; NFAT: nuclear factor of activated T-cells; NFAT5: nuclear factor of activated T-cells 5; NF-κB: nuclear factor κB; NF-κB65: nuclear factor κB (p65); NFY: nuclear factor Y (Y-box binding factor); NKX11: NK1 homeobox 1, SAX2; NKX12: NK1 homeobox 2, Sax1-like; NKX25: homeodomain factor Nkx-2.5/Csx, tinman homolog low affinity sites; NKX29: NK2 homeobox 9, NKX2H; NKX31: NK3 homeobox 1, NKX-3 ALPHA, BAPX2; NKX61: NK6 homeobox 1; NM23: NME/NM23 nucleoside diphosphate kinase1 and 2; NMP4: nuclear matrix protein 4; NOBOX: homeobox containing germ cell-specific transcription factor NOBOX; NRF1: nuclear respiratory factor 1; NRL: neural retinal basic leucine zipper factor (bZIP); NUDR: nuclear DEAF-1 related transcriptional regulator protein; NXF_ARNT: bHLH-PAS type transcription factors NXF/ARNT heterodimer; OC2: CUT-homeodomain transcription factor Onecut-2; OCT1: octamer-binding factor 1; OCT3_4: POU domain, class 5, transcription factor 1: OLF1: olfactory neuron-specific factor; OLIG2: oligodendrocyte lineage transcription factor 2; OSR1: odd-skipped related 1; OVOL1: zinc finger transcription factor OVO homolog-like 1; P53: tumor suppressor p53; PARAXIS: Paraxis (TCF15), member of the Twist subfamily of Class B bHLH factors, forms heterodimers with E12; PAX2: paired box protein 2; PAX3: paired box protein 3; PAX4: paired box protein 4; PAX6: paired box protein 4; PAX6_HD: paired box 6, homeodomain binding site; PAX7: paired box protein 7; PAX8: PAX 2/5/8 binding site; PBX1: pre B cell leukemia homeobox 1; PCE1: photoreceptor conserved element 1; PEA3: polyomavirus enhancer A binding protein 3, ETV4 (Ets variant gene 4); PEG3: paternally expressed 3; PHOX2: paired like homeobox 2a and 2b; PHOX2A: paired like homeobox 2a; PIT1: GHF-1 pituitary specific pou domain transcription factor; PLAG1: pleomorphic adenoma gene 1; PLAGL1: pleiomorphic adenoma gene-like 1; PLU1_JARID1B: Jumonji, AT rich interactive domain 1B; PLZF: promyelocytic leukemia zinc finger (TF with nine Krueppel-like zink fingers); PNR: photoreceptor-specific nuclear receptor subfamily 2, group E, member 3 (Nr2e3), DR1 sites; POU3F3: POU class 3 homeobox 3 (POU3F3), OTF8; POU6F2: retina-derived POU-domain factor-1, dimeric binding site; PPARG: peroxisome proliferator-activated receptor gamma, DR1 sites; PRDM1: PRDI (positive regulatory domain I element) binding factor 1; PRDM5: PR domain containing 5; PRDM14: PR domain zinc finger protein 14; PREB: prolactin regulatory element-binding protein; PROP1: prophet of Pit 1, PROP paired-like homeobox 1; PSE: proximal sequence element (PSE) of RNA polymerase II-transcribed snRNA genes; PTX1: pituitary Homeobox 1 (Ptx1, Pitx-1); PURALPHA: purine-rich element binding protein A; PXR_RXR: pregnane X receptor/retinoid X receptor heterodimer, DR4 sites; RARA: retinoic acid receptor alpha, homodimer DR4 binding site; RARG: retinoic acid

receptor gamma, homodimer DR2 binding site; RAX: retina and anterior neural fold homeobox; RBPJK: mammalian transcriptional repressor RBP-Jkappa/CBF1; REX1: REX1 transcription factor; zinc finger protein 42; RFX1: regulatory factor X 1; RFX4: regulatory factor X 4; RFX5: regulatory factor X, 5 (influences HLA class II expression); RORA: RAR-related orphan receptor alpha; RP58: zinc finger protein RP58 (ZNF238); RREB1: ras-responsive element binding protein 1; RU49: zinc finger transcription factor RU49 (zinc finger proliferation 1 - Zipro 1); RXR_RXR: retinoid X receptor homodimer, DR1 sites; S8: S8 type homeodomains; SALL1: spalt-like transcription factor 1; SATB1: special AT-rich sequence-binding protein 1; SCX: tendon-specific bHLH transcription factor scleraxis; SF1: steroidogenic factor 1; SIX1: sine oculis homeobox homolog 1; SIX2: sine oculis homeobox homolog 2; SL1: member of the RSRF (related to serum response factor) protein family from *Xenopus laevis*; SMAD3: transcription factor involved in TGF-beta signaling; SMARCA3: SWI/SNF related, matrix associated, actin dependent regulator of chromatin, subfamily a, member 3; SOX1: SRY (sex determining region Y)-box 1; SOX3: SRY-box containing gene 3; SOX4: SRY-box containing gene 4; SOX5: SRY-box containing gene 5; SOX6: SRY-box containing gene 6; SOX8: SRY-box containing gene 8; SOX9: SRY-box containing gene 9; SOX10: SRY-box containing gene 10; SOX17: SRY-box containing gene 17; SOX21: SRY-box containing gene 21; SP1: stimulating protein 1; SP4: Sp4 transcription factor; SPI1: SPI-1 proto-oncogene; hematopoietic transcription factor PU.1; SPIB: Spi-B transcription factor (Spi-1/PU.1 related); SPT: T-box gene 16 (tbx16) of *zebrafish*; SREBP: sterol regulatory element binding protein; SRF: serum response factor; SRY: sex determining region Y; STAT: signal transducers and activators of transcription; STAT1: signal transducer and activator of transcription 1; STAT3: signal transducer and activator of transcription 3; STAT5A: signal transducer and activator of transcription 5A; STAT6: signal transducer and activator of transcription 6; SZF1: hematopoietic progenitor-restricted KRAB-zinc finger protein; TAL1_E2A: T-cell acute lymphocytic leukemia 1, SCL; TAL1BETAHEB: Tal-1beta/HEB heterodimer; TCF7L1: HMG box transcription factor Tcf7l1 (TCF3); TCF11: TCF11/LCR-F1/Nrf1 homodimers; TCF12: helix-loop-helix transcription factor 12; TCFAP2A: transcription factor AP-2, alpha; TCFAP2E: transcription factor AP-2, epsilon; TCFCP2L1: transcription factor CP2-like 1 (LBP-9); TEAD4: TEA domain family member 4, TEF-3; TEF: thyrotrophic embryonic factor; TEF_HLF: thyrotrophic embryonic factor/hepatic leukemia factor; TGIF: TG-interacting factor belonging to TALE class of homeodomain factors; THR: THR/RXR heterodimer and THR homodimer DR4 binding sites; THRA: thyroid hormone receptor, alpha (ER4 - everted repeat, spacer 4); TIEG: TGFbeta-inducible early gene (TIEG)/early growth response gene alpha (EGRalpha); TLX1: T-cell leukemia homeobox 1; TST1: POU-factor Tst-1/Oct-6; VBP: PAR-type chicken vitellogenin promoter-binding protein; VDR_RXR: bipartite binding site of VDR/RXR heterodimers, DR4 sites; VERBA: vErbA, viral homolog of thyroid hormone receptor alpha1; VMYB: v-Myb, variant of AMV v-myb; VSX1: visual system homeobox 1; WHN: winged helix protein; WT1: wilms tumor suppressor; XBOX: motif bound by regulatory factor X (RFX) proteins; XBP1: X-box-binding protein 1; YB1: Y box binding protein 1; YY1: Yin and Yang 1 activator sites; ZBED4: zinc finger, BED-type containing 4; ZBP89: zinc finger transcription factor; ZBTB3: zinc finger and BTB domain containing 3; ZBTB7: zinc finger and BTB domain containing 7A, pokemon; ZF5: zinc finger/POZ domain transcription factor; ZFP410: zinc finger protein 410, APA-1; ZFP652: Zinc finger protein 652; ZFX: X-linked zinc finger protein; ZIC2: zic family member 2 (odd-paired Drosophila homolog); ZIC3: zic family member 3 (odd-paired Drosophila homolog); ZKSCAN3: zinc finger with KRAB and SCAN domains 3; ZNF217: zinc finger protein 217; ZNF219: Krueppel-like zinc finger protein 219; ZNF263: zinc finger protein 263, ZKSCAN12 (zinc finger protein with KRAB and SCAN domains 12); ZNF282: zinc finger protein 282 (HTLV-I U5 repressive element-binding protein 1); ZNF300: KRAB-containing zinc finger protein 300; ZNF354C: KRAB-zinc finger protein synten (KID3); ZTRE: ZTRE motifs

Supplementary Table S3.

Computationally detected putative TF-binding sites in the promoter and intron regions of the gene for human Gal-3.

Practically, the core similarity was set to 1.0 and only matrices with a value > 0.8 were used. The positions (*Pos.*) of potential TF-binding sites are given relative to the transcription start point (TSP) +1a[41]. Orientation (*Ori.*) gives information whether the motif is located on the sense (+) or antisense (-) strand. The core sequence is indicated by capital letters. The core similarity (*Core Sim.*) gives information about the score for this core sequence, which consists of the four highest conserved positions of the matrix. The matrix similarity (*Matrix Sim.)* shows the score for the DNA sequence with the respective Matrix family. Each position in the DNA sequence is compared with the highest conserved nucleotide at the same position in the matrix. The used matrices of the MatInspector software for the calculation of the matrix similarity are shown (*Matrix*). Functionally related TFs are clustered and named for their *families*.

Promoter region
-2500 bp upstream of the transcription start point

Family	Matrix	Pos.	Ori.	Sequence	Core Sim.	Matrix Sim.
O$PTBP	O$PTATA.01	-612	(+)	gggaTATAaacattc	1.0	0.910
	O$PTATA.02	-507	(-)	acatTATAtatatat	1.0	0.936
	O$PTATA.02	-508	(+)	tataTATAtataatg	1.0	0.928
	O$PTATA.02	-509	(-)	attaTATAtatatat	1.0	0.936
	O$PTATA.02	-510	(+)	tataTATAtatataa	1.0	0.936
	O$PTATA.02	-511	(-)	tataTATAtatatac	1.0	0.936
	O$PTATA.02	-512	(+)	ggtaTATAtatatat	1.0	0.936
	O$PTATA.02	-513	(-)	tataTATAtataccc	1.0	0.947
	O$PTATA.02	-514	(+)	agggTATAtatatat	1.0	0.926
	O$PTATA.02	-1552	(-)	atgcTATAaatctta	1.0	0.929
	O$PTATA.02	-1884	(+)	aaatTATAaattccc	1.0	0.922
O$TELO	O$ZSCAN4.01	-1868	(+)	gGCACaccctgatca	1.0	0.850
O$TF2B	O$BRE.01	-123	(-)	ccgCGCC	1.0	1.000
O$VTBP	O$ATATA.01	-1138	(+)	gtttacaTAAGccagtc	1.0	0.813
	O$ATATA.01	-2274	(-)	aagtcttTAAGgataag	1.0	0.822
	O$LTATA.01	-503	(+)	ataTATAatgtgaattt	1.0	0.841
	O$VTATA.01	-455	(-)	ccttaTAAAgcaattaa	1.0	0.906
	O$VTATA.01	-611	(+)	ggataTAAAcattcaga	1.0	0.910
	O$VTATA.01	-1555	(-)	tgctaTAAAtcttaaga	1.0	0.932
	O$VTATA.01	-1883	(+)	aattaTAAAttcccagg	1.0	0.940
	O$VTATA.02	-740	(-)	cctaaTAAAagaggccg	1.0	0.896
	O$VTATA.02	-1977	(-)	cccaaTAAAatcaatta	1.0	0.928
	O$VTATA.02	-2114	(-)	gctgaTAAAagcaagga	1.0	0.891

O$YTBP	O$SPT15.01	-508	(-)	cacattaTATAtatata	1.0	0.922
	O$SPT15.01	-509	(+)	atatataTATAtaatgt	1.0	0.933
	O$SPT15.01	-510	(-)	cattataTATAtatata	1.0	0.921
	O$SPT15.01	-511	(+)	gtatataTATAtataat	1.0	0.940
	O$SPT15.01	-512	(-)	ttatataTATAtatacc	1.0	0.954
	O$SPT15.01	-513	(+)	gggtataTATAtatata	1.0	0.914
	O$SPT15.01	-514	(-)	atatataTATAtaccct	1.0	0.914
	O$SPT15.01	-515	(+)	gagggtaTATAtatata	1.0	0.951
	O$SPT15.01	-896	(+)	taggcaaTATAcattta	1.0	0.836
V$ABDB	V$HOXA10.01	-1918	(+)	ttgggtaaTAAAgagcc	1.0	0.909
	V$HOXB9.01	-1177	(-)	ctggttccTAAAtaatt	1.0	0.914
	V$HOXB9.01	-1502	(-)	ctgggtagTAAAaaggt	1.0	0.918
	V$HOXB13.01	-1974	(-)	gaacccaaTAAAatcaa	1.0	0.994
	V$HOXC9.01	-460	(-)	taaagcaaTTAAtcaga	1.0	0.864
	V$HOXC9.01	-1212	(+)	ttatgttaTTAAttggt	1.0	0.904
	V$HOXC10.01	-920	(+)	aaatatcaTAAActggg	1.0	0.864
	V$HOXC13.01	-736	(-)	gtgccctaaTAAAagag	1.0	0.957
	V$HOXC13.01	-1460	(+)	ccattcaaaTAAAttgg	1.0	0.934
	V$HOXC13.01	-1904	(-)	cacatgcaaTAAAcggc	1.0	0.922
	V$HOXC13.02	-2111	(-)	gccgctgaTAAAagcaa	1.0	0.849
	V$HOXD10.01	-2349	(+)	caaaaaaaTAAAataaa	1.0	0.924
V$AHRR	V$AHRARNT.03	-2211	(-)	gagggaagctGCGTgagaacttcag	1.0	0.974
V$AIRE	V$AIRE.01	-1209	(+)	tgTTATtaattggtt	1.0	0.978
V$AP1F	V$AP1.02	-982	(+)	acatGAGTgagct	1.0	0.896
	V$AP1.02	-1996	(-)	cattGAGTaactt	1.0	0.901
	V$BATF.01	-1568	(+)	tcatgaCTCAaag	1.0	0.987
	V$FOSL2.01	-1272	(+)	aagtgAGTCcttc	1.0	0.852
	V$JUNB.01	-1485	(+)	aggttaGTCAttt	1.0	0.925
	V$JUNB.01	-1568	(-)	ctttgaGTCAtga	1.0	0.988
V$AP1R	V$BACH2.02	-1573	(-)	taagacttTGAGtcatgaggcct	1.0	0.953
	V$MAFA.01	-1659	(+)	tcccttgcctccacAGCAattca	1.0	0.928
V$AP2F	V$TCFAP2A.02	-156	(+)	cgcccCCTGcggcggc	1.0	0.963
V$ARID	V$BRIGHT.01	-331	(-)	attcaATTAataggttgcttt	1.0	0.946
	V$BRIGHT.01	-466	(-)	aagcaATTAatcagacaactc	1.0	0.942
	V$BRIGHT.01	-1215	(-)	aaccaATTAataacataaaga	1.0	0.961
	V$BRIGHT.01	-1989	(-)	aatcaATTAagatatcattga	1.0	0.949
	V$JARID2.01	-952	(+)	ttctctTTTAgtctgtttggg	1.0	0.899
	V$JARID2.01	-1462	(-)	aaccaaTTTAtttgaatggat	1.0	0.900
	V$JARID2.01	-1975	(+)	attgatTTTAttgggttcctt	1.0	0.934
	V$JARID2.01	-2352	(-)	atttatTTTAttttttgaga	1.0	0.896
V$ATBF	V$ATBF1.01	-1205	(-)	tatagtaaccAATTaat	1.0	0.834
	V$ATBF1.01	-1212	(+)	ttatgttattAATTggt	1.0	0.872
V$BARB	V$BARBIE.01	-741	(-)	aataAAAGaggccgg	1.0	0.887
	V$BARBIE.01	-2170	(-)	tgggAAAGcaggtgg	1.0	0.909
V$BCDF	V$CRX.03	-1405	(+)	ataaataATCCcagcc	1.0	0.963
	V$PCE1.01	-324	(+)	cctatTAATtgaattcc	1.0	0.947
	V$PCE1.01	-459	(+)	ctgatTAATtgctttat	1.0	0.916
	V$PCE1.01	-1208	(+)	gttatTAATtggttact	1.0	0.938
	V$PCE1.01	-1982	(+)	tatctTAATtgatttta	1.0	0.957
	V$PCE1.01	-2308	(-)	gacagTAATtggagaag	1.0	0.907
	V$PTX1.01	-1609	(+)	atggCTAAccctctaag	1.0	0.945
V$BCL6	V$BCL6.02	-1948	(+)	cttatctTAGAaaaggg	1.0	0.851
	V$BCL6.04	-992	(+)	ttgTTCCtagacatgag	1.0	0.981
V$BEDF	V$ZBED4.01	-16	(-)	gcgGGGCggtggctc	1.0	0.960
	V$ZBED4.01	-47	(+)	gccGGGCggggctgg	1.0	0.942
	V$ZBED4.01	-76	(-)	gcgGGGCggcgcccc	1.0	0.964
	V$ZBED4.01	-275	(+)	agaGGGCgggggaca	1.0	0.969
V$BHLH	V$BHLHA15.01	-630	(-)	agttcaTATGttg	1.0	0.899
	V$MESP1_2.01	-631	(+)	tcaaCATAtgaac	1.0	0.959
V$BPTF	V$FAC1.01	-2022	(+)	caaatAACAca	1.0	0.971
V$BRN5	V$BRN5.01	-1412	(+)	aacttgCATAaataatccccagc	1.0	0.920
	V$BRN5.04	-467	(+)	agagttgtctgATTAattgcttt	1.0	0.873
	V$BRN5.04	-1627	(+)	ctacttttcttATTAccaatggc	1.0	0.863
	V$POU6F2.01	-332	(+)	taaagcaacctATTAattgaatt	1.0	0.802
V$BRNF	V$BRN2.01	-510	(-)	caCATTatatatatatata	1.0	0.893
	V$BRN2.03	-1100	(+)	cccccctaATTTtttttt	1.0	0.941
	V$BRN2.03	-1460	(-)	aaccaatttATTTgaatgg	1.0	0.984
	V$BRN2.03	-1980	(+)	tcttaattgATTTtattgg	1.0	0.960

	V$BRN2.03	-2345	(-)	tatttatttATTTtatttt	1.0	0.957
	V$BRN3.01	-1228	(-)	taaagataaATTAtttcct	1.0	0.868
	V$BRN3.02	-326	(+)	aacctatTAATtgaattcc	1.0	0.931
	V$BRN3.02	-461	(+)	gtctgatTAATtgctttat	1.0	0.902
	V$BRN3.02	-1210	(+)	atgttatTAATtggttact	1.0	0.916
	V$BRN3.03	-464	(-)	aagcaatTAATcagacaac	1.0	0.915
	V$BRN3.03	-1213	(-)	aaccaatTAATaacataaa	1.0	0.910
	V$BRN4.01	-1214	(+)	ctttatgttatTAATtggt	1.0	0.902
	V$BRN4.01	-2335	(+)	aaataaataaaTAATcggc	1.0	0.895
	V$TST1.01	-329	(-)	attcaATTAataggttgct	1.0	0.906
	V$TST1.01	-668	(-)	gatgtATTAggaagtggag	1.0	0.934
V$BTBF	V$KAISO.01	-1350	(-)	gttcCTGCaac	1.0	0.986
	V$KAISO.01	-1854	(-)	gatcCTGCtat	1.0	0.998
V$BZIP	V$CEBPE_ATF4.02	-1412	(-)	atttatGCAAgtt	1.0	0.860
V$CAAT	V$ACAAT.01	-724	(+)	ggcaCCAAtctcatt	1.0	0.875
	V$ACAAT.01	-1454	(-)	gcaaCCAAtttattt	1.0	0.853
	V$CAAT.01	-1207	(-)	gtaaCCAAttaataa	1.0	0.943
	V$NFY.04	-1925	(-)	ttacCCAAtccaaag	1.0	0.932
	V$NFY.04	-1972	(-)	gaacCCAAtaaaatc	1.0	0.936
V$CART	V$ISX.01	-2307	(+)	ttctccAATTactgtctttct	1.0	0.873
	V$MIXL1.01	-1217	(-)	ccaatTAATaacataaagata	1.0	0.832
	V$PHOX2.01	-741	(-)	tgcccTAATaaaagaggccgg	1.0	0.944
	V$PHOX2A.01	-464	(-)	taaagcAATTaatcagacaac	1.0	0.845
	V$PHOX2A.01	-1213	(-)	gtaaccAATTaataacataaa	1.0	0.891
	V$PROP1.01	-1987	(-)	aaaatcAATTaagatatcatt	1.0	0.882
	V$PROP1.02	-324	(-)	gagaggaattcaATTAatagg	1.0	0.892
	V$S8.01	-324	(+)	cctatTAATtgaattcctctc	1.0	1.000
	V$S8.01	-459	(+)	ctgatTAATtgctttataagg	1.0	0.995
	V$S8.01	-1208	(+)	gttatTAATtggttactataa	1.0	1.000
	V$S8.01	-1982	(+)	tatctTAATtgattttattgg	1.0	1.000
	V$S8.01	-2312	(-)	gacagTAATtggagaagaaag	1.0	1.000
	V$VSX1.01	-329	(-)	gaattcAATTaataggttgct	1.0	0.870
	V$VSX1.01	-1227	(+)	ggaaatAATTtatctttatgt	1.0	0.834
V$CDXF	V$CDX1.01	-739	(+)	ggcctctTTTAttagggca	1.0	0.976
	V$CDX1.01	-1504	(+)	ataccttTTTActacccag	1.0	0.987
	V$CDX2.02	-614	(-)	ctgaatgtTTATatcccgc	1.0	0.866
	V$CDX2.02	-890	(+)	atatacatTTATttcccac	1.0	0.915
	V$CDX2.02	-920	(-)	tacccagtTTATgatattt	1.0	0.851
	V$CDX2.02	-1918	(-)	acggctctTTATtacccaa	1.0	0.947
	V$CDX2.03	-1554	(+)	cttaagatTTATagcattt	1.0	0.932
	V$CDX2.03	-1839	(+)	tgttagctTTATggggcat	1.0	0.952
	V$CDX2.03	-1907	(+)	agagccgtTTATtgcatgt	1.0	0.988
	V$CDX2.03	-1976	(+)	aattgattTTATtgggttc	1.0	0.987
V$CEBP	V$CEBPB.01	-636	(-)	catatgttGAAAtcc	1.0	0.943
	V$CEBPB.01	-2165	(-)	aaacttggGAAAgca	1.0	0.947
	V$CEBPB.02	-489	(+)	tttccTGTGtaaaat	1.0	0.933
	V$CEBPB.02	-1378	(+)	ttctaTGTGcaagcc	1.0	0.944
V$CHOP	V$CHOP.01	-1901	(-)	acatGCAAtaaac	1.0	0.911
V$CHRF	V$CHR.01	-1420	(-)	aagtTTGAactct	1.0	0.966
	V$CHR.01	-1461	(-)	ttatTTGAatgga	1.0	0.979
	V$CHR.01	-2028	(-)	ttatTTGAaatag	1.0	0.955
V$CIZF	V$NMP4.01	-1086	(-)	ggAAAAaaaaa	1.0	0.978
	V$NMP4.01	-1239	(+)	tgAAAAactgc	1.0	0.971
V$CLOX	V$CDP.02	-726	(+)	agggcacCAATctcattcataag	1.0	0.968
	V$CDP.02	-1213	(-)	tagtaacCAATtaataacataaa	1.0	0.960
	V$CDP.02	-1618	(+)	ttattacCAATggctaaccctct	1.0	0.954
	V$CDP.02	-2477	(-)	ccctggcCAATttttagtagaga	1.0	0.944
	V$CDPCR3HD.01	-421	(+)	ggcctattGATCtagaataagta	1.0	0.950
	V$CDPCR3HD.01	-1474	(-)	tttgaatgGATCacccatagtaa	1.0	0.955
	V$CPHX.01	-700	(-)	agTGATtaggtcataggggcagg	1.0	0.880
	V$CPHX.01	-1466	(+)	ggTGATccattcaaataaattgg	1.0	0.856
	V$CPHX.01	-1765	(-)	ccTGATtgtatgatcagacatgc	1.0	0.862
V$CP2F	V$CP2.01	-825	(+)	gtCTGGtgagggcctgctt	1.0	0.910
	V$TCFCP2L1.01	-2223	(+)	ttCTGGctctaactgaagt	1.0	0.873
V$CREB	V$CREB2.01	-696	(+)	cccctaTGACctaatcacttc	1.0	0.906
	V$E4BP4.01	-936	(+)	ttgggcttctGTAAcaaaata	1.0	0.838
	V$E4BP4.01	-972	(+)	gcttggataaGTAAatcattt	1.0	0.802
	V$E4BP4.01	-1143	(-)	actggcttatGTAAactatgg	1.0	0.947

	V$E4BP4.01	-2000	(-)	atatcattgaGTAActtctat	1.0	0.802
V$CSEN	V$DREAM.01	-588	(-)	ctGTCAgggtg	1.0	0.985
V$CTCF	V$CTCF.01	-54	(+)	gaggcgggccgggcGGGGctgggagta	1.0	0.855
	V$CTCF.01	-150	(+)	tgcggcggcggctcGGGGtgtctggcc	1.0	0.811
	V$CTCF.05	-288	(+)	caggtgattctggAGAGggcgggggac	1.0	0.865
V$DEAF	V$NUDR.01	-175	(+)	ccgTCGGgagcttcctggt	1.0	0.842
V$DICE	V$DICE.01	-100	(-)	agtcCTCTccccgga	1.0	0.803
V$DLXF	V$DLX1.02	-323	(-)	agaggaattcAATTaatag	1.0	0.885
	V$DLX1.02	-1207	(-)	tatagtaaccAATTaataa	1.0	0.890
	V$DLX2.01	-458	(-)	cttataaagcAATTaatca	1.0	0.930
	V$DLX2.01	-2311	(+)	tttcttctccAATTactgt	1.0	0.931
	V$DLX3.01	-328	(+)	gcaacctatTAATtgaatt	1.0	0.941
	V$DLX3.01	-463	(+)	ttgtctgatTAATtgcttt	1.0	0.982
	V$DLX3.01	-1182	(-)	gttcctaaaTAATtcctgt	1.0	0.916
	V$DLX3.01	-1212	(+)	ttatgttatTAATtggtta	1.0	0.947
	V$DLX3.01	-1986	(+)	atgatatctTAATtgattt	1.0	0.947
	V$DLX3.01	-2306	(-)	gaaagacagTAATtggaga	1.0	0.947
V$DMRT	V$DMRT3.01	-935	(-)	atattttgtTACAgaagccca	1.0	0.858
V$DMTF	V$DMP1.02	-2087	(-)	tctacGGATgcagag	1.0	0.969
V$DUXF	V$DUX4.01	-692	(-)	agtGATTaggtcata	1.0	0.891
	V$DUX4.01	-967	(-)	aatGATTtacttatc	1.0	0.848
V$E2FF	V$E2F.02	-622	(-)	atatcccgcCAAAgttc	1.0	0.897
	V$E2F.02	-1818	(+)	agttggcacCAAAgtat	1.0	0.849
	V$E2F.03	-208	(-)	gagggGCGCgacctccg	1.0	0.853
	V$E2F1.01	-47	(+)	gccgGGCGgggctggga	1.0	0.975
	V$E2F1.01	-73	(-)	ccgaGGCGgggcggcgc	1.0	0.981
	V$E2F1.01	-275	(+)	agagGGCGggggacaga	1.0	0.972
	V$E2F1.01	-619	(+)	ctttGGCGggatataaa	1.0	0.971
	V$E2F3.02	-81	(-)	gggcgGCGCccctgccc	1.0	0.870
	V$E2F4.01	-56	(+)	gagagGCGGgccgggcg	1.0	0.966
	V$E2F7.02	-865	(+)	gaaggtcGGGAaatcca	1.0	0.876
	V$E2F7.02	-2149	(-)	cacaggaGGGAaaggag	1.0	0.894
	V$E2F7.02	-2198	(-)	gtcaggaGGGAagctgc	1.0	0.876
V$EBOX	V$ATF6.01	-1394	(+)	ccagCCACactgtgact	1.0	0.951
	V$MYCMAX.03	-127	(-)	cgagcccCGCGccggcc	1.0	0.919
V$EGRF	V$EGR1.02	-75	(-)	ccgaggcgGGGCggcgccc	1.0	0.889
	V$EGR1.02	-162	(-)	cgccgcagGGGCgaccagg	1.0	0.884
	V$EGR1.04	-153	(+)	ccctgcggcGGCGgctcgg	1.0	0.876
	V$EGR1.04	-280	(+)	tctggagagGGCGgggac	1.0	0.908
V$EREF	V$ESR2.01	-701	(-)	ttagGTCAtaggggcaggg	1.0	0.916
V$ESRR	V$ESRRA.03	-1535	(+)	tccctggagaccAAGGcattcc	1.0	0.922
	V$ESRRA.03	-2189	(-)	gtggctgtccagAAGGtcaggag	1.0	0.949
V$ETSF	V$ERG.02	-497	(-)	tttacacaGGAAattcacatt	1.0	0.962
	V$ERG.02	-1235	(+)	aaactgcaGGAAataatttat	1.0	0.949
	V$ETS2.01	-1187	(+)	gggctacAGGAattatttagg	1.0	0.935
	V$ETV1.02	-173	(-)	ggcgaccaGGAAgctcccgac	1.0	0.987
	V$PEA3.01	-671	(-)	atgtattAGGAagtggaggcc	1.0	0.955
	V$PEA3.01	-1789	(+)	acaagtaAGGAagggaagcca	1.0	0.943
	V$SPI1.02	-771	(+)	cacaaggtGGAAgtggcaagg	1.0	0.973
	V$SPI1.02	-1117	(-)	ggggtaggGGAAgaatctttg	1.0	0.965
	V$SPI1.03	-687	(-)	aggccttgGGAAgtgattagg	1.0	0.899
	V$SPI1.04	-320	(-)	cgctgagaGGAAttcaattaa	1.0	0.905
	V$SPI1.04	-2161	(-)	ggaaaggaGGAAacttgggaa	1.0	0.905
	V$SPIB.01	-1969	(-)	cgtaaaaaGGAAcccaataaa	1.0	0.905
	V$SPIB.01	-2267	(-)	acaaaaagGGAAagtctttaa	1.0	0.888
V$EVI1	V$EVI1.02	-2291	(-)	cagaaAAGAgaaagaaa	1.0	0.899
	V$EVI1.02	-2317	(-)	tggagAAGAaagcagag	1.0	0.835
	V$EVI1.06	-541	(+)	acaaaaAGATaaggtga	1.0	0.875
	V$MEL1.01	-1426	(+)	cagatAAGAgttcaaac	1.0	0.819
	V$MEL1.03	-2249	(-)	agatgaaGATGagaaca	1.0	0.995
V$FAST	V$FAST1.01	-376	(-)	ttcaatgtcTATTccct	1.0	0.863
	V$FAST1.01	-895	(-)	ataaatgtaTATTgcct	1.0	0.902
	V$FAST1.02	-500	(+)	tataaTGTGaatttcct	1.0	0.931
	V$FAST1.02	-1935	(+)	agggaTGTGactttgga	1.0	0.811
V$FKHD	V$FHXB.01	-570	(+)	gagataATAAtacttat	1.0	0.929
	V$FREAC2.01	-583	(+)	tgacagTAAAaatgaga	1.0	0.842
	V$FREAC2.01	-1961	(-)	taagcgTAAAaaggaac	1.0	0.847
	V$FREAC7.01	-612	(+)	gggataTAAAcattcag	1.0	0.988

	V$FREAC7.01	-2338	(+)	aataaaTAAAtaaataa	1.0	0.966
	V$FREAC7.01	-2342	(+)	ataaaaTAAAtaaataa	1.0	0.966
	V$HFH1.01	-1144	(-)	cttatgTAAActatgga	1.0	0.858
	V$HNF3B.01	-2334	(+)	aataaataAATAatcgg	1.0	0.975
V$GABF	V$GAGA.01	-280	(+)	tctggAGAGggcgggggacagacgc	1.0	0.857
V$GATA	V$GATA.01	-971	(+)	cttgGATAagtaa	1.0	0.931
	V$GATA.01	-1428	(+)	ttcaGATAagagt	1.0	0.995
	V$GATA.01	-2109	(-)	cgctGATAaaagc	1.0	0.982
	V$GATA.01	-2277	(-)	taagGATAagcag	1.0	0.940
	V$GATA1.03	-1222	(-)	taaaGATAaatta	1.0	0.959
	V$GATA1.06	-538	(+)	aaaaGATAaggtg	1.0	0.990
	V$GATA2.02	-1987	(-)	ttaaGATAtcatt	1.0	0.917
	V$GATA2.03	-1951	(-)	ctaaGATAagcgt	1.0	0.982
	V$GATA3.01	-572	(+)	atgaGATAataat	1.0	0.981
	V$GATA4.01	-561	(-)	aagaGATAagtat	1.0	0.992
V$GCF2	V$LRRFIP1.01	-157	(+)	tcgCCCCtgcggcggcggc	1.0	0.826
V$GCMF	V$GCM1.01	-201	(+)	cgcgcCCCTcagtgc	1.0	0.867
	V$GCM1.01	-649	(-)	cctaaCCCTcacggt	1.0	0.886
	V$GCM1.03	-701	(+)	ccctgCCCCtatgac	1.0	0.946
	V$GCM1.03	-1111	(+)	ttcttCCCCtacccc	1.0	0.882
	V$GCM1.03	-1365	(-)	atcgtCCCCtctagg	1.0	0.847
	V$GCM1.03	-1736	(+)	caagcCCCCtaagta	1.0	0.844
V$GFI1	V$GFI1.02	-2093	(+)	tacAATCtctgcatc	1.0	0.900
	V$GFI1B.01	-687	(+)	cctAATCacttccca	1.0	0.870
	V$GFI1B.01	-1980	(-)	taaAATCaattaaga	1.0	0.868
V$GLIF	V$GLIS3.01	-1104	(+)	cctaCCCCccttaattt	1.0	0.928
V$GREF	V$ARE.01	-2021	(-)	acagctcactgTGTTattt	1.0	0.808
	V$ARE.02	-2260	(+)	ctttcccttttGTTCtca	1.0	0.927
V$GRHL	V$GRHL2.01	-1158	(-)	aaAACCaggtatc	1.0	0.913
	V$GRHL2.01	-1168	(+)	ggAACCagatgat	1.0	0.868
	V$GRHL3.01	-1157	(+)	atacctGGTTttc	1.0	0.830
V$HAML	V$AML3.01	-1338	(-)	cttaGTGGttggcac	1.0	0.865
	V$AML3.01	-1397	(-)	cagtGTGGctgggga	1.0	0.842
V$HAND	V$PARAXIS.01	-634	(+)	atttcAACAtatgaactttgg	1.0	0.866
	V$SCX.01	-2177	(-)	gggaaagcaggTGGCtgtcca	1.0	0.944
	V$TAL1BETAHEB.01	-1170	(+)	taggaacCAGAtgatacctgg	1.0	0.926
V$HBOX	V$EMX2.01	-465	(-)	agcaatTAATcagacaact	1.0	0.831
	V$EN1.01	-1458	(-)	gcaaccaaTTTAtttgaat	1.0	0.874
	V$EN1.02	-461	(-)	ataaagcAATTaatcagac	1.0	0.891
	V$EN1.02	-1100	(-)	aaaaaaaAATTaagggggg	1.0	0.878
	V$EN1.02	-1210	(-)	agtaaccAATTaataacat	1.0	0.910
	V$EN1.02	-1984	(-)	taaaatcAATTaagatatc	1.0	0.893
	V$GBX1.01	-2308	(+)	cttctccAATTactgtctt	1.0	0.871
	V$GSH1.01	-329	(+)	agcaacctatTAATtgaat	1.0	0.870
	V$GSH1.01	-2305	(-)	agaaagacagTAATtggag	1.0	0.870
	V$GSH2.01	-325	(+)	acctatTAATtgaattcct	1.0	0.957
	V$GSH2.01	-460	(+)	tctgatTAATtgctttata	1.0	0.953
	V$GSH2.01	-1209	(+)	tgttatTAATtggttacta	1.0	0.977
	V$GSH2.01	-1983	(+)	atatctTAATtgattttat	1.0	0.957
	V$GSH2.01	-2309	(-)	agacagTAATtggagaaga	1.0	0.966
	V$MEOX1.01	-1184	(+)	ctacaggAATTatttagga	1.0	0.841
V$HDBP	V$HDBP1_2.01	-55	(-)	ccccgcCCGGcccgcctct	1.0	0.892
	V$HDBP1_2.01	-131	(+)	gtctggCCGGcgcggggct	1.0	0.859
	V$HDBP1_2.01	-134	(-)	cccgcgCCGGccagacacc	1.0	0.870
V$HEAT	V$HSF1.01	-2189	(-)	aggtggctgtccAGAAggtcaggag	1.0	0.867
	V$HSF2.02	-795	(-)	gaagacacagccAGAAggctccctc	1.0	0.954
V$HICF	V$HIC1.01	-1340	(+)	ccgTGCCaaccac	1.0	0.955
	V$HIC1.01	-1820	(-)	tggTGCCaacttc	1.0	0.901
	V$HIC1.02	-730	(-)	tggTGCCctaata	1.0	0.971
V$HIFF	V$HIF1.02	-2500	(+)	ctgaccaaCGTGgagaa	1.0	0.973
V$HMTB	V$MTBF.01	-1878	(-)	gggaATTTa	1.0	0.912
	V$MTBF.01	-2022	(-)	tgttATTTg	1.0	0.922
V$HNF1	V$HNF1.01	-1995	(+)	aGTTActcaatgatatc	1.0	0.832
	V$HNF1.03	-1209	(+)	tGTTAttaattggttac	1.0	0.880
	V$HNF1.03	-1484	(+)	gGTTAgtcatttactat	1.0	0.927
	V$HNF1.04	-531	(+)	aaggtgaaGTTAaaagg	1.0	0.858
	V$HNF1.04	-1846	(+)	atccttttGTTAgcttt	1.0	0.852
V$HNF6	V$HNF6.01	-321	(-)	agaggaatTCAAttaat	1.0	0.872

	V$HNF6.01	-405	(+)	ataagtagTCAAtttgt	1.0	0.852
	V$HNF6.01	-1997	(+)	gaagttacTCAAtgata	1.0	0.845
	V$HNF6.02	-420	(-)	attctagatCAATaggc	1.0	0.968
	V$HNF6.02	-1979	(-)	caataaaatCAATtaag	1.0	0.974
	V$OC2.01	-1003	(-)	gaacaaAATCcagaaat	1.0	0.895
V$HOMF	V$BARHL2.01	-1984	(+)	gatatctTAATtgatttta	1.0	0.986
	V$BARX1.01	-444	(-)	ctcaaagcAATTcccttat	1.0	0.916
	V$BARX1.01	-2309	(+)	tcttctccAATTactgtct	1.0	0.912
	V$BARX2.01	-329	(-)	attcaatTAATaggttgct	1.0	0.968
	V$BARX2.02	-1184	(-)	tcctaaatAATTcctgtag	1.0	0.881
	V$BARX2.02	-1229	(+)	caggaaatAATTtatcttt	1.0	0.886
	V$BSX.01	-460	(-)	tataaagcAATTaatcaga	1.0	0.958
	V$HHEX.01	-465	(+)	agttgtctgatTAATtgct	1.0	0.953
	V$HHEX.01	-1214	(+)	ctttatgttatTAATtggt	1.0	0.950
	V$HHEX.01	-1988	(+)	caatgatatctTAATtgat	1.0	0.960
	V$HMX2.01	-1558	(-)	gctataaatCTTAagactt	1.0	0.918
	V$HMX2.01	-1563	(+)	actcaaagtCTTAagattt	1.0	0.913
	V$HMX2.02	-1908	(-)	catgcaatAAACggctctt	1.0	0.867
	V$HMX3.02	-325	(-)	aggaattcaaTTAAtaggt	1.0	0.979
	V$HMX3.02	-330	(+)	aagcaacctaTTAAttgaa	1.0	0.981
	V$HMX3.02	-1104	(+)	cctacccccTTAAttttt	1.0	0.937
	V$HMX3.02	-1983	(-)	ataaaatcaaTTAAgatat	1.0	0.936
	V$MSX.01	-326	(+)	aacctatTAATtgaattcc	1.0	0.978
	V$MSX.01	-1890	(-)	gaatttaTAATttgtacac	1.0	0.972
	V$MSX1.01	-1185	(+)	gctacaggAATTatttagg	1.0	0.894
	V$MSX1.01	-1209	(-)	tagtaaccAATTaataaca	1.0	0.942
	V$MSX1.01	-1228	(-)	taaagataAATTatttcct	1.0	0.902
	V$MSX3.01	-1100	(+)	ccccctTAATtttttt	1.0	0.913
	V$NOBOX.01	-461	(+)	gtctgatTAATtgctttat	1.0	0.992
	V$NOBOX.01	-1210	(+)	atgttatTAATtggttact	1.0	1.000
	V$NOBOX.01	-2308	(-)	aagacagTAATtggagaag	1.0	0.963
	V$TLX1.01	-657	(-)	accctcaCGGTgatgtatt	1.0	0.855
	V$TLX1.01	-2381	(+)	gaggttgCGGTgagccgag	1.0	0.879
V$HOXC	V$HOXA9.01	-462	(+)	tgtctGATTaattgctt	1.0	0.947
	V$HOXB4.02	-661	(-)	acggTGATgtattagga	1.0	0.857
	V$HOXB4.02	-1284	(-)	cactTGATggaattgag	1.0	0.841
	V$HOXC9.02	-1226	(+)	gaaataaTTTAtcttta	1.0	0.911
	V$HOXC9.02	-1410	(-)	gggattaTTTAtgcaag	1.0	0.923
	V$HOXC9.02	-1554	(+)	cttaagaTTTAtagcat	1.0	0.965
	V$HOXC9.02	-2334	(-)	ccgattaTTTAtttatt	1.0	0.917
	V$HOXC9.02	-2338	(-)	ttatttaTTTAtttatt	1.0	0.912
	V$HOXC9.02	-2342	(-)	ttatttaTTTAttttat	1.0	0.912
	V$MEIS1.03	-967	(-)	aaaatGATTtacttatc	1.0	0.961
	V$MEIS1.03	-1670	(+)	agtgtGATTcatccctt	1.0	0.901
V$HOXF	V$HOXA3.01	-459	(+)	ctgatTAATtgctttataa	1.0	0.874
	V$HOXA3.01	-1208	(+)	gttatTAATtggttactat	1.0	0.872
	V$HOXA5.01	-2310	(-)	gacagtAATTggagaagaa	1.0	0.841
	V$HOXB3.01	-690	(+)	tgaccTAATcacttcccaa	1.0	0.883
	V$HOXB4.01	-324	(+)	cctattAATTgaattcctc	1.0	0.871
	V$HOXB4.01	-1982	(+)	tatcttAATTgattttatt	1.0	0.881
	V$HOXB7.01	-1186	(-)	ctaaatAATTcctgtagcc	1.0	0.857
	V$HOXB7.01	-1230	(-)	aagataAATTatttcctgc	1.0	0.844
	V$HOXB8.01	-327	(-)	gaattcaATTAataggttg	1.0	0.890
	V$HOXB8.01	-462	(-)	taaagcaATTAatcagaca	1.0	0.952
	V$HOXB8.01	-1183	(+)	tacaggaATTAtttaggaa	1.0	0.832
	V$HOXC4.01	-1985	(-)	aaaatcAATTaagatatca	1.0	0.881
	V$HOXC8.01	-1921	(-)	gctctttATTAcccaatcc	1.0	0.860
	V$HOXD8.01	-328	(+)	gcaacctATTAattgaatt	1.0	0.831
	V$HOXD8.01	-463	(+)	ttgtctgATTAattgcttt	1.0	0.861
	V$HOXD8.01	-1101	(-)	aaaaaaaATTAaggggggt	1.0	0.816
	V$HOXD8.01	-1211	(-)	gtaaccaATTAataacata	1.0	0.906
	V$HOXD8.01	-1212	(+)	ttatgttATTAattggtta	1.0	0.886
	V$NANOG.01	-610	(-)	tagtctgAATGtttatatc	1.0	0.952
	V$NANOG.01	-721	(-)	ccttatgAATGagattggt	1.0	0.957
	V$NANOG.01	-1467	(-)	ttatttgAATGgatcaccc	1.0	0.965
V$HUB1	V$ZNF282.01	-2025	(+)	TTTCaaataacacag	1.0	0.802
V$IKRS	V$IK1.01	-1878	(-)	gcctGGGAattta	1.0	0.932
	V$IK2.01	-2165	(-)	acttGGGAaagca	1.0	0.984

	V$IK3.01	-862	(+)	ggtcgGGAAatcc	1.0	0.849
V$INSM	V$INSM1.01	-1400	(-)	tggctGGGGatta	1.0	0.940
V$IRFF	V$IRF2.01	-2266	(-)	tgagaacaaaaaggGAAAgtcttta	1.0	0.903
	V$IRF3.01	-815	(-)	ccctctgtgaaccaGAAAgcaggcc	1.0	0.870
	V$ISRE.02	-960	(-)	aacagactaaaagaGAAAatgattt	1.0	0.892
	V$ISRE.02	-2294	(-)	ataagcagaaaagaGAAAgaaagac	1.0	0.889
V$KLFS	V$BKLF.01	-1873	(-)	gatcaGGGTgtgcctggga	1.0	0.965
	V$EKLF.01	-591	(-)	ttttactgtcaGGGTgcta	1.0	0.926
	V$EKLF.01	-653	(+)	catcaccgtgaGGGTtagg	1.0	0.941
	V$GKLF.01	-1103	(-)	aaaaaattaAGGGgggtag	1.0	0.916
	V$GKLF.01	-1787	(+)	aagtaaggaAGGGaagcca	1.0	0.939
	V$GKLF.02	-527	(+)	tgaagttaAAAGgagggta	1.0	0.965
	V$KKLF.01	-73	(-)	tcccgaggcGGGGcggcgc	1.0	0.945
	V$KLF6.01	-76	(-)	cgaggcGGGGcggcgcccc	1.0	0.902
	V$KLF7.01	-49	(+)	gggccgGGCGgggctggga	1.0	0.947
	V$KLF7.01	-277	(+)	ggagagGGCGggggacaga	1.0	0.949
V$LEFF	V$LEF1.02	-437	(-)	ctagtctCAAAgcaatt	1.0	0.944
	V$LEF1.02	-1567	(+)	catgactCAAAgtctta	1.0	0.944
	V$LEF1.04	-323	(-)	aggaatTCAAttaatag	1.0	0.858
	V$LEF1.04	-422	(-)	tctagaTCAAtaggcct	1.0	0.889
	V$LEF1.04	-1981	(-)	ataaaaTCAAttaagat	1.0	0.926
	V$TCF7L1.01	-837	(-)	cagacatCAAAtctgct	1.0	0.868
V$LHXF	V$ISL2.01	-327	(-)	agaggaattcaATTAataggttg	1.0	0.918
	V$ISL2.01	-332	(+)	taaagcaacctATTAattgaatt	1.0	0.910
	V$ISL2.01	-462	(-)	cttataaagcaATTAatcagaca	1.0	0.947
	V$ISL2.01	-1101	(-)	aaaaaaaaaaaATTAaggggggt	1.0	0.892
	V$ISL2.01	-1211	(-)	tatagtaaccaATTAataacata	1.0	0.953
	V$ISL2.01	-1230	(-)	cataaagataaATTAtttcctgc	1.0	0.873
	V$ISL2.01	-1985	(-)	caataaaatcaATTAagatatca	1.0	0.988
	V$LHX1.01	-1986	(+)	atgatatcttAATTgattttatt	1.0	0.860
	V$LHX2.01	-1212	(+)	ttatgttattAATTggttactat	1.0	0.869
	V$LHX3.01	-466	(-)	taaagcaaTTAAtcagacaactc	1.0	0.837
	V$LHX3.01	-1215	(-)	gtaaccaaTTAAtaacataaaga	1.0	0.830
	V$LHX3.02	-511	(+)	gtatatatatataTAATgtgaat	1.0	0.869
	V$LHX3.02	-1182	(-)	tctggttcctaaaTAATtcctgt	1.0	0.827
	V$LHX3.02	-1888	(-)	gcctgggaatttaTAATttgtac	1.0	0.850
	V$LHX3.02	-2337	(+)	ataaataaataaaTAATcggctc	1.0	0.838
	V$LHX5.01	-328	(+)	gcaacctattAATTgaattcctc	1.0	0.886
	V$LHX6.01	-739	(-)	ttggtgcccTAATaaaagaggcc	1.0	0.847
	V$LHX9.01	-463	(+)	ttgtctgattAATTgctttataa	1.0	0.844
V$LTSM	V$LTSM.03	-1859	(-)	ATCCtgctatgatca	1.0	0.979
V$MAZF	V$MAZR.01	-46	(+)	ccgggcGGGGctg	1.0	0.901
V$MEF2	V$MEF2.06	-346	(+)	ttgtattctacAAATaaagcaac	1.0	0.892
	V$SL1.01	-330	(+)	aagcaacCTATtaattgaattcc	1.0	0.844
V$MEF3	V$SIX.01	-1750	(+)	caaTCAGgtgtaa	1.0	0.890
V$MIZ1	V$MIZ1.01	-276	(-)	cccgcCCTCtc	1.0	0.987
	V$MIZ1.01	-820	(-)	caggcCCTCac	1.0	0.987
	V$MIZ1.01	-2053	(-)	ttagcCCTCaa	1.0	0.968
V$MYBL	V$MYBL1.01	-1346	(-)	tagtggttggcACGGttcctg	1.0	0.810
	V$MYBL1.02	-2223	(+)	ttctggctctAACTgaagttc	1.0	0.841
	V$VMYB.02	-229	(+)	cacggaacctAACGgtggcag	1.0	0.992
	V$VMYB.05	-1909	(-)	acatgcaataAACGgctcttt	1.0	0.953
V$MYOD	V$MYOD.01	-217	(+)	cggtGGCAgcggaggtc	1.0	0.883
	V$MYOD.01	-2417	(+)	ctgaGGCAggaggagaa	1.0	0.915
	V$TCF12.01	-2174	(-)	ggaaagcaGGTGgctgt	1.0	0.989
	V$TCFE2A.02	-1169	(+)	aggaaccaGATGatacc	1.0	0.947
V$MYT1	V$MYT1.02	-626	(-)	ccaAAGTtcatat	1.0	0.891
	V$MYT1.02	-1715	(-)	acaAAGTtcatgc	1.0	0.892
V$MZF1	V$MZF1.01	-1361	(+)	gaGGGGacgat	1.0	0.992
	V$MZF1.02	-1111	(-)	taGGGGaagaa	1.0	1.000
V$NBRE	V$NBRE.01	-2189	(-)	ccagAAGGtcaggag	1.0	0.953
V$NEUR	V$MASH1.01	-2173	(+)	cagccacCTGCtttc	1.0	1.000
V$NF1F	V$NF1.01	-1349	(-)	tggTTGGcacggttcctgcaa	1.0	0.870
	V$NF1.04	-1349	(+)	ttgcaggaaccgtgCCAAcca	1.0	0.957
V$NFAT	V$NFAT.01	-1088	(-)	ctctgaGGAAaaaaaaaaa	1.0	1.000
	V$NFAT.01	-2161	(-)	aaaggaGGAAacttgggaa	1.0	0.966
	V$NFAT5.02	-1039	(-)	acctGGAAagcaccaggc	1.0	0.925
	V$NFAT5.02	-1260	(-)	ttagGGAAatgttcctgtg	1.0	0.876

V$NFKB	V$CREL.01	-172	(+)	tcgggagcTTCCtgg	1.0	0.927
	V$CREL.01	-1524	(+)	caagggcaTTCCttt	1.0	0.942
	V$NFKAPPAB65.01	-496	(+)	atgtgaatTTCCtgt	1.0	0.888
	V$NFKAPPAB65.01	-860	(-)	tcttggatTTCCcga	1.0	0.871
V$NGRE	V$IR2_NGRE.01	-1539	(-)	gtCTCCagggaaaat	1.0	0.891
V$NKX1	V$NKX11.01	-2307	(+)	ttctccAATTactgtct	1.0	0.865
	V$NKX12.01	-459	(+)	ctgattAATTgctttat	1.0	0.888
	V$NKX12.01	-1983	(-)	aaaatcAATTaagatat	1.0	0.880
	V$NKX12.01	-2308	(-)	gacagtAATTggagaag	1.0	0.880
V$NKX6	V$NKX61.01	-324	(+)	cctaTTAAttgaatt	1.0	0.927
	V$NKX61.01	-459	(+)	ctgaTTAAttgcttt	1.0	0.967
	V$NKX61.01	-1098	(+)	ccccTTAAttttttt	1.0	0.917
	V$NKX61.01	-1211	(-)	ccaaTTAAtaacata	1.0	0.956
	V$NKX61.02	-327	(-)	tcaaTTAAtaggttg	1.0	0.925
	V$NKX61.02	-1208	(+)	gttaTTAAttggtta	1.0	0.963
	V$NKX61.02	-1982	(+)	tatcTTAAttgattt	1.0	0.962
	V$NKX63.01	-462	(-)	gcaatTAATcagaca	1.0	0.894
V$NKXH	V$NKX25.01	-1278	(+)	tccatcAAGTgagtccttc	1.0	1.000
	V$NKX25.01	-2407	(-)	aggttcAAGTgattctcct	1.0	1.000
	V$NKX25.02	-324	(+)	cctatTAATtgaattcctc	1.0	0.903
	V$NKX25.02	-459	(+)	ctgatTAATtgctttataa	1.0	0.903
	V$NKX25.02	-1098	(+)	cccctTAATttttttttt	1.0	0.957
	V$NKX25.02	-1208	(+)	gttatTAATtggttactat	1.0	0.903
	V$NKX25.02	-1982	(+)	tatctTAATtgattttatt	1.0	1.000
	V$NKX25.05	-300	(-)	tcaccTGAGtgaagaattc	1.0	0.986
	V$NKX25.05	-984	(+)	agacaTGAGtgagcttgga	1.0	0.975
	V$NKX31.01	-409	(+)	tagaatAAGTagtcaattt	1.0	0.925
	V$NKX31.01	-568	(-)	agagatAAGTattattatc	1.0	0.954
	V$NKX31.01	-970	(+)	ttggatAAGTaaatcattt	1.0	0.920
	V$NKX31.01	-1732	(+)	ccccctAAGTagccagggc	1.0	0.851
V$NOLF	V$EBF1.01	-387	(+)	gtcagtTCCCtagggaatagaca	1.0	0.945
	V$OLF1.01	-388	(-)	gtctatTCCCtagggaactgact	1.0	0.948
	V$OLF1.02	-1541	(+)	gcatttTCCCtggagaccaaggg	1.0	0.887
V$NR2F	V$HNF4G.01	-629	(-)	tatatcccgccAAAGttcatatgtt	1.0	0.973
V$NR2F	V$HNF4G.01	-1718	(-)	cagagatagacAAAGttcatgccct	1.0	0.970
V$OCT1	V$OCT1.02	-1415	(-)	tttATGCaagtttga	1.0	0.891
	V$OCT1.03	-464	(+)	gttgtctgATTAatt	1.0	0.853
	V$OCT1.03	-1213	(+)	tttatgttATTAatt	1.0	0.858
	V$OCT1.04	-1142	(-)	ctTATGtaaactatg	1.0	0.830
	V$OCT1.06	-499	(+)	ataatgtgAATTtcc	1.0	0.869
	V$OCT1.06	-1984	(+)	gatatcttAATTgat	1.0	0.889
	V$POU3F3.01	-1412	(+)	aacttGCATaaataa	1.0	0.862
V$OSRF	V$OSR1.01	-2433	(-)	ccccaGTAGctgg	1.0	0.912
V$OVOL	V$OVOL1.01	-225	(-)	gccaccGTTAggttc	1.0	0.953
V$P53F	V$P53.02	-1720	(-)	gagatagacaaagttCATGccctgg	1.0	0.925
	V$P53.03	-1779	(+)	aagggaagccaaaagCATGtctgat	1.0	0.941
	V$P53.08	-1770	(-)	gattgtatgatcagaCATGcttttg	1.0	0.856
V$PARF	V$DBP.01	-921	(-)	ccagtTTATgatatttt	1.0	0.843
	V$DBP.01	-1413	(-)	attatTTATgcaagttt	1.0	0.875
	V$HLF.01	-1923	(+)	ttggattggGTAAtaaa	1.0	0.900
	V$HLF.01	-1997	(-)	tatcattgaGTAActtc	1.0	0.934
	V$TEF.01	-1140	(-)	ctggcttatGTAAacta	1.0	0.972
	V$TEF_HLF.01	-1141	(+)	atagtTTACataagcca	1.0	0.830
	V$TEF_HLF.01	-1922	(-)	ctttaTTACccaatcca	1.0	0.817
	V$VBP.01	-935	(+)	tgggcttctGTAAcaaa	1.0	0.897
V$PAX3	V$PAX3.03	-1805	(+)	gtattCATGgggatgtaca	1.0	0.954
V$PAX5	V$PAX8.01	-1331	(-)	ctaggataaacTTGAaagtgtcttagtgg	1.0	0.961
V$PAX6	V$PAX6.01	-1956	(+)	ttttACGCttatcttaga	1.0	0.854
	V$PAX6.02	-853	(+)	atccaagatCCAGgccagc	1.0	0.935
	V$PAX6.02	-2227	(-)	cagttagagCCAGaagaga	1.0	0.873
V$PAX7	V$PAX7.01	-688	(-)	gggaagtGATTaggt	1.0	0.815
	V$PAX7.01	-1979	(+)	cttaattGATTttat	1.0	0.953
V$PAXH	V$PAX6_HD.01	-458	(+)	tgattAATTgcttta	1.0	0.895
	V$PAX6_HD.01	-1207	(+)	ttattAATTggttac	1.0	0.896
	V$PAX6_HD.01	-1208	(-)	taaccAATTaataac	1.0	0.879
V$PCBE	V$PREB.01	-465	(-)	attaaTCAGacaact	1.0	0.880
	V$PREB.01	-1765	(-)	tatgaTCAGacatgc	1.0	0.885
	V$PREB.01	-1866	(-)	tatgaTCAGggtgtg	1.0	0.872

V$PDX1	V$IPF1.01	-1212	(+)	ttatgttatTAATtggtta	1.0	0.843
V$PERO	V$PPARG.03	-626	(-)	ttatatcccgccAAAGttcatat	1.0	0.854
	V$PPARG.03	-1715	(-)	tcagagatagacAAAGttcatgc	1.0	0.863
V$PIT1	V$PIT1.01	-1886	(+)	acaaaTTATaaattc	1.0	0.931
	V$PIT1.01	-2335	(-)	attatTTATttattt	1.0	0.926
	V$PIT1.01	-2339	(-)	tttatTTATttattt	1.0	0.926
	V$PIT1.02	-325	(+)	acctaTTAAttgaat	1.0	0.814
	V$PIT1.02	-460	(+)	tctgaTTAAttgctt	1.0	0.829
	V$PIT1.02	-461	(-)	agcaaTTAAtcagac	1.0	0.874
	V$PIT1.02	-1209	(+)	tgttaTTAAttggtt	1.0	0.838
	V$PIT1.02	-1210	(-)	accaaTTAAtaacat	1.0	0.876
V$PLAG	V$PLAG1.02	-1115	(-)	aaGGGGggtaggggaagaatctt	1.0	1.000
	V$PLAG1.02	-1748	(-)	taGGGGcttgcttacacctgat	1.0	1.000
	V$PLAGL1.01	-78	(+)	cagggGCGCcgccccgcctcggg	1.0	0.886
	V$PLAGL1.01	-87	(-)	gggcgGCGCccctgcccagccag	1.0	0.901
V$PLZF	V$PLZF.01	-2013	(-)	atgTACAgctcactg	1.0	0.877
V$PRDF	V$PRDM1.02	-961	(-)	taaaagaGAAAatgattta	1.0	0.858
	V$PRDM1.02	-1327	(-)	taaacttGAAAgtgtctta	1.0	0.845
	V$PRDM1.02	-2153	(-)	caggaggGAAAggaggaaa	1.0	0.844
	V$PRDM1.02	-2267	(-)	aaaaaggGAAAgtctttaa	1.0	0.955
	V$PRDM1.02	-2295	(-)	gaaaagaGAAAgaaagaca	1.0	0.893
V$PURA	V$PURALPHA.01	-1660	(-)	ggAGGCaagggat	1.0	0.971
V$RBPF	V$RBPJK.01	-681	(-)	gcctTGGGaagtg	1.0	0.897
	V$RBPJK.02	-881	(-)	actgTGGGaaata	1.0	0.982
	V$RBPJK.02	-1877	(-)	tgccTGGGaattt	1.0	0.956
	V$RBPJK.02	-2164	(-)	aactTGGGaaagc	1.0	0.955
V$RORA	V$RORA.01	-701	(-)	aagtgattagGTCAtaggggcaggg	1.0	0.900
V$RP58	V$RP58.01	-1166	(-)	gtatCATCtggtt	1.0	0.855
V$RUSH	V$SMARCA3.02	-563	(+)	taatACTTatc	1.0	0.993
	V$SMARCA3.02	-967	(-)	atttACTTatc	1.0	0.986
	V$SMARCA3.02	-1790	(-)	ccttACTTgta	1.0	0.986
V$RXRF	V$RARG.01	-932	(-)	gtttatgatatTTTGttacagaagc	1.0	0.890
	V$THRA.01	-782	(+)	ggctgtgtcttcacaAGGTggaagt	1.0	0.858
	V$VDR_RXR.05	-767	(+)	aggtgGAAGtggcaaggggtctctc	1.0	0.801
V$SATB	V$SATB1.01	-1214	(-)	attAATAacataaag	1.0	0.963
V$SF1F	V$FTF.02	-1528	(+)	agaccAAGGgcattc	1.0	0.944
	V$SF1.01	-2042	(-)	gggaCAAGgcaagtg	1.0	0.961
V$SIXF	V$SIX1.01	-1162	(-)	aaccaggTATCatct	1.0	0.934
	V$SIX1.01	-1509	(-)	aaaaaggTATCaccc	1.0	0.945
	V$SIX1.01	-1988	(-)	attaagaTATCattg	1.0	0.891
V$SMAD	V$SMAD3.01	-134	(+)	ggtGTCTggcc	1.0	0.991
	V$SMAD3.01	-2072	(-)	gctGTCTggtt	1.0	0.997
	V$SMAD3.02	-828	(+)	gatGTCTggtg	1.0	0.991
	V$SMAD4.01	-1297	(-)	gctGTCTtggc	1.0	0.943
V$SNAP	V$PSE.02	-1251	(+)	atttcCCTAagttgaaaaa	1.0	0.867
V$SORY	V$HBP1.01	-724	(-)	tccttatgAATGagattggtgcc	1.0	0.926
	V$HBP1.02	-897	(-)	ggaaataAATGtatattgcctat	1.0	0.860
	V$HBP1.02	-1471	(-)	ttatttgAATGgatcacccatag	1.0	0.936
	V$HBP1.02	-1489	(-)	catagtaAATGactaacctctgg	1.0	0.838
	V$HMGA.01	-463	(+)	ttgtctgattAATTgctttataa	1.0	0.907
	V$HMGA.01	-722	(-)	ggtccttatgAATGagattggtg	1.0	0.910
	V$HMGA.01	-894	(-)	gtgggaaataAATGtatattgcc	1.0	0.927
	V$HMGA.01	-1230	(-)	cataaagataAATTatttcctgc	1.0	0.904
	V$HMGA.01	-1459	(+)	cattcaaataAATTggttgccag	1.0	0.903
	V$HMGA.01	-1651	(-)	tagccaaatgAATTgctgtggag	1.0	0.915
	V$HMGIY.01	-495	(+)	tgtgAATTtcctgtgtaaaatgt	1.0	0.927
	V$HMGIY.01	-506	(-)	aggaAATTcacattatatatata	1.0	0.930
	V$SOX1.04	-376	(+)	aggGAATagacattgaaaagatt	1.0	0.809
	V$SOX1.04	-410	(+)	ctaGAATaagtagtcaatttgta	1.0	0.809
	V$SOX3.01	-1854	(-)	agctaaCAAAaggatcctgctat	1.0	0.974
	V$SOX3.04	-328	(-)	gagGAATtcaattaataggttgc	1.0	0.800
	V$SOX4.01	-2264	(-)	tgagaACAAaaagggaaagtctt	1.0	0.919
	V$SOX6.01	-1720	(-)	gatagACAAagttcatgccctgg	1.0	0.984
	V$SOX8.02	-1657	(-)	aatGAATtgctgtggaggcaagg	1.0	0.820
	V$SOX8.02	-1680	(-)	gatGAATcacactcttgcctttt	1.0	0.859
	V$SOX9.01	-429	(-)	ctagatCAATaggcctagtctca	1.0	0.912
	V$SOX9.09	-1573	(-)	taagactttgaGTCAtgaggcct	1.0	0.801
	V$SOX17.02	-728	(-)	tatGAATgagattggtgccctaa	1.0	0.833

	V$SOX21.03	-317	(+)	attGAATtcctctcagcgaattc	1.0	0.809
	V$SRY.02	-577	(-)	taagtATTAttatctcattttta	1.0	0.881
V$SP1F	V$GC.01	-71	(-)	tcccgaGGCGgggcggc	1.0	0.906
	V$SP1.01	-44	(+)	gggcgGGGCtgggagta	1.0	0.896
	V$SP1.03	-49	(+)	gggccGGGCggggctgg	1.0	0.960
	V$SP1.03	-76	(-)	aggcgGGGCggcgcccc	1.0	0.951
	V$SP1.03	-277	(+)	ggagaGGGCgggggaca	1.0	0.956
	V$SP1.03	-705	(-)	catagGGGCagggtcct	1.0	0.913
	V$TIEG.01	-140	(+)	gctcGGGGtgtctggcc	1.0	0.879
V$SPZ1	V$SPZ1.01	-517	(+)	aGGAGggtata	1.0	0.989
	V$SPZ1.01	-2146	(-)	aGGAGggaaag	1.0	0.966
	V$SPZ1.01	-2195	(-)	aGGAGggaagc	1.0	0.950
V$SREB	V$SREBP.03	-293	(-)	gaaTCACctgagtga	1.0	0.941
V$SRFF	V$SRF.05	-452	(+)	attgctttatAAGGgaatt	1.0	0.836
V$STAT	V$STAT.01	-552	(-)	ctttttgttGGAAgaaaga	1.0	0.876
	V$STAT.01	-1353	(+)	gatgttgcaGGAAccgtgc	1.0	0.912
	V$STAT1.02	-995	(-)	tcatgtctaGGAAcaaaat	1.0	0.855
	V$STAT1.02	-1034	(-)	actttacctGGAAaagcac	1.0	0.859
	V$STAT3.02	-168	(+)	gagcTTCCtggtcgcccct	1.0	0.954
	V$STAT3.02	-1032	(+)	gcttTTCCaggtaaagtgt	1.0	0.977
	V$STAT5A.01	-550	(+)	tttcTTCCaacaaaaagat	1.0	0.842
	V$STAT5A.01	-993	(+)	tttgTTCCtagacatgagt	1.0	0.860
	V$STAT6.01	-1267	(-)	aatgTTCCtgtgaaggact	1.0	0.927
V$STEM	V$OCT3_4.01	-1772	(+)	gccaaaaGCATgtctgatc	1.0	0.812
	V$OCT3_4.02	-2090	(+)	aatctctGCATccgtagaa	1.0	0.927
V$TAIP	V$CSRNP1.01	-1672	(+)	AGAGtgt	1.0	1.000
V$TCFF	V$TCF11.01	-1479	(+)	GTCAttt	1.0	1.000
	V$TCF11.01	-1693	(-)	GTCAttt	1.0	1.000
V$TEAF	V$TEAD.01	-716	(+)	tctCATTcataag	1.0	0.902
	V$TEAD4.01	-1521	(+)	gggcATTCctttg	1.0	0.975
V$XBBF	V$RFX1.01	-1447	(-)	gaggtagcctgGCAAccaa	1.0	0.998
	V$RFX4.03	-1447	(+)	ttgGTTGccaggctacctc	1.0	0.931
	V$RFX5.01	-339	(+)	ctacaaataaAGCAaccta	1.0	0.935
	V$XBOX.01	-385	(+)	cagttccctAGGGaataga	1.0	0.917
	V$XBOX.01	-386	(-)	ctattccctAGGGaactga	1.0	0.955
	V$XBOX.01	-681	(+)	cacttcccaAGGCctccac	1.0	0.906
V$YBXF	V$YB1.01	-1132	(-)	gggacTGGCttat	1.0	0.882
V$YY1F	V$REX1.01	-1622	(-)	ggttagCCATtggtaataagaaa	1.0	0.922
V$ZF02	V$ZBP89.01	-1108	(+)	ttcccctaCCCCccttaattttt	1.0	0.931
	V$ZBTB7.01	-1105	(+)	ccctacCCCCcttaattttttt	1.0	0.947
	V$ZBTB7.01	-1738	(+)	agcaagCCCCctaagtagccagg	1.0	0.921
	V$ZBTB7.03	-54	(-)	cccagCCCCgcccggcccgcctc	1.0	0.894
	V$ZBTB7.03	-72	(+)	cgccgCCCCgcctcgggagaggc	1.0	0.884
	V$ZBTB7.03	-282	(-)	ctgtcCCCCgccctctccagaat	1.0	0.901
	V$ZNF300.01	-2436	(-)	tcagcctCCCCagtagctgggat	1.0	0.994
V$ZF03	V$ZNF217.01	-1812	(-)	GAATactttggtg	1.0	0.904
	V$ZNF217.01	-2117	(+)	GAATccttgcttt	1.0	0.943
V$ZF05	V$ZFP410.01	-1940	(+)	agaaaagGGATgtga	1.0	0.851
V$ZF10	V$PRDM14.01	-1253	(-)	aacTTAGggaaatgt	1.0	0.852
	V$PRDM14.01	-2223	(-)	cagTTAGagccagaa	1.0	0.879
V$ZF11	V$ZBTB3.01	-89	(-)	cccaGCCAgtc	1.0	0.991
	V$ZBTB3.01	-786	(-)	cacaGCCAgaa	1.0	0.993
V$ZF12	V$ZFP652.01	-480	(+)	taaaatgtgTTAAag	1.0	0.833
	V$ZFP652.01	-1562	(+)	ctcaaagtcTTAAga	1.0	0.806
V$ZF5F	V$ZF5.01	-239	(+)	ccgggaGCGCcacgg	1.0	0.957
	V$ZF5.02	-190	(+)	gtgccCGCGctctcc	1.0	0.843
	V$ZF5.02	-191	(-)	gagagCGCGggcact	1.0	0.844
V$ZFHX	V$DELTAEF1.01	-291	(-)	gaatcACCTgagt	1.0	0.991
	V$DELTAEF1.01	-534	(-)	acttcACCTtatc	1.0	0.994
V$ZFXY	V$ZFX.01	-846	(-)	ctGGCCtggat	1.0	0.974
	V$ZFX.01	-1042	(+)	ttGGCCtggtg	1.0	0.972
V$ZTRE	V$ZTRE.01	-66	(+)	cccgcctcGGGAgaggc	1.0	0.854
	V$ZTRE.03	-2398	(-)	caCTCCcaggttcaagt	1.0	0.976
	V$ZTRE.04	-2390	(+)	ctgGGAGtggaggttgc	1.0	0.978

Intron 1

O$MTEN	O$DMTE.01	+373	(-)	gccgcagAGCGcaggcggcgg	1.0	0.826
	O$HMTE.01	+206	(+)	ggAGCGgggcggcgggcagcg	1.0	0.994
O$PTBP	O$PTATA.01	+1633	(+)	agttTATAaacattt	1.0	0.910
	O$PTATA.02	+3120	(-)	acacTATAgatatgt	1.0	0.922
	O$PTATA.02	+4156	(+)	acccTATAtatactg	1.0	0.966
	O$PTATA.02	+4157	(-)	acagTATAtataggg	1.0	0.928
	O$PTATA.02	+5958	(+)	atgaTATAaataata	1.0	0.949
	O$PTATA.02	+6230	(+)	tgtgTATAaatgaag	1.0	0.903
	O$PTATA.02	+7303	(+)	caggTATAtatattt	1.0	0.920
	O$PTATA.02	+7304	(-)	taaaTATAtatacct	1.0	0.947
O$TELO	O$ZSCAN4.01	+5873	(-)	tGCACaggctggagt	1.0	0.816
O$TF2B	O$BRE.01	+66	(-)	ccgCGCC	1.0	1.000
	O$BRE.01	+123	(-)	ccgCGCC	1.0	1.000
O$TF3C	O$TFIIIC.01	+521	(+)	GGTTggagccc	1.0	0.882
	O$TFIIIC.01	+1540	(-)	GGTTcgagacc	1.0	0.988
O$VTBP	O$ATATA.01	+2240	(-)	tggcattTAAGgtcaac	1.0	0.822
	O$ATATA.01	+2264	(-)	aagtacaTAAGtttaca	1.0	0.892
	O$ATATA.01	+2408	(-)	tggttctTAAGttaccc	1.0	0.812
	O$LTATA.01	+3709	(+)	catTATAattagcaaat	1.0	0.825
	O$LTATA.01	+4313	(-)	agtTATAatgacattct	1.0	0.828
	O$LTATA.01	+5364	(-)	ccgTATAagttgctact	1.0	0.890
	O$LTATA.01	+5376	(-)	ccgTATAagaaaccgta	1.0	0.863
	O$LTATA.01	+7414	(-)	aagTATAactggattgt	1.0	0.831
	O$MTATA.01	+1627	(-)	gtttaTAAActtgaggt	1.0	0.865
	O$MTATA.01	+2147	(-)	ctatgTAAAaaggtacc	1.0	0.848
	O$MTATA.01	+5441	(-)	cttttTAAAgcaaaagt	1.0	0.863
	O$VTATA.01	+1634	(+)	gtttaTAAAcatttcat	1.0	0.905
	O$VTATA.01	+4645	(-)	ccttaTAAAgggtccta	1.0	0.928
	O$VTATA.01	+5959	(+)	tgataTAAAtaatagca	1.0	0.938
	O$VTATA.01	+6213	(+)	ctgtaTAAAatatccag	1.0	0.956
	O$VTATA.01	+6231	(+)	gtgtaTAAAtgaagcat	1.0	0.957
	O$VTATA.02	+1668	(+)	ttccaTAAAaggtaaag	1.0	0.960
	O$VTATA.02	+3922	(+)	tacttTAAAatttcaaa	1.0	0.910
	O$VTATA.02	+4696	(-)	ttcaaTAAAaaccaaaa	1.0	0.931
	O$VTATA.02	+5446	(+)	tgcttTAAAaagccaag	1.0	0.909
	O$VTATA.02	+6298	(-)	aaccaTAAAatattacc	1.0	0.943
O$XCPE	O$XCPE1.01	+318	(+)	ggGCGGcacgg	1.0	0.816
	O$XCPE1.01	+449	(-)	caGCGGgacct	1.0	0.823
O$YTBP	O$SPT15.01	+4155	(+)	aaccctaTATAtactgt	1.0	0.900
	O$SPT15.01	+4156	(-)	aacagtaTATAtagggt	1.0	0.892
	O$SPT15.01	+4158	(-)	aaaacagTATAtatagg	1.0	0.834
	O$SPT15.01	+7302	(+)	gcaggtaTATAtattta	1.0	0.975
	O$SPT15.01	+7303	(-)	ataaataTATAtacctg	1.0	0.908
	O$SPT15.01	+7304	(+)	aggtataTATAtttatg	1.0	0.834
	O$SPT15.01	+7305	(-)	ccataaaTATAtatacc	1.0	0.936
V$ABDB	V$HOXA9.02	+7310	(-)	gtatgccaTAAAtatat	1.0	0.890
	V$HOXB9.01	+1099	(+)	aatgtttgTAAAgtgac	1.0	0.891
	V$HOXB9.01	+1813	(+)	caacttccTAAAttcat	1.0	0.891
	V$HOXB9.01	+2240	(+)	gttgacctTAAAtgcca	1.0	0.889
	V$HOXB9.02	+6301	(-)	aaaaaccaTAAAatatt	1.0	0.957
	V$HOXB9.02	+7259	(-)	aaaaaaaaTAAAagccg	1.0	0.881
	V$HOXC10.01	+8083	(-)	ttttatccTAAAagctt	1.0	0.841
	V$HOXC13.01	+2387	(+)	tcttctcatTAAAttct	1.0	0.925
	V$HOXC13.01	+3688	(-)	tttaaccaaTAAAgtgt	1.0	0.951
	V$HOXC13.01	+4579	(-)	aaagtcaaaTAAAgaag	1.0	0.913
	V$HOXC13.01	+6644	(-)	gggatccaaTAAAtgtc	1.0	0.959
	V$HOXC13.01	+7035	(-)	tattccaaaTAAAatat	1.0	0.938
	V$HOXC13.01	+8061	(-)	aatttccatTAAActga	1.0	0.961
	V$HOXC13.02	+1577	(+)	cggcctccTAAAgtgct	1.0	0.890
	V$HOXC13.02	+2258	(+)	gtggcttgTAAActtat	1.0	0.865
	V$HOXC13.02	+2788	(-)	tctgctcaTAAAggtcc	1.0	0.928
	V$HOXC9.01	+2035	(+)	agcggtctTTAAactat	1.0	0.859
	V$HOXC9.01	+3278	(-)	caagattaTTAAcctca	1.0	0.845
	V$HOXC9.01	+3877	(-)	ttgtgaaaTTAAttctc	1.0	0.848
	V$HOXC9.01	+3894	(+)	tgaagtaaTTAAcactt	1.0	0.923
	V$HOXC9.01	+5211	(+)	atgtgtaaTTAAaggaa	1.0	0.907
	V$HOXD13.01	+1665	(+)	tgtttccaTAAAaggta	1.0	0.921
	V$HOXD13.01	+4699	(-)	tggttcaaTAAAaacca	1.0	0.974

V$AHRR	V$AHRARNT.03	+885	(+)	gttggtcctaGCGTgcagagcccta	1.0	0.971
	V$NXF_ARNT.01	+337	(+)	ctgcggagcctCGTGggcttcgccg	1.0	0.904
V$AIRE	V$AIRE.02	+4501	(+)	tttggttagaTTGGa	1.0	0.915
	V$AIRE.02	+4790	(+)	ccaggggataTTGGc	1.0	0.813
V$AP1F	V$AP1.01	+7178	(-)	ctatgAGTCagaa	1.0	1.000
	V$AP1.02	+2941	(-)	acatGAGTtagaa	1.0	0.884
	V$AP1.02	+3863	(+)	agatGAGTaagtc	1.0	0.938
	V$AP1.02	+6687	(+)	gattGAGTaaata	1.0	0.890
	V$BATF.01	+1157	(+)	cagtgaCTCAggc	1.0	0.974
	V$BATF.01	+7178	(+)	ttctgaCTCAtag	1.0	0.988
	V$JUNB.01	+1157	(-)	gcctgaGTCActg	1.0	0.988
	V$JUNB.01	+2517	(+)	agatcaGTCAtga	1.0	0.924
	V$JUNB.01	+3867	(+)	gagtaaGTCAgag	1.0	0.913
	V$JUNB.01	+5932	(+)	attttaGTCAaag	1.0	0.913
	V$JUNB.01	+7999	(-)	gcctaaGTCAgta	1.0	0.910
V$AP1R	V$BACH1.01	+1923	(-)	taacttagTGAGgcatcctcta	1.0	0.825
	V$BACH2.01	+7173	(-)	agatgctaTGAGtcagaatgtgt	1.0	0.970
	V$BACH2.02	+1152	(-)	agacagccTGAGtcactgaggtc	1.0	0.980
	V$MAFA.01	+471	(+)	ggaggggctcgctcAGCAaacca	1.0	0.984
	V$MAFA.01	+731	(-)	ttttctgtgggatcAGCAaccag	1.0	0.923
	V$MAFA.01	+2842	(-)	ttctaggcccacccAGCAatcag	1.0	0.955
	V$MAFB.01	+1842	(+)	tgatgtttaaaaTCAGcacttag	1.0	0.846
	V$MAFF.01	+471	(-)	tggtttGCTGagcgagcccctcc	1.0	0.901
	V$MAFF.01	+731	(+)	ctggttGCTGatcccacagaaaa	1.0	0.842
	V$MAFF.01	+1842	(-)	ctaagtGCTGattttaaacatca	1.0	0.835
	V$MAFF.01	+5664	(+)	tgggatGCTGaggtgggaggatc	1.0	0.836
	V$MAFK.01	+1846	(+)	gtttaaaatcAGCActtagtcca	1.0	0.870
	V$MAFK.01	+2976	(-)	acctacagtaAGCAgttcagtgt	1.0	0.835
	V$MAFK.01	+4734	(-)	ctagctagttAGCAgtgtatccc	1.0	0.833
	V$MAFK.01	+6071	(-)	atctccagtgAGCAtttctgtag	1.0	0.844
	V$MARE.03	+2665	(-)	gtccttGCTGaaacacagagcag	1.0	0.832
	V$NFE2.01	+7173	(+)	acacattCTGActcatagcatct	1.0	0.900
	V$NRL.02	+475	(+)	gggctcgctcAGCAaaccagacg	1.0	0.972
	V$NRL.02	+5026	(-)	caggatgttcAGCAgcattgctg	1.0	0.982
	V$TCF11MAFG.01	+3717	(-)	cattttgtataGTCAtttgctaa	1.0	0.812
	V$TCF11MAFG.01	+7647	(-)	tgatctgcatgGTCAaaggggcc	1.0	0.842
	V$VMAF.01	+3650	(-)	gggtatgccccGTCAacttccaa	1.0	0.823
V$AP2F	V$AP2.02	+2733	(+)	agtGCCTaggggctc	1.0	0.935
	V$AP2.02	+5189	(+)	gttGCCTtagggtgg	1.0	0.955
	V$AP2.02	+6801	(+)	catGCCTcaggggcc	1.0	0.968
	V$AP2.02	+6931	(-)	tttGCCTaaggggtg	1.0	0.956
	V$TCFAP2A.02	+4119	(+)	acccCCTGtggatgc	1.0	0.941
	V$TCFAP2A.02	+6801	(-)	ggccCCTGaggcatg	1.0	0.978
	V$TCFAP2B.01	+759	(+)	cttGCCCcatggcct	1.0	0.894
	V$TCFAP2B.01	+2734	(-)	tgaGCCCctaggcac	1.0	0.906
	V$TCFAP2B.01	+6656	(-)	tcgGCCCcaagggat	1.0	0.894
	V$TCFAP2E.01	+5189	(-)	ccaccctaAGGCaac	1.0	0.911
	V$TCFAP2E.01	+6931	(+)	cacccettAGGCaaa	1.0	0.914
V$AP4R	V$AP4.01	+636	(+)	agcatCAGCtgaggctg	1.0	0.892
	V$AP4.02	+635	(-)	agcctcAGCTgatgctc	1.0	0.969
	V$AP4.02	+698	(-)	ggccgcAGCTggtcccg	1.0	0.960
	V$AP4.02	+6848	(-)	cttcacAGCTgttgtag	1.0	0.979
	V$AP4.03	+699	(+)	gggaccaGCTGcggccg	1.0	0.986
	V$AP4.03	+6811	(+)	gggcccaGCTGccctcc	1.0	0.982
V$ARID	V$ARID5A.01	+1819	(-)	tctaaATATtatgaatttagg	1.0	0.940
	V$ARID5A.01	+1826	(+)	tcataATATttagaggtgatg	1.0	0.969
	V$ARID5A.01	+1890	(-)	gctaaATATtcaaggaccatc	1.0	0.936
	V$ARID5A.01	+1897	(+)	cttgaATATttagcacttaga	1.0	0.940
	V$ARID5A.01	+3743	(-)	attgaATATtgaatacaagaa	1.0	0.960
	V$ARID5A.01	+3750	(-)	aaataATATtgaatattgaat	1.0	0.989
	V$ARID5A.01	+3750	(+)	attcaATATtcaatattattt	1.0	0.946
	V$ARID5A.01	+3757	(+)	attcaATATtattttgtgctt	1.0	0.952
	V$ARID5A.01	+4547	(-)	atttaATATttattcttttca	1.0	0.970
	V$ARID5A.01	+4554	(+)	aataaATATtaaattatggaa	1.0	0.949
	V$ARID5A.01	+4885	(-)	cgggaATATtgcttgagccca	1.0	0.964
	V$ARID5A.01	+4892	(+)	aagcaATATtcccgcctcagc	1.0	0.944
	V$ARID5A.01	+5918	(-)	ctaaaATATttttgcacatct	1.0	0.941
	V$ARID5A.01	+5925	(+)	caaaaATATtttagtcaaagg	1.0	0.940

	V$ARID5A.01	+6290	(-)	ataaaATATtaccttattttt	1.0	0.945
	V$ARID5A.01	+6297	(+)	aggtaATATtttatggttttt	1.0	0.963
	V$ARID5A.01	+6505	(-)	gggcaATATggcaaaaccctg	1.0	0.880
	V$ARID5A.01	+6961	(-)	aaaaaATATttgggtctaaga	1.0	0.942
	V$ARID5A.01	+6968	(+)	cccaaATATttttttccttca	1.0	0.951
	V$ARID5A.01	+7023	(-)	ataaaATATttggcagtacaa	1.0	0.942
	V$ARID5A.01	+7030	(+)	gccaaATATtttatttggaat	1.0	0.950
	V$BRIGHT.01	+2909	(+)	taccaATTAacagccgattaa	1.0	0.926
	V$BRIGHT.01	+3876	(+)	agagaATTAatttcacaatga	1.0	0.935
	V$BRIGHT.01	+3896	(+)	aagtaATTAacactttcaaag	1.0	0.929
	V$BRIGHT.01	+4613	(-)	ttcaaATTAagatgtgtttaa	1.0	0.921
	V$BRIGHT.01	+5213	(+)	gtgtaATTAaaggaactaagc	1.0	0.945
	V$BRIGHT.01	+5299	(+)	tctgaATTAaaaattttcatt	1.0	0.953
	V$BRIGHT.01	+7264	(-)	taagaATTAaaaaaaaataaa	1.0	0.949
	V$JARID2.01	+2916	(-)	gaccatTTTAatcggctgtta	1.0	0.938
	V$JARID2.01	+6207	(-)	ggatatTTTAtacaggttgag	1.0	0.891
V$ATBF	V$ATBF1.01	+938	(+)	gtttattctgAATTatc	1.0	0.816
	V$ATBF1.01	+1209	(-)	ttttcttcagAATTaac	1.0	0.802
	V$ATBF1.01	+2903	(+)	aaaagctaccAATTaac	1.0	0.833
	V$ATBF1.01	+3712	(-)	gtcatttgctAATTata	1.0	0.817
	V$ATBF1.01	+3829	(+)	atttgtttacAATTtca	1.0	0.808
	V$ATBF1.01	+7267	(+)	attttttttAATTctt	1.0	0.814
	V$ATBF1.01	+7274	(-)	aaagattaagAATTaaa	1.0	0.866
	V$ATBF1.01	+7453	(-)	tatagtcaatAATTtca	1.0	0.830
V$BARB	V$BARBIE.01	+241	(-)	aatcAAAGgcgactg	1.0	0.882
	V$BARBIE.01	+623	(-)	ctcaAAAGgggccgg	1.0	0.902
	V$BARBIE.01	+5435	(-)	agcaAAAGttgtgag	1.0	0.892
	V$BARBIE.01	+7255	(-)	aataAAAGccgaagg	1.0	0.940
V$BCDF	V$CRX.01	+2917	(-)	catttTAATcggctgtt	1.0	0.974
	V$CRX.01	+6106	(-)	atcccTAATctgaagag	1.0	0.958
	V$CRX.03	+1589	(-)	gcctataATCCcagcac	1.0	0.961
	V$CRX.03	+8021	(-)	agttctaATCCatctct	1.0	0.981
	V$DMBX1.01	+4921	(-)	acctgtAATCccagtca	1.0	0.936
	V$PCE1.01	+509	(+)	ttctcTAATtggggttg	1.0	0.960
	V$PCE1.01	+1231	(-)	gtagtTAATtgtatcag	1.0	0.923
	V$PCE1.01	+2906	(-)	gctgtTAATtggtagct	1.0	0.944
	V$PCE1.01	+3479	(+)	ctggcTAATtctttgta	1.0	0.905
	V$PCE1.01	+5296	(-)	attttTAATtcagaaaa	1.0	0.891
	V$PTX1.01	+2179	(+)	gactCTAAaccttaact	1.0	0.952
	V$PTX1.01	+7098	(-)	ggagCTAAgcctcaaag	1.0	0.955
V$BCL6	V$BCL6.01	+1645	(-)	aatTTCCtagtatgaaa	1.0	0.806
	V$BCL6.02	+6492	(+)	tttttcaTAGAgacagg	1.0	0.802
	V$BCL6.02	+7131	(+)	tccttctTAGAagtaag	1.0	0.845
	V$BCL6.04	+1110	(-)	caaTTCCcagagtcact	1.0	0.894
	V$BCL6.04	+2548	(+)	accTTCCttgatgccag	1.0	0.948
	V$BCL6.04	+4109	(+)	gcaTTCCaagacccccct	1.0	0.891
	V$BCL6.04	+7363	(-)	attTTCCatgatgtgat	1.0	0.880
V$BEDF	V$ZBED4.01	+209	(+)	gcgGGGCggcgggca	1.0	0.964
	V$ZBED4.02	+552	(+)	cggccgcGGGGgagc	1.0	0.937
	V$ZBED4.02	+1500	(+)	ggtggtgGGGGgggg	1.0	0.978
	V$ZBED4.02	+1503	(+)	ggtggggGGGGgggg	1.0	0.989
	V$ZBED4.02	+1505	(+)	tggggggGGGGgggt	1.0	0.993
	V$ZBED4.02	+1507	(+)	gggggggGGGGgtcc	1.0	0.936
V$BHLH	V$BHLHA15.01	+4061	(+)	tttcctTATGgtg	1.0	0.827
	V$MESP1_2.01	+5007	(+)	gcagCATAtgtat	1.0	0.952
	V$MESP1_2.01	+5008	(-)	aataCATAtgctg	1.0	0.947
	V$MESP1_2.01	+6241	(+)	gaagCATAtgtta	1.0	0.973
	V$MESP1_2.01	+6242	(-)	ttaaCATAtgctt	1.0	0.968
V$BNCF	V$BNC.01	+1139	(-)	gaggtccaggTGTCctcat	1.0	0.876
	V$BNC.01	+2330	(-)	tgggagcctgTGTCccaaa	1.0	0.874
V$BPTF	V$FAC1.01	+3804	(+)	accaaAACAca	1.0	0.966
	V$FAC1.01	+5769	(-)	cacacAACAcc	1.0	0.984
	V$FAC1.01	+7019	(-)	agtacAACAaa	1.0	0.963
V$BRAC	V$EOMES.02	+275	(-)	ggtctcgtaaGGTGctgccaaccttcccc	1.0	0.880
	V$EOMES.02	+1131	(-)	ctgaggtccaGGTGtcctcattgcgcagc	1.0	0.887
	V$EOMES.02	+2008	(+)	aagtagaataGGTGtctcgctctgttaag	1.0	0.905
	V$EOMES.02	+2986	(+)	gcttactgtaGGTGtttacaaatgtggag	1.0	0.885
	V$EOMES.02	+3461	(-)	gaattagccaGGTGtggtggtgggcgcct	1.0	0.899

	V$EOMES.02	+4934	(-)	cttcaggccaGGTGtggtggctcacacct	1.0	0.899
	V$MGA.01	+3968	(+)	gcctaaataaggagtaaaaagACACgagc	1.0	0.802
	V$SPT.01	+4925	(+)	tgggattacagGTGTgagccaccacacct	1.0	1.000
	V$SPT.01	+7672	(-)	ctcctatctagGTGTgaagatgaataggc	1.0	1.000
V$BRN5	V$BRN5.01	+4330	(-)	tgttcaCATAccttatctatttt	1.0	0.810
	V$BRN5.01	+4555	(-)	catttcCATAatttaatatttat	1.0	0.836
	V$BRN5.01	+6239	(+)	atgaagCATAtgttaagccctag	1.0	0.804
	V$BRN5.01	+6295	(-)	aaaaacCATAaaatattacctta	1.0	0.807
	V$BRN5.01	+7316	(+)	ttatggCATAcgttagatgtttt	1.0	0.810
	V$BRN5.02	+4555	(+)	ataaatattaaATTAtggaaatg	1.0	0.827
	V$BRN5.03	+1689	(+)	aTAATgatcatttacttttctac	1.0	0.854
	V$BRN5.03	+2153	(-)	cTAATgagggttctatgtaaaaa	1.0	0.847
	V$BRN5.03	+2907	(-)	tTAATcggctgttaattggtagc	1.0	0.818
	V$BRN5.03	+3692	(-)	aTAATgacattttaaccaataaa	1.0	0.876
	V$BRN5.03	+7357	(+)	cTAATaatcacatcatggaaaat	1.0	0.802
	V$BRN5.03	+7493	(-)	aTAATgaatgaataaggccgggt	1.0	0.868
	V$BRN5.04	+1682	(-)	agtaaatgatcATTAttcaactt	1.0	0.842
	V$BRN5.04	+2160	(+)	atagaaccctcATTAgccagact	1.0	0.933
	V$BRN5.04	+2383	(+)	tggatcttctcATTAaattctga	1.0	0.940
	V$BRN5.04	+3699	(+)	gttaaaatgtcATTAtaattagc	1.0	0.849
	V$BRN5.04	+3706	(-)	gtcatttgctaATTAtaatgaca	1.0	0.916
	V$BRN5.04	+4310	(+)	caaagaatgtcATTAtaactaaa	1.0	0.848
	V$POU6F2.01	+3890	(+)	acaatgaagtaATTAacactttc	1.0	0.857
V$BRNF	V$BRN2.01	+1763	(+)	ctCATTctaaagatgatca	1.0	0.954
	V$BRN2.01	+7508	(+)	ttCATTataactatttttg	1.0	0.874
	V$BRN2.03	+4618	(+)	cacatcttaATTTgaatag	1.0	0.972
	V$BRN2.04	+3707	(+)	gtcattaTAATtagcaaat	1.0	0.869
	V$BRN2.04	+3708	(-)	catttgcTAATtataatga	1.0	0.861
	V$BRN2.04	+3892	(+)	aatgaagTAATtaacactt	1.0	0.917
	V$BRN2.04	+5209	(+)	gtatgtgTAATtaaaggaa	1.0	0.860
	V$BRN3.01	+2134	(-)	ggtacctaaATTAtacatg	1.0	0.806
	V$BRN3.01	+4557	(+)	aaatattaaATTAtggaaa	1.0	0.823
	V$BRN3.01	+7502	(+)	tattcattcATTAtaacta	1.0	0.891
	V$BRN3.02	+3893	(-)	aaagtgtTAATtacttcat	1.0	0.929
	V$BRN3.02	+5210	(-)	gttccttTAATtacacata	1.0	0.929
	V$BRN3.02	+7269	(+)	tttttttTAATtcttaatc	1.0	0.898
	V$BRN3.02	+7354	(+)	tgcctaaTAATcacatcat	1.0	0.913
	V$BRN3.02	+7800	(+)	tcagtagTAATcaatcact	1.0	0.904
	V$BRN3.03	+2386	(-)	cagaattTAATgagaagat	1.0	0.853
	V$BRN4.01	+1235	(-)	gcccaagtagtTAATtgta	1.0	0.920
	V$BRN4.01	+3276	(+)	tttgaggttaaTAATcttg	1.0	0.912
	V$BRN4.01	+3473	(+)	ccacacctggcTAATtctt	1.0	0.926
	V$BRN4.01	+3888	(+)	tcacaatgaagTAATtaac	1.0	0.906
	V$BRN4.01	+5957	(+)	tatgatataaaTAATagca	1.0	0.907
	V$BRN4.02	+1234	(+)	atacaaTTAActacttggg	1.0	0.847
	V$BRN4.02	+3873	(-)	gtgaaaTTAAttctctgac	1.0	0.882
	V$BRN4.02	+3876	(+)	agagaaTTAAtttcacaat	1.0	0.892
	V$TST1.01	+944	(+)	tctgaATTAtcgggtgctc	1.0	0.906
	V$TST1.01	+2920	(+)	agccgATTAaaatggtctg	1.0	0.913
	V$TST1.01	+3474	(-)	aaagaATTAgccaggtgtg	1.0	0.927
	V$TST1.01	+4615	(-)	ttcaaATTAagatgtgttt	1.0	0.941
	V$TST1.01	+5299	(+)	tctgaATTAaaaattttca	1.0	0.930
	V$TST1.01	+6743	(-)	cctgaATTAcctggacctt	1.0	0.920
V$BTBF	V$KAISO.01	+4190	(+)	aagcCTGCtac	1.0	0.923
	V$KAISO.01	+4283	(-)	tgtcCTGCcat	1.0	0.983
	V$KAISO.01	+5970	(-)	tataCTGCtat	1.0	0.926
V$CAAT	V$CAAT.01	+3689	(-)	ttaaCCAAtaaagtg	1.0	0.952
	V$NFY.02	+5454	(+)	aaagCCAAgcactac	1.0	0.833
	V$NFY.04	+510	(-)	aaccCCAAttagaga	1.0	0.925
	V$NFY.04	+2907	(+)	gctaCCAAttaacag	1.0	0.923
	V$NFY.04	+4009	(+)	tctgCCAAtgacaga	1.0	0.955
	V$NFY.04	+4751	(-)	accgCCAAtctagct	1.0	0.928
	V$NFY.04	+6611	(+)	ccagCCAAtatcttg	1.0	0.926
	V$NFY.04	+6645	(-)	ggatCCAAtaaatgt	1.0	0.932
V$CABL	V$CABL.01	+3256	(-)	aaAACAaaaaa	1.0	0.997
V$CARE	V$CARF.01	+4053	(-)	aaactGAGGct	1.0	0.956
	V$CARF.01	+5412	(+)	ggaagGAGGca	1.0	0.925
V$CART	V$ALX3.01	+3869	(-)	gaaattAATTctctgacttac	1.0	0.856

	V$CART1.01	+3698	(-)	aattaTAATgacattttaacc	1.0	0.892
	V$CART1.01	+4309	(-)	agttaTAATgacattctttgt	1.0	0.887
	V$CART1.01	+4620	(+)	catctTAATttgaatagagtt	1.0	0.884
	V$CART1.01	+7445	(-)	gtcaaTAATttcattgtacgt	1.0	0.923
	V$ESX1.01	+2907	(+)	gctaccAATTaacagccgatt	1.0	0.881
	V$ISX.01	+504	(-)	aaccccAATTagagaaactgg	1.0	0.971
	V$MIXL1.01	+7356	(+)	cctaaTAATcacatcatggaa	1.0	0.826
	V$PHOX2.01	+241	(-)	ctcgaTAATcaaaggcgactg	1.0	0.871
	V$PHOX2.01	+4554	(-)	ttccaTAATttaatatttatt	1.0	0.871
	V$PHOX2.01	+6102	(-)	atcccTAATctgaagagccaa	1.0	0.889
	V$PHOX2.01	+8017	(-)	agttcTAATccatctctaggc	1.0	0.876
	V$PHOX2.01	+8062	(+)	cagttTAATggaaattggtga	1.0	0.883
	V$PHOX2A.01	+3874	(+)	tcagagAATTaatttcacaat	1.0	0.844
	V$PROP1.01	+5292	(-)	atttttAATTcagaaaaagaa	1.0	0.860
	V$PROP1.01	+5297	(+)	tttctgAATTaaaaattttca	1.0	0.858
	V$RAX.01	+6471	(-)	tacaaaAATTagccgggtgtg	1.0	0.861
	V$S8.01	+509	(+)	ttctcTAATtggggttggagc	1.0	1.000
	V$S8.01	+1227	(-)	gtagtTAATtgtatcagtctc	1.0	0.995
	V$S8.01	+2902	(-)	gctgtTAATtggtagcttttc	1.0	1.000
	V$S8.01	+3704	(-)	tttgcTAATtataatgacatt	1.0	0.992
	V$S8.01	+3709	(+)	cattaTAATtagcaaatgact	1.0	0.997
	V$S8.01	+3889	(-)	agtgtTAATtacttcattgtg	1.0	0.992
	V$S8.01	+3894	(+)	tgaagTAATtaacactttcaa	1.0	0.997
	V$S8.01	+5206	(-)	tccttTAATtacacatacttc	1.0	0.992
	V$S8.01	+5211	(+)	atgtgTAATtaaaggaactaa	1.0	0.997
	V$XVENT2.01	+3878	(+)	agaatTAATttcacaatgaag	1.0	0.825
	V$XVENT2.01	+7353	(+)	atgccTAATaatcacatcatg	1.0	0.844
V$CDEF	V$CDE.01	121	(+)	ggggCGCGgtccg	1.0	0.882
V$CDXF	V$CDX2.02	+2786	(+)	agggacctTTATgagcaga	1.0	0.857
	V$CDX2.02	+7744	(-)	cctgctatTTATtcctcta	1.0	0.882
	V$CDX2.03	+1665	(-)	tttaccttTTATggaaaca	1.0	0.957
	V$CDX2.03	+3685	(+)	agtacactTTATtggttaa	1.0	0.984
	V$CDX2.03	+4697	(+)	tttggtttTTATtgaacca	1.0	0.992
	V$CDX2.03	+6299	(+)	gtaatattTTATggttttt	1.0	0.959
	V$CDX2.03	+6641	(+)	gtggacatTTATtggatcc	1.0	0.984
	V$CDX2.03	+7281	(+)	cttaatctTTATgggtaca	1.0	0.956
	V$CDX2.03	+7308	(+)	atatatatTTATggcatac	1.0	0.962
V$CEBP	V$CEBPB.01	+3293	(-)	gctcttttGAAAtca	1.0	0.946
	V$CEBPB.01	+4299	(-)	tttgtggtGAAAtct	1.0	0.949
	V$CEBPB.01	+6673	(-)	cacttttaGAAAtgg	1.0	0.953
	V$CEBPB.01	+7934	(+)	agggtttgGCAAtct	1.0	0.983
	V$CEBPB.02	+916	(-)	catgtTGTGaaagcc	1.0	0.996
	V$CEBPB.02	+3883	(-)	ttcatTGTGaaatta	1.0	0.998
	V$CEBPB.02	+5120	(+)	gctccTGTGcaatga	1.0	0.945
	V$CEBPB.02	+5207	(+)	aagtaTGTGtaatta	1.0	0.935
	V$CEBPB.02	+5877	(+)	cagccTGTGcaacag	1.0	0.947
	V$CEBPB.02	+7204	(-)	ggaaaTGTGaaataa	1.0	0.984
	V$CEBPB.02	+7731	(+)	aagtgTGTGcaaata	1.0	0.939
	V$CEBPE.02	+2635	(+)	tgtctttgGCAAatg	1.0	0.993
	V$CEBPE.02	+5916	(+)	aaagatgtGCAAaaa	1.0	0.973
	V$CEBPE.02	+6933	(+)	ccccttagGCAAacc	1.0	0.987
	V$CEBPG.01	+1129	(-)	ctcaTTGCgcagcaa	1.0	0.857
V$CHOP	V$CHOP.01	+7346	(+)	gtatGCAAtgcct	1.0	0.933
	V$CHOP.02	+3393	(+)	cacTGCAacctct	1.0	0.974
V$CHRF	V$CHR.01	+2483	(+)	tcttTTGAactgt	1.0	0.939
	V$CHR.01	+3293	(-)	tcttTTGAaatca	1.0	0.926
	V$CHR.01	+3772	(+)	gtgcTTGAatgaa	1.0	0.929
	V$CHR.01	+3929	(-)	cactTTGAaattt	1.0	0.933
	V$CHR.01	+4130	(-)	tggtTTGAagcat	1.0	0.933
	V$CHR.01	+4542	(+)	ggctTTGAaaaga	1.0	0.924
	V$CHR.01	+4625	(+)	taatTTGAataga	1.0	0.961
	V$CHR.01	+6188	(-)	ttatTTGAaatag	1.0	0.955
	V$CHR.01	+6625	(+)	gcatTTGAatcaa	1.0	0.979
	V$CHR.01	+7154	(+)	gggtTTGAattcc	1.0	0.993
	V$CHR.01	+7436	(-)	acgtTTGAaaata	1.0	0.929
V$CIZF	V$NMP4.01	+1267	(+)	agAAAAagctt	1.0	0.973
	V$NMP4.01	+3059	(-)	ggAAAAaaaaa	1.0	0.978
	V$NMP4.01	+3328	(-)	agAAAAagaaa	1.0	0.970

	V$NMP4.01	+5291	(-)	agAAAAagaaa	1.0	0.970
	V$NMP4.01	+6974	(-)	ggAAAAaaata	1.0	0.976
V$CLOX	V$CDP.02	+2905	(+)	aagctacCAATtaacagccgatt	1.0	0.958
	V$CDP.02	+3683	(-)	ttttaacCAATaaagtgtacttt	1.0	0.960
	V$CDP.02	+4007	(+)	gatctgcCAATgacagatgactt	1.0	0.956
	V$CDP.02	+4498	(-)	tattttcCAATctaaccaaatcc	1.0	0.952
	V$CDP.02	+4787	(-)	acattgcCAATatcccctgggag	1.0	0.944
	V$CDP.02	+6639	(-)	agggatcCAATaaatgtccacta	1.0	0.956
	V$CPHX.01	+1230	(+)	acTGATacaattaactacttggg	1.0	0.848
	V$CPHX.01	+3767	(-)	ggTGATtcattcaagcacaaaat	1.0	0.879
	V$CPHX.01	+4175	(+)	ttTGATctgataaccaagcctgc	1.0	0.870
	V$CPHX.01	+6364	(-)	tgTGATggcaccactgcactcca	1.0	0.841
	V$CPHX.01	+7351	(-)	caTGATgtgattattaggcattg	1.0	0.858
V$CP2F	V$CP2.01	+2847	(+)	tgCTGGgtgggcctagaat	1.0	0.902
	V$TCFCP2L1.01	+6730	(+)	ttCTGGttgtttcaaggtc	1.0	0.894
V$CREB	V$ATF1.01	+6980	(+)	tttccttcACGTcacgttgcc	1.0	0.859
	V$ATF1.02	+409	(+)	atccggTGACgagccgcagtc	1.0	0.936
	V$ATF1.02	+522	(-)	gaagggTGACggggctccaac	1.0	0.935
	V$ATF1.02	+2761	(+)	cttctgTGACggttgcgcaca	1.0	0.930
	V$ATF1.02	+7899	(+)	ctagtgTGACggaagtttaaa	1.0	0.949
	V$ATF2.01	+1269	(-)	ggaagcTGACgaaagcttttt	1.0	0.930
	V$ATF6.02	+6979	(-)	gcaacgtGACGtgaaggaaaa	1.0	1.000
	V$CREB.02	+406	(+)	cccatccggTGACgagccgca	1.0	0.940
	V$CREB.02	+525	(-)	ggggaagggTGACggggctcc	1.0	0.936
	V$CREB.02	+1272	(-)	tttggaagcTGACgaaagctt	1.0	0.906
	V$CREB.02	+3649	(+)	gttggaagtTGACggggcata	1.0	0.898
	V$CREB.02	+6982	(-)	caggcaacgTGACgtgaagga	1.0	0.911
	V$CREB1.02	+3541	(-)	ggatcATGAggttagggtttt	1.0	0.936
	V$CREB1.02	+3888	(+)	tcacaATGAagtaattaacac	1.0	0.937
	V$CREB1.02	+6335	(+)	ttgagATGAggtcgctctgtc	1.0	0.924
	V$CREB1.02	+7357	(-)	tttccATGAtgtgattattag	1.0	0.943
	V$CREB3L2.01	+3652	(+)	ggaagttGACGgggcataccc	1.0	0.867
	V$E4BP4.01	+1298	(-)	aaaaccttagGTAAacaaatc	1.0	0.901
	V$E4BP4.01	+1936	(-)	tgcttcttacGTAActtagtg	1.0	0.948
	V$E4BP4.01	+1937	(+)	actaagttacGTAAgaagcag	1.0	0.956
	V$E4BP4.01	+2149	(-)	agggttctatGTAAaaaggta	1.0	0.830
	V$E4BP4.01	+2703	(-)	tgctcctcaaGTAAcacaaga	1.0	0.826
	V$E4BP4.01	+5324	(+)	ttccttttctGTAAcaactct	1.0	0.833
	V$E4BP4.01	+5469	(+)	aggatgtaacGTAAatgaaag	1.0	0.841
	V$E4BP4.01	+6289	(+)	caaaaataagGTAAtatttta	1.0	0.830
	V$JUNDM2.01	+5156	(-)	cagcaataACGTtatcaacat	1.0	0.817
	V$JUNDM2.01	+5157	(+)	tgttgataACGTtattgctgg	1.0	0.813
	V$XBP1.01	+300	(-)	ccccggggACGTgtgtgggtc	1.0	0.914
V$CSEN	V$DREAM.01	+2781	(+)	agGTCAgggac	1.0	0.992
	V$DREAM.01	+5092	(+)	gtGTCAgtggt	1.0	0.950
	V$DREAM.01	+6396	(-)	agGTCAaggct	1.0	0.960
	V$DREAM.01	+6881	(-)	atGTCAaggct	1.0	0.965
V$CTCF	V$CTCF.01	+107	(+)	cccgggcgctgcttGGGGcgcggtccg	1.0	0.837
	V$CTCF.01	+261	(+)	gggcgctggcgttcGGGGaaggttggc	1.0	0.804
	V$CTCF.01	+449	(+)	aggtcccgctgcgaGGGGaggcggagg	1.0	0.857
V$DLXF	V$DLX1.01	+2131	(+)	tctcatgtatAATTaggt	1.0	0.910
	V$DLX1.01	+3705	(+)	atgtcattatAATTagcaa	1.0	0.988
	V$DLX1.01	+5207	(+)	aagtatgtgtAATTaaagg	1.0	0.988
	V$DLX1.01	+5212	(-)	tagttcctttAATTacaca	1.0	0.982
	V$DLX1.02	+3870	(+)	taagtcagagAATTaattt	1.0	0.881
	V$DLX1.02	+3890	(+)	acaatgaagtAATTaacac	1.0	0.994
	V$DLX1.02	+3895	(-)	tgaaagtgttAATTacttc	1.0	0.988
	V$DLX2.01	+1228	(+)	agactgatacAATTaacta	1.0	0.920
	V$DLX2.01	+3710	(-)	gtcatttgctAATTataat	1.0	0.985
	V$DLX3.01	+505	(+)	cagtttctcTAATtggggt	1.0	0.932
	V$DLX3.01	+1233	(-)	ccaagtagtTAATtgtatc	1.0	0.950
	V$DLX3.01	+2908	(-)	atcggctgtTAATtggtag	1.0	0.956
	V$DLX3.01	+3874	(+)	tcagagaatTAATttcaca	1.0	0.916
	V$DLX3.01	+7451	(-)	tatagtcaaTAATttcatt	1.0	0.924
	V$DLX3.02	+1467	(+)	atgcccggcTAATtttgta	1.0	0.936
	V$DLX4.01	+4560	(-)	gcatttccatAATTtaata	1.0	0.885
	V$DLX5.01	+2903	(+)	aaaagctaccAATTaacag	1.0	0.921
V$DMRT	V$DMRT2.01	+1226	(+)	tgagactgaTACAattaacta	1.0	0.870

	V$DMRT3.01	+5325	(-)	aagagttgtTACAgaaaagga	1.0	0.849
	V$DMRT3.01	+7287	(+)	ctttatgggTACAtagcaggt	1.0	0.839
	V$DMRT7.01	+1224	(-)	gttaATTGtatcagtctcatt	1.0	0.820
V$DMTF	V$DMP1.02	+403	(-)	tcaccGGATggggct	1.0	0.976
V$DUXF	V$DUX4.01	+1295	(+)	tctGATTtgtttacc	1.0	0.889
	V$DUX4.01	+4496	(+)	ctgGATTtggttaga	1.0	0.827
V$E2FF	V$E2F.01	+1982	(+)	attgaccccGAAAacta	1.0	0.816
	V$E2F.02	+1334	(-)	aacaagagcGAAActgc	1.0	0.849
	V$E2F.02	+6097	(-)	ctgaagagcCAAAatcc	1.0	0.849
	V$E2F.02	+6872	(-)	tcaaggctcCAAAagca	1.0	0.849
	V$E2F.03	+119	(+)	ttgggGCGCggtccgga	1.0	0.876
	V$E2F1_DP2.01	+351	(-)	cgacGGCGgcgaagccc	1.0	0.830
	V$E2F2.01	+116	(-)	ggaccGCGCcccaagca	1.0	0.854
	V$E2F2.01	+160	(-)	tccgcGCGCgccccggc	1.0	0.905
	V$E2F2.01	+162	(-)	tctccGCGCgcgccccg	1.0	0.901
	V$E2F2.01	+163	(+)	ggggcGCGCgcggagag	1.0	0.899
	V$E2F2.01	+5058	(+)	ggacaGCGCcccacagc	1.0	0.851
	V$E2F3.01	+99	(+)	agaggGCGCccgggcgc	1.0	0.897
	V$E2F3.01	+161	(+)	ccgggGCGCgcgcggag	1.0	0.931
	V$E2F3.02	+588	(+)	ataggGCGCctcccggg	1.0	0.872
	V$E2F3.02	+3455	(-)	ggtggGCGCctgtaatc	1.0	0.869
	V$E2F3.02	+7982	(-)	gtgtgGCGCcacaaaaa	1.0	0.961
	V$E2F3.02	+7985	(+)	ttgtgGCGCcacactac	1.0	0.956
	V$E2F4.01	+212	(+)	gggcgGCGGgcagcgat	1.0	0.972
	V$E2F4.01	+4895	(-)	ctgagGCGGgaatattg	1.0	0.986
	V$E2F6.01	+446	(-)	tcgcaGCGGgacctggt	1.0	0.906
	V$E2F7.02	+2506	(+)	cgtagggGGGAagatca	1.0	0.880
V$E4FF	V$E4F.01	+1939	(-)	cttACGTaactta	1.0	0.881
	V$E4F.01	+5471	(-)	tttACGTtacatc	1.0	0.864
	V$E4F.01	+5474	(+)	gtaACGTaaatga	1.0	0.825
	V$E4F.01	+6982	(-)	gtgACGTgaagga	1.0	0.910
	V$E4F.01	+6985	(+)	ttcACGTcacgtt	1.0	0.881
V$EBOX	V$CMYC.02	+341	(+)	ggagcctCGTGggcttc	1.0	0.941
	V$MNT.01	+4709	(+)	tgaacCACGtgtgtaca	1.0	0.991
	V$USF.01	+4708	(-)	gtacaCACGtggttcaa	1.0	0.994
	V$USF.01	+5727	(-)	ttagaCACGgggtctca	1.0	0.874
	V$USF.03	+318	(-)	ggtggccCGTGccgccc	1.0	0.897
	V$USF.03	+5728	(+)	gagacccCGTGtctaaa	1.0	0.898
	V$USF1.01	+5681	(+)	aggaTCACttgaaccca	1.0	0.956
	V$USF1.02	+1553	(-)	cgggTCACctgaggtcg	1.0	0.906
	V$USF1.02	+5815	(+)	aggaTCACatgagcctg	1.0	0.840
	V$USF1.02	+6987	(+)	cacgTCACgttgcctga	1.0	0.846
V$EGRF	V$CKROX.01	+7538	(-)	gggaGGGAggtggggatag	1.0	0.901
	V$CKROX.01	+7550	(-)	gtgaGGGAgggtgggaggg	1.0	0.953
	V$EGR1.01	+354	(-)	aggtgcgacGGCGgcgaag	1.0	0.850
	V$EGR1.03	+553	(+)	ggccgcgGGGGagcgaagg	1.0	0.864
	V$EGR1.03	+1502	(+)	tggtgggGGGGggggggtc	1.0	0.901
	V$EGR1.03	+1504	(+)	gtgggggGGGGggggtccc	1.0	0.878
	V$EGR1.04	+207	(+)	gagcggggcGGCGggcagc	1.0	0.872
	V$EGR1.04	+371	(-)	cagagcgcaGGCGgcggag	1.0	0.873
	V$EGR1.04	+459	(+)	gcgaggggaGGCGgagggg	1.0	0.907
	V$EGR2.02	+5674	(+)	aggTGGGaggatcacttga	1.0	0.936
	V$EGR2.02	+5808	(+)	aggTGGGaggatcacatga	1.0	0.936
	V$EGR2.02	+6414	(-)	aggTGGGaggatcatttga	1.0	0.936
	V$EGR2.02	+7542	(-)	gggTGGGagggaggtgggg	1.0	0.981
	V$EGR3.01	+1600	(+)	atagGCGTgggccactgcg	1.0	0.820
	V$WT1.01	+1500	(+)	ggtggTGGGgggggggggg	1.0	0.948
	V$WT1.01	+7544	(-)	gagggTGGGagggaggtgg	1.0	0.948
V$EREF	V$ER.01	+5503	(+)	tggaGTCAccgtgacttcc	1.0	0.852
	V$ER.03	+1140	(+)	tgaggacacctgGACCtca	1.0	0.878
	V$ER.03	+6341	(-)	gggtgacagagcGACCtca	1.0	0.827
	V$ER.04	+1528	(+)	gttgGTCAggctggtctcg	1.0	0.898
	V$ER.04	+5589	(-)	catgGTCAttctgaaacct	1.0	0.873
	V$ER.04	+7644	(-)	catgGTCAaaggggccaca	1.0	0.873
V$ESRR	V$ERR.01	+2670	(+)	ctgtgtttcagcAAGGacaggaa	1.0	0.897
	V$ESRRA.01	+5339	(+)	aactcttctctcAAGGtccccct	1.0	0.898
	V$ESRRA.01	+6731	(+)	tctggttgtttcAAGGtccaggt	1.0	0.902
	V$ESRRA.02	+1980	(-)	cagctagttttcggGGTCaatcc	1.0	0.934

	V$ESRRA.03	+4397	(-)	gacacattagacAAGGccactag	1.0	0.918
	V$ESRRB.01	+2238	(-)	cacttggcatttaAGGTcaacac	1.0	0.939
V$ETSF	V$ELF5.01	+1647	(+)	tcatactaGGAAattgtgtgt	1.0	0.890
	V$ELF5.01	+2440	(-)	agcgaactGGAAgtatacagc	1.0	0.933
	V$ELF5.01	+5509	(-)	cttgacagGGAAgtcacggtg	1.0	0.928
	V$ELK1.02	+7901	(+)	agtgtgacGGAAgtttaaact	1.0	0.915
	V$ERG.02	+2309	(-)	cgatcacaGGAAagaagagct	1.0	0.956
	V$ERG.02	+4817	(-)	caaaaaaaGGAAaacaaaatg	1.0	0.966
	V$ERG.02	+5315	(-)	acagaaaaGGAAaataaatga	1.0	0.950
	V$ERG.02	+6972	(-)	gacgtgaaGGAAaaaaatatt	1.0	0.937
	V$ERG.02	+7116	(-)	gaaggaaaGGAAaggtgctgt	1.0	0.957
	V$ERG.02	+7121	(-)	tctaagaaGGAAaggaaaggt	1.0	0.964
	V$ERG.02	+7783	(-)	ctgagacaGGAAagacaatga	1.0	0.976
	V$ETS1.01	+854	(+)	tgtgcccaGGAAatggtgggt	1.0	0.946
	V$ETV1.02	+2681	(+)	caaggacaGGAAgtagactgc	1.0	0.998
	V$ETV1.02	+2741	(+)	ggggctcaGGAAgtagtcttc	1.0	0.989
	V$ETV4.01	+400	(-)	tcgtcacCGGAtggggcttac	1.0	0.896
	V$PDEF.01	+5034	(-)	gcactgcaGGATgttcagcag	1.0	0.974
	V$PDEF.01	+5462	(+)	gcactacaGGATgtaacgtaa	1.0	0.953
	V$PEA3.01	+1808	(-)	tgaatttAGGAagttgcttaa	1.0	0.953
	V$SPI1.02	+531	(-)	tgatctggGGAAgggtgacgg	1.0	0.963
	V$SPI1.02	+1179	(+)	gattctggGGAAgaaatggga	1.0	0.962
	V$SPI1.02	+2594	(-)	gaataaggGGAActggtcctt	1.0	0.980
	V$SPI1.02	+5404	(+)	ccttttggGGAAggaggcaca	1.0	0.964
	V$SPI1.04	+7740	(+)	caaatagaGGAAtaaatagca	1.0	0.898
	V$SPIB.01	+2950	(-)	cagaataaGGAAtttaaacat	1.0	0.896
	V$SPIC.01	+4455	(+)	gaaacagtGGAAatgcaacga	1.0	0.834
V$EVI1	V$EVI1.02	+1261	(+)	ctgagAAGAaaaagctt	1.0	0.885
	V$EVI1.02	+3194	(+)	ctgaaAAGAaaaaagaa	1.0	0.885
	V$EVI1.02	+3317	(-)	aagaaAAGAaaagcatt	1.0	0.899
	V$EVI1.05	+4176	(+)	ttgatctGATAaccaag	1.0	0.819
	V$EVI1.05	+5853	(-)	gtgacatGATAtcaggg	1.0	0.865
	V$EVI1.06	+4293	(+)	agtacaAGATttcacca	1.0	0.854
	V$EVI1.07	+1767	(+)	ttctaAAGAtgatcata	1.0	0.977
	V$EVI1.07	+2307	(-)	caggaAAGAagagctat	1.0	0.903
	V$EVI1.07	+4614	(-)	aaattAAGAtgtgttta	1.0	0.917
	V$EVI1.07	+5912	(+)	aaaaaAAGAtgtgcaaa	1.0	0.912
	V$EVI1.07	+6859	(-)	agcacAAGAtgcttcac	1.0	0.911
	V$EVI1.07	+6953	(-)	ggtctAAGAtgctacca	1.0	0.900
	V$MEL1.02	+2566	(+)	gagctatGATGagaaag	1.0	0.995
	V$MEL1.02	+3185	(-)	cttttcaGATGagtagt	1.0	0.998
	V$MEL1.02	+3857	(+)	agttacaGATGagtaag	1.0	0.998
	V$MEL1.02	+5980	(+)	agtaaagGATGagcatc	1.0	0.993
	V$MEL1.02	+6332	(+)	tttttgaGATGaggtcg	1.0	0.994
	V$MEL1.02	+7225	(-)	caaggcaGATGagatgc	1.0	0.992
	V$MEL1.03	+1883	(+)	gtttgatGATGgtcctt	1.0	0.984
	V$MEL1.03	+7673	(-)	gtgtgaaGATGaatagg	1.0	0.973
V$FKHD	V$FHXB.01	+1822	(+)	aaattcATAAtatttag	1.0	0.931
	V$FHXB.01	+3309	(+)	tcagttATAAtgctttt	1.0	0.843
	V$FHXB.01	+6300	(-)	aaaaccATAAaatatta	1.0	0.870
	V$FHXB.01	+8096	(+)	aaaatgATAAtctttgt	1.0	0.836
	V$FOXA1.01	+1660	(-)	tttatggaAACAcacaa	1.0	0.959
	V$FOXJ1.01	+4608	(+)	agcctttaAACAcatct	1.0	0.915
	V$FOXO1.01	+2234	(-)	ttaaggtcAACAcgaga	1.0	0.900
	V$FOXO1.01	+4341	(+)	ggtatgtgAACAtgctt	1.0	0.887
	V$FOXP1.01	+1297	(-)	cttaggtAAACaaatca	1.0	0.996
	V$FOXP1.01	+2993	(-)	catttgtAAACacctac	1.0	0.990
	V$FOXP1.01	+3094	(+)	atcctggAAACaccagg	1.0	0.943
	V$FOXP1.01	+3827	(-)	aaattgtAAACaaattt	1.0	0.997
	V$FOXP1.01	+4480	(-)	gtttgggAAACaaagca	1.0	0.945
	V$FOXP1.02	+1705	(+)	tttctacAACAatagtc	1.0	1.000
	V$FOXP1.02	+7015	(-)	gcagtacAACAaactaa	1.0	1.000
	V$FOXP1_ES.01	+3255	(-)	tcaggaaAACAaaaaag	1.0	1.000
	V$FOXP1_ES.01	+3802	(+)	tgaccaaAACAcaatcc	1.0	1.000
	V$FOXP1_ES.01	+3939	(-)	aatagaaAACAtcacac	1.0	1.000
	V$FOXP1_ES.01	+4163	(-)	tcaaaaaAACAgtatat	1.0	1.000
	V$FOXP1_ES.01	+4816	(-)	aaaggaaAACAaaatgt	1.0	1.000
	V$FOXP1_ES.01	+7327	(-)	gtgtcaaAACAtctaac	1.0	1.000

	V$FOXP1_ES.01	+7619	(-)	aactcaaAACAattgag	1.0	1.000
	V$FOXP1_ES.01	+7974	(-)	ccacaaaAACAtctctt	1.0	1.000
	V$FREAC2.01	+2148	(-)	tctatgTAAAaaggtac	1.0	0.841
	V$FREAC3.01	+1692	(-)	gaaaaGTAAatgatcat	1.0	0.842
	V$FREAC3.01	+3976	(+)	aaggaGTAAaaagacac	1.0	0.864
	V$FREAC7.01	+5958	(+)	atgataTAAAtaatagc	1.0	0.967
	V$HFH1.01	+3035	(-)	aaaaaaTAAActaaaag	1.0	0.872
	V$HFH1.01	+4513	(+)	ggaaaaTAAActaaaca	1.0	0.872
	V$HNF3B.01	+6969	(-)	aggaaaaaAATAtttgg	1.0	0.950
	V$HNF3B.02	+4518	(+)	ataaacTAAAcatgctt	1.0	0.915
	V$HNF3B.02	+6688	(+)	attgagTAAAtatcaca	1.0	0.941
	V$XFD2.01	+1633	(+)	agtttaTAAAcatttca	1.0	0.987
	V$XFD2.01	+1840	(-)	tgatttTAAAcatcacc	1.0	0.908
	V$XFD2.01	+2946	(-)	ggaattTAAAcatgagt	1.0	0.908
	V$XFD2.01	+4550	(+)	aaagaaTAAAtattaaa	1.0	0.897
V$FXRE	V$FXRE.01	+2781	(-)	AGGTccctgacct	1.0	0.923
	V$FXRE.01	+2781	(+)	AGGTcagggacct	1.0	0.862
	V$FXRE.01	+3280	(+)	AGGTtaataatct	1.0	0.875
	V$FXRE.01	+7780	(+)	AGGTcattgtctt	1.0	0.829
V$GATA	V$GATA.01	+1229	(+)	gactGATAcaatt	1.0	0.933
	V$GATA.01	+3235	(-)	ttcaGATAagtat	1.0	0.992
	V$GATA1.01	+7689	(+)	cctaGATAggagc	1.0	0.976
	V$GATA1.04	+946	(-)	acccGATAattca	1.0	0.912
	V$GATA1.04	+4179	(+)	atctGATAaccaa	1.0	0.967
	V$GATA1.04	+8089	(+)	ttagGATAaaatg	1.0	0.916
	V$GATA1.05	+250	(-)	cctcGATAatcaa	1.0	0.882
	V$GATA1.06	+1370	(-)	ccgaGATAgtgcc	1.0	0.968
	V$GATA1.06	+4232	(+)	tgtaGATAggctg	1.0	0.962
	V$GATA1.06	+4332	(+)	aataGATAaggta	1.0	0.990
	V$GATA1.06	+5157	(+)	tgttGATAacgtt	1.0	0.962
	V$GATA2.01	+8097	(+)	aaatGATAatctt	1.0	0.922
	V$GATA2.03	+7817	(-)	tgaaGATAagcag	1.0	0.986
	V$GATA3.01	+4038	(-)	gagaGATAacatg	1.0	0.994
	V$GATA3.02	+4173	(-)	atcAGATcaaaaa	1.0	0.918
V$GCF2	V$LRRFIP1.01	+60	(-)	cgtCCCCcgcgccccgcgc	1.0	0.859
	V$LRRFIP1.01	+225	(-)	ctgCCCCgggcccagatcg	1.0	0.864
	V$LRRFIP1.01	+433	(+)	tcaCCCCagtcccaccagg	1.0	0.825
	V$LRRFIP1.01	+548	(-)	gctCCCCcgcggccgtgat	1.0	0.846
V$GCMF	V$GCM1.01	+2161	(+)	tagaaCCCTcattag	1.0	0.908
	V$GCM1.01	+5277	(-)	agtctCCCTcatttt	1.0	0.895
	V$GCM1.02	+1074	(-)	tggaaCCCGcatggc	1.0	0.995
	V$GCM1.03	+2504	(-)	cttccCCCCtacgga	1.0	0.886
	V$GCM1.03	+2735	(-)	ctgagCCCCtaggca	1.0	0.856
	V$GCM1.03	+5991	(+)	agcatCCCCaatgca	1.0	0.876
	V$GCM1.03	+7837	(-)	cccatCCCCtactct	1.0	0.876
V$GCNR	V$RTR.01	+4868	(-)	caggagtTCAAgaccagcc	1.0	0.824
V$GFI1	V$GFI1.01	+2581	(+)	agaAATCctaggaaa	1.0	0.963
	V$GFI1.02	+1290	(-)	acaAATCagaacttt	1.0	0.929
	V$GFI1.02	+4772	(-)	caaAATCatggctag	1.0	0.945
	V$GFI1.02	+6710	(+)	gcaAATCaaagagaa	1.0	0.934
	V$GFI1.02	+7809	(+)	atcAATCactgctta	1.0	0.943
	V$GFI1B.01	+1849	(+)	taaAATCagcactta	1.0	0.902
V$GLIF	V$GLI1.01	+5824	(-)	tccacctCCCAggctca	1.0	0.908
	V$GLI3.02	+767	(-)	atgcCCACtcaggccat	1.0	0.896
	V$GLI3.02	+2844	(-)	aggcCCACccagcaatc	1.0	0.966
	V$GLIS2.01	+1506	(-)	gggaCCCCccccccccc	1.0	0.981
	V$GLIS2.01	+4116	(+)	aagaCCCCctgtggatg	1.0	0.925
	V$GLIS3.01	+1499	(-)	ccccCCCCccaccacca	1.0	0.901
	V$ZIC2.01	+231	(-)	gcgactgCCCCgggccc	1.0	0.892
	V$ZIC2.01	+6926	(+)	cagaccaCCCCttaggc	1.0	1.000
	V$ZIC3.02	+904	(-)	aagccCCCCggtagggc	1.0	0.808
	V$ZIC3.02	+5352	(+)	aggtcCCCCtgcagtag	1.0	0.815
V$GREF	V$GRE.01	+2467	(+)	caagtgtcatgtGTTCtct	1.0	0.881
	V$GRE.01	+4072	(-)	ggagactactgtGTTCtca	1.0	0.882
	V$PRE.01	+4382	(+)	atgccaggtttTGTTctag	1.0	0.902
	V$PRE.01	+5888	(-)	agacagggtctTGTTctgt	1.0	0.843
V$GRHL	V$GRHL1.01	+6207	(-)	tatacaGGTTgag	1.0	0.885
	V$GRHL2.01	+1399	(-)	tgAACCcggtagg	1.0	0.883

	V$GRHL3.01	+2417	(-)	tagactGGTTctt	1.0	0.887
V$HAML	V$AML1.02	4939	(-)	aggtGTGGtggctca	1.0	0.960
	V$AML1.02	4984	(+)	gagtGTGGtatgctg	1.0	0.969
	V$AML1.02	5763	(+)	gggtGTGGtgttgtg	1.0	0.971
	V$AML1.02	6463	(-)	gggtGTGGtggcgca	1.0	0.960
	V$AML1.02	6598	(-)	gggtGTGGtggctca	1.0	0.960
	V$AML2.01	2284	(+)	actTGTGgtaactga	1.0	0.944
	V$AML2.01	4300	(-)	cttTGTGgtgaaatc	1.0	0.955
	V$AML3.01	5570	(-)	agaaGTGGttaggtt	1.0	0.856
V$HAND	V$HAND2_E12.01	+3375	(-)	gtgagccaagaTGGCaccact	1.0	0.807
	V$HEN1.01	+633	(-)	ccagcctCAGCtgatgctcaa	1.0	0.843
	V$HEN1.01	+696	(-)	ccggccgCAGCtggtcccggt	1.0	0.922
	V$HEN1.01	+697	(+)	ccgggacCAGCtgcggccggg	1.0	0.922
	V$HEN1.01	+6808	(-)	aggagggCAGCtgggcccctg	1.0	0.863
	V$HEN1.01	+6809	(+)	aggggccCAGCtgccctcctc	1.0	0.907
	V$HEN1.02	+634	(+)	tgagcatcaGCTGaggctgga	1.0	0.829
	V$LYL1_E12.01	+3299	(-)	attataactGATGctcttttg	1.0	0.836
	V$LYL1_E12.01	+3855	(+)	gtagttacaGATGagtaagtc	1.0	0.831
	V$LYL1_E12.01	+7969	(+)	ccttgaagaGATGtttttgtg	1.0	0.878
	V$PARAXIS.01	+2466	(-)	aagagAACAcatgacacttgg	1.0	0.915
	V$PARAXIS.01	+6237	(-)	ggcttAACAtatgcttcattt	1.0	0.919
	V$SCX.01	+5413	(-)	tcctctgcatgTGCCtccttc	1.0	0.912
	V$TAL1_E2A.01	+3468	(-)	aattagcCAGGtgtggtggtg	1.0	0.986
	V$TAL1_E2A.01	+4941	(-)	ttcaggcCAGGtgtggtggct	1.0	0.986
	V$TAL1_E2A.02	+7468	(-)	gacagcaCAGCagggtgacta	1.0	0.991
	V$TAL1ALPHAE47.01	+7183	(-)	caagtcaCAGAtgctatgagt	1.0	0.919
	V$TAL1BETAE47.01	+5071	(-)	ttcaggtCAGAtgctttgctg	1.0	0.888
	V$TAL1BETAITF2.01	+4013	(+)	ccaatgaCAGAtgactttggg	1.0	0.868
	V$TH1E47.01	+2167	(+)	cctcattagCCAGactctaaa	1.0	0.948
V$HASF	V$HAS.01	+305	(+)	acaCACGtccc	1.0	0.976
V$HBOX	V$EN1.01	+8057	(+)	aggttcagTTTAatggaaa	1.0	0.806
	V$EN1.02	+3893	(+)	atgaagtAATTaacacttt	1.0	0.962
	V$EN2.01	+1230	(-)	agtagttAATTgtatcagt	1.0	0.864
	V$EVX1.01	+2163	(+)	gaaccctcATTAgccagac	1.0	0.864
	V$EVX2.01	+6475	(+)	cccggcTAATttttgtatt	1.0	0.810
	V$GBX1.01	+507	(-)	caaccccAATTagagaaac	1.0	0.908
	V$GSH1.01	+1466	(+)	catgcccggcTAATtttgt	1.0	0.873
	V$GSH1.01	+3277	(+)	ttgaggttaaTAATcttga	1.0	0.874
	V$GSH1.01	+3759	(-)	aagcacaaaaTAATattga	1.0	0.930
	V$GSH2.01	+508	(+)	tttctcTAATtggggttgg	1.0	1.000
	V$GSH2.01	+2162	(-)	tctggcTAATgagggttct	1.0	0.969
	V$GSH2.01	+2905	(-)	ggctgtTAATtggtagctt	1.0	0.977
	V$GSH2.01	+3708	(+)	tcattaTAATtagcaaatg	1.0	0.955
	V$GSH2.01	+4405	(+)	cttgtcTAATgtgtcgaga	1.0	0.962
	V$GSH2.01	+4619	(+)	acatctTAATttgaataga	1.0	0.964
	V$GSH2.01	+7524	(-)	gatagtTAATgggtgacaa	1.0	0.953
	V$GSH2.02	+3707	(-)	atttgctAATTataatgac	1.0	0.959
	V$GSH2.02	+3892	(-)	aagtgttAATTacttcatt	1.0	0.956
	V$MEOX1.01	+1231	(+)	ctgatacAATTaactactt	1.0	0.887
	V$VAX1.01	+5210	(+)	tatgtgtAATTaaaggaac	1.0	0.941
	V$VAX2.01	+2906	(+)	agctaccaATTAacagccg	1.0	0.881
	V$VAX2.01	+4618	(-)	ctattcaaATTAagatgtg	1.0	0.851
	V$VAX2.01	+5209	(-)	ttcctttaATTAcacatac	1.0	0.927
V$HDBP	V$HDBP1_2.01	+75	(-)	gcccggCCGGagccgcgtc	1.0	0.841
	V$HDBP1_2.01	+79	(-)	ccctgcCCGGccggagccg	1.0	0.844
	V$HDBP1_2.01	+152	(-)	cgcgccCCGGcccggtccc	1.0	0.867
V$HEAT	V$HSF1.03	+6721	(-)	cttgaaacaaccAGAAtatccttct	1.0	0.853
	V$HSF2.01	+132	(-)	gtcccggggagccGAACcctctccg	1.0	0.886
	V$HSF2.01	+3071	(-)	atcagagaattttGAACagtcagag	1.0	0.888
	V$HSF2.01	+4125	(-)	ctaaccatggtttGAAGcatccaca	1.0	0.894
	V$HSF2.02	+1187	(+)	ggaagaaatgggAGAAggctcagtt	1.0	0.954
	V$HSF2.02	+2150	(+)	accttttacatAGAAccctcatta	1.0	0.959
	V$HSF2.02	+3625	(+)	gcttttcaaaaaAGAAatctcatgg	1.0	0.956
V$HESF	V$DEC1.01	+6983	(+)	ccttCACGtcacgtt	1.0	0.867
	V$DEC1.02	+4709	(-)	tacacaCGTGgttca	1.0	0.927
	V$DEC2.01	+4710	(+)	gaaccaCGTGtgtac	1.0	0.978
	V$HELT.01	+320	(+)	gcggCACGggccacc	1.0	0.955
	V$HELT.01	+3986	(+)	aagaCACGagcccta	1.0	0.919

	V$HES7.01	+319	(-)	gtggccCGTGccgcc	1.0	0.899
V$HICF	V$HIC1.01	+279	(-)	tgcTGCCaacctt	1.0	0.937
	V$HIC1.01	+3377	(+)	tggTGCCatcttg	1.0	0.881
	V$HIC1.02	+853	(+)	ctgTGCCcaggaa	1.0	0.953
	V$HIC1.02	+1245	(-)	ctgTGCCcaagta	1.0	0.989
V$HIFF	V$ARNTL.01	6982	(-)	caacgtgaCGTGaagga	1.0	0.945
	V$HIF1.01	302	(+)	cccacacACGTccccgg	1.0	0.881
	V$HIF1.01	7318	(+)	atggcatACGTtagatg	1.0	0.873
	V$HIF1.02	4709	(-)	tgtacacaCGTGgttca	1.0	0.991
	V$HRE.02	303	(-)	cccggggaCGTGtgtgg	1.0	0.987
	V$HRE.02	4708	(+)	ttgaaccaCGTGtgtac	1.0	0.972
	V$HRE.03	6987	(-)	tcaggcaACGTgacgtg	1.0	0.938
V$HMTB	V$MTBF.01	+827	(-)	gggcATTTg	1.0	0.901
	V$MTBF.01	+2248	(-)	tggcATTTa	1.0	0.902
	V$MTBF.01	+2955	(-)	aggaATTTa	1.0	0.912
	V$MTBF.01	+4379	(-)	tggcATTTg	1.0	0.922
	V$MTBF.01	+6694	(-)	tgatATTTa	1.0	0.902
	V$MTBF.01	+7488	(-)	gggtATTTg	1.0	0.979
	V$MTBF.01	+7752	(-)	tgctATTTa	1.0	0.933
V$HNF1	V$HMBOX.01	+3850	(+)	gttatgtaGTTAcagat	1.0	0.841
	V$HMBOX.01	+4320	(-)	ctattttaGTTAtaatg	1.0	0.855
	V$HMBOX.01	+4741	(-)	tctagctaGTTAgcagt	1.0	0.951
	V$HMBOX.01	+7045	(-)	aggtacttGTTAttcca	1.0	0.877
	V$HMBOX.01	+7195	(+)	tgtgacttGTTAtttca	1.0	0.883
	V$HMBOX.01	+7426	(+)	tacttttaGTTAttttc	1.0	0.860
	V$HMBOX.01	+7510	(-)	caaaaataGTTAtaatg	1.0	0.859
	V$HNF1.01	+1208	(+)	aGTTAattctgaagaaa	1.0	0.837
	V$HNF1.01	+1229	(-)	aGTTAattgtatcagtc	1.0	0.819
	V$HNF1.01	+2179	(-)	aGTTAaggtttagagtc	1.0	0.823
	V$HNF1.01	+2529	(-)	aGTTActttctgcttct	1.0	0.805
	V$HNF1.01	+2904	(-)	tGTTAattggtagcttt	1.0	0.853
	V$HNF1.01	+3891	(-)	tGTTAattacttcattg	1.0	0.850
	V$HNF1.01	+5741	(-)	aGTTAttttttttttta	1.0	0.857
	V$HNF1.01	+7523	(-)	aGTTAatgggtgacaaa	1.0	0.859
	V$HNF1.02	+3706	(-)	tgcTAATtataatgaca	1.0	0.805
	V$HNF1.03	+3281	(+)	gGTTAataatcttgatt	1.0	0.882
	V$HNF1.04	+1201	(+)	aaggctcaGTTAattct	1.0	0.897
	V$HNF1.04	+2023	(+)	ctcgctctGTTAagcgg	1.0	0.844
	V$HNF1.04	+2186	(-)	tggctgcaGTTAaggtt	1.0	0.864
	V$HNF1.04	+2287	(-)	tgagatcaGTTAccaca	1.0	0.858
	V$HNF1.04	+2406	(-)	gttcttaaGTTAcccac	1.0	0.880
	V$HNF1.04	+2886	(+)	tactctttGTTAaaggg	1.0	0.864
	V$HNF1.04	+3691	(+)	ctttattgGTTAaaatg	1.0	0.881
	V$HNF1.04	+4180	(-)	caggcttgGTTAtcaga	1.0	0.845
	V$HNF1.04	+6121	(-)	tatgcccaGTTAagcat	1.0	0.861
	V$HNF1.04	+6242	(+)	aagcatatGTTAagccc	1.0	0.845
	V$HNF1.04	+7919	(+)	acttgtgaGTTAaatag	1.0	0.902
	V$TCF2.01	+1236	(-)	cccaagtaGTTAattgt	1.0	0.893
	V$TCF2.01	+7530	(-)	tggggataGTTAatggg	1.0	0.891
	V$TCF2.01	+7583	(-)	gaaggatgGTTAccaga	1.0	0.803
V$HNF6	V$HNF6.01	+1730	(-)	attgagagTCAAtgtct	1.0	0.854
	V$HNF6.01	+3287	(-)	ttttgaaaTCAAgatta	1.0	0.847
	V$HNF6.01	+3744	(+)	tcttgtatTCAAtattc	1.0	0.875
	V$HNF6.01	+3751	(+)	ttcaatatTCAAtatta	1.0	0.885
	V$HNF6.01	+6684	(-)	atatttacTCAAtcact	1.0	0.841
	V$HNF6.01	+7456	(-)	gactatagTCAAtaatt	1.0	0.906
	V$HNF6.02	+7802	(+)	agtagtaatCAATcact	1.0	0.965
	V$OC2.01	+1291	(-)	taaacaAATCagaactt	1.0	0.832
	V$OC2.01	+3156	(-)	acatacAATCaaatgtc	1.0	0.866
	V$OC2.01	+4421	(+)	agacacAATCtatactg	1.0	0.847
	V$OC2.01	+5143	(+)	aaaagaAATCaaaatgt	1.0	0.943
	V$OC2.01	+7410	(+)	acaaacAATCcagttat	1.0	0.869
V$HOMF	V$BARX1.01	+8066	(+)	ttaatggaAATTggtgaaa	1.0	0.916
	V$BARX2.01	+1204	(+)	gctcagtTAATtctgaaga	1.0	0.967
	V$BARX2.01	+1231	(-)	aagtagtTAATtgtatcag	1.0	0.967
	V$BARX2.01	+2386	(-)	cagaattTAATgagaagat	1.0	0.951
	V$BARX2.01	+7525	(-)	ggatagtTAATgggtgaca	1.0	0.984
	V$BSX.01	+1230	(+)	actgatacAATTaactact	1.0	0.953

	V$BSX.01	+3707	(+)	gtcattatAATTagcaaat	1.0	0.967
	V$BSX.01	+3708	(-)	catttgctAATTataatga	1.0	0.960
	V$BSX.01	+3892	(+)	aatgaagtAATTaacactt	1.0	0.982
	V$BSX.01	+3893	(-)	aaagtgttAATTacttcat	1.0	0.976
	V$BSX.01	+5209	(+)	gtatgtgtAATTaaaggaa	1.0	0.955
	V$HHEX.01	+1235	(-)	gcccaagtagtTAATtgta	1.0	0.956
	V$HHEX.01	+2390	(-)	cactcagaattTAATgaga	1.0	0.966
	V$HHEX.01	+2921	(-)	acagaccatttTAATcggc	1.0	0.972
	V$HHEX.01	+4557	(-)	tttccataattTAATattt	1.0	0.972
	V$HHEX.01	+5214	(-)	cttagttccttTAATtaca	1.0	0.977
	V$HHEX.01	+5300	(-)	atgaaaattttTAATtcag	1.0	0.964
	V$HHEX.01	+7265	(+)	ttattttttttTAATtctt	1.0	0.970
	V$HHEX.01	+8056	(+)	gaggttcagttTAATggaa	1.0	0.969
	V$HMX2.01	+1798	(+)	tttgggggaCTTAagcaac	1.0	0.897
	V$HMX2.01	+1803	(-)	aggaagttgCTTAagtccc	1.0	0.892
	V$HMX2.01	+2405	(+)	agtgggtaaCTTAagaacc	1.0	0.926
	V$HMX2.01	+2410	(-)	agactggttCTTAagttac	1.0	0.915
	V$HMX2.01	+4614	(+)	taaacacatCTTAatttga	1.0	0.842
	V$HMX2.02	+969	(+)	aaacactaAAACgattgcc	1.0	0.929
	V$HMX2.02	+1632	(+)	aagtttatAAACatttcat	1.0	0.843
	V$HMX2.02	+2322	(-)	tgtgtcccAAACgatcaca	1.0	0.852
	V$HMX2.02	+6023	(+)	aaaatcccAAACgttttgc	1.0	0.971
	V$HMX2.02	+6028	(-)	ggcatgcaAAACgtttggg	1.0	0.973
	V$HMX2.03	+2917	(-)	accattTTAAtcggctgtt	1.0	0.843
	V$HMX3.01	+2248	(+)	taaatgccAAGTggcttgt	1.0	0.922
	V$HMX3.01	+4970	(+)	gttgtcacAAGTtggagtg	1.0	0.899
	V$HMX3.01	+5572	(-)	ctattcagAAGTggttagg	1.0	0.905
	V$HMX3.01	+7723	(+)	gcaggcatAAGTgtgtgca	1.0	0.904
	V$HMX3.02	+1200	(+)	gaaggctcagTTAAttctg	1.0	0.925
	V$HMX3.02	+2022	(+)	tctcgctctgTTAAgcggt	1.0	0.937
	V$HMX3.02	+2027	(-)	taaagaccgcTTAAcagag	1.0	0.943
	V$HMX3.02	+2237	(+)	cgtgttgaccTTAAatgcc	1.0	0.922
	V$HMX3.02	+2242	(-)	cacttggcatTTAAggtca	1.0	0.922
	V$HMX3.02	+2905	(+)	aagctaccaaTTAAcagcc	1.0	0.964
	V$HMX3.02	+2910	(-)	taatcggctgTTAAttggt	1.0	0.969
	V$HMX3.02	+5131	(+)	atgaggacatTTAAaagaa	1.0	0.945
	V$HMX3.02	+5136	(-)	ttgatttcttTTAAatgtc	1.0	0.946
	V$MSX.01	+507	(+)	gtttctcTAATtggggttg	1.0	1.000
	V$MSX.01	+3876	(+)	agagaatTAATttcacaat	1.0	0.972
	V$MSX.01	+5210	(-)	gttccttTAATtacacata	1.0	0.983
	V$MSX.01	+5296	(-)	aaattttTAATtcagaaaa	1.0	0.977
	V$MSX.01	+7269	(+)	tttttttTAATtcttaatc	1.0	0.977
	V$MSX2.01	+3477	(+)	acctggCTAAttctttgta	1.0	0.956
	V$NOBOX.01	+2906	(-)	cggctgtTAATtggtagct	1.0	1.000
	V$NOBOX.02	+508	(-)	ccaaccccAATTagagaaa	1.0	0.945
	V$NOBOX.02	+4618	(+)	cacatcttAATTtgaatag	1.0	0.891
	V$TLX1.01	+1378	(-)	gaggttgCGGTgagccgag	1.0	0.879
	V$TLX2.01	+3873	(-)	gtgaaaTTAAttctctgac	1.0	0.851
	V$TLX2.01	+4556	(+)	taaataTTAAattatggaa	1.0	0.850
V$HOXC	V$HOX_PBX.01	+1972	(+)	cgccTGATggattgacc	1.0	0.934
	V$HOX_PBX.01	+2839	(+)	ggacTGATtgctgggtg	1.0	0.886
	V$HOXA9.01	+4251	(+)	gggatGATTcatgtccc	1.0	0.925
	V$HOXB4.02	+7804	(-)	gcagTGATtgattacta	1.0	0.967
	V$HOXC9.02	+5958	(-)	gctattaTTTAtatcat	1.0	0.914
	V$HOXC9.02	+7308	(+)	atatataTTTAtggcat	1.0	0.910
	V$MEIS1.03	+3775	(-)	tgggtGATTcattcaag	1.0	0.908
	V$MEIS1.03	+6160	(-)	tttctGATTtacatttt	1.0	0.946
	V$MEIS1.03	+6624	(-)	tacttGATTcaaatgca	1.0	0.883
	V$MEIS1.03	+6682	(+)	aaagtGATTgagtaaat	1.0	0.890
	V$PBX1.01	+1976	(+)	tgatgGATTgaccccga	1.0	0.819
V$HOXF	V$HOX1-3.01	+509	(+)	ttctcTAATtggggttgga	1.0	0.850
	V$HOX1-3.01	+939	(-)	cccgaTAATtcagaataaa	1.0	0.826
	V$HOX1-3.01	+2117	(+)	gaggcTAATgctggtctca	1.0	0.852
	V$HOX1-3.01	+2161	(-)	ctggcTAATgagggttcta	1.0	0.887
	V$HOX1-3.01	+3282	(+)	gttaaTAATcttgatttca	1.0	0.853
	V$HOX1-3.01	+3754	(-)	caaaaTAATattgaatatt	1.0	0.864
	V$HOXA3.01	+2904	(-)	gctgtTAATtggtagcttt	1.0	0.866
	V$HOXA3.01	+3871	(-)	gaaatTAATtctctgactt	1.0	0.862

	V$HOXA3.02	+3276	(-)	caagattATTAacctcaaa	1.0	0.852
	V$HOXA3.02	+3706	(-)	tttgctaATTAtaatgaca	1.0	0.967
	V$HOXA3.02	+3709	(+)	cattataATTAgcaaatga	1.0	0.969
	V$HOXA3.02	+3874	(+)	tcagagaATTAatttcaca	1.0	0.843
	V$HOXA3.02	+5211	(+)	atgtgtaATTAaaggaact	1.0	0.966
	V$HOXA4.01	+1206	(+)	tcagttAATTctgaagaaa	1.0	0.828
	V$HOXA4.01	+7271	(+)	ttttttAATTcttaatctt	1.0	0.810
	V$HOXB3.01	+7501	(-)	agttaTAATgaatgaataa	1.0	0.850
	V$HOXB5.01	+3279	(+)	gaggtTAATaatcttgatt	1.0	0.857
	V$HOXB8.01	+1232	(+)	tgatacaATTAactacttg	1.0	0.853
	V$HOXB8.01	+2907	(+)	gctaccaATTAacagccga	1.0	0.868
	V$HOXB8.01	+3875	(-)	ttgtgaaATTAattctctg	1.0	0.894
	V$HOXC4.01	+5294	(-)	atttttAATTcagaaaaag	1.0	0.858
	V$HOXC4.01	+5297	(+)	tttctgAATTaaaaatttt	1.0	0.854
	V$HOXC6.01	+3878	(+)	agaattAATTtcacaatga	1.0	0.850
	V$HOXC6.01	+3894	(+)	tgaagtAATTaacactttc	1.0	0.988
	V$HOXC8.01	+3891	(-)	agtgttaATTActtcattg	1.0	0.995
	V$HOXC8.01	+5208	(-)	tcctttaATTAcacatact	1.0	0.987
	V$HOXC8.01	+6745	(-)	ggcctgaATTAcctggacc	1.0	0.851
	V$HOXC8.01	+7799	(-)	gtgattgATTActactgag	1.0	0.851
	V$HOXD3.01	+1229	(-)	gtagttAATTgtatcagtc	1.0	0.891
	V$HOXD8.01	+7450	(+)	caatgaaATTAttgactat	1.0	0.821
	V$NANOG.01	+2385	(-)	agaatttAATGagaagatc	1.0	0.941
	V$NANOG.01	+7498	(-)	tataatgAATGaataaggc	1.0	0.947
	V$NANOG.01	+7524	(-)	gatagttAATGggtgacaa	1.0	0.983
V$HOXH	V$MEIS1B_HOXA9.01	+3696	(-)	TGACattttaaccaa	1.0	0.908
V$HZIP	V$HOMEZ.01	+974	(-)	gggcaATCGttttag	1.0	0.897
	V$HOMEZ.01	+1408	(-)	ggagaATCGcttgaa	1.0	0.832
	V$HOMEZ.01	+2321	(+)	ctgtgATCGtttggg	1.0	0.878
	V$HOMEZ.01	+3416	(-)	ggagaATCGcttgaa	1.0	0.832
V$IKRS	V$IK1.01	+571	(-)	tcctGGGAagtcc	1.0	0.938
	V$IK2.01	+1115	(+)	ctctGGGAattgg	1.0	0.981
	V$IK2.01	+4484	(-)	gtttGGGAaacaa	1.0	0.993
	V$IK2.01	+6022	(-)	gtttGGGAttttg	1.0	0.982
	V$IK3.01	+2814	(-)	tcccaGGAAcact	1.0	0.846
	V$IK3.01	+2819	(+)	tcctgGGAAccca	1.0	0.867
	V$IK3.01	+4896	(-)	aggcgGGAAtatt	1.0	0.851
	V$IK3.01	+6446	(+)	agctgGGAAcaca	1.0	0.875
	V$IK3.01	+7041	(+)	tatttGGAAtaac	1.0	0.853
	V$LYF1.01	+3354	(-)	gccTGGGagactg	1.0	0.983
V$INSM	V$INSM1.01	+116	(+)	tgcttGGGGcgcg	1.0	0.910
	V$INSM1.01	+458	(+)	tgcgaGGGGaggc	1.0	0.915
	V$INSM1.01	+1502	(+)	tggtgGGGGgggg	1.0	0.909
	V$INSM1.01	+4098	(+)	tttcaGGGGatgc	1.0	0.913
	V$INSM1.01	+5352	(-)	tgcagGGGGacct	1.0	0.906
V$IRFF	V$IRF1.01	+954	(+)	cgggtgctcaaataGAAAcactaaa	1.0	0.877
	V$IRF1.01	+3507	(-)	ctggccaacacagtGAAAccccatc	1.0	0.878
	V$IRF1.01	+3624	(+)	tgcttttcaaaaaaGAAAtctcatg	1.0	0.889
	V$IRF2.01	+2888	(+)	ctctttgttaaaggGAAAagctacc	1.0	0.853
	V$IRF2.02	+3315	(-)	aagaaaaaGAAAagaaaagcattat	1.0	0.877
	V$IRF3.01	+2522	(+)	agtcatgagaagcaGAAAgtaactc	1.0	0.876
	V$IRF3.01	+5284	(-)	ttaattcagaaaaaGAAAgtctccc	1.0	0.958
	V$IRF3.01	+5316	(-)	ttgttacagaaaagGAAAataaatg	1.0	0.958
	V$IRF4.01	+3187	(+)	tactcatctgaaaaGAAAaaagaaa	1.0	0.951
	V$IRF4.01	+3321	(-)	catctcaagaaaaaGAAAagaaaag	1.0	0.941
	V$IRF4.01	+3941	(-)	attctacaccaataGAAAacatcac	1.0	0.986
	V$IRF4.01	+4818	(-)	tctaccaaaaaaagGAAAacaaaat	1.0	0.978
	V$IRF4.01	+5133	(+)	gaggacatttaaaaGAAAtcaaaat	1.0	0.969
	V$IRF4.03	+1331	(-)	tgggcaacaagagcGAAActgcgtc	1.0	0.964
	V$IRF4.03	+4672	(+)	tagttttaacaattGAAActacctt	1.0	0.881
	V$IRF4.03	+5255	(-)	tttggatgccatacGAAAcaatctt	1.0	0.911
	V$IRF7.01	+5139	(+)	atttaaaaGAAAtcaaaatgttgat	1.0	0.863
	V$ISGF3G.01	+4054	(-)	gttctcaccataagGAAActgaggc	1.0	0.860
V$IRXF	V$IRX2.01	+4724	(+)	cattCATGtaggg	1.0	0.862
	V$IRX3.01	+2210	(+)	tgttCATGtagta	1.0	0.865
	V$IRX5.01	+922	(-)	caaaCATGttgtg	1.0	0.944
	V$IRX5.01	+923	(+)	acaaCATGtttga	1.0	0.943
	V$IRX6.01	+2209	(-)	actaCATGaacaa	1.0	0.866

V$KLFS	V$BKLF.01	+3104	(+)	caccaGGGTggggttcaca	1.0	0.982
	V$BKLF.01	+6464	(-)	tagccGGGTgtggtggcgc	1.0	0.965
	V$BKLF.02	+827	(-)	gggcagGGAGggcatttg	1.0	0.992
	V$BKLF.02	+7543	(-)	agggtgGGAGggaggtggg	1.0	0.940
	V$BKLF.02	+7551	(-)	agtgagGGAGggtgggagg	1.0	0.940
	V$EKLF.01	+127	(+)	cggtccggagaGGGTtcgg	1.0	0.926
	V$EKLF.01	+6941	(-)	gctaccaaagaGGGTttgc	1.0	0.893
	V$EKLF.02	+866	(+)	atggtgGGTGtggcaaaat	1.0	0.988
	V$GKLF.01	+7381	(-)	aatgagaagAGGGataccc	1.0	0.892
	V$GKLF.02	+241	(-)	cgataatcAAAGgcgactg	1.0	0.989
	V$GKLF.02	+2889	(+)	tctttgttAAAGggaaaag	1.0	0.967
	V$GKLF.02	+3905	(+)	acactttcAAAGgactgta	1.0	0.991
	V$GKLF.02	+4604	(-)	atgtgtttAAAGgctgatt	1.0	0.970
	V$GKLF.02	+5213	(+)	gtgtaattAAAGgaactaa	1.0	0.981
	V$GKLF.02	+5933	(+)	ttttagtcAAAGgtaatgt	1.0	0.966
	V$GKLF.02	+7053	(-)	tttttttcAAAGgtacttg	1.0	0.975
	V$GKLF.02	+7091	(-)	taagcctcAAAGgggagct	1.0	0.991
	V$GKLF.02	+7645	(-)	gcatggtcAAAGgggccac	1.0	0.986
	V$GKLF.03	+5758	(+)	tagctgggtGTGGtgttgt	1.0	0.984
	V$GKLF.03	+6599	(-)	tggctgggtGTGGtggctc	1.0	0.988
	V$KKLF.01	+86	(+)	ggccgggcaGGGGagaggg	1.0	0.947
	V$KKLF.01	+154	(+)	gaccgggccGGGGcgcgcg	1.0	0.915
	V$KKLF.01	+1497	(+)	ggtggtggtGGGGgggggg	1.0	0.958
	V$KKLF.01	+1500	(+)	ggtggtgggGGGGgggggg	1.0	0.956
	V$KLF12.01	+3369	(+)	gagtgcaGTGGtgccatct	1.0	0.916
	V$KLF12.01	+6366	(+)	gagtgcaGTGGtgccatca	1.0	0.916
	V$KLF2.01	+2846	(+)	ttgctGGGTgggcctagaa	1.0	0.982
	V$KLF2.01	+4208	(+)	cttgtGGGTgggtagcatc	1.0	0.991
	V$KLF2.01	+6922	(-)	ctaagGGGTggtctggaga	1.0	0.982
	V$KLF2.01	+7547	(-)	agggaGGGTgggagggagg	1.0	0.982
	V$KLF6.01	+205	(+)	aggagcGGGGcggcgggca	1.0	0.890
	V$KLF6.01	+1503	(+)	ggtgggGGGGggggggtcc	1.0	0.951
	V$KLF6.01	+1506	(+)	ggggggGGGGgggtccctc	1.0	0.949
	V$KLF7.01	+117	(+)	gcttggGGCGcggtccgga	1.0	0.943
V$LEFF	V$LEF1.01	+2202	(-)	acatgaaCAAAggtctt	1.0	0.916
	V$LEF1.01	+2885	(-)	cctttaaCAAAgagtag	1.0	0.888
	V$LEF1.01	+4477	(-)	tgggaaaCAAAgcagat	1.0	0.914
	V$LEF1.01	+8102	(-)	aaagaaaCAAAgattat	1.0	0.871
	V$LEF1.02	+3905	(+)	acactttCAAAggactg	1.0	0.961
	V$LEF1.02	+3928	(+)	aaaatttCAAAgtgtga	1.0	0.986
	V$LEF1.02	+4539	(-)	ttcttttCAAAgcctct	1.0	0.969
	V$LEF1.02	+5175	(-)	aacatatCAAAgaccca	1.0	0.968
	V$LEF1.02	+7055	(-)	tttttttCAAAggtact	1.0	0.964
	V$LEF1.02	+7093	(-)	taagcctCAAAggggag	1.0	0.951
	V$LEF1.02	+7647	(-)	gcatggtCAAAggggcc	1.0	0.942
	V$LEF1.03	+1785	(-)	caaagaTCAAggatggg	1.0	0.833
	V$LEF1.03	+3998	(-)	ggcagaTCAAggagtag	1.0	0.843
	V$LEF1.04	+4701	(-)	cgtggtTCAAtaaaaac	1.0	0.841
	V$LEF1.04	+6682	(-)	atttacTCAAtcacttt	1.0	0.883
	V$LEF1.04	+7804	(+)	tagtaaTCAAtcactgc	1.0	0.979
	V$TCF7.01	+2480	(-)	aacagttCAAAagagaa	1.0	0.870
	V$TCF7.01	+3043	(-)	aaaacttCAAAaaataa	1.0	0.851
	V$TCF7.01	+4169	(-)	atcagatCAAAaaaaca	1.0	0.918
	V$TCF7L1.01	+5145	(+)	aagaaatCAAAatgttg	1.0	0.866
	V$TCF7L2.02	+243	(-)	cgataatcAAAGgcgac	1.0	0.952
	V$TCF7L2.02	+5933	(+)	ttttagtcAAAGgtaat	1.0	0.894
	V$TCF7L2.02	+6709	(+)	ggcaaatcAAAGagaag	1.0	0.964
V$LHXF	V$ISL1.01	+2113	(+)	tcaagaggcTAATgctggtctca	1.0	0.892
	V$ISL1.01	+2384	(-)	ctcagaattTAATgagaagatcc	1.0	0.844
	V$ISL1.01	+3275	(+)	ttttgaggtTAATaatcttgatt	1.0	0.880
	V$ISL1.01	+3700	(-)	tgctaattaTAATgacattttaa	1.0	0.821
	V$ISL2.01	+2903	(+)	aaaagctaccaATTAacagccga	1.0	0.920
	V$ISL2.01	+3890	(+)	acaatgaagtaATTAacactttc	1.0	0.926
	V$ISL2.01	+7522	(+)	ttttgtcacccATTAactatccc	1.0	0.893
	V$LHX1.01	+5294	(-)	gaaaattttt AATTcagaaaaag	1.0	0.857
	V$LHX2.01	+506	(-)	ctccaaccccAATTagagaaact	1.0	0.856
	V$LHX2.01	+2904	(-)	atcggctgttAATTggtagcttt	1.0	0.863
	V$LHX3.01	+1202	(+)	aggctcagTTAAttctgaagaaa	1.0	0.854

	V$LHX3.01	+1229	(-)	ccaagtagTTAAttgtatcagtc	1.0	0.864
	V$LHX3.01	+1232	(+)	tgatacaaTTAActacttgggca	1.0	0.855
	V$LHX3.01	+3871	(-)	ttgtgaaaTTAAttctctgactt	1.0	0.912
	V$LHX3.01	+3874	(+)	tcagagaaTTAAtttcacaatga	1.0	0.907
	V$LHX3.01	+7920	(+)	cttgtgagTTAAatagggtttgg	1.0	0.815
	V$LHX3.02	+1198	(+)	gagaaggctcagtTAATtctgaa	1.0	0.848
	V$LHX3.02	+1233	(-)	gtgcccaagtagtTAATtgtatc	1.0	0.838
	V$LHX3.02	+2127	(+)	ctggtctcatgtaTAATttaggt	1.0	0.840
	V$LHX3.02	+3870	(+)	taagtcagagaatTAATttcaca	1.0	0.881
	V$LHX3.02	+3875	(-)	ttcattgtgaaatTAATtctctg	1.0	0.833
	V$LHX3.02	+3886	(+)	tttcacaatgaagTAATtaacac	1.0	0.822
	V$LHX3.02	+5203	(+)	gaagaagtatgtgTAATtaaagg	1.0	0.825
	V$LHX3.02	+5298	(-)	aaatgaaaattttTAATtcagaa	1.0	0.839
	V$LHX3.02	+7527	(-)	aggtggggatagtTAATgggtga	1.0	0.833
	V$LHX4.01	+505	(+)	cagtttctctAATTggggttgga	1.0	0.901
	V$LHX6.01	+243	(-)	cgccctcgaTAATcaaaggcgac	1.0	0.849
	V$LHX6.01	+2161	(-)	gagtctggcTAATgagggttcta	1.0	0.841
	V$LHX6.01	+3705	(+)	atgtcattaTAATtagcaaatga	1.0	0.880
	V$LHX6.01	+3706	(-)	gtcatttgcTAATtataatgaca	1.0	0.912
	V$LHX6.01	+3891	(-)	tgaaagtgtTAATtacttcattg	1.0	0.936
	V$LHX6.01	+5207	(+)	aagtatgtgTAATtaaaggaact	1.0	0.889
	V$LHX6.01	+5208	(-)	tagttccttTAATtacacatact	1.0	0.902
	V$LMX1A.01	+5293	(+)	tctttttctgAATTaaaaatttt	1.0	0.862
V$LTSM	V$LTSM.01	+1777	(+)	gatcatacccCATCc	1.0	0.944
	V$LTSM.02	+7874	(-)	gaatcttaccCATCt	1.0	0.961
	V$LTSM.03	+3558	(+)	ATCCtcctgccttcg	1.0	0.845
	V$LTSM.03	+4094	(-)	ATCCcctgaaaataa	1.0	0.842
	V$LTSM.03	6224	(+)	ATCCagtgtgtataa	1.0	0.844
V$MAZF	V$MAZ.01	+457	(+)	ctgcGAGGgagg	1.0	0.991
	V$MAZ.01	+830	(-)	caggGAGGgcat	1.0	0.913
	V$MAZ.01	+3568	(-)	ttggGAGGcgaag	1.0	0.921
	V$MAZ.01	+6821	(-)	agggGAGGagggc	1.0	0.930
	V$MAZ.01	+6826	(-)	cagtGAGGgagg	1.0	0.921
	V$MAZR.01	+468	(+)	ggcggaGGGGctc	1.0	0.892
	V$MAZR.01	+1506	(+)	ggggggGGGGggg	1.0	0.948
	V$MAZR.01	+1509	(+)	ggggggGGGGtcc	1.0	0.977
	V$MAZR.01	+3107	(+)	cagggtGGGGttc	1.0	0.883
	V$MAZR.01	+7539	(-)	ggaggtGGGGata	1.0	0.885
V$MEF2	V$MEF2.06	+5739	(+)	tctaaaaaaaaAAATaacttagc	1.0	0.876
	V$SL1.01	+2066	(-)	aaagcacCTATgcatatttgtag	1.0	0.853
	V$SL1.01	+3116	(-)	acagacaCTATagatatgtgaac	1.0	0.884
	V$SL1.01	+4152	(+)	tcaaaccCTATatatactgtttt	1.0	0.936
V$MEF3	V$MEF3.01	+1530	(+)	tggTCAGgctggt	1.0	0.918
	V$MEF3.01	+2781	(+)	aggTCAGggacct	1.0	0.894
	V$SIX.01	+1555	(+)	accTCAGgtgacc	1.0	0.965
	V$SIX2.02	+1278	(+)	tcgTCAGcttcca	1.0	0.953
	V$SIX2.02	+4054	(+)	gccTCAGtttcct	1.0	0.959
	V$SIX2.02	+6005	(-)	attTCAGatttct	1.0	0.971
V$MITF	V$CLEAR.01	+1555	(-)	cgggTCACctgaggt	1.0	0.929
	V$MIT.01	+2469	(+)	agtgtCATGtgttct	1.0	0.931
	V$MIT.01	+5417	(-)	ctctgCATGtgcctc	1.0	0.926
	V$MIT.01	+5816	(-)	aggctCATGtgatcc	1.0	0.984
V$MIZ1	V$MIZ1.01	+195	(+)	tctgcCCTCca	1.0	0.962
	V$MIZ1.01	+982	(+)	attgcCCTCtg	1.0	0.994
	V$MIZ1.01	+4434	(+)	actgcCCTCcg	1.0	0.988
	V$MIZ1.01	+7866	(-)	tctgcCCTCtg	1.0	0.994
V$MOKF	V$MOK2.02	+1883	(+)	gtttgatgatggtCCTTgaat	1.0	0.981
	V$MOK2.02	+2138	(+)	tataatttaggtaCCTTttta	1.0	0.984
	V$MOK2.02	+2590	(-)	aaggggaactggtCCTTtcct	1.0	0.984
	V$MOK2.02	+2778	(+)	cacaggtcagggaCCTTtatg	1.0	0.992
	V$MOK2.02	+5347	(-)	tactgcagggggaCCTTgaga	1.0	0.989
	V$MOK2.02	+6739	(-)	tgaattacctggaCCTTgaaa	1.0	0.989
	V$MOK2.02	+6870	(+)	tgtgcttttggagCCTTgaca	1.0	0.991
	V$MOK2.02	+7046	(+)	ggaataacaagtaCCTTtgaa	1.0	0.984
	V$MOK2.02	+7109	(+)	agctccaacagcaCCTTtcct	1.0	0.986
V$MTF1	V$MTF-1.01	+3605	(+)	cactGCACtcggcct	1.0	0.965
	V$MTF-1.02	+4712	(-)	atgtacaCACGtggt	1.0	0.855
V$MYBL	V$CMYB.01	+2764	(-)	acctgtgcgCAACcgtcacag	1.0	0.990

	V$CMYB.01	+6842	(+)	gagcacctaCAACagctgtga	1.0	0.906
	V$CMYB.02	+2907	(+)	gctaccaatTAACagccgatt	1.0	0.973
	V$CMYB.02	+7847	(-)	tgtccccacTAACtgccccat	1.0	0.987
	V$MYBL1.01	+2758	(+)	cttcttctgtgACGGttgcgc	1.0	0.899
	V$MYBL1.01	+5366	(+)	tagcaacttatACGGtttctt	1.0	0.831
	V$VMYB.02	+1357	(+)	gctggagtgcAACGgcactat	1.0	0.982
	V$VMYB.04	+5154	(+)	aaatgttgatAACGttattgc	1.0	0.880
	V$VMYB.04	+5466	(+)	tacaggatgtAACGtaaatga	1.0	0.888
	V$VMYB.04	+7319	(-)	caaaacatctAACGtatgcca	1.0	0.884
	V$VMYB.05	+5159	(-)	acccagcaatAACGttatcaa	1.0	0.909
V$MYOD	V$E47.01	+6811	(-)	ggaggGCAGctgggccc	1.0	0.928
	V$E47.02	+4927	(+)	ggattacaGGTGtgagc	1.0	0.938
	V$E47.02	+6764	(+)	atggcacaGGTGatcct	1.0	0.935
	V$MYF5.01	+719	(-)	accagaCAGCggctgca	1.0	0.908
	V$MYF6.01	+6848	(+)	ctacaACAGctgtgaag	1.0	0.960
	V$MYOD.02	+699	(-)	cggccgCAGCtggtccc	1.0	0.929
	V$MYOGENIN.02	+635	(+)	gagcatCAGCtgaggct	1.0	0.910
	V$MYOGENIN.03	+6849	(-)	gcttcaCAGCtgttgta	1.0	0.961
	V$TCFE2A.02	+4014	(+)	caatgacaGATGacttt	1.0	0.943
	V$TCFE2A.02	+5074	(-)	tcaggtcaGATGctttg	1.0	0.943
	V$TCFE2A.02	+7867	(+)	agagggcaGATGggtaa	1.0	0.952
	V$TCFE2A.03	+698	(+)	cgggacCAGCtgcggcc	1.0	0.990
	V$TCFE2A.03	+6810	(+)	ggggccCAGCtgccctc	1.0	0.993
V$MYRF	V$MYRF.01	4261	(+)	atgtcCCAGgctg	1.0	0.910
V$MYT1	V$MYT1.01	+3681	(+)	gaaAAGTacactt	1.0	0.856
	V$MYT1.02	+1288	(+)	ccaAAGTtctgat	1.0	0.897
	V$MYT1.02	+1679	(+)	gtaAAGTtgaata	1.0	0.884
	V$MYT1.02	+1878	(+)	gagAAGTttgatg	1.0	0.893
	V$MYT1.02	+2263	(-)	catAAGTttacaa	1.0	0.890
	V$MYT1.02	+3050	(+)	ttgAAGTtttttt	1.0	0.893
	V$MYT1.02	+5435	(-)	caaAAGTtgtgag	1.0	0.898
	V$MYT1.02	+7080	(-)	ctgAAGTttgtat	1.0	0.884
	V$MYT1.02	+7908	(+)	cggAAGTttaaac	1.0	0.889
	V$MYT1.02	+7913	(-)	cacAAGTttaaac	1.0	0.888
	V$MYT1L.01	+933	(+)	tgagAGTTtattc	1.0	0.958
	V$MYT1L.01	+2051	(+)	tgatAGTTcgctg	1.0	0.958
	V$MYT1L.01	+3269	(+)	tgaaAGTTttgag	1.0	1.000
	V$MYT1L.01	+7531	(-)	ggatAGTTaatgg	1.0	0.925
V$MZF1	V$MZF1.01	+461	(+)	gaGGGGaggcg	1.0	0.992
	V$MZF1.01	+2510	(+)	ggGGGGaagat	1.0	0.996
	V$MZF1.01	+6824	(-)	gaGGGGaggag	1.0	0.992
	V$MZF1.01	+7537	(-)	gtGGGGatagt	1.0	1.000
	V$MZF1.01	+7859	(+)	gtGGGGacaga	1.0	1.000
	V$MZF1.02	+2600	(-)	aaGGGGaactg	1.0	1.000
	V$MZF1.02	+3063	(-)	gaGGGGaaaaa	1.0	0.998
	V$MZF1.02	+5408	(+)	ttGGGGaagga	1.0	0.995
	V$MZF1.02	+7090	(-)	aaGGGGagctg	1.0	0.991
	V$MZF1.02	+7841	(+)	taGGGGatggg	1.0	0.995
V$NACA	V$NACA1.01	+2664	(-)	aacaCAGAgcagg	1.0	0.934
	V$NACA1.01	+3871	(+)	aagtCAGAgaatt	1.0	0.934
	V$NACA1.01	+7832	(+)	aaccCAGAgtagg	1.0	0.950
V$NBRE	V$NBRE.01	+2199	(-)	aacaAAGGtcttggc	1.0	0.862
	V$NBRE.01	+2238	(-)	atttAAGGtcaacac	1.0	0.911
	V$NBRE.01	+2784	(-)	cataAAGGtccctga	1.0	0.861
V$NDPK	V$NM23.01	+1504	(+)	gtGGGGggggggggtc	1.0	0.951
V$NEUR	V$NEUROG.01	+2639	(-)	agcCCATttgccaaa	1.0	0.933
	V$NGN_NEUROD.01	+3857	(-)	tactCATCtgtaact	1.0	0.985
	V$NGN_NEUROD.01	+4015	(-)	aagtCATCtgtcatt	1.0	1.000
	V$NGN_NEUROD.01	+5075	(+)	aaagCATCtgacctg	1.0	0.989
	V$NGN_NEUROD.01	+7187	(+)	atagCATCtgtgact	1.0	0.987
	V$NGN_NEUROD.01	+7227	(+)	atctCATCtgccttg	1.0	0.988
	V$NGN_NEUROD.01	+7868	(-)	taccCATCtgccctc	1.0	0.988
	V$OLIG2.01	+700	(+)	ggaccaGCTGcggcc	1.0	0.988
	V$OLIG2.01	+6812	(+)	ggcccaGCTGccctc	1.0	0.992
V$NF1F	V$NF1.01	+3384	(+)	atcTTGGctcactgcaacctc	1.0	0.834
	V$NF1.02	+5519	(-)	ctgcTGGCagcttgacaggga	1.0	0.827
	V$NF1.03	+2547	(+)	taccttccttgatGCCAggga	1.0	0.983
	V$NF1.03	+2637	(-)	ggaaagcccatttGCCAaaga	1.0	0.955

	V$NF1.03	+3999	(+)	tactccttgatctGCCAatga	1.0	0.958
	V$NF1.03	+5001	(-)	caatacatatgctGCCAgaac	1.0	0.926
	V$NF1.03	+7017	(+)	agtttgttgtactGCCAaata	1.0	0.958
	V$NF1.04	+4100	(+)	tcaggggatgcattCCAAgac	1.0	0.914
	V$NF1.04	+6134	(+)	cataaggacatattCCAAaat	1.0	0.908
	V$NF1.04	+7099	(+)	tttgaggcttagctCCAAcag	1.0	0.910
V$NFAT	V$NFAT.01	+1659	(-)	ttttatGGAAacacacaat	1.0	0.957
	V$NFAT.01	+2645	(-)	acttgaGGAAagcccattt	1.0	0.976
	V$NFAT.01	+6972	(-)	cgtgaaGGAAaaaaatatt	1.0	0.972
	V$NFAT5.01	+4459	(+)	cagtGGAAatgcaacgaga	1.0	0.850
	V$NFAT5.01	+4815	(-)	aaaaGGAAacaaaatgtc	1.0	0.852
	V$NFAT5.01	+7369	(+)	tcatGGAAaatggggtatc	1.0	0.846
	V$NFAT5.01	+7781	(-)	gacaGGAAagacaatgacc	1.0	0.858
	V$NFAT5.02	+1657	(-)	ttatGGAAacacacaattt	1.0	0.896
	V$NFAT5.02	+3676	(+)	ttttGGAAaagtacacttt	1.0	0.970
	V$NFAT5.02	+4509	(+)	gattGGAAaataaactaaa	1.0	0.880
	V$NFAT5.02	+7114	(-)	gaaaGGAAaggtgctgttg	1.0	0.876
	V$NFAT5.02	+7204	(-)	ccatGGAAatgtgaaataa	1.0	0.872
	V$NFATC1.01	+7208	(-)	gcatccATGGaaatgtgaa	1.0	0.835
	V$NFATC1.01	+7213	(+)	atttccATGGatgcatctc	1.0	0.883
V$NFKB	V$CREL.01	+2646	(+)	aatgggctTTCCtca	1.0	0.989
	V$CREL.01	+3096	(-)	cctggtgtTTCCagg	1.0	0.961
	V$CREL.01	+4568	(-)	gaaggcatTTCCata	1.0	0.944
	V$NFKAPPAB.01	+567	(-)	ctGGGAagtcccttc	1.0	0.952
	V$NFKAPPAB.01	+568	(+)	aaGGGActtcccagg	1.0	0.991
	V$NFKAPPAB.01	+1510	(-)	gaGGGAcccccccc	1.0	0.915
	V$NFKAPPAB.01	+7377	(-)	gaGGGAtaccccatt	1.0	0.927
	V$NFKAPPAB50.01	+5987	(-)	ttgGGGAtgctcatc	1.0	0.865
V$NGRE	V$IR1_NGRE.01	+198	(-)	ccgctcctGGAGggc	1.0	0.880
	V$IR1_NGRE.01	+198	(+)	gccctccaGGAGcgg	1.0	0.880
	V$IR2_NGRE.01	+4785	(+)	tgCTCCcaggggata	1.0	0.888
V$NKX1	V$NKX11.01	+508	(-)	aaccccAATTagagaaa	1.0	0.878
	V$NKX11.01	+509	(+)	ttctctAATTggggttg	1.0	0.870
	V$NKX11.01	+2906	(-)	gctgttAATTggtagct	1.0	0.845
	V$NKX11.01	+2907	(+)	gctaccAATTaacagcc	1.0	0.842
	V$NKX12.01	+1231	(-)	gtagttAATTgtatcag	1.0	0.885
	V$NKX12.01	+3709	(+)	cattatAATTagcaaat	1.0	0.881
	V$NKX12.01	+3893	(-)	agtgttAATTacttcat	1.0	0.948
	V$NKX12.01	+3894	(+)	tgaagtAATTaacactt	1.0	0.921
	V$NKX12.01	+5210	(-)	tcctttAATTacacata	1.0	0.871
	V$NKX12.01	+5211	(+)	atgtgtAATTaaaggaa	1.0	0.886
V$NKX6	V$NKX61.01	+1206	(+)	tcagTTAAttctgaa	1.0	0.910
	V$NKX61.01	+1233	(-)	gtagTTAAttgtatc	1.0	0.923
	V$NKX61.01	+3279	(+)	gaggTTAAtaatctt	1.0	0.912
	V$NKX61.01	+3875	(-)	gaaaTTAAttctctg	1.0	0.910
	V$NKX61.01	+3878	(+)	agaaTTAAtttcaca	1.0	0.961
	V$NKX61.01	+3895	(-)	agtgTTAAttacttc	1.0	1.000
	V$NKX61.01	+4620	(+)	catcTTAAtttgaat	1.0	0.920
	V$NKX61.01	+5212	(-)	tcctTTAAttacaca	1.0	1.000
	V$NKX61.01	+5298	(-)	atttTTAAttcagaa	1.0	0.914
	V$NKX61.01	+7271	(+)	ttttTTAAttcttaa	1.0	0.910
	V$NKX61.02	+2908	(-)	gctgTTAAttggtag	1.0	0.936
	V$NKX61.02	+2919	(-)	cattTTAAtcggctg	1.0	0.910
	V$NKX61.02	+7527	(-)	atagTTAAtgggtga	1.0	0.871
	V$NKX61.02	+8062	(+)	cagtTTAAtggaaat	1.0	0.896
	V$NKX61.03	+3894	(+)	tgaagTAATtaacac	1.0	0.902
	V$NKX63.01	+2388	(-)	gaattTAATgagaag	1.0	0.885
	V$NKX63.01	+3709	(+)	cattaTAATtagcaa	1.0	0.872
V$NKXH	V$BAPX1.01	+7725	(+)	aggcatAAGTgtgtgcaaa	1.0	0.899
	V$NKX25.01	+5678	(-)	gggttcAAGTgatcctccc	1.0	1.000
	V$NKX25.02	+1206	(+)	tcagtTAATtctgaagaaa	1.0	0.899
	V$NKX25.02	+1229	(-)	gtagtTAATtgtatcagtc	1.0	0.927
	V$NKX25.02	+2904	(-)	gctgtTAATtggtagcttt	1.0	0.927
	V$NKX25.02	+3891	(-)	agtgtTAATtacttcattg	1.0	0.899
	V$NKX25.02	+4556	(-)	ttccaTAATttaatattta	1.0	0.936
	V$NKX25.02	+4620	(+)	catctTAATttgaatagag	1.0	0.957
	V$NKX25.05	+768	(+)	tggccTGAGtgggcatgac	1.0	0.994
	V$NKX25.05	+2339	(-)	accctTGAGtgggagcctg	1.0	0.998

	V$NKX25.05	+2398	(+)	aattcTGAGtgggtaactt	1.0	0.990
	V$NKX26.01	+2250	(+)	aatgccaAGTGgcttgtaa	1.0	0.912
	V$NKX26.01	+5570	(-)	attcagaAGTGgttaggtt	1.0	0.868
	V$NKX29.01	+2268	(-)	agtatcaaGTACataagtt	1.0	0.852
	V$NKX31.01	+2260	(-)	gtacatAAGTttacaagcc	1.0	0.876
	V$NKX31.01	+3228	(-)	tcagatAAGTatcatcctt	1.0	0.935
	V$NKX31.01	+7418	(-)	aactaaAAGTataactgga	1.0	0.846
	V$NKX31.02	+2140	(+)	taatttagGTACcttttta	1.0	0.821
	V$NKX31.02	+2267	(+)	aaacttatGTACttgatac	1.0	0.867
	V$NKX31.04	+1902	(-)	acttctaAGTGctaaatat	1.0	0.965
	V$NKX32.01	+1850	(-)	tggactaAGTGctgatttt	1.0	0.968
	V$NKX32.01	+2093	(-)	ctcactaAGTGaaaagtct	1.0	0.964
	V$NKX32.01	+4196	(+)	gctactaAGTGacttgtgg	1.0	0.969
V$NOLF	V$EBF1.01	+60	(-)	gccgcgTCCCccgcgccccgcgc	1.0	0.905
	V$EBF1.01	+141	(+)	ttcggcTCCCcgggaccgggccg	1.0	0.882
	V$EBF1.01	+306	(+)	cacacgTCCCcggggcggcacgg	1.0	0.964
	V$EBF1.01	+548	(-)	cttcgcTCCCccgcggccgtgat	1.0	0.912
	V$EBF1.01	+4781	(-)	ccaataTCCCctgggagcaaaat	1.0	0.984
	V$EBF1.01	+4782	(+)	ttttgcTCCCaggggatattggc	1.0	0.912
	V$EBF1.01	+6651	(+)	attggaTCCCttggggccgatgc	1.0	0.943
	V$OLF1.02	+589	(-)	tgtgggTCCCgggaggcgcccta	1.0	0.910
V$NR2F	V$COUP.01	+2197	(-)	actacatgaacaaAGGTcttggctg	1.0	0.895
	V$COUP.01	+2768	(+)	gacggttgcgcacAGGTcagggacc	1.0	0.904
	V$COUP.02	+1780	(-)	ccccaaagaTCAAggatggggtatg	1.0	0.888
	V$COUP.02	+3993	(-)	attggcagaTCAAggagtagggctc	1.0	0.857
	V$COUP.02	+6388	(-)	cctaggaggTCAAggctgcagtgag	1.0	0.941
	V$HNF4.01	+7642	(-)	tctgcatggtCAAAggggccacagg	1.0	0.838
	V$HNF4A.02	+5651	(-)	ctcagcatcccaAAGTgctgggatt	1.0	0.877
	V$HNF4G.01	+1279	(+)	cgtcagcttccAAAGttctgatttg	1.0	0.937
	V$NR2F6.01	+5930	(+)	atattttagtcaAAGGtaatgtctg	1.0	0.827
	V$PNR.01	+3149	(-)	gacatacaaTCAAatgtcacaaatc	1.0	0.884
V$NRF1	V$NRF1.01	+103	(-)	agcaGCGCccgggcgcc	1.0	0.802
	V$NRF1.01	+161	(-)	ctccGCGCgcgccccgg	1.0	0.826
	V$NRF1.02	+373	(-)	cagagCGCAggcggcgg	1.0	0.898
	V$NRF1.02	+1450	(-)	ggtggCGCAtgcctgta	1.0	0.814
	V$NRF1.02	+6455	(-)	ggtggCGCAtgcctgtg	1.0	0.814
	V$NRF1.02	+7695	(-)	cactgCGCAggctccta	1.0	0.922
V$NRSF	V$NRSF.02	+5086	(-)	ttgtgaacctcaGCACcactgacacttcagg	1.0	0.806
V$OCT1	V$OCT1.01	+2859	(-)	acTATGctattctag	1.0	0.823
	V$OCT1.02	+6032	(-)	ggcATGCaaaacgtt	1.0	0.961
	V$OCT1.02	+6617	(-)	caaATGCaagatatt	1.0	0.856
	V$OCT1.02	+7345	(+)	ggtATGCaatgccta	1.0	0.863
	V$OCT1.02	+7657	(+)	accATGCagatcatg	1.0	0.852
	V$OCT1.03	+3708	(+)	tcattataATTAgca	1.0	0.926
	V$OCT1.03	+3711	(-)	atttgctaATTAtaa	1.0	0.945
	V$OCT1.03	+3893	(+)	atgaagtaATTAaca	1.0	0.939
	V$OCT1.03	+3896	(-)	aagtgttaATTAct t	1.0	0.924
	V$OCT1.03	+5210	(+)	tatgtgtaATTAaag	1.0	0.927
	V$OCT1.03	+5213	(-)	ttcctttaATTAcac	1.0	0.897
	V$OCT1.03	+7503	(+)	attcattcATTAtaa	1.0	0.858
	V$OCT1.03	+7804	(-)	agtgattgATTActa	1.0	0.851
	V$OCT1.04	+4566	(+)	atTATGgaaatgcct	1.0	0.912
	V$OCT1.04	+6245	(+)	caTATGttaagccct	1.0	0.801
	V$OCT1.04	+6488	(-)	tcTATGaaaaataca	1.0	0.810
	V$OCT1.05	+2465	(-)	caCATGacacttggt	1.0	0.924
	V$OCT1.05	+3636	(-)	acCATGagatttctt	1.0	0.899
	V$OCT1.06	+4618	(+)	cacatcttAATTtga	1.0	0.833
	V$OCT1.06	+4623	(-)	tctattcaAATTaag	1.0	0.884
	V$OCT1.06	+5307	(-)	taaatgaaAATTttt	1.0	0.891
	V$OCT1.06	+8066	(+)	ttaatggaAATTggt	1.0	0.899
	V$POU3F3.01	+2068	(-)	cctatGCATatttgt	1.0	0.956
	V$POU3F3.01	+2071	(+)	aatatGCATaggtgc	1.0	0.935
	V$POU3F3.01	+8005	(+)	cttagGCATaatgcc	1.0	0.866
V$OSRF	V$OSR1.01	+5360	(+)	ctgcaGTAGcaac	1.0	0.920
V$OVOL	V$OVOL1.01	+1203	(+)	ggctcaGTTAattct	1.0	0.865
	V$OVOL1.01	+2287	(-)	agatcaGTTAccaca	1.0	0.830
	V$OVOL1.01	+3306	(+)	gcatcaGTTAtaatg	1.0	0.804
	V$OVOL1.01	+3786	(+)	cacccaGTTAgtttt	1.0	0.820

	V$OVOL1.01	+3898	(-)	gaaagtGTTAattac	1.0	0.806
	V$OVOL1.01	+5158	(-)	aataacGTTAtcaac	1.0	0.837
	V$OVOL1.01	+5161	(+)	gataacGTTAttgct	1.0	0.812
	V$OVOL1.01	+5470	(-)	atttacGTTAcatcc	1.0	0.847
	V$OVOL1.01	+7321	(+)	gcatacGTTAgatgt	1.0	0.839
V$P53F	V$P53.02	+766	(+)	catggcctgagtgggCATGacccgg	1.0	0.919
	V$P53.02	+775	(-)	tttgcaaagccgggtCATGcccact	1.0	0.982
	V$P53.02	+5555	(-)	tggttaggttatgacCATGcccatt	1.0	0.939
	V$P53.03	+921	(-)	gaataaactctcaaaCATGttgtga	1.0	0.925
	V$P53.06	+765	(-)	cgggtCATGcccactcaggccatgg	1.0	0.808
	V$P53.08	+1450	(-)	ccgggcatggtggcgCATGcctgta	1.0	0.888
	V$P53.08	+1451	(+)	acaggcatgcgccacCATGcccggc	1.0	0.897
	V$TP63.01	+4713	(+)	ccacgtgtgtacattCATGtaggga	1.0	0.832
V$PARF	V$DBP.01	+3846	(+)	gtatgTTATgtagttac	1.0	0.866
	V$DBP.01	+4562	(+)	ttaaaTTATggaaatgc	1.0	0.866
	V$DBP.01	+4634	(+)	tagagTTATgttaggac	1.0	0.843
	V$HLF.01	+6684	(+)	agtgattgaGTAAatat	1.0	0.877
	V$TEF.01	+1301	(-)	aaaccttagGTAAacaa	1.0	0.903
	V$TEF.01	+3889	(+)	cacaatgaaGTAAttaa	1.0	0.898
	V$TEF_HLF.01	+5471	(-)	ttcatTTACgttacatc	1.0	0.806
	V$VBP.01	+1938	(+)	ctaagttacGTAAgaag	1.0	0.949
	V$VBP.01	+1939	(-)	gcttcttacGTAActta	1.0	0.994
	V$VBP.01	+2152	(-)	gggttctatGTAAaaag	1.0	0.870
	V$VBP.01	+2706	(-)	gctcctcaaGTAAcaca	1.0	0.890
	V$VBP.01	+5325	(+)	tccttttctGTAAcaac	1.0	0.893
	V$VBP.01	+5465	(+)	ctacaggatGTAAcgta	1.0	0.912
	V$VBP.01	+5470	(+)	ggatgtaacGTAAatga	1.0	0.914
	V$VBP.01	+6290	(+)	aaaaataagGTAAtatt	1.0	0.865
V$PAX2	V$PAX2.01	+2950	(-)	ttcagaataaggaatttAAACat	1.0	0.805
	V$PAX2.01	+4670	(+)	gctagttttaacaattgAAACta	1.0	0.851
	V$PAX2.01	+4672	(-)	ggtagtttcaattgttaAAACta	1.0	0.801
	V$PAX2.01	+7900	(+)	tagtgtgacggaagtttAAACtt	1.0	0.805
V$PAX3	V$PAX3.01	+403	(-)	cTCGTcaccggatggggct	1.0	0.817
	V$PAX3.02	+7185	(-)	caagTCACagatgctatga	1.0	0.868
	V$PAX3.02	+7893	(-)	tccgTCACactagcctgtg	1.0	0.896
	V$PAX3.03	+3640	(+)	aatctCATGgttggaagtt	1.0	0.956
	V$PAX3.03	+4767	(-)	aaaatCATGgctagttgag	1.0	0.931
V$PAX5	V$PAX5.04	+5830	(-)	tcagggctcACTGcagcctccacctccca	1.0	0.905
	V$PAX5.04	+6382	(+)	tcacagctcACTGcagccttgacctccta	1.0	0.880
	V$PAX8.01	+1728	(-)	taccctaaggaTTGAgagtcaatgtctaa	1.0	0.888
	V$PAX8.01	+2336	(-)	aggagctacccTTGAgtgggagcctgtgt	1.0	0.915
	V$PAX8.01	+3150	(+)	atttgtgacatTTGAttgtatgtctctct	1.0	0.885
	V$PAX8.01	+3765	(+)	ttattttgtgcTTGAatgaatcacccagt	1.0	0.907
V$PAX6	V$PAX6.02	+2376	(-)	tgagaagatCCAGttcagg	1.0	0.916
	V$PAX6.04	+6629	(-)	tgtCCACtacttgattcaa	1.0	0.851
V$PAX7	V$PAX7.01	+6628	(-)	actacttGATTcaaa	1.0	0.829
	V$PAX7.01	+7804	(-)	agtgattGATTacta	1.0	0.880
V$PAXH	V$PAX4.02	+509	(-)	accccAATTagagaa	1.0	0.841
	V$PAX4.02	+2908	(+)	ctaccAATTaacagc	1.0	0.850
	V$PAX4.02	+3709	(-)	ttgctAATTataatg	1.0	0.902
	V$PAX4.02	+3710	(+)	attatAATTagcaaa	1.0	0.928
	V$PAX4.02	+3894	(-)	gtgttAATTacttca	1.0	0.910
	V$PAX4.02	+3895	(+)	gaagtAATTaacact	1.0	0.929
	V$PAX4.02	+5211	(-)	cctttAATTacacat	1.0	0.898
	V$PAX4.02	+5212	(+)	tgtgtAATTaaagga	1.0	0.901
	V$PAX6_HD.01	+3878	(-)	tgtgaAATTaattct	1.0	0.863
V$PBXC	V$PBX1_MEIS1.02	+6160	(-)	tttcTGATttacatttt	1.0	0.846
V$PDX1	V$IPF1.01	+2908	(-)	atcggctgtTAATtggtag	1.0	0.847
	V$IPF1.01	+3705	(+)	atgtcattaTAATtagcaa	1.0	0.945
	V$IPF1.01	+3710	(-)	gtcatttgcTAATtataat	1.0	0.927
	V$IPF1.01	+3890	(+)	acaatgaagTAATtaacac	1.0	0.969
	V$IPF1.01	+3895	(-)	tgaaagtgtTAATtacttc	1.0	0.962
	V$IPF1.01	+5207	(+)	aagtatgtgTAATtaaagg	1.0	0.907
	V$IPF1.01	+5212	(-)	tagttccttTAATtacaca	1.0	0.903
V$PEG3	V$PEG3.01	+2249	(+)	aaatgccaagTGGCt	1.0	0.899
V$PERO	V$PPARG.02	+7645	(-)	atctgcatggtcAAAGgggccac	1.0	0.885
	V$PPARG.03	+241	(-)	ccctcgataatcAAAGgcgactg	1.0	0.837
	V$PPARG.03	+1663	(+)	tgtgtttccataAAAGgtaaagt	1.0	0.843

	V$PPARG.03	+1790	(-)	cttaagtcccccAAAGatcaagg	1.0	0.835
	V$PPARG.03	+2200	(-)	tactacatgaacAAAGgtcttgg	1.0	0.869
	V$PPARG.03	+5929	(+)	aatattttagtcAAAGgtaatgt	1.0	0.915
	V$PPARG.03	+7091	(-)	gagctaagcctcAAAGgggagct	1.0	0.835
V$PIT1	V$PIT1.01	+2133	(-)	ctaaaTTATacatga	1.0	0.990
	V$PIT1.01	+7496	(+)	cggccTTATtcattc	1.0	0.921
	V$PIT1.02	+1234	(-)	agtagTTAAttgtat	1.0	0.814
	V$PIT1.02	+1235	(+)	tacaaTTAActactt	1.0	0.836
	V$PIT1.02	+3876	(-)	tgaaaTTAAttctct	1.0	0.877
	V$PIT1.02	+3877	(+)	gagaaTTAAtttcac	1.0	0.888
V$PLAG	V$PLAG1.01	+328	(-)	acGAGGctccgcagaaggtggcc	1.0	0.891
	V$PLAG1.01	+459	(+)	gcGAGGggaggcggaggggctcg	1.0	0.913
	V$PLAG1.01	+2714	(+)	ttGAGGagcagtgtagggcagtg	1.0	0.890
	V$PLAG1.01	+6421	(-)	tcGAGGggctgaggtgggaggat	1.0	0.935
	V$PLAG1.02	+2508	(+)	taGGGGggaagatcagtcatgag	1.0	1.000
	V$PLAG1.02	+4073	(-)	aaGGGGgagactactgtgttctc	1.0	1.000
	V$PLAG1.02	+4104	(-)	caGGGGgtcttggaatgcatccc	1.0	1.000
	V$PLAG1.02	+5340	(-)	caGGGGgaccttgagagaagagt	1.0	1.000
	V$PLAGL1.01	+90	(-)	cccggGCGCcctctcccctgccc	1.0	0.941
	V$PLAGL1.01	+99	(+)	agaggGCGCccgggcgctgcttg	1.0	0.953
	V$PLAGL1.01	+579	(-)	gggagGCGCcctatgcagtcctg	1.0	0.877
	V$PLAGL1.01	+588	(+)	ataggGCGCctcccgggacccac	1.0	0.871
	V$PLAGL1.02	+1511	(+)	ggggGGGGtccctccatgttggt	1.0	0.892
	V$PLAGL1.02	+1534	(-)	gtcgGGGGttcgagaccagcctg	1.0	0.844
	V$PLAGL1.02	+7376	(+)	aaatGGGGtatccctcttctcat	1.0	0.815
V$PLZF	V$PLZF.01	+2984	(-)	accTACAgtaagcag	1.0	0.896
	V$PLZF.02	+3912	(-)	aagTACAgtcctttg	1.0	0.936
	V$PLZF.02	+4224	(+)	atcTACAgtgtagat	1.0	0.932
	V$PLZF.02	+7240	(-)	gccTACAgtgcagca	1.0	0.883
V$PRDF	V$BLIMP1.01	+500	(-)	aattagaGAAActggagcg	1.0	0.851
	V$BLIMP1.01	+5305	(-)	aataaatGAAAatttttaa	1.0	0.816
	V$BLIMP1.01	+5478	(+)	cgtaaatGAAAgatgcagt	1.0	0.857
	V$BLIMP1.01	+7116	(-)	aggaaagGAAAggtgctgt	1.0	0.820
	V$BLIMP1.01	+8065	(+)	tttaatgGAAAttggtgaa	1.0	0.837
	V$PRDM1.01	+2090	(-)	actaagtGAAAagtctttg	1.0	0.855
	V$PRDM1.01	+3878	(-)	tcattgtGAAAttaattct	1.0	0.816
	V$PRDM1.02	+2529	(+)	agaagcaGAAAgtaactct	1.0	0.835
	V$PRDM1.02	+5283	(-)	agaaaaaGAAAgtctccct	1.0	0.886
V$PTF1	V$PTF1.01	+6690	(-)	cccaCCTGtgatatttactca	1.0	0.829
V$PURA	V$PURALPHA.01	+465	(+)	ggAGGCggagggg	1.0	0.989
	V$PURALPHA.01	+3565	(-)	ggAGGCgaaggca	1.0	0.977
V$RBP2	V$PLU1_JARID1B.01	+7476	(-)	GCACagcag	1.0	0.962
V$RBPF	V$RBPJK.01	+2818	(+)	ttccTGGGaaccc	1.0	0.896
	V$RBPJK.01	+6887	(+)	gacaTGGGaagga	1.0	0.896
	V$RBPJK.02	+572	(-)	gtccTGGGaagtc	1.0	0.941
	V$RBPJK.02	+3139	(+)	agagTGGGaagat	1.0	0.966
	V$RBPJK.02	+4485	(-)	agttTGGGaaaca	1.0	0.942
V$RORA	V$REV-ERBA.01	+772	(-)	gcaaagccggGTCAtgcccactcag	1.0	0.884
	V$REV-ERBA.01	+1522	(+)	ctccatgttgGTCAggctggtctcg	1.0	0.912
	V$REV-ERBA.01	+5589	(-)	tttgaacatgGTCAttctgaaacct	1.0	0.888
	V$REV-ERBA.02	+3863	(+)	agatgagtaaGTCAgagaattaatt	1.0	0.805
	V$RORA.01	+2773	(+)	ttgcgcacagGTCAgggacctttat	1.0	0.871
	V$RORA.01	+5072	(-)	gacacttcagGTCAgatgctttgct	1.0	0.852
	V$RORA.01	+6390	(-)	agcctaggagGTCAaggctgcagtg	1.0	0.880
	V$RORA1.01	+2231	(-)	ggcatttaaGGTCaacacgagattc	1.0	0.955
	V$RORA1.01	+7772	(+)	atgtctggaGGTCattgtctttcct	1.0	0.944
	V$VERBA.01	+2102	(+)	acttagtgaGGTCaagaggctaatg	1.0	0.937
V$RREB	V$RREB1.01	+110	(-)	cCCCAagcagcgccc	1.0	0.825
	V$RREB1.01	+436	(+)	cCCCAgtcccaccag	1.0	0.821
	V$RREB1.01	+1495	(-)	cCCCAccaccaccac	1.0	0.811
	V$RREB1.01	+2322	(-)	tCCCAaacgatcaca	1.0	0.823
V$RU49	V$RU49.01	+1966	(+)	gAGTAcc	1.0	0.990
	V$RU49.01	+7054	(+)	aAGTAcc	1.0	1.000
V$RUSH	V$SMARCA3.01	+748	(-)	caCCATttctt	1.0	0.961
	V$SMARCA3.01	+861	(-)	caCCATttcct	1.0	0.973
	V$SMARCA3.01	+1189	(-)	tcCCATttctt	1.0	0.961
	V$SMARCA3.01	+6671	(+)	tgCCATttcta	1.0	0.962
	V$SMARCA3.02	+1698	(+)	atttACTTttc	1.0	0.986

	V$SMARCA3.02	+1914	(-)	tagcACTTcta	1.0	0.987
	V$SMARCA3.02	+2280	(+)	tgatACTTgtg	1.0	0.993
	V$SMARCA3.02	+3233	(+)	tgatACTTatc	1.0	0.993
	V$SMARCA3.02	+3681	(-)	gtgtACTTttc	1.0	1.000
	V$SMARCA3.02	+5204	(-)	acatACTTctt	1.0	0.987
	V$SMARCA3.02	+7051	(-)	aggtACTTgtt	1.0	1.000
	V$SMARCA3.02	+7423	(+)	ttatACTTtta	1.0	0.993
	V$SMARCA3.02	+7728	(-)	acacACTTatg	1.0	0.986
V$RXRF	V$CAR_RXR.01	+6742	(+)	caaGGTCcaggtaattcaggccatg	1.0	0.821
	V$PXR_RXR.01	+428	(+)	tctGGTCaccccagtcccaccaggt	1.0	0.830
	V$RAR_RXR.01	+2769	(+)	acggttgcgcacAGGTcagggacct	1.0	0.824
	V$RAR_RXR.01	+5931	(+)	tattttagtcaaAGGTaatgtctga	1.0	0.841
	V$RAR_RXR.03	+5103	(+)	gctgagGTTCacaataagctcctgt	1.0	0.817
	V$RARG.01	+2319	(+)	tcctgtgatcgTTTGggacacaggc	1.0	0.877
	V$RARG.01	+3289	(-)	actgatgctctTTTGaaatcaagat	1.0	0.882
	V$RARG.01	+3723	(-)	aagaatgccatTTTGtatagtcatt	1.0	0.865
	V$RARG.01	+6865	(+)	catcttgtgctTTTGgagccttgac	1.0	0.874
	V$THR.01	+1546	(-)	gcgggtcacctGAGGtcgggggttc	1.0	0.884
	V$THRA.01	+6397	(-)	tcatttgagcctaggAGGTcaaggc	1.0	0.823
	V$THRB.01	+6396	(+)	agccttGACCtcctaggctcaaatg	1.0	0.805
	V$VDR_RXR.01	+7857	(+)	tagtggggacaGAGGgcagatgggt	1.0	0.910
	V$VDR_RXR.04	+2103	(+)	cttagtgaGGTCaagaggctaatgc	1.0	0.875
	V$VDR_RXR.04	+7643	(-)	atctgcatGGTCaaaggggccacag	1.0	0.861
	V$VDR_RXR.06	+2765	(+)	tgtgacggttgcgcacAGGTcaggg	1.0	0.828
V$SAL1	V$SALL1.01	+5961	(+)	atATAAataatag	1.0	0.971
	V$SALL1.01	+6151	(+)	aaATAAaaaaaaa	1.0	0.995
V$SAL2	V$SALL2.01	+2849	(+)	ctggGTGGgcc	1.0	0.952
	V$SALL2.01	+3107	(+)	caggGTGGggt	1.0	0.986
	V$SALL2.01	+4211	(+)	gtggGTGGgta	1.0	0.961
	V$SALL2.01	+6927	(-)	agggGTGGtct	1.0	0.917
	V$SALL2.01	+7552	(-)	gaggGTGGgag	1.0	0.970
V$SATB	V$SATB1.01	+3755	(-)	aatAATAttgaatat	1.0	0.957
V$SF1F	V$FTF.01	+5238	(-)	acttCAAGgtgggca	1.0	0.950
	V$FTF.01	+6559	(-)	aggcCAAGgtgggca	1.0	0.949
	V$FTF.01	+6738	(+)	gtttCAAGgtccagg	1.0	0.977
	V$FTF.02	+5346	(+)	ctctcAAGGtccccc	1.0	0.913
	V$SF1.01	+2677	(+)	tcagCAAGgacagga	1.0	0.951
	V$SF1.01	+4398	(-)	tagaCAAGgccacta	1.0	0.996
V$SIX3	V$SIX3.02	+3580	(-)	atgcctgTAATcccagcactt	1.0	0.946
	V$SIX3.02	+5643	(+)	atgcctgTAATcccagcactt	1.0	0.946
V$SIXF	V$SIX1.01	+4179	(-)	gcttggtTATCagat	1.0	0.877
	V$SIX1.01	+5852	(+)	gccctgaTATCatgt	1.0	0.851
	V$SIX1.01	+5853	(-)	gacatgaTATCaggg	1.0	0.862
	V$SIX3.01	+7377	(+)	aatggggTATCcctc	1.0	0.892
V$SMAD	V$GC_SBE.01	+77	(+)	cgcggCTCCgg	1.0	0.946
	V$SMAD.01	+5947	(+)	aatGTCTgact	1.0	0.987
	V$SMAD3.01	+424	(+)	gcaGTCTggtc	1.0	0.994
	V$SMAD3.01	+489	(-)	gccGTCTggtt	1.0	0.997
	V$SMAD3.01	+726	(+)	gctGTCTggtt	1.0	0.997
	V$SMAD3.01	+842	(-)	acaGTCTgggc	1.0	0.994
	V$SMAD3.01	+2174	(-)	agaGTCTggct	1.0	0.995
	V$SMAD3.01	+2827	(-)	gttGTCTgggt	1.0	0.997
	V$SMAD3.02	+2365	(+)	tgtGTCTggtg	1.0	0.982
	V$SMAD3.02	+7771	(+)	tatGTCTggag	1.0	0.992
	V$SMAD4.01	+2199	(-)	aagGTCTtggc	1.0	0.943
	V$SMAD4.01	+2422	(+)	ccaGTCTaggc	1.0	0.995
	V$SMAD4.01	+4417	(-)	tgtGTCTcgac	1.0	0.967
V$SORY	V$HBP1.01	+3955	(+)	tggtgtagAATGagcctaaataa	1.0	0.863
	V$HBP1.01	+4713	(-)	cctacatgAATGtacacacgtgg	1.0	0.866
	V$HBP1.01	+7385	(-)	cacaaataAATGagaagagggat	1.0	0.876
	V$HBP1.02	+3772	(+)	gtgcttgAATGaatcacccagtt	1.0	0.985
	V$HBP1.02	+5304	(-)	gaaaataAATGaaaatttttaat	1.0	0.888
	V$HBP1.02	+5475	(+)	taacgtaAATGaaagatgcagta	1.0	0.885
	V$HBP1.02	+6231	(+)	gtgtataAATGaagcatatgtta	1.0	0.887
	V$HBP1.02	+7494	(-)	tataatgAATGaataaggccggg	1.0	0.991
	V$HMGA.01	+3871	(-)	ttgtgaaattAATTctctgactt	1.0	0.887
	V$HMGA.01	+6228	(+)	agtgtgtataAATGaagcatatg	1.0	0.884
	V$HMGA.01	+7497	(-)	agttataatgAATGaataaggcc	1.0	0.904

	V$HMGIY.01	+5306	(+)	taaaAATTttcatttattttcct	1.0	0.925
	V$SOX1.01	+7447	(+)	gtaCAATgaaattattgactata	1.0	0.875
	V$SOX1.03	+7618	(+)	gctCAATtgttttgagttttaga	1.0	0.824
	V$SOX1.04	+4550	(+)	aaaGAATaaatattaaattatgg	1.0	0.804
	V$SOX15.01	+1231	(+)	ctgatACAAttaactacttgggc	1.0	0.883
	V$SOX15.01	+5108	(+)	ggttcACAAtaagctcctgtgca	1.0	0.886
	V$SOX21.03	+1684	(+)	gttGAATaatgatcatttacttt	1.0	0.818
	V$SOX21.03	+5582	(+)	tctGAATaggtttcagaatgacc	1.0	0.830
	V$SOX21.03	+7869	(-)	tctGAATcttacccatctgccct	1.0	0.819
	V$SOX3.01	+4373	(+)	gccacaCAAAtgccaggttttgt	1.0	0.954
	V$SOX30.01	+7772	(-)	gaaagACAAtgacctccagacat	1.0	0.922
	V$SOX5.01	+1708	(+)	ctacaaCAATagtctcccttta	1.0	0.991
	V$SOX5.01	+4675	(+)	ttttaaCAATtgaaactaccttt	1.0	0.991
	V$SOX5.01	+5245	(-)	acgaaaCAATcttgaacttcaag	1.0	0.982
	V$SOX5.01	+7409	(+)	tacaaaCAATccagttatacttt	1.0	0.988
	V$SOX5.01	+7610	(-)	tcaaaaCAATtgagcccacagag	1.0	0.987
	V$SOX6.01	+2195	(-)	catgaACAAaggtcttggctgca	1.0	0.993
	V$SOX6.01	+2878	(-)	ctttaACAAagagtagctatccg	1.0	0.979
	V$SOX6.01	+3477	(-)	aaaatACAAagaattagccaggt	1.0	0.984
	V$SOX6.01	+4304	(+)	tcaccACAAagaatgtcattata	1.0	0.977
	V$SOX6.01	+4470	(-)	gggaaACAAagcagatctcgttg	1.0	0.994
	V$SOX6.01	+8095	(-)	aagaaACAAagattatcatttta	1.0	0.989
	V$SOX9.03	+4467	(-)	aaACAAagcagatctcgttgcat	1.0	0.804
	V$SRY.01	+3246	(-)	ggaaaACAAaaaagacatctatt	1.0	0.935
	V$SRY.02	+3700	(-)	tgctaATTAtaatgacattttaa	1.0	0.994
	V$SRY.02	+3705	(+)	atgtcATTAtaattagcaaatga	1.0	0.983
V$SP1F	V$SP1.03	+207	(+)	gagcgGGGCggcgggca	1.0	0.938
	V$SP1.03	+313	(+)	ccccgGGGCggcacggg	1.0	0.921
	V$SP1.03	+618	(-)	aaaagGGGCcgggccag	1.0	0.923
	V$SP1.03	+7958	(-)	tcaagGGGCagggatgc	1.0	0.913
	V$SP2.01	+438	(-)	ggacctggtgGGACtgg	1.0	0.810
	V$SP2.01	+642	(+)	agctgaggctGGACttg	1.0	0.856
	V$SP2.01	+2659	(-)	acacagagcaGGACttg	1.0	0.810
	V$SP2.01	+4234	(+)	tagataggctGGACata	1.0	0.829
	V$SP2.01	+4263	(+)	gtcccaggctGGACagt	1.0	0.804
	V$SP4.02	+1597	(+)	attataGGCGtgggcca	1.0	0.903
V$SPZ1	V$SPZ1.01	+7554	(-)	gGGAGggtggg	1.0	0.958
V$SREB	V$SREBP.01	+430	(+)	tggTCACcccagtcc	1.0	0.965
V$SRFF	V$SRF.04	+1665	(-)	tttacctttTATGgaaaca	1.0	0.923
	V$SRF.05	+328	(-)	ggctccgcagAAGGtggcc	1.0	0.805
	V$SRF.05	+1666	(+)	gtttccataaAAGGtaaag	1.0	0.947
	V$SRF.05	+3966	(+)	gagcctaaatAAGGagtaa	1.0	0.840
	V$SRF.05	+4648	(+)	gaccctttatAAGGcaaat	1.0	0.835
	V$SRF.05	+4823	(-)	ctaccaaaaaAAGGaaaac	1.0	0.825
	V$SRF.05	+5399	(-)	ccttccccaaAAGGctgcc	1.0	0.817
	V$SRF.05	+6189	(+)	tatttcaaatAAGGgatac	1.0	0.832
V$STAF	V$STAF.02	+7541	(+)	tccccacctccctCCCAccctccctcactac	1.0	0.841
V$STAT	V$STAT.01	+268	(+)	ggcgttcggGGAAggttgg	1.0	0.971
	V$STAT.01	+3259	(-)	aaactttcaGGAAaacaaa	1.0	0.912
	V$STAT.01	+3671	(+)	ccaatttttGGAAaagtac	1.0	0.876
	V$STAT.01	+5215	(+)	gtaattaaaGGAActaagc	1.0	0.914
	V$STAT.01	+5403	(+)	gccttttggGGAAggaggc	1.0	0.915
	V$STAT.01	+8062	(+)	cagtttaatGGAAattggt	1.0	0.898
	V$STAT1.02	+1646	(+)	ttcatactaGGAAattgtg	1.0	0.861
	V$STAT1.02	+2582	(+)	gaaatcctaGGAAaggacc	1.0	0.864
	V$STAT3.02	+2814	(+)	agtgTTCCtgggaacccag	1.0	0.954
	V$STAT3.02	+3088	(-)	gtgtTTCCaggatcagaga	1.0	0.961
	V$STAT5.01	+7128	(-)	ttacTTCTaagaaggaaag	1.0	0.975
	V$STAT5.01	+7130	(+)	ttccTTCTtagaagtaagc	1.0	0.977
	V$STAT5A.01	+266	(-)	aaccTTCCccgaacgccag	1.0	0.904
	V$STAT5A.01	+571	(+)	ggacTTCCcaggactgcat	1.0	0.868
	V$STAT5A.01	+1109	(-)	ccaaTTCCcagagtcactt	1.0	0.861
	V$STAT5A.01	+1644	(-)	caatTTCCtagtatgaaat	1.0	0.868
	V$STAT5A.01	+2580	(-)	tcctTTCCtaggatttctt	1.0	0.867
	V$STAT5A.01	+3669	(-)	acttTTCCaaaaattgggt	1.0	0.843
	V$STAT5A.01	+4108	(+)	tgcaTTCCaagaccccctg	1.0	0.865
	V$STAT5B.01	+329	(-)	aggctccgcAGAAggtggc	1.0	0.926
	V$STAT5B.01	+1175	(-)	ttcttccccAGAAtcctag	1.0	0.942

	V$STAT5B.01	+1865	(-)	cttctccccAGAAtctgga	1.0	0.938
	V$STAT5B.01	+7144	(-)	ttcaaacccAGAAagctta	1.0	0.935
	V$STAT6.01	+2813	(-)	tgggTTCCcaggaacactt	1.0	0.960
V$STEM	V$OCT3_4.01	+2615	(+)	caacttaGCATcactgaat	1.0	0.844
	V$OCT3_4.01	+5023	(-)	ttcagcaGCATtgctggct	1.0	0.825
	V$OCT3_4.02	+2066	(-)	cacctatGCATatttgtag	1.0	0.924
	V$OCT3_4.02	+2069	(+)	caaatatGCATaggtgctt	1.0	0.897
	V$OCT3_4.02	+5416	(-)	ctcctctGCATgtgcctcc	1.0	0.919
	V$OCT3_4.02	+5993	(-)	gatttctGCATtggggatg	1.0	0.901
	V$OCT3_4.02	+6033	(+)	acgttttGCATgcctgcat	1.0	0.910
	V$OCT3_4.02	+7652	(-)	atgatctGCATggtcaaag	1.0	0.943
	V$OSNT.01	+5660	(-)	cacctcaGCATcccaaagt	1.0	0.854
	V$OSNT.01	+6618	(+)	atatcttGCATttgaatca	1.0	0.807
V$TAIP	V$CSRNP1.01	+3139	(+)	AGAGtgg	1.0	1.000
V$TALE	V$MEIS1.01	+4013	(-)	aagtcatcTGTCattgg	1.0	0.969
	V$MEIS1.01	+5515	(+)	gacttcccTGTCaagct	1.0	0.970
	V$TGIF.01	+5085	(+)	acctgaagtGTCAgtgg	1.0	1.000
	V$TGIF.01	+7334	(-)	catacctgtGTCAaaac	1.0	1.000
	V$TGIF.01	+7477	(+)	tgctgtgctGTCAaata	1.0	1.000
V$TCFF	V$TCF11.01	+3722	(-)	GTCAttt	1.0	1.000
	V$TCF11.01	+4918	(-)	GTCAttt	1.0	1.000
V$TEAF	V$TEAD.01	+1093	(-)	aaaCATTccgcag	1.0	0.945
	V$TEAD.01	+3957	(-)	gctCATTctacac	1.0	0.907
	V$TEAD4.01	+4107	(+)	atgcATTCcaaga	1.0	0.945
V$THAP	V$THAP1.01	+840	(-)	agtctgGGCAg	1.0	0.919
	V$THAP1.01	+5000	(+)	agttctGGCAg	1.0	0.913
	V$THAP1.01	+5530	(-)	actgctGGCAg	1.0	0.933
V$WHNF	V$WHN.01	+1022	(+)	gggACGCatgg	1.0	0.958
V$XBBF	V$RFX4.02	+1931	(+)	tgcctcactaaGTTAcgta	1.0	0.899
	V$RFX4.03	+1125	(+)	tggGTTGctgcgcaatgag	1.0	0.873
	V$RFX5.01	+731	(-)	ctgtgggatcAGCAaccag	1.0	0.945
	V$RFX5.01	+1125	(-)	ctcattgcgcAGCAaccca	1.0	0.938
	V$RFX5.01	+1801	(+)	gggggacttaAGCAacttc	1.0	0.941
	V$RFX5.01	+5357	(+)	cccctgcagtAGCAactta	1.0	0.944
	V$RFX5.01	+7755	(+)	atagcagggcAGCAactat	1.0	0.937
V$YBXF	V$YB1.01	+4754	(+)	tagatTGGCggtc	1.0	0.881
V$YY1F	V$YY1.02	+740	(-)	caagcaCCATttctgtgggatc	1.0	0.969
	V$YY1.02	+2634	(-)	gaaagcCCATttgccaaagacaa	1.0	0.941
	V$YY1.02	+5543	(-)	ccatgcCCATtttccaacctagg	1.0	0.978
	V$YY1.02	+5597	(+)	gaatgaCCATgttcaaaaagaga	1.0	0.966
	V$YY1.02	+6667	(+)	ccgatgCCATttctaaaagtgat	1.0	0.940
	V$YY1.02	+7365	(-)	gataccCCATtttccatgatgtg	1.0	0.959
	V$YY1.03	+4031	(+)	gggccaCCATgttatctctctga	1.0	0.855
	V$YY2.01	+1518	(+)	gtccctCCATgttggtcaggctg	1.0	0.964
	V$YY2.01	+2918	(-)	tacagaCCATtttaatcggctgt	1.0	0.964
	V$YY2.01	+3376	(+)	gtggtgCCATcttggctcactgc	1.0	0.967
	V$YY2.01	+3724	(-)	agaatgCCATtttgtatagtcat	1.0	0.965
	V$YY2.01	+6509	(+)	gttttgCCATattgcccaggcta	1.0	0.970
	V$YY2.02	+853	(-)	cacccaCCATttcctgggcacag	1.0	0.857
	V$YY2.02	+1181	(-)	cttctcCCATttcttccccagaa	1.0	0.887
	V$YY2.02	+7525	(+)	tgtcacCCATtaactatccccac	1.0	0.887
V$ZBED	V$ZBED1.02	+4414	(-)	gTGTCtcgacaca	1.0	0.893
	V$ZBED1.02	+4415	(+)	gTGTCgagacaca	1.0	0.935
V$ZF02	V$ZBP89.01	+549	(-)	ccttcgctCCCCcgcggccgtga	1.0	0.945
	V$ZBTB7.01	+1789	(-)	ttaagtCCCCcaaagatcaagga	1.0	0.922
	V$ZBTB7.01	+2498	(-)	atcttcCCCCctacggaaaagct	1.0	0.916
	V$ZBTB7.03	+460	(-)	gcgagCCCCtccgcctcccctcg	1.0	0.918
	V$ZBTB7.03	+828	(+)	aaatgCCCCtccctgcccagact	1.0	0.930
	V$ZKSCAN3.01	+1492	(-)	cccccCCCCaccaccaccaccac	1.0	1.000
	V$ZKSCAN3.01	+3099	(-)	gtgaaCCCCaccctggtgtttcc	1.0	1.000
	V$ZKSCAN3.01	+7537	(+)	actatCCCCacctccctcccacc	1.0	1.000
	V$ZNF219.01	+546	(-)	tcgctCCCCcgcggccgtgatct	1.0	0.937
	V$ZNF219.01	+1496	(-)	cccccCCCCccccaccaccacca	1.0	0.974
	V$ZNF219.01	+1499	(-)	ggaccCCCCccccccccaccacca	1.0	0.997
	V$ZNF300.01	+429	(+)	ctggtcaCCCCagtcccaccagg	1.0	0.994
	V$ZNF300.01	+1863	(-)	aacttctCCCCagaatctggact	1.0	0.994
V$ZF03	V$ZNF217.01	+947	(+)	GAATtatcgggtg	1.0	0.904
V$ZF04	V$ZID.01	+519	(-)	ggGCTCcaacccc	1.0	0.886

	V$ZID.01	+3339	(-)	agGCTCcatctca	1.0	0.897
	V$ZID.01	+7616	(+)	ggGCTCaattgtt	1.0	0.864
V$ZF05	V$ZFP410.01	+5659	(+)	cactttgGGATgctg	1.0	0.881
	V$ZFP410.01	+7838	(+)	gagtaggGGATgggg	1.0	0.868
V$ZF06	V$ZBTB7.02	+6925	(+)	ccaGACCacccct	1.0	0.965
V$ZF07	V$ZNF263.01	+460	(-)	ctccgcCTCCcctcg	1.0	0.921
	V$ZNF263.01	+466	(-)	gagcccCTCCgcctc	1.0	0.943
	V$ZNF263.01	+6818	(+)	gctgccCTCCtcccc	1.0	0.961
	V$ZNF263.02	+6821	(+)	gccctcCTCCcctca	1.0	0.987
V$ZF10	V$PRDM14.01	+505	(-)	caaTTAGagaaactg	1.0	0.876
	V$PRDM14.01	+2175	(-)	ggtTTAGagtctggc	1.0	0.861
	V$PRDM14.01	+6112	(+)	agaTTAGggatgctt	1.0	0.853
V$ZF12	V$ZFP652.01	+3900	(-)	ttgaaagtgTTAAtt	1.0	0.913
V$ZF15	V$ZSCAN10.01	+372	(+)	tccgccgccTGCGctct	1.0	0.888
	V$ZSCAN10.01	+1606	(+)	gtgggccacTGCGcctg	1.0	0.804
	V$ZSCAN10.01	+7694	(+)	ataggagccTGCGcagt	1.0	0.894
V$ZF35	V$ZNF35.01	+2510	(+)	ggggggAAGAtca	1.0	0.974
	V$ZNF35.01	+4895	(-)	ggcgggAATAttg	1.0	0.976
	V$ZNF35.01	+5166	(-)	cccagcAATAacg	1.0	0.963
V$ZF57	V$ZFP57.01	+316	(-)	ccgTGCCgccccg	1.0	0.953
	V$ZFP57.01	+871	(-)	tttTGCCacaccc	1.0	0.843
V$ZF5F	V$ZF5.01	+161	(-)	ccgcgcGCGCcccgg	1.0	0.965
	V$ZF5.01	+162	(+)	cggggcGCGCgcgga	1.0	0.969
	V$ZF5.01	+164	(+)	gggcgcGCGCggaga	1.0	0.967
	V$ZF5.01	+3458	(-)	tggtggGCGCctgta	1.0	0.986
	V$ZF5.02	+119	(-)	cggacCGCGccccaa	1.0	0.838
	V$ZF5.02	+163	(-)	ctccgCGCGcgcccc	1.0	0.930
	V$ZF5.03	+53	(-)	cccCGCGcagctcac	1.0	0.880
	V$ZF5.03	+60	(-)	cccCGCGccccgcgc	1.0	0.879
V$ZFHX	V$AREB6.01	+2777	(-)	ccctgACCTgtgc	1.0	0.933
	V$AREB6.01	+7340	(-)	tgcatACCTgtgt	1.0	0.975
	V$AREB6.02	+6767	(-)	ggatCACCtgtgc	1.0	0.980
	V$AREB6.04	+1045	(+)	cttagGTTTcagc	1.0	0.981
	V$AREB6.04	+2669	(+)	tctgtGTTTcagc	1.0	0.982
	V$AREB6.04	+4452	(-)	ccactGTTTcaac	1.0	0.997
	V$AREB6.04	+5375	(+)	atacgGTTTctta	1.0	0.991
	V$AREB6.04	+5586	(+)	aatagGTTTcaga	1.0	0.981
	V$AREB6.04	+6170	(-)	gaagtGTTTctga	1.0	0.982
	V$DELTAEF1.01	+7007	(+)	ttttcACCTtagt	1.0	0.994
	V$DELTAEF1.02	+4930	(-)	ctcacACCTgtaa	1.0	0.985
	V$DELTAEF1.02	+6700	(-)	tgcccACCTgtga	1.0	1.000
	V$SIP1.01	+1142	(+)	aggacACCTggac	1.0	0.985
	V$SIP1.01	+3472	(+)	accacACCTggct	1.0	0.988
	V$SIP1.01	+4945	(+)	accacACCTggcc	1.0	0.988
V$ZICF	V$ZIC2.02	+7472	(-)	cagcaCAGCagggtg	1.0	0.983
	V$ZIC3.01	+605	(+)	acccaCAGCttggct	1.0	0.881
	V$ZIC3.03	+450	(-)	cctcgCAGCgggacc	1.0	0.971
	V$ZIC3.03	+700	(-)	ggccgCAGCtggtcc	1.0	0.931
V$ZTRE	V$ZTRE.03	+834	(+)	ccCTCCctgcccagact	1.0	0.995
	V$ZTRE.03	+7550	(+)	ccCTCCcaccctccctc	1.0	0.987
	V$ZTRE.03	+7558	(+)	ccCTCCctcactacctt	1.0	0.988
	V$ZTRE.04	+826	(-)	cagGGAGgggcatttga	1.0	0.998
	V$ZTRE.04	+7542	(-)	gtgGGAGggaggtgggg	1.0	0.985
	V$ZTRE.04	+7550	(-)	gagGGAGggtgggaggg	1.0	0.984

Intron 2

O$VTBP	O$LTATA.01	+8505	(+)	tgcTATAagtagaggag	1.0	0.878
	O$VTATA.02	+8143	(-)	aggcaTAAAacacttac	1.0	0.897
O$XCPE	O$XCPE1.01	+8383	(+)	ggGCGGaaggg	1.0	0.806
V$AARF	V$AARE.01	+8490	(-)	tTTTCatca	1.0	0.979
V$ABDB	V$HOXB9.02	+8146	(-)	aacaggcaTAAAacact	1.0	0.916
	V$HOXC9.01	+8664	(-)	gcatatcaTTAAacttt	1.0	0.882
V$AHRR	V$AHRARNT.02	+8233	(+)	ctctcccactGCGTggcttcccctg	1.0	0.835
V$AIRE	V$AIRE.01	+8645	(-)	acTTATtaatagttt	1.0	0.860
V$AP1F	V$AP1.02	+8347	(-)	ctgtGAGTgagtc	1.0	0.886
	V$FOSL1.01	+8343	(-)	gagtgAGTCccag	1.0	0.852

V$ATBF	V$ATBF1.01	+8540	(-)	gcattttcatAATTagc	1.0	0.841
V$BCDF	V$OTX2.01	+8606	(+)	atatgTAATcccaacat	1.0	0.963
	V$PCE1.01	+8766	(+)	tcccgTAATtgtgtatg	1.0	0.896
V$BCL6	V$BCL6.02	8629	(+)	ttagtctTAGAaactca	1.0	0.844
V$BRN5	V$BRN5.03	+8651	(+)	tTAATaagtggaaaaagtttaat	1.0	0.814
	V$BRN5.04	+8533	(+)	gacttgagctaATTAtgaaaatg	1.0	0.946
	V$BRN5.04	+8644	(-)	tttttccacttATTAatagtttg	1.0	0.872
	V$BRN5.04	+8662	(-)	acatgcatatcATTAaacttttt	1.0	0.844
V$BRNF	V$BRN2.01	+8258	(-)	ctCATTggacaaatgagtc	1.0	0.872
	V$BRN2.04	+8535	(+)	cttgagcTAATtatgaaaa	1.0	0.896
	V$BRN2.04	+8536	(-)	attttcaTAATtagctcaa	1.0	0.881
	V$BRN3.02	+8642	(-)	cacttatTAATagtttgag	1.0	0.890
	V$BRN3.03	+8645	(+)	aaactatTAATaagtggaa	1.0	0.940
	V$BRN3.03	+8663	(+)	aaaagttTAATgatatgca	1.0	0.837
	V$BRN4.01	+8540	(-)	ctgcattttcaTAATtagc	1.0	0.892
V$CAAT	V$NFY.04	+8265	(+)	ttgtCCAAtgagggc	1.0	0.947
V$CARE	V$CARF.01	+8333	(+)	agagcGAGGcc	1.0	1.000
V$CART	V$RHOX6.01	+8647	(+)	actatTAATaagtggaaaaag	1.0	0.871
	V$S8.01	+8532	(-)	tttcaTAATtagctcaagtca	1.0	0.997
	V$S8.01	+8537	(+)	tgagcTAATtatgaaaatgca	1.0	0.992
	V$S8.01	+8766	(+)	tcccgTAATtgtgtatgtctt	1.0	0.995
V$CDXF	V$CDX2.01	+8144	(+)	taagtgtTTTAtgcctgtt	1.0	0.885
V$CIZF	V$NMP4.01	+8294	(-)	ggAAAAacaag	1.0	0.981
	V$NMP4.01	+8493	(+)	tgAAAAagtat	1.0	0.977
	V$NMP4.01	+8597	(+)	tgAAAAagtat	1.0	0.977
	V$NMP4.01	+8660	(+)	ggAAAAagttt	1.0	0.997
V$CLOX	V$CDP.02	+8263	(+)	atttgtcCAATgagggcttgcaa	1.0	0.954
V$CREB	V$E4BP4.01	+8600	(+)	aaaagtatatGTAAtcccaac	1.0	0.847
V$DLXF	V$DLX1.01	+8538	(-)	gcattttcatAATTagctc	1.0	0.976
	V$DLX2.01	+8533	(+)	gacttgagctAATTatgaa	1.0	0.976
	V$DLX2.01	+8762	(+)	tctgtcccgtAATTgtgta	1.0	0.928
	V$DLX5.01	+8767	(-)	agacatacacAATTacggg	1.0	0.926
V$E2FF	V$E2F6.01	+8429	(+)	gagtaGCGGgaagtgcg	1.0	0.915
V$ETSF	V$ETS1.01	+8429	(+)	gagtagcgGGAAgtgcggtac	1.0	0.957
	V$SPI1.02	+8156	(-)	gatcaaggGGAAgaaacaggc	1.0	0.965
	V$SPI1.02	+8241	(-)	agtccaggGGAAgccacgcag	1.0	0.967
	V$SPI1.02	+8379	(+)	cccagggcGGAAgggagtgga	1.0	0.962
	V$SPIB.01	+8213	(-)	gtgaaaagGGAAagtcatggt	1.0	0.881
	V$SPIB.01	+8715	(-)	ggaaaataGGAAtatgtgatc	1.0	0.901
V$FKHD	V$FOXP1_ES.01	+8200	(-)	tggtaaaAACAaaaccc	1.0	1.000
	V$FOXP1_ES.01	+8290	(-)	ctggaaaAACAaggctc	1.0	1.000
	V$FOXP1_ES.01	+8470	(-)	tgagaaaAACAatcaca	1.0	1.000
	V$FREAC2.01	+8203	(-)	tcatggTAAAaacaaaa	1.0	0.847
V$GCMF	V$GCM1.01	+8186	(-)	cccaaCCCTcaacca	1.0	0.880
	V$GCM1.01	+8269	(-)	gcaagCCCTcattgg	1.0	0.918
V$GLIF	V$GLIS2.01	+8190	(-)	aaaaCCCCcaaccctca	1.0	0.861
V$HBOX	V$EMX2.01	+8646	(+)	aactatTAATaagtggaaa	1.0	0.839
	V$GSH1.01	+8614	(+)	tcccaacatcTAATtttag	1.0	0.861
	V$GSH1.01	+8761	(+)	ttctgtcccgTAATtgtgt	1.0	0.873
	V$GSH2.01	+8535	(-)	ttttcaTAATtagctcaag	1.0	0.955
	V$GSH2.02	+8536	(+)	ttgagctAATTatgaaaat	1.0	0.966
	V$VAX2.01	+8645	(-)	ttccacttATTAatagttt	1.0	0.877
V$HDBP	V$HDBP1_2.01	+8396	(-)	ctcctgCCGGcagagtcca	1.0	0.854
V$HEAT	V$HSF1.04	+8724	(+)	ttcctattttcctgaaaaTTCTgct	1.0	0.955
	V$HSF2.02	+8733	(-)	attctcaaaagcAGAAttttcagga	1.0	0.976
V$HNF1	V$HNF1.02	+8539	(+)	agcTAATtatgaaaatg	1.0	0.854
V$HNF6	V$OC2.01	+8466	(-)	aaaaacAATCacaaaca	1.0	0.839
V$HOMF	V$BARX2.01	+8642	(-)	cacttatTAATagtttgag	1.0	0.964
	V$BARX2.01	+8645	(+)	aaactatTAATaagtggaa	1.0	0.955
	V$BSX.01	+8535	(+)	cttgagctAATTatgaaaa	1.0	0.955
	V$BSX.01	+8536	(-)	attttcatAATTagctcaa	1.0	0.961
	V$HHEX.01	+8659	(+)	tggaaaaagttTAATgata	1.0	0.961
	V$HMX3.01	+8648	(+)	ctattaatAAGTggaaaaa	1.0	0.912
	V$HMX3.02	+8641	(+)	actcaaactaTTAAtaagt	1.0	0.920
	V$MSX.01	+8617	(+)	caacatcTAATtttagtct	1.0	0.989
	V$NOBOX.01	+8764	(+)	tgtcccgTAATtgtgtatg	1.0	0.951
	V$TLX1.01	+8480	(+)	tttctcaCGGTgatgaaaa	1.0	0.855

V$HOXC	V$PBX1.01	+8468	(+)	tttgtGATTgtttttct	1.0	0.807
V$HOXF	V$HOXA3.02	8537	(+)	tgagctaATTAtgaaaatg	1.0	0.969
	V$HOXB6.01	8766	(+)	tcccgtAATTgtgtatgtc	1.0	0.853
	V$HOXD3.01	8534	(-)	tttcatAATTagctcaagt	1.0	0.972
	V$HOXD8.01	8643	(+)	tcaaactATTAataagtgg	1.0	0.851
	V$HOXD8.01	8644	(-)	tccacttATTAatagtttg	1.0	0.876
V$HUB1	V$ZNF282.01	+8221	(+)	TTTCccttttcactc	1.0	0.824
V$IKRS	V$IK2.01	+8313	(+)	atttGGGAagaaa	1.0	0.992
	V$IK2.01	+8609	(-)	tgttGGGAttaca	1.0	0.980
V$IRFF	V$IRF1.01	+8584	(+)	ggcaagaataaagtGAAAaagtata	1.0	0.895
	V$IRF2.01	+8221	(-)	cgcagtgggagagtGAAAagggaaa	1.0	0.854
	V$IRF7.01	+8630	(+)	tagtcttaGAAActcaaactattaa	1.0	0.879
	V$ISRE.02	+8214	(-)	ggagagtgaaaaggGAAAgtcatgg	1.0	0.909
V$IRXF	V$IRX3.01	+8676	(+)	tatgCATGtaatg	1.0	0.872
	V$IRX6.01	+8675	(-)	attaCATGcatat	1.0	0.862
V$KLFS	V$BKLF.02	+8552	(-)	gatcagGGAGggctgcatt	1.0	0.936
	V$BTEB3.01	+8382	(+)	agggcggaagGGAGtggac	1.0	0.961
	V$KLF12.01	+8230	(-)	ccacgcaGTGGgagagtga	1.0	0.925
	V$KLF7.02	+8378	(+)	gcccagGGCGgaagggagt	1.0	0.896
V$LEFF	V$LEF1.03	+8164	(-)	agctgaTCAAggggaag	1.0	0.844
V$LHXF	V$ISL2.01	+8639	(+)	aaactcaaactATTAataagtgg	1.0	0.895
	V$ISL2.01	+8644	(-)	tttttccacttATTAatagtttg	1.0	0.881
	V$LHX3.02	+8529	(+)	tcctgacttgagcTAATtatgaa	1.0	0.822
	V$LHX3.02	+8611	(+)	taatcccaacatcTAATtttagt	1.0	0.849
	V$LHX6.01	+8533	(+)	gacttgagcTAATtatgaaaatg	1.0	0.931
	V$LHX6.01	+8534	(-)	gcattttcaTAATtagctcaagt	1.0	0.890
	V$LHX6.01	+8643	(+)	tcaaactatTAATaagtggaaaa	1.0	0.851
V$LTSM	V$LTSM.02	+8463	(-)	caatcacaaaCATCa	1.0	0.962
V$MEF2	V$SL1.01	+8500	(+)	gtatgtgCTATaagtagaggagc	1.0	0.905
	V$SL1.01	+8641	(+)	actcaaaCTATtaataagtggaa	1.0	0.945
V$MOKF	V$MOK2.02	+8280	(+)	ttgcaagctggagCCTTgttt	1.0	0.991
	V$MOK2.02	+8371	(-)	cttccgccctgggCCTTttgg	1.0	1.000
V$MYT1	V$MYT1.02	+8662	(+)	aaaAAGTttaatg	1.0	0.992
V$MZF1	V$MZF1.02	+8162	(-)	aaGGGGaagaa	1.0	1.000
V$NFAT	V$NFAT.01	+8654	(+)	ataagtGGAAaaagtttaa	1.0	0.972
	V$NFAT5.02	+8290	(-)	tgctGGAAaaacaaggctc	1.0	0.884
	V$NFAT5.02	+8656	(+)	aagtGGAAaaagtttaatg	1.0	0.876
V$NKX1	V$NKX12.01	+8536	(-)	tttcatAATTagctcaa	1.0	0.911
	V$NKX12.01	+8537	(+)	tgagctAATTatgaaaa	1.0	0.915
V$NKX6	V$NKX61.02	+8644	(-)	cttaTTAAtagtttg	1.0	0.923
	V$NKX63.01	+8647	(+)	actatTAATaagtgg	1.0	0.916
	V$NKX63.01	+8665	(+)	aagttTAATgatatg	1.0	0.870
V$NKXH	V$NKX25.02	+8534	(-)	tttcaTAATtagctcaagt	1.0	0.951
	V$NKX25.02	+8766	(+)	tcccgTAATtgtgtatgtc	1.0	0.939
	V$NKX25.05	+8343	(-)	tactgTGAGtgagtcccag	1.0	0.972
	V$NKX31.01	+8505	(+)	tgctatAAGTagaggagcg	1.0	0.924
	V$NKX31.01	+8596	(+)	gtgaaaAAGTatatgtaat	1.0	0.859
	V$NKX32.01	+8650	(+)	attaataAGTGgaaaaagt	1.0	0.979
V$NOLF	V$EBF1.01	+8245	(+)	gtggctTCCCctggactcatttg	1.0	0.884
V$OCT1	V$OCT1.03	+8536	(+)	ttgagctaATTAtga	1.0	0.934
	V$OCT1.03	+8539	(-)	ttttcataATTAgct	1.0	0.947
	V$OCT1.04	+8544	(+)	atTATGaaaatgcag	1.0	0.905
	V$POU3F3.01	+8671	(-)	tacatGCATatcatt	1.0	0.893
	V$POU3F3.01	+8674	(+)	gatatGCATgtaatg	1.0	0.849
V$P53F	V$P53.05	+8277	(-)	aaaaCAAGgctccagcttgcaagcc	1.0	0.834
V$PARF	V$DBP.01	+8540	(+)	gctaaTTATgaaaatgc	1.0	0.864
	V$VBP.01	+8601	(+)	aaagtatatGTAAtccc	1.0	0.889
V$PAX2	V$PAX2.01	+8628	(+)	tttagtcttagaaactcAAACta	1.0	0.810
V$PAX3	V$PAX3.03	+8205	(-)	aaagtCATGgtaaaaacaa	1.0	0.987
V$PAX6	V$PAX6.02	+8371	(+)	ccaaaaggcCCAGggcgga	1.0	0.887
V$PAXH	V$PAX4.02	+8537	(-)	ttcatAATTagctca	1.0	0.937
	V$PAX4.02	+8538	(+)	gagctAATTatgaaa	1.0	0.911
V$PDX1	V$IPF1.01	+8533	(+)	gacttgagcTAATtatgaa	1.0	0.927
	V$IPF1.01	+8538	(-)	gcattttcaTAATtagctc	1.0	0.960
V$PERO	V$PPARG.02	+8652	(+)	taataagtggaaAAAGtttaatg	1.0	0.891
V$PIT1	V$PIT1.02	+8646	(+)	aactaTTAAtaagtg	1.0	0.813
V$PLZF	V$PLZF.01	+8444	(+)	cggTACAgtctgggc	1.0	0.895

V$PRDF	V$BLIMP1.01	+8220	(-)	ggagagtGAAAagggaaag	1.0	0.897
	V$BLIMP1.01	+8358	(-)	ttttggtGAAAgggttact	1.0	0.829
	V$PRDM1.01	+8591	(+)	ataaagtGAAAaagtatat	1.0	0.839
	V$PRDM1.02	+8213	(-)	gaaaaggGAAAgtcatggt	1.0	0.961
V$RREB	V$RREB1.01	+8187	(-)	cCCCAaccctcaacc	1.0	0.848
V$RUSH	V$SMARCA3.02	+8495	(-)	acatACTTttt	1.0	0.993
	V$SMARCA3.02	+8599	(-)	atatACTTttt	1.0	0.993
V$SATB	V$SATB1.01	+8641	(-)	attAATAgtttgagt	1.0	0.947
V$SMAD	V$GC_SBE.01	+8287	(-)	caaggCTCCag	1.0	1.000
	V$SMAD3.01	+8448	(+)	acaGTCTgggc	1.0	0.994
V$SORY	V$SOX3.01	+8251	(-)	attggaCAAAtgagtccagggga	1.0	0.955
	V$SOX5.01	+8461	(-)	gaaaaaCAATcacaaacatcaga	1.0	0.984
	V$SRY.05	+8667	(+)	gtttAATGatatgcatgtaatgc	1.0	0.836
V$STAT	V$STAT1.01	+8428	(+)	tgagtagcgGGAAgtgcgg	1.0	0.803
	V$STAT1.02	+8726	(-)	gaattttcaGGAAaatagg	1.0	0.915
	V$STAT3.01	+8728	(+)	tattTTCCtgaaaattctg	1.0	0.802
V$STEM	V$OCT3_4.01	+8678	(-)	ggcaaagGCATtacatgca	1.0	0.828
	V$OCT3_4.02	+8672	(+)	atgatatGCATgtaatgcc	1.0	0.912
V$TAIP	V$CSRNP1.01	+8230	(-)	AGAGtga	1.0	1.000
V$THAP	V$THAP1.01	+8450	(+)	agtctgGGCAt	1.0	0.917
V$XBBF	V$RFX4.03	+8574	(+)	gacGTTGggaggcaagaat	1.0	0.804
	V$RFX5.01	+8295	(+)	ttgtttttccAGCAgcaga	1.0	0.912
V$YY1F	V$YY1.03	+8689	(+)	cctttgCCATattcctctccttc	1.0	0.830
V$ZF02	V$ZNF219.01	+8185	(-)	caaaaCCCCcaaccctcaaccat	1.0	0.928
V$ZF03	V$ZNF217.01	+8754	(+)	GAATgctttctgt	1.0	0.923
V$ZF04	V$ZID.01	+8283	(-)	agGCTCcagcttg	1.0	0.870
V$ZF12	V$ZFP652.01	+8660	(+)	ggaaaaagtTTAAtg	1.0	0.803
	V$ZNF652.02	+8357	(-)	gtgaaaGGGTtactg	1.0	0.882
V$ZFHX	V$AREB6.04	+8155	(+)	tgcctGTTTcttc	1.0	0.997
V$ZFXY	V$ZFX.01	+8338	(+)	gaGGCCtggga	1.0	0.989
V$ZTRE	V$ZTRE.03	+8381	(-)	caCTCCcttccgccctg	1.0	0.965
	V$ZTRE.03	+8559	(+)	ccCTCCctgatctgaga	1.0	0.995
	V$ZTRE.04	+8389	(+)	aagGGAGtggactctgc	1.0	0.961
	V$ZTRE.04	+8551	(-)	cagGGAGggctgcattt	1.0	0.998

Intron 3

O$INRE	O$DINR.01	+10353	(-)	tcTCAGttgtt	1.0	0.987
O$PTBP	O$PTATA.01	+9282	(+)	ttagTATAaacagaa	1.0	0.921
	O$PTATA.01	+9335	(+)	tgaaTATAaatcaag	1.0	0.916
	O$PTATA.02	+9712	(-)	agatTATAaatctgg	1.0	0.905
O$VTBP	O$LTATA.01	+9449	(+)	cgcTATAagatactgtt	1.0	0.838
	O$LTATA.01	+9509	(+)	tgaTATAatttgtccag	1.0	0.829
	O$MTATA.01	+9174	(-)	agcttTAAAgctcaggg	1.0	0.912
	O$MTATA.01	+9179	(+)	agcttTAAAgctgctgc	1.0	0.873
	O$VTATA.01	+9283	(+)	tagtaTAAAcagaaggt	1.0	0.941
	O$VTATA.01	+9336	(+)	gaataTAAAtcaagatt	1.0	0.928
	O$VTATA.01	+9709	(-)	gattaTAAAtctggaaa	1.0	0.928
V$ABDB	V$HOXA10.01	9741	(+)	acatataaTAAAtatga	1.0	0.873
	V$HOXA9.02	10450	(+)	agaggccaTAAAtaaat	1.0	0.903
	V$HOXC10.01	10426	(+)	ggaagtctTAAAaaggg	1.0	0.932
	V$HOXC13.01	10502	(+)	gttggtcaaTAAAcatt	1.0	0.922
	V$HOXC9.01	9459	(-)	tggggttaTTAAcagta	1.0	0.915
	V$HOXD13.01	9315	(+)	ggcactgaTAAAactgc	1.0	0.910
V$AHRR	V$AHRARNT.03	+9605	(-)	attttaatttGCGTgaaaattcaac	1.0	0.965
V$AIRE	V$AIRE.01	+9458	(-)	ggTTATtaacagtat	1.0	0.817
V$AP1F	V$AP1.02	+10984	(-)	taagGAGTcatgt	1.0	0.876
	V$FOSL2.01	+10008	(+)	ttaggAGTCatag	1.0	0.857
	V$JUNB.01	+9902	(+)	tggttaGTCAgtt	1.0	0.917
V$AP1R	V$MAFA.01	+10149	(+)	tttgaggcactagcAGCAaatct	1.0	0.934
	V$MAFK.01	+9320	(-)	tatattcataAGCAgttttatca	1.0	0.824
	V$TCF11MAFG.01	+9897	(+)	agagatggttaGTCAgttgcaga	1.0	0.848
V$AP2F	V$TCFAP2B.01	+10653	(+)	cttGCCCccaggaag	1.0	0.865
	V$TCFAP2B.01	+10866	(+)	ttgGCCCctgggcaa	1.0	0.940
	V$TCFAP2B.01	+10867	(-)	tttGCCCaggggcca	1.0	0.916
V$ARID	V$ARID5A.01	+9255	(+)	tgttcATATtgagcttattgt	1.0	0.876
	V$ARID5A.01	+10207	(-)	tccaaATATttctttctagt	1.0	0.945

	V$ARID5A.01	+10214	(+)	agaaaATATttggataggctg	1.0	0.942
	V$ARID5A.01	+10549	(-)	actcaATATttatggaatgaa	1.0	0.960
	V$ARID5A.01	+10556	(+)	cataaATATtgagtatctgcc	1.0	0.967
	V$BRIGHT.01	+9617	(+)	cgcaaATTAaaatatgtcata	1.0	0.939
V$ATBF	V$ATBF1.01	+10824	(+)	ctttcttttcAATTatt	1.0	0.820
V$BARB	V$BARBIE.01	+9174	(-)	ctttAAAGctcaggg	1.0	0.898
	V$BARBIE.01	+10393	(+)	ataaAAAGgtttggg	1.0	0.919
V$BCDF	V$CRX.03	+9775	(-)	tggtctaATCCcatact	1.0	0.986
	V$PCE1.01	+10827	(-)	acaaaTAATtgaaaaga	1.0	0.913
	V$PTX1.02	+10250	(+)	gcctgtAATCccaccac	1.0	0.902
V$BEDF	V$ZBED4.02	+10098	(+)	aactgatGGGGgagg	1.0	0.936
	V$ZBED4.02	+10104	(+)	tgggggaGGGGgtag	1.0	0.926
V$BHLH	V$BHLHA15.01	+10572	(+)	ctgccaTATGcaa	1.0	0.927
	V$BHLHA15.01	+10613	(-)	ttctcaTATGctt	1.0	0.887
	V$MESP1_2.01	+10573	(-)	tttgCATAtggca	1.0	0.925
	V$MESP1_2.01	+10612	(+)	aaagCATAtgaga	1.0	0.961
	V$MESP1_2.01	+10999	(+)	gacaCATAtgtac	1.0	0.954
	V$MESP1_2.01	+11000	(-)	agtaCATAtgtgt	1.0	0.924
V$BPTF	V$FAC1.01	+10922	(-)	tgaaaAACAca	1.0	0.964
V$BRAC	V$EOMES.03	+10318	(-)	ggagtgcagtggTGTGatcatagctcact	1.0	0.987
	V$TBOX.01	+9466	(-)	catttccccatGTGTgaaatggggttatt	1.0	0.931
	V$TBOX.01	+10123	(-)	aaattcaacaaGTGTgaactagaatggtc	1.0	0.930
	V$TBOX.01	+10918	(-)	actattaatatGTGTgaaaaacacataac	1.0	0.948
V$BRN5	V$BRN5.04	+9291	(-)	tcaaaatactcATTAccttctgt	1.0	0.931
	V$BRN5.04	+9989	(+)	agaatctactgATTAtgtcttag	1.0	0.891
	V$BRN5.04	+10797	(-)	cagtaaatctcATTActacggcc	1.0	0.944
V$BRNF	V$BRN3.01	+9740	(-)	ctcatatttATTAtatgtt	1.0	0.815
	V$BRN3.01	+10826	(+)	ttcttttcaATTAtttgta	1.0	0.807
	V$BRN3.02	+10930	(-)	tcactatTAATatgtgtga	1.0	0.909
	V$BRN3.03	+10798	(+)	gccgtagTAATgagattta	1.0	0.834
	V$BRN3.03	+10933	(+)	cacatatTAATagtgatgg	1.0	0.837
	V$BRN4.01	+9414	(+)	atctacatggaTAATctca	1.0	0.896
	V$TST1.01	+11006	(-)	tgggaATTAacaagtacat	1.0	0.983
V$BTBF	V$KAISO.01	+10158	(-)	tttgCTGCtag	1.0	0.924
V$CART	V$ALX4.01	+9615	(+)	cacgcaAATTaaaatatgtca	1.0	0.832
	V$CART1.01	+9420	(+)	atggaTAATctcatttaaccc	1.0	0.868
	V$CART1.01	+9430	(-)	cgtggTAATggggttaaatga	1.0	0.863
	V$CART1.01	+10800	(+)	cgtagTAATgagatttactga	1.0	0.903
	V$ISX.01	+10777	(-)	catcccAATTgtactcattc	1.0	0.917
	V$ISX.01	+10782	(+)	agtacaAATTgggatggccgt	1.0	0.916
	V$S8.01	+10823	(-)	acaaaTAATtgaaaagaaagc	1.0	1.000
	V$XVENT2.01	+9460	(+)	actgtTAATaaccccatttca	1.0	0.849
V$CDXF	V$CDX1.01	+10386	(-)	aaaccttTTTAttttttga	1.0	0.941
	V$CDX1.01	+10426	(-)	ttcccttTTTAagacttcc	1.0	0.965
	V$CDX2.01	+9710	(+)	ttccagaTTTAtaatctag	1.0	0.874
	V$CDX2.01	+9729	(-)	tatatgtTTTAtcatccaa	1.0	0.879
	V$CDX2.02	+9741	(-)	cctcatatTTATtatatgt	1.0	0.866
	V$CDX2.03	+10450	(-)	ctatttatTTATggcctct	1.0	0.965
	V$CDX2.03	+10503	(-)	acaaatgtTTATtgaccaa	1.0	0.990
	V$CDX2.03	+10550	(-)	ctcaatatTTATggaatga	1.0	0.957
V$CEBP	V$CEBPA.01	+9527	(+)	gtcattgaGCAAaca	1.0	0.959
	V$CEBPB.01	+9481	(+)	cacatgggGAAAtgg	1.0	0.947
	V$CEBPB.01	+9489	(+)	gaaatgggGCAAtag	1.0	0.947
	V$CEBPB.02	+9473	(-)	ccatgTGTGaaatgg	1.0	0.977
	V$CEBPB.02	+9934	(+)	aaggcTGTGgaaagg	1.0	0.921
	V$CEBPB.02	+10925	(-)	atatgTGTGaaaaac	1.0	0.975
	V$CEBPD.01	+10514	(+)	acattTGTGgaatga	1.0	0.973
	V$CEBPE.02	+10869	(+)	gcccctggGCAAagt	1.0	0.973
V$CIZF	V$NMP4.01	+10965	(-)	ggAAAAaacga	1.0	0.982
V$CP2F	V$TCFCP2L1.01	+9666	(-)	atCTGGatcccccatggag	1.0	0.901
V$CREB	V$CREB2.01	+9830	(-)	ctaaacTGACctaaaaggaga	1.0	0.906
V$DLXF	V$DLX2.01	+10824	(+)	ctttcttttcAATTatttg	1.0	0.934
	V$DLX3.01	+10829	(-)	atgtacaaaTAATtgaaaa	1.0	0.950
	V$DLX3.02	+9578	(+)	ttctaaagaTAATtttgtg	1.0	0.936
	V$DLX5.01	+11007	(+)	tgtacttgttAATTcccag	1.0	0.914
V$DMRT	V$DMRT1.01	+10368	(+)	gattttgagaccTTGTctca	1.0	0.825
V$E2FF	V$E2F.02	+9608	(-)	aatttgcgtGAAAattc	1.0	0.910
	V$E2F.02	+10861	(-)	cccaggggcCAAAgaga	1.0	0.849

V$EBOX	V$USF1.01	10286	(+)	aggaTCACttgagtcca	1.0	0.956
V$EGRF	V$CKROX.01	+10103	(+)	atggGGGAgggggtaggga	1.0	1.000
	V$CKROX.01	+10266	(+)	ctgtGGGAggctgaggcag	1.0	0.885
	V$EGR2.02	+9868	(+)	gagTGGGtggcattgagag	1.0	0.948
	V$EGR2.02	+10172	(+)	aggTGGGaggaagtacagg	1.0	0.953
	V$WT1.01	+9866	(+)	gggagTGGGtggcattgag	1.0	0.922
V$ESRR	V$ESRRA.01	+10372	(-)	atttttgagacAAGGtctcaaa	1.0	0.886
V$ETSF	V$ETV1.02	+9969	(+)	attacagaGGAAgaaccccca	1.0	0.964
	V$ETV1.02	+10655	(+)	tgcccccaGGAAgcagaaatat	1.0	0.991
	V$PEA3.01	+9696	(-)	tctggaaAGGAaggatcctgt	1.0	0.947
	V$SPI1.02	+10088	(+)	catgaaggGGAActgatgggg	1.0	0.980
	V$SPI1.02	+10172	(+)	aggtgggaGGAAgtacaggac	1.0	0.988
	V$SPI1.02	+10418	(+)	acccagggGGAAgtcttaaaa	1.0	0.990
	V$SPI1.03	+9480	(+)	acacatggGGAAatggggcaa	1.0	0.891
	V$SPI1.04	+9540	(+)	catgtagaGGAAccagggtaa	1.0	0.921
	V$SPIB.01	+10433	(+)	ttaaaaagGGAAagaaaagag	1.0	0.887
V$EVI1	V$EVI1.02	+10819	(-)	ttgaaAAGAaagccatc	1.0	0.838
	V$EVI1.05	+9450	(+)	gctataaGATActgtta	1.0	0.821
	V$EVI1.06	+10735	(+)	atcacaAGATctagctt	1.0	0.848
	V$MEL1.03	+10340	(-)	tgttgagGATGgagtgc	1.0	0.968
V$FAST	V$FAST1.01	+10505	(-)	acaaatgttTATTgacc	1.0	0.926
V$FKHD	V$FOXO1.01	+10533	(-)	acactgttAACActgtg	1.0	0.882
	V$FOXO1.01	+10534	(+)	acagtgttAACAgtgtt	1.0	0.889
	V$FOXO1.01	+10764	(+)	aaatagtcAACAtgaat	1.0	0.898
	V$FOXP1_ES.01	+9652	(+)	agtgtaaAACAtaactc	1.0	1.000
	V$FOXP1_ES.01	+9733	(+)	atgataaAACAtataat	1.0	1.000
	V$FOXP1_ES.01	+10918	(-)	tgtgaaaAACAcataac	1.0	1.000
	V$FREAC7.01	+10505	(+)	ggtcaaTAAAcatttgt	1.0	0.963
	V$HNF3.01	+9530	(+)	attgagcAAACatgtag	1.0	1.000
	V$HNF3B.01	+10210	(+)	agaaagaaAATAtttgg	1.0	0.945
	V$XFD1.01	+10456	(+)	cataaaTAAAtagagct	1.0	0.921
	V$XFD2.01	+9282	(+)	ttagtaTAAAcagaagg	1.0	0.975
	V$XFD2.01	+9743	(+)	atataaTAAAtatgagg	1.0	0.897
V$GATA	V$GATA.01	+9317	(+)	cactGATAaaact	1.0	0.987
	V$GATA1.02	+9554	(-)	accaGATTaccct	1.0	1.000
	V$GATA1.03	+9581	(+)	taaaGATAatttt	1.0	0.952
	V$GATA1.03	+9731	(+)	ggatGATAaaaca	1.0	0.951
	V$GATA1.06	+9453	(+)	ataaGATActgtt	1.0	0.961
	V$GATA2.02	+9506	(+)	aagtGATAtaatt	1.0	0.908
	V$GATA2.03	+10854	(-)	aagaGATAaggct	1.0	0.997
V$GCMF	V$GCM1.03	+9667	(-)	tggatCCCCcatgga	1.0	0.855
	V$GCM1.03	+10100	(-)	cccctCCCCcatcag	1.0	0.870
V$GFI1	V$GFI1.02	+10360	(-)	aaaAATCcgagtctc	1.0	0.903
V$GLIF	V$GLI1.01	+9979	(+)	aagaaccCCCAgaatct	1.0	0.875
	V$GLI3.02	+9865	(-)	aatgCCACccactcccc	1.0	0.886
V$GREF	V$GRE.03	+9361	(-)	aataaccaaaaTGTTctcc	1.0	0.910
	V$GRE.03	+9945	(-)	ttacatctgccTGTTcctt	1.0	0.883
V$GRHL	V$GRHL2.01	+9557	(-)	aaAACCagattac	1.0	0.896
	V$GRHL3.01	+9558	(+)	taatctGGTTttg	1.0	0.848
V$GUCE	V$TFII-IR4.01	+9779	(+)	tggGATTagac	1.0	1.000
V$HAML	V$AML1.02	+10234	(+)	gggtGTGGtggctca	1.0	0.960
	V$AML2.01	+9274	(+)	gtgTGTGgttagtat	1.0	0.980
	V$AML3.01	+10620	(-)	gcttGTGGgttctca	1.0	0.843
V$HAND	V$TAL1ALPHAE47.01	+10893	(+)	gtctggaCAGAtggcatatac	1.0	0.919
	V$TAL1BETAITF2.01	+9285	(+)	gtataaaCAGAaggtaatgag	1.0	0.852
V$HBOX	V$EN1.02	+9241	(+)	gtcaaaaAATTaagtgttc	1.0	0.873
	V$EN2.01	+9507	(-)	ctggacaAATTatatcact	1.0	0.873
	V$GSH1.01	+9347	(+)	aagattgtagTAATggaga	1.0	0.863
	V$GSH1.01	+9577	(+)	cttctaaagaTAATtttgt	1.0	0.859
	V$GSH1.01	+10830	(-)	gatgtacaaaTAATtgaaa	1.0	0.857
	V$GSH1.01	+10930	(+)	tcacacatatTAATagtga	1.0	0.890
	V$GSH2.01	+9613	(-)	atatttTAATttgcgtgaa	1.0	0.964
	V$VAX2.01	+9292	(-)	aaatactcATTAccttctg	1.0	0.859
	V$VAX2.01	+9614	(+)	tcacgcaaATTAaaatatg	1.0	0.857
V$HEAT	V$HSF2.02	+10119	(-)	caagtgtgaactAGAAtggtcttcc	1.0	0.957
V$HICF	V$HIC1.01	+9121	(-)	gaaTGCCatctca	1.0	0.900
	V$HIC1.01	+9870	(-)	caaTGCCacccac	1.0	0.963
	V$HIC1.01	+10899	(-)	ataTGCCatctgt	1.0	0.895

V$HNF1	V$HMBOX.01	+11006	(+)	atgtacttGTTAattcc	1.0	0.910
	V$HNF1.01	+9903	(+)	gGTTAgtcagttgcaga	1.0	0.831
	V$HNF1.01	+10724	(+)	tGTTAatgttgatcaca	1.0	0.877
	V$HNF1.02	+9370	(-)	tgtTAATaataaccaaa	1.0	0.924
	V$HNF1.03	+9373	(+)	gGTTAttattaacactt	1.0	0.855
	V$HNF1.04	+10405	(+)	gggtgataGTTAaaccc	1.0	0.883
	V$HNF1.04	+10536	(-)	tgaacactGTTAacact	1.0	0.849
V$HNF6	V$HNF6.01	+9337	(+)	aatataaaTCAAgattg	1.0	0.857
	V$HNF6.01	+9394	(+)	agtacatgTCAAtagta	1.0	0.822
	V$HNF6.01	+10559	(-)	gcagatacTCAAtattt	1.0	0.875
	V$OC2.01	+9992	(-)	agacatAATCagtagat	1.0	0.898
V$HOMF	V$BARX2.01	+9434	(-)	agcgtggTAATggggttaa	1.0	0.958
	V$BARX2.01	+10720	(+)	aaaatgtTAATgttgatca	1.0	0.955
	V$BARX2.01	+10933	(+)	cacatatTAATagtgatgg	1.0	0.977
	V$BARX2.02	+10826	(+)	ttcttttcAATTatttgta	1.0	0.892
	V$HHEX.01	+9618	(-)	atgacatatttTAATttgc	1.0	0.976
	V$HMX2.01	+10696	(+)	tgacagggcCTTAagagtg	1.0	0.900
	V$HMX2.01	+10701	(-)	tgtatcactCTTAaggccc	1.0	0.928
	V$HMX2.01	+10740	(+)	aagatctagCTTAagcctt	1.0	0.885
	V$HMX2.01	+10745	(-)	ttggaaaggCTTAagctag	1.0	0.896
	V$HMX2.02	+10394	(-)	tatcacccAAACcttttta	1.0	0.825
	V$HMX2.02	+10504	(+)	tggtcaatAAACatttgtg	1.0	0.839
	V$HMX2.02	+10960	(-)	tggggaaaAAACgatgttg	1.0	0.855
	V$HMX2.03	+9244	(+)	aaaaaaTTAAgtgttcata	1.0	0.867
	V$HMX3.01	+9197	(-)	cccttgagAAGTggctcta	1.0	0.895
	V$HMX3.01	+10132	(-)	aattcaacAAGTgtgaact	1.0	0.897
	V$HMX3.02	+9240	(+)	agtcaaaaaaTTAAgtgtt	1.0	0.928
	V$HMX3.02	+9245	(-)	atatgaacacTTAAttttt	1.0	0.946
	V$HMX3.02	+10934	(-)	tccatcactaTTAAtatgt	1.0	0.923
	V$MSX.01	+9507	(+)	agtgataTAATttgtccag	1.0	0.972
	V$MSX.01	+9614	(-)	catatttTAATttgcgtga	1.0	0.994
	V$MSX3.01	+9241	(-)	gaacactTAATtttttgac	1.0	0.891
	V$NOBOX.01	+10827	(-)	gtacaaaTAATtgaaaaga	1.0	0.954
V$HOXC	V$HOXC9.02	+9710	(+)	ttccagaTTTAtaatct	1.0	0.950
	V$HOXC9.02	+10452	(-)	ctatttaTTTAtggcct	1.0	0.934
	V$HOXC9.02	+10552	(-)	ctcaataTTTAtggaat	1.0	0.914
	V$HOXC9.02	+10806	(+)	aatgagaTTTActgatg	1.0	0.934
	V$MEIS1.03	+9335	(-)	atcttGATTtatattca	1.0	0.979
V$HOXF	V$HOX1-3.01	+9352	(+)	tgtagTAATggagaacatt	1.0	0.941
	V$HOX1-3.01	+9432	(-)	cgtggTAATggggttaaat	1.0	0.878
	V$HOX1-3.01	+10722	(+)	aatgtTAATgttgatcaca	1.0	0.829
	V$HOX1-3.01	+10800	(+)	cgtagTAATgagatttact	1.0	0.824
	V$HOX1-3.01	+10935	(+)	catatTAATagtgatggaa	1.0	0.877
	V$HOXA2.01	+9294	(+)	gaaggTAATgagtattttg	1.0	0.862
	V$HOXA3.02	+9615	(+)	cacgcaaATTAaaatatgt	1.0	0.844
	V$HOXB3.01	+9990	(-)	agacaTAATcagtagattc	1.0	0.867
	V$HOXB5.01	+9460	(+)	actgtTAATaaccccattt	1.0	0.864
	V$HOXB8.01	+9457	(-)	tggggttATTAacagtatc	1.0	0.864
	V$HOXC6.01	+10825	(-)	acaaatAATTgaaaagaaa	1.0	0.852
	V$HOXD3.01	+9509	(+)	tgatatAATTtgtccaggg	1.0	0.856
	V$HOXD8.01	+9242	(+)	tcaaaaaATTAagtgttca	1.0	0.830
	V$NANOG.01	+10771	(+)	caacatgAATGagtacaaa	1.0	0.967
V$HZIP	V$HOMEZ.01	+10959	(+)	gcaacATCGtttttt	1.0	0.958
V$IKRS	V$IK3.01	+10077	(+)	aactgGGAActca	1.0	0.847
	V$IK3.01	+10114	(+)	ggtagGGAAgacc	1.0	0.882
V$INSM	V$INSM1.01	+10654	(-)	ttcctGGGGgcaa	1.0	0.932
V$IRFF	V$IRF2.01	+10428	(+)	aagtcttaaaaaggGAAAgaaaaga	1.0	0.888
V$IRXF	V$IRX2.01	+9136	(-)	agtaCATGaaaga	1.0	0.869
	V$IRX2.01	+9414	(-)	tatcCATGtagat	1.0	0.866
	V$IRX3.01	+9137	(+)	ctttCATGtactt	1.0	0.864
	V$IRX5.01	+9393	(-)	ttgaCATGtactc	1.0	0.949
	V$IRX5.01	+9394	(+)	agtaCATGtcaat	1.0	0.953
	V$IRX5.01	+9415	(+)	tctaCATGgataa	1.0	0.86
	V$IRX5.01	+9535	(-)	tctaCATGtttgc	1.0	0.965
	V$IRX5.01	+9536	(+)	caaaCATGtagag	1.0	0.969
V$KLFS	V$BKLF.02	+10102	(+)	gatgggGGAGggggtaggg	1.0	0.996
	V$BTEB3.01	+9857	(+)	aagacttgggGGAGtgggt	1.0	0.932
	V$GKLF.02	+9827	(-)	ctgacctaAAAGgagacaa	1.0	0.961

	V$GKLF.03	+10229	(+)	aggctgggtGTGGtggctc	1.0	0.988
	V$KLF6.01	+10100	(+)	ctgatgGGGGagggggtag	1.0	0.941
V$LEFF	V$LEF1.04	+10826	(+)	ttctttTCAAttatttg	1.0	0.842
V$LHXF	V$ISL1.01	+9290	(+)	aacagaaggTAATgagtattttg	1.0	0.833
	V$ISL2.01	+9238	(+)	agagtcaaaaaATTAagtgttca	1.0	0.901
	V$LHX1.01	+10824	(+)	ctttcttttcAATTatttgtaca	1.0	0.824
	V$LHX3.01	+9239	(-)	atgaacacTTAAtttttgactc	1.0	0.855
	V$LHX3.01	+9612	(-)	gacatattTTAAtttgcgtgaaa	1.0	0.812
	V$LHX3.02	+9243	(-)	caatatgaacactTAATtttttg	1.0	0.892
	V$LHX3.02	+9501	(+)	tagagaagtgataTAATttgtcc	1.0	0.897
	V$LHX3.02	+9574	(+)	aaccttctaaagaTAATtttgtg	1.0	0.873
	V$LHX3.02	+9616	(-)	ctatgacatatttTAATttgcgt	1.0	0.831
	V$LHX3.02	+9994	(-)	gactcctaagacaTAATcagtag	1.0	0.825
	V$LHX3.02	+10829	(-)	ctagatgtacaaaTAATtgaaaa	1.0	0.884
	V$LHX9.01	+10825	(-)	atgtacaaatAATTgaaaagaaa	1.0	0.838
V$LTFM	V$LACTOFERRIN.01	+10154	(+)	gGCACtagc	1.0	0.913
V$LTSM	V$LTSM.01	+9676	(+)	gatccagataCATCt	1.0	0.939
V$MAZF	V$MAZ.01	+10105	(+)	ggggGAGGgggta	1.0	0.920
V$MEF2	V$MEF2.06	+10452	(+)	aggccataaatAAATagagcttt	1.0	0.870
	V$MEF2C.01	+10753	(+)	agcctttccaaAAATagtcaaca	1.0	0.984
	V$SL1.01	+10754	(-)	atgttgaCTATttttggaaaggc	1.0	0.864
	V$SL1.01	+10930	(-)	tccatcaCTATtaatatgtgtga	1.0	0.940
V$MITF	V$MIT.01	+10596	(+)	ggagcCATGtgagta	1.0	0.945
V$MIZ1	V$MIZ1.01	+9156	(-)	gaagcCCTCtg	1.0	0.972
	V$MIZ1.01	+9882	(-)	taggcCCTCtc	1.0	0.987
V$MOKF	V$MOK2.01	+9309	(-)	agttttatcagtgCCTTcaaa	1.0	0.843
	V$MOK2.02	+10478	(+)	gatattcacagtaCCTTgaat	1.0	0.980
	V$MOK2.02	+10691	(+)	taccatgacagggCCTTaaga	1.0	0.992
	V$MOK2.02	+10843	(+)	tacatctagggagCCTTatct	1.0	0.986
V$MYBL	V$CMYB.01	+9904	(-)	ctctctctgCAACtgactaac	1.0	0.956
	V$CMYB.02	+9455	(-)	tggggttatTAACagtatctt	1.0	0.961
	V$MYBL2.01	+10532	(+)	tcacagtgtTAACagtgttca	1.0	0.822
V$MYOD	V$TCFE2A.02	+9949	(+)	aacaggcaGATGtaaga	1.0	0.947
	V$TCFE2A.02	+10894	(+)	tctggacaGATGgcata	1.0	0.980
V$MYT1	V$MYT1.02	+10947	(-)	ctaAAGTtccatc	1.0	0.881
	V$MYT1L.01	+10408	(+)	tgatAGTTaaacc	1.0	0.939
V$MZF1	V$MZF1.02	+9484	(+)	atGGGGaaatg	1.0	0.995
	V$MZF1.02	+10092	(+)	aaGGGGaactg	1.0	1.000
	V$MZF1.02	+10422	(+)	agGGGGaagtc	1.0	0.995
	V$MZF1.02	+10969	(-)	ttGGGGaaaaa	1.0	0.995
V$NDPK	V$NM23.01	+10104	(+)	tgGGGGagggggtaggg	1.0	0.940
V$NEUR	V$NGN_NEUROD.01	+9950	(-)	cttaCATCtgcctgt	1.0	0.988
	V$NGN_NEUROD.01	+10895	(-)	atgcCATCtgtccag	1.0	1.000
V$NF1F	V$NF1.02	+10863	(+)	tcttTGGCccctgggcaaagt	1.0	0.817
V$NFAT	V$NFAT.01	+9752	(+)	atatgaGGAAagagctaag	1.0	1.000
	V$NFAT.01	+9936	(+)	ggctgtGGAAaggaacagg	1.0	0.960
	V$NFAT5.01	+9938	(+)	ctgtGGAAaggaacaggca	1.0	0.876
	V$NFAT5.01	+10437	(+)	aaagGGAAagaaaagaggc	1.0	0.851
V$NFKB	V$CREL.01	+9974	(-)	gggggttcTTCCtct	1.0	0.923
	V$NFKAPPAB50.01	+9165	(+)	tcaGGGAgaccctga	1.0	0.832
V$NGRE	V$IR1_NGRE.01	+9161	(+)	ggcttcagGGAGacc	1.0	0.818
V$NKX6	V$NKX61.01	+9243	(-)	acacTTAAtttttg	1.0	0.917
	V$NKX61.01	+9374	(-)	agtgTTAAtaataac	1.0	0.912
	V$NKX61.01	+9460	(+)	actgTTAAtaacccc	1.0	0.956
	V$NKX61.01	+9616	(-)	tattTTAAtttgcgt	1.0	0.920
	V$NKX61.01	+11011	(+)	cttgTTAAttcccag	1.0	0.954
	V$NKX61.02	+10935	(+)	cataTTAAtagtgat	1.0	0.859
V$NKXH	V$NKX25.01	+10283	(-)	ggactcAAGTgatcctcct	1.0	1.000
	V$NKX25.02	+9239	(-)	acactTAATtttttgactc	1.0	0.957
	V$NKX25.02	+10825	(-)	acaaaTAATtgaaaagaaa	1.0	0.883
	V$NKX26.01	+9195	(-)	cttgagaAGTGgctctaag	1.0	0.838
	V$NKX29.01	+9136	(+)	tctttcatGTACttgacat	1.0	0.859
	V$NKX29.01	+9137	(-)	catgtcaaGTACatgaaag	1.0	0.857
	V$NKX31.01	+9762	(+)	agagctAAGTaccagtatg	1.0	0.858
	V$NKX31.02	+11000	(+)	acacatatGTACttgttaa	1.0	0.834
	V$NKX31.04	+9246	(+)	aaaattaAGTGttcatatt	1.0	0.992
	V$NKX31.04	+9377	(-)	ctccgtaAGTGttaataat	1.0	0.960

	V$NKX31.04	+9645	(+)	aattctaAGTGtaaaacat	1.0	0.958
V$NOLF	V$OLF1.02	+9155	(-)	agggtcTCCCtgaagccctctgc	1.0	0.892
	V$OLF1.02	+9853	(-)	acccacTCCCccaagtctttcta	1.0	0.881
	V$OLF1.02	+10412	(-)	aagactTCCCcctgggtttaact	1.0	0.884
V$OCT1	V$OCT1.02	+10576	(+)	catATGCaaagcact	1.0	0.972
	V$OCT1.03	+10802	(-)	taaatctcATTActa	1.0	0.853
	V$OCT1.05	+9396	(+)	taCATGtcaatagta	1.0	0.927
	V$OCT1.06	+9618	(-)	catattttAATTtgc	1.0	0.896
	V$POU3F3.01	+9404	(-)	tagatGCATactatt	1.0	0.888
	V$POU3F3.01	+9407	(+)	agtatGCATctacat	1.0	0.841
	V$POU3F3.01	+9796	(-)	cttctGCATagtcct	1.0	0.872
	V$POU3F3.01	+10573	(-)	gctttGCATatggca	1.0	0.884
V$OVOL	V$OVOL1.01	+9457	(+)	gatactGTTAataac	1.0	0.837
	V$OVOL1.01	+10536	(-)	aacactGTTAacact	1.0	0.875
V$PARF	V$VBP.01	+9951	(+)	caggcagatGTAAgaga	1.0	0.872
V$PAX3	V$PAX3.03	+10684	(-)	cctgtCATGgtacatgctt	1.0	0.992
V$PAX5	V$PAX5.04	+10301	(-)	tcatagctcACTGcagcctcaacctcttg	1.0	0.873
V$PAX6	V$PAX6.04	+10508	(-)	attCCACaaatgtttattg	1.0	0.894
V$PAXH	V$PAX4.02	+9615	(-)	attttAATTtgcgtg	1.0	0.843
V$PCBE	V$PREB.01	+10094	(-)	ccccaTCAGttcccc	1.0	0.904
V$PDX1	V$IPF1.01	+9290	(+)	aacagaaggTAATgagtat	1.0	0.865
	V$IPF1.01	+10796	(+)	tggccgtagTAATgagatt	1.0	0.845
V$PEG3	V$PEG3.01	+9692	(-)	aaggatcctgTGGCt	1.0	0.855
V$PIT1	V$PIT1.01	+9418	(-)	tgagaTTATccatgt	1.0	0.937
	V$PIT1.01	+10455	(-)	tctatTTATttatgg	1.0	0.924
V$PLAG	V$PLAG1.02	+10109	(+)	gaGGGGgtagggaagaccattct	1.0	1.000
	V$PLAG1.02	+10421	(+)	caGGGGgaagtcttaaaaaggga	1.0	1.000
	V$PLAGL1.02	+9969	(-)	tctgGGGGttcttcctctgtaat	1.0	0.844
V$PLZF	V$PLZF.01	+10182	(+)	aagTACAggactagt	1.0	0.932
V$PRDF	V$PRDM1.02	+10435	(+)	aaaaaggGAAAgaaaagag	1.0	0.893
V$RORA	V$REV-ERBA.02	+9622	(+)	attaaaatatGTCAtagcaagtgaa	1.0	0.892
	V$RORA1.01	+9831	(+)	ctccttttaGGTCagtttagagtag	1.0	0.936
V$RP58	V$RP58.01	+9952	(-)	cttaCATCtgcct	1.0	0.861
V$RREB	V$RREB1.01	+9861	(-)	aCCCActcccccaag	1.0	0.808
	V$RREB1.01	+10974	(+)	cCCCAaagaaacatg	1.0	0.840
V$RU49	V$RU49.01	+9768	(+)	aAGTAcc	1.0	1.000
	V$RU49.01	+10486	(+)	cAGTAcc	1.0	0.994
V$RUSH	V$SMARCA3.01	+9471	(+)	ccCCATttcac	1.0	0.961
	V$SMARCA3.01	+9479	(-)	ccCCATgtgtg	1.0	0.961
	V$SMARCA3.01	+9487	(-)	ccCCATttccc	1.0	0.961
	V$SMARCA3.01	+10573	(+)	tgCCATatgca	1.0	0.985
	V$SMARCA3.02	+10135	(+)	tcacACTTgtt	1.0	0.986
	V$SMARCA3.02	+11006	(+)	atgtACTTgtt	1.0	1.000
V$RXRF	V$CAR_RXR.01	+9837	(+)	ttaGGTCagtttagagtagaaagac	1.0	0.802
	V$THRB.02	+9823	(+)	agcattgtctccttttaGGTCagtt	1.0	0.804
V$SAL2	V$SALL2.01	+9870	(+)	gtggGTGGcat	1.0	0.926
V$SATB	V$SATB1.01	+9368	(-)	aatAATAaccaaaat	1.0	0.954
	V$SATB1.01	+9744	(+)	tatAATAaatatgag	1.0	0.955
	V$SATB1.01	+10938	(+)	attAATAgtgatgga	1.0	0.943
V$SF1F	V$FTF.01	+10485	(-)	cattCAAGgtactgt	1.0	0.946
V$SIX3	V$SIX3.02	+10248	(+)	atgcctgTAATcccaccactg	1.0	0.956
V$SMAD	V$GC_SBE.01	+10594	(-)	catggCTCCag	1.0	1.000
	V$SMAD3.01	+10890	(+)	agaGTCTggac	1.0	0.996
V$SORY	V$HBP1.01	+10516	(+)	atttgtggAATGaacctcacagt	1.0	0.872
	V$HBP1.01	+10540	(-)	atttatggAATGaacactgttaa	1.0	0.911
	V$HBP1.01	+10770	(+)	tcaacatgAATGagtacaaattg	1.0	0.923
	V$HBP1.02	+10489	(+)	taccttgAATGtagttggtcaat	1.0	0.856
	V$HMGA.01	+10768	(+)	agtcaacatgAATGagtacaaat	1.0	0.915
	V$HMGA.01	+10824	(+)	ctttcttttcAATTatttgtaca	1.0	0.891
	V$SOX10.03	+9605	(+)	gtTGAAttttcacgcaaattaaa	1.0	0.808
	V$SOX21.03	+9333	(+)	tatGAATataaatcaagattgta	1.0	0.887
	V$SOX21.03	+10143	(+)	gttGAATttgaggcactagcagc	1.0	0.815
	V$SOX5.01	+9813	(-)	aggagaCAATgctaccttgtgct	1.0	0.871
	V$SOX6.01	+10605	(+)	tgagtACAAagcatatgagaacc	1.0	0.982
	V$SOX7.04	+9327	(-)	cttgatttatatTCATaagcagt	1.0	0.810
	V$SRY.02	+9617	(+)	cgcaaATTAaaatatgtcatagc	1.0	0.883
V$SP1F	V$TIEG.01	+9861	(+)	cttgGGGGagtgggtgg	1.0	0.858

V$SREB	V$SREBP.04	+10327	(+)	tgaTCACcaccactgc	1.0	0.981
V$SRFF	V$SRF.03	+9891	(+)	tataccagagATGGttagt	1.0	0.802
V$STAT	V$STAT.01	+10752	(-)	actatttttGGAAaggctt	1.0	0.876
	V$STAT.01	+10966	(-)	tttctttggGGAAaaaacg	1.0	0.915
V$STEM	V$OCT3_4.02	+9145	(-)	gccctctGCATgtcaagta	1.0	0.966
	V$OCT3_4.02	+9405	(+)	atagtatGCATctacatgg	1.0	0.957
	V$OCT3_4.02	+9794	(-)	gtcttctGCATagtcctga	1.0	0.920
	V$OCT3_4.02	+10571	(-)	gtgctttGCATatggcaga	1.0	0.938
V$TAIP	V$CSRNP1.01	+10586	(-)	AGAGtgc	1.0	1.000
	V$CSRNP1.01	+10709	(+)	AGAGtga	1.0	1.000
V$TALE	V$MEIS1.01	+10692	(-)	taaggcccTGTCatggt	1.0	0.969
	V$TGIF.01	+9392	(+)	ggagtacatGTCAatag	1.0	1.000
	V$TGIF.01	+10994	(-)	tacatatgtGTCAataa	1.0	1.000
V$TEAF	V$TEAD.01	+10518	(-)	gttCATTccacaa	1.0	0.954
	V$TEAD.01	+10548	(+)	gttCATTccataa	1.0	0.945
	V$TEAD4.01	+9126	(+)	tggcATTCcttct	1.0	0.980
V$WHNF	V$WHN.01	+9445	(+)	accACGCtata	1.0	0.960
V$XBBF	V$RFX5.01	+10034	(+)	ttcaaggctgAGCAactag	1.0	0.942
	V$RFX5.01	+10948	(+)	atggaactttAGCAacatc	1.0	0.979
	V$XBOX.01	+10865	(-)	actttgcccAGGGgccaaa	1.0	0.911
V$YY1F	V$REX1.01	+9346	(-)	tgttctCCATtactacaatcttg	1.0	0.871
	V$YY1.01	+10782	(-)	ctacggCCATcccaatttgtact	1.0	0.830
	V$YY1.02	+9471	(-)	atttccCCATgtgtgaaatgggg	1.0	0.942
	V$YY1.02	+9783	(+)	attagaCCATttcaggactatgc	1.0	0.965
	V$YY1.02	+10569	(+)	tatctgCCATatgcaaagcactc	1.0	0.941
	V$YY2.02	+9434	(+)	ttaaccCCATtaccacgctataa	1.0	0.952
V$ZF02	V$ZBP89.01	+9854	(-)	cacccactCCCCcaagtctttct	1.0	0.956
	V$ZBP89.01	+10095	(-)	taccccctCCCCcatcagttccc	1.0	0.986
	V$ZBTB7.03	+10097	(-)	cctacCCCCtcccccatcagttc	1.0	0.989
	V$ZNF219.01	+9851	(-)	ccactCCCCcaagtctttctact	1.0	0.936
V$ZF04	V$ZID.01	+9526	(-)	ttGCTCaatgacc	1.0	0.863
	V$ZID.01	+10590	(-)	tgGCTCcagcaga	1.0	0.867
V$ZF05	V$ZFP410.01	+9774	(+)	cagtatgGGATtaga	1.0	0.867
	V$ZFP410.01	+10786	(+)	caaattgGGATggcc	1.0	0.920
V$ZF07	V$ZNF263.01	+10103	(-)	taccccCTCCcccat	1.0	0.979
	V$ZNF263.02	+10172	(-)	tacttcCTCCcacct	1.0	0.917
V$ZF10	V$PRDM14.01	+9193	(+)	tgcTTAGagccactt	1.0	0.876
V$ZF12	V$ZFP652.01	+9379	(-)	ccgtaagtgTTAAta	1.0	0.852
	V$ZFP652.01	+10717	(+)	acaaaaatgTTAAtg	1.0	0.814
	V$ZFP652.01	+10748	(-)	tggaaaggcTTAAgc	1.0	0.894
	V$ZNF652.02	+9432	(-)	gtaatgGGGTtaaat	1.0	0.818
V$ZF57	V$ZFP57.01	+9490	(-)	tatTGCCccattt	1.0	0.843
V$ZFHX	V$AREB6.04	+10978	(-)	gtcatGTTTcttt	1.0	0.984
V$ZTRE	V$ZTRE.03	+9856	(-)	caCTCCccccaagtcttt	1.0	0.974
	V$ZTRE.03	+10097	(-)	ccCTCCccccatcagttc	1.0	0.993
	V$ZTRE.04	+9864	(+)	gggGGAGtgggtggcat	1.0	0.962
	V$ZTRE.04	+10105	(+)	gggGGAGggggtaggga	1.0	0.984

Intron 4

O$PTBP	O$PTATA.02	+11428	(-)	tgtcTATAaaaaggg	1.0	0.966
	O$PTATA.02	+11554	(+)	tcatTATAaatcaac	1.0	0.908
	O$PTATA.02	+12970	(-)	ttacTATAgataatg	1.0	0.919
O$VTBP	O$ATATA.01	+11693	(-)	tgatatgTAAGtactac	1.0	0.881
	O$ATATA.01	+12350	(-)	tttttttTAAGtttact	1.0	0.825
	O$MTATA.01	+11225	(-)	gttttTAAAattttact	1.0	0.848
	O$MTATA.01	+11614	(-)	ctaaaTAAAgcctcacc	1.0	0.854
	O$MTATA.01	+12838	(-)	tcctcTAAAacagcaag	1.0	0.850
	O$MTATA.01	+13006	(+)	ggctcTAAAcctctgta	1.0	0.841
	O$VTATA.01	+11425	(-)	gtctaTAAAaaggggag	1.0	0.996
	O$VTATA.01	+11555	(+)	cattaTAAAtcaacctt	1.0	0.924
O$XCPE	O$XCPE1.01	+12619	(-)	gaGCGGtaaca	1.0	0.814
O$YTBP	O$SPT15.01	+12077	(+)	tacaaaaTATAtacatc	1.0	0.878
	O$SPT15.01	+12078	(-)	ggatgtaTATAttttgt	1.0	0.860
	O$SPT15.01	+12080	(-)	gaggatgTATAtatttt	1.0	0.862
V$ABDB	V$HOXA13.01	+12919	(-)	atacctggTAAAggcaa	1.0	0.840
	V$HOXB9.01	+12344	(+)	aatggtagTAAActtaa	1.0	0.921

	V$HOXB9.01	+12762	(-)	aaatgtggTAAAcattc	1.0	0.886
	V$HOXB9.01	+12771	(-)	catgatagTAAAtgtgg	1.0	0.905
	V$HOXB9.01	+12785	(-)	ctaagttgTAAAgtcat	1.0	0.885
	V$HOXC13.01	+12173	(-)	gatatttaaTAAAtcat	1.0	0.935
	V$HOXC9.01	+12489	(-)	tccagtgaTTAAatttg	1.0	0.889
	V$HOXC9.01	+12814	(-)	taaggtagTTAAtattc	1.0	0.835
	V$HOXC9.01	+12864	(-)	cctcataaTTAAccaga	1.0	0.832
	V$HOXC9.01	+13302	(-)	accagttaTTAAaatga	1.0	0.881
	V$HOXD13.01	+11428	(-)	tttgtctaTAAAaaggg	1.0	0.931
V$AP1F	V$JUNB.01	+12450	(+)	tatttaGTCAaat	1.0	0.912
	V$JUNB.01	+12661	(+)	gggttaGTCAaag	1.0	0.913
V$AP1R	V$MAFB.01	+11370	(+)	atatgtattatgTCAGctaatct	1.0	0.869
	V$TCF11MAFG.01	+12778	(-)	taagttgtaaaGTCAtgatagta	1.0	0.828
	V$VMAF.01	+12602	(-)	gtaacaccagaGTCAtcagtagg	1.0	0.829
V$ARID	V$ARID5A.01	+11405	(-)	gagcaATATtccttatcctgc	1.0	0.950
	V$ARID5A.01	+11412	(+)	aaggaATATtgctctcccctt	1.0	0.964
	V$ARID5A.01	+12804	(-)	agttaATATtcagtatcttgt	1.0	0.956
	V$ARID5A.01	+12811	(+)	actgaATATtaactaccttat	1.0	0.936
	V$BRIGHT.01	+12858	(-)	tcataATTAaccagaagccct	1.0	0.922
	V$MRF2.01	+12221	(+)	actgAATAcccagtttggtat	1.0	0.985
V$ATBF	V$ATBF1.01	+11634	(+)	aatacatgctAATTatg	1.0	0.812
	V$ATBF1.01	+12071	(-)	tatattttgtAATTtgc	1.0	0.803
	V$ATBF1.01	+12868	(-)	gcttcctcatAATTaac	1.0	0.813
V$BARB	V$BARBIE.01	+11423	(-)	ataaAAAGgggagag	1.0	0.908
	V$BARBIE.01	+12934	(+)	attgAAAGcaaactg	1.0	0.890
V$BCDF	V$CRX.01	+12490	(+)	aaattTAATcactggaa	1.0	0.951
	V$CRX.01	+12579	(-)	tatttTAATctgcactc	1.0	0.977
	V$CRX.01	+13294	(+)	agcttTAATcattttaa	1.0	0.946
	V$CRX.03	+11270	(-)	tgggataATCCtatatg	1.0	0.964
	V$PCE1.01	+11638	(+)	catgcTAATtatggctg	1.0	0.895
	V$PCE1.01	+12319	(+)	aaaacTAATtctggttg	1.0	0.893
	V$PTX1.01	+12655	(-)	ttgaCTAAccccatccc	1.0	0.957
	V$PTX1.01	+13006	(+)	ggctCTAAacctctgta	1.0	0.959
V$BCL6	V$BCL6.02	+13250	(+)	tgcttttTAGAaaatta	1.0	0.840
	V$BCL6.04	+11620	(-)	attTTCCtaaataaagc	1.0	0.886
V$BRAC	V$TBX20.02	+11668	(-)	ctacataaccagtggtGACAaccagggcc	1.0	0.938
V$BRN5	V$BRN5.03	+11627	(-)	aTAATtagcatgtattttcctaa	1.0	0.828
	V$BRN5.04	+11545	(+)	aaaacttcatcATTAtaaatcaa	1.0	0.838
	V$BRN5.04	+11634	(+)	aatacatgctaATTAtggctgtc	1.0	0.933
V$BRNF	V$BRN2.03	+13190	(+)	ttctgatttATTTgctttc	1.0	0.947
	V$BRN2.04	+11636	(+)	tacatgcTAATtatggctg	1.0	0.859
	V$BRN2.04	+11637	(-)	acagccaTAATtagcatgt	1.0	0.868
	V$BRN2.04	+12172	(-)	agatattTAATaaatcatg	1.0	0.841
	V$BRN2.04	+12863	(+)	ttctggtTAATtatgagga	1.0	0.897
	V$BRN2.04	+12864	(-)	ttcctcaTAATtaaccaga	1.0	0.853
	V$BRN3.02	+13292	(+)	acagcttTAATcattttaa	1.0	0.913
	V$BRN3.03	+13301	(+)	atcatttTAATaactggtc	1.0	0.832
	V$BRN4.02	+12175	(+)	gatttaTTAAatatctgct	1.0	0.807
	V$TST1.01	+11124	(+)	gcaaaATTAacaagtctac	1.0	0.917
	V$TST1.01	+13298	(-)	cagttATTAaaatgattaa	1.0	0.910
V$BTBF	V$KAISO.01	+11878	(+)	catcCTGCaat	1.0	0.986
	V$KAISO.01	+12376	(+)	agtcCTGCaaa	1.0	0.977
V$BZIP	V$CEBPE_ATF4.01	+13091	(+)	aatgagGCAAaaa	1.0	0.985
	V$CEBPE_ATF4.02	+11829	(+)	gtttatGCAAgaa	1.0	0.883
V$CAAT	V$CAAT.01	+12734	(-)	agaaCCAAtgaaatt	1.0	0.950
	V$NFY.04	+11148	(-)	aaaaCCAAtctacaa	1.0	0.923
	V$NFY.05	+11659	(-)	agggCCAAgcagccc	1.0	0.846
V$CARE	V$CARF.01	+13089	(+)	acaatGAGGca	1.0	0.933
V$CART	V$HESX1.01	+12063	(-)	ttttgTAATttgctatgagac	1.0	0.808
	V$MIXL1.01	+13232	(-)	gcaaaTAATttcataatacag	1.0	0.885
	V$RHOX6.01	+12168	(-)	atattTAATaaatcatgagtg	1.0	0.857
	V$RHOX6.01	+13303	(+)	catttTAATaactggtctttg	1.0	0.844
	V$S8.01	+11633	(-)	agccaTAATtagcatgtattt	1.0	0.997
	V$S8.01	+11638	(+)	catgcTAATtatggctgtcct	1.0	0.992
	V$S8.01	+12860	(-)	cctcaTAATtaaccagaagcc	1.0	0.997
	V$S8.01	+12865	(+)	ctggtTAATtatgaggaagcc	1.0	0.992
	V$XVENT2.01	+13294	(+)	agcttTAATcattttaataac	1.0	0.822
V$CDXF	V$CDX1.01	+11219	(-)	ttaaaatTTTActaaaatt	1.0	0.944

	V$CDX1.01	+13299	(+)	taatcatTTTAataactgg	1.0	0.957
	V$CDX1.02	+12344	(-)	ttttaagTTTActaccatt	1.0	0.875
	V$CDX2.03	+11279	(-)	gtatggatTTATgggataa	1.0	0.954
	V$CDX2.03	+11426	(+)	tccccttTTATagacaaa	1.0	0.937
	V$CDX2.03	+11838	(-)	gagactgtTTATggattct	1.0	0.957
V$CEBP	V$CEBP.02	+11827	(+)	aggtttatGCAAgaa	1.0	0.951
	V$CEBPB.01	+13233	(+)	tgtattatGAAAtta	1.0	0.979
	V$CEBPB.02	+11917	(-)	ttgaaTGTGaaatta	1.0	0.982
	V$CEBPB.02	+12003	(-)	ggctgTGTGgaaaga	1.0	0.917
	V$CEBPB.02	+13272	(-)	tgcacTGTGtaaaga	1.0	0.931
	V$CEBPE.02	+11964	(+)	ctgcttctGCAAtgg	1.0	0.974
V$CHRF	V$CHR.01	+11158	(+)	gtttTTGAataaa	1.0	0.930
	V$CHR.01	+11212	(+)	tattTTGAatttt	1.0	0.941
	V$CHR.01	+11520	(+)	gggtTTGAagaga	1.0	0.938
	V$CHR.01	+11807	(-)	tggcTTGAatttg	1.0	0.926
	V$CHR.01	+11923	(-)	gcatTTGAatgtg	1.0	0.975
	V$CHR.01	+13177	(+)	cagtTTGAaccca	1.0	0.962
V$CIZF	V$NMP4.01	+12475	(-)	ggAAAAaacag	1.0	0.982
	V$NMP4.01	+13097	(+)	gcAAAAaattc	1.0	0.971
V$CLOX	V$CDP.02	+11142	(-)	caaaaacCAATctacaaatagag	1.0	0.948
	V$CDP.02	+12728	(-)	ccagaacCAATgaaattgctagt	1.0	0.977
	V$CPHX.01	+11907	(+)	tcTGATctgataatttcacattc	1.0	0.851
	V$CPHX.01	+11912	(+)	tcTGATaatttcacattcaaatg	1.0	0.851
V$CREB	V$BATF3.01	+11539	(-)	taatGATGaagtttttaactt	1.0	0.836
	V$CREB1.02	+12745	(-)	attctATGAtgtgagccagaa	1.0	0.946
	V$CREB2.01	+11370	(-)	attagcTGACataatacatat	1.0	0.915
	V$E4BP4.01	+11693	(-)	tgtatgatatGTAAgtactac	1.0	0.866
	V$E4BP4.01	+12020	(+)	gacaatttctGTAAgcacaga	1.0	0.816
	V$E4BP4.01	+12069	(-)	tatatattttGTAAtttgcta	1.0	0.844
V$DLXF	V$DLX1.01	+12861	(+)	gcttctggttAATTatgag	1.0	0.982
V$DLXF	V$DLX1.01	+12866	(-)	gcttcctcatAATTaacca	1.0	0.988
	V$DLX2.01	+11634	(+)	aatacatgctAATTatggc	1.0	0.981
	V$DLX3.01	+11908	(+)	ctgatctgaTAATttcaca	1.0	0.933
	V$DLX3.01	+13238	(-)	aaaagcaaaTAATttcata	1.0	0.924
	V$DLX4.01	+11639	(-)	ggacagccatAATTagcat	1.0	0.977
V$DMRT	V$DMRT5.01	+11336	(-)	aaggtgtGTTAccgtttacta	1.0	0.848
V$DMTF	V$DMP1.01	+11873	(-)	ttgcaGGATgtggag	1.0	0.833
V$DUXF	V$DUX4.01	+12488	(-)	agtGATTaaatttgt	1.0	0.832
V$E2FF	V$E2F7.02	+11934	(-)	aggtggaGGGAatgagc	1.0	0.889
V$EBOX	V$ATF6.01	+12995	(-)	agagCCACtctgaggac	1.0	0.930
V$EGRF	V$EGR2.02	+12190	(-)	ttgTGGGaggaggagagca	1.0	0.957
V$ETSF	V$ERG.02	+12109	(-)	tgttgggaGGAAatacggata	1.0	0.931
	V$ERG.02	+12473	(-)	atttgtaaGGAAaaaacagca	1.0	0.935
	V$ERG.02	+12949	(+)	gaagagcaGGAAagagataac	1.0	0.940
	V$GABP.01	+12940	(+)	agcaaactGGAAgagcaggaa	1.0	0.953
	V$NRF2.01	+12495	(+)	taatcactGGAAgatcatttg	1.0	0.878
	V$PDEF.01	+11870	(-)	tgattgcaGGATgtggagcaa	1.0	0.979
	V$PEA3.01	+12871	(+)	aattatgAGGAagccaggtat	1.0	0.952
	V$SPI1.02	+11759	(+)	aggttagtGGAActgtctcta	1.0	0.962
	V$SPI1.02	+12034	(-)	ttgtacggGGAAgttctgtgc	1.0	0.986
	V$SPI1.02	+12844	(+)	gttttagaGGAActagggctt	1.0	0.977
	V$SPIB.01	+13165	(-)	ttcaaactGGAAaatatccta	1.0	0.884
	V$SPIC.01	+11571	(+)	ttactattGGAAgtagtttga	1.0	0.839
V$EVI1	V$EVI1.02	+12239	(-)	ctaacAAGAcaaggata	1.0	0.842
	V$EVI1.05	+11497	(+)	ttgagcaGATActattt	1.0	0.813
	V$EVI1.05	+11908	(+)	ctgatctGATAatttca	1.0	0.819
	V$EVI1.05	+12180	(-)	gagagcaGATAtttaat	1.0	0.855
	V$EVI1.06	+12801	(+)	gcaacaAGATactgaat	1.0	0.952
	V$EVI1.07	+11995	(-)	gtggaAAGAtgggttgg	1.0	0.911
	V$MEL1.02	+11587	(+)	tttgattGATGaggtgg	1.0	0.996
V$FAST	V$FAST1.01	+12441	(+)	aacattgtaTATTtagt	1.0	0.901
	V$FAST1.01	+12544	(-)	ttgtctgtgTATTttga	1.0	0.905
	V$FAST1.03	+11144	(+)	ctatttgtaGATTggtt	1.0	0.908
	V$FAST1.03	+12292	(+)	agagatgtaGATTgaga	1.0	0.911
V$FKHD	V$FHXB.01	+11370	(-)	gctgacATAAtacatat	1.0	0.884
	V$FOXO1.01	+12895	(-)	atatagttAACAgccat	1.0	0.890
	V$FOXP1_ES.01	+12470	(-)	aggaaaaAACAgcatag	1.0	1.000
	V$FOXP1_ES.01	+12837	(-)	cctctaaAACAgcaaga	1.0	1.000

	V$FREAC7.01	+11840	(+)	aatccaTAAAcagtctc	1.0	0.962
	V$HFH1.01	+12267	(-)	tagaacTAAActttcca	1.0	0.850
	V$HNF3.01	+12677	(-)	ttaatgcAAACacaagt	1.0	0.995
	V$HNF3.01	+12760	(-)	atgtggtAAACattcta	1.0	0.986
	V$HNF3B.01	+11504	(-)	cataagcaAATAgtatc	1.0	0.964
	V$HNF3B.01	+13193	(-)	ggaaagcaAATAaatca	1.0	0.947
	V$HNF3B.01	+13241	(-)	aaaaagcaAATAatttc	1.0	0.983
	V$HNF3B.03	+13267	(-)	actgtgTAAAgaacatg	1.0	0.880
V$GATA	V$GATA.01	+11405	(+)	gcagGATAaggaa	1.0	0.940
	V$GATA.01	+12104	(-)	tacgGATAaggac	1.0	0.985
	V$GATA.01	+12147	(-)	acagGATAaggtg	1.0	0.938
	V$GATA1.01	+12968	(-)	tataGATAatggt	1.0	0.974
	V$GATA1.02	+11383	(-)	tgaaGATTagctg	1.0	1.000
	V$GATA1.03	+11911	(+)	atctGATAatttc	1.0	0.964
	V$GATA1.05	+12983	(+)	aaggGATAaagtg	1.0	0.894
	V$GATA1.06	+12804	(+)	acaaGATActgaa	1.0	0.961
	V$GATA2.02	+11500	(+)	agcaGATActatt	1.0	0.907
	V$GATA3.02	+12311	(+)	ggcAGATcaaaac	1.0	0.920
	V$GATA4.01	+12824	(-)	atgaGATAaggta	1.0	0.995
	V$GATA4.01	+12960	(+)	aagaGATAaccat	1.0	0.967
V$GCMF	V$GCM1.03	+11748	(+)	gcctgCCCCtaaggt	1.0	0.837
	V$GCM1.03	+12204	(+)	cacaaCCCCtaacgc	1.0	0.835
V$GFI1	V$GFI1.02	+12493	(+)	tttAATCactggaag	1.0	0.904
	V$GFI1.02	+13186	(-)	ataAATCagaatggg	1.0	0.907
V$GREF	V$PRE.01	+13258	(+)	agaaaattacaTGTTcttt	1.0	0.892
V$GRHL	V$GRHL1.01	+11484	(-)	aagacgGGTTccc	1.0	0.863
	V$GRHL1.01	+11681	(+)	accactGGTTatg	1.0	0.879
	V$GRHL2.01	+11821	(-)	taAACCtgtgtac	1.0	0.869
	V$GRHL3.01	+11822	(+)	tacacaGGTTtat	1.0	0.888
V$HAML	V$AML2.01	+12511	(+)	attTGTGgttgaatg	1.0	0.978
V$HAND	V$LYL1_E12.01	+13057	(+)	ggcattacaGATGaatggaaa	1.0	0.825
	V$LYL1_E12.01	+13102	(-)	aagacaataGATGttgaattt	1.0	0.850
	V$TAL1ALPHAE47.01	+11456	(+)	ggttctaCAGAtgatcagtgg	1.0	0.928
	V$TAL1BETAE47.01	+11730	(-)	ggcatctCAGAtggtttcagg	1.0	0.919
V$HBOX	V$GBX1.01	+12318	(+)	caaaactAATTctggttga	1.0	0.861
	V$GSH1.01	+11299	(+)	tattcccataTAATtttgc	1.0	0.911
	V$GSH1.01	+11633	(+)	aaatacatgcTAATtatgg	1.0	0.886
	V$GSH1.01	+12275	(+)	ttagttctaaTAATgctag	1.0	0.857
	V$GSH1.01	+12314	(+)	agatcaaaacTAATtctgg	1.0	0.920
	V$GSH1.01	+13239	(-)	aaaaagcaaaTAATttcat	1.0	0.884
	V$GSH2.01	+11636	(-)	cagccaTAATtagcatgta	1.0	0.955
	V$GSH2.01	+12066	(-)	attttgTAATttgctatga	1.0	0.952
	V$GSH2.02	+11637	(+)	acatgctAATTatggctgt	1.0	0.958
	V$GSH2.02	+12863	(-)	tcctcatAATTaaccagaa	1.0	0.947
	V$GSH2.02	+12864	(+)	tctggttAATTatgaggaa	1.0	0.943
	V$MEOX1.01	+13255	(+)	tttagaaAATTacatgttc	1.0	0.855
V$HMTB	V$MTBF.01	+11209	(+)	tgctATTTt	1.0	0.923
	V$MTBF.01	+12886	(+)	aggtATTTg	1.0	0.979
V$HNF1	V$HMBOX.01	+11126	(-)	gtagacttGTTAatttt	1.0	0.911
	V$HMBOX.01	+11680	(+)	caccactgGTTAtgtag	1.0	0.905
	V$HMBOX.01	+12243	(+)	cttgtcttGTTAgagtc	1.0	0.863
	V$HMBOX.01	+12333	(+)	ttgaaatgGTTAatggt	1.0	0.848
	V$HMBOX.01	+12860	(+)	ggcttctgGTTAattat	1.0	0.933
	V$HNF1.01	+12808	(-)	aGTTAatattcagtatc	1.0	0.887
	V$HNF1.02	+12961	(-)	agaTAATggttatctct	1.0	0.810
	V$HNF1.03	+11119	(-)	tGTTAattttgctctcc	1.0	0.853
	V$HNF1.03	+12340	(+)	gGTTAatggtagtaaac	1.0	0.855
	V$HNF1.03	+12867	(+)	gGTTAattatgaggaag	1.0	0.884
	V$HNF1.04	+11533	(+)	ttctacaaGTTAaaaac	1.0	0.861
	V$HNF1.04	+12893	(+)	tgatggctGTTAactat	1.0	0.876
	V$TCF2.01	+12815	(-)	ataaggtaGTTAatatt	1.0	0.892
	V$TCF2.01	+12898	(-)	gctatataGTTAacagc	1.0	0.966
V$HNF6	V$HNF6.01	+11556	(+)	attataaaTCAAccttt	1.0	0.819
	V$HNF6.02	+11587	(-)	ccacctcatCAATcaaa	1.0	0.917
	V$OC2.01	+11146	(-)	aaaaccAATCtacaaat	1.0	0.855
	V$OC2.01	+13127	(-)	tcacaaAATCtaaaggt	1.0	0.866
V$HOMF	V$BARX2.01	+11121	(-)	gacttgtTAATtttgctct	1.0	0.955
	V$BARX2.01	+11548	(-)	tgatttaTAATgatgaagt	1.0	0.959

	V$BARX2.01	+12336	(+)	aaatggtTAATggtagtaa	1.0	0.957
	V$BARX2.01	+12863	(+)	ttctggtTAATtatgagga	1.0	0.969
	V$BARX2.01	+12963	(-)	ctatagaTAATggttatct	1.0	0.967
	V$BSX.01	+11636	(+)	tacatgctAATTatggctg	1.0	0.955
	V$BSX.01	+11637	(-)	acagccatAATTagcatgt	1.0	0.961
	V$BSX.01	+12864	(-)	ttcctcatAATTaaccaga	1.0	0.959
	V$HHEX.01	+12176	(-)	gagcagatattTAATaaat	1.0	0.976
	V$HHEX.01	+12484	(+)	ccttacaaattTAATcact	1.0	0.965
	V$HHEX.01	+12583	(-)	tattcgtatttTAATctgc	1.0	0.954
	V$HHEX.01	+13288	(+)	atgtacagcttTAATcatt	1.0	0.953
	V$HHEX.01	+13297	(+)	tttaatcatttTAATaact	1.0	0.987
	V$HMX2.01	+12412	(+)	aaaaaagtgCTTAaggtga	1.0	0.942
	V$HMX2.01	+12417	(-)	ctctgtcacCTTAagcact	1.0	0.928
	V$HMX2.02	+11332	(+)	ctcatagtAAACggtaaca	1.0	0.863
	V$MSX2.01	+12317	(+)	tcaaaaCTAAttctggttg	1.0	0.987
	V$TLX2.01	+12175	(+)	gatttaTTAAatatctgct	1.0	0.852
V$HOXC	V$HOXB4.02	+13076	(+)	aataTGATcgaagacaa	1.0	0.864
	V$HOXC9.02	+11281	(-)	gtatggaTTTAtgggat	1.0	0.960
	V$MEIS1.03	+11554	(-)	aggttGATTtataatga	1.0	0.984
	V$MEIS1.03	+12170	(+)	ctcatGATTtattaaat	1.0	0.987
	V$MEIS1.03	+12488	(-)	ccagtGATTaaatttgt	1.0	0.889
	V$MEIS1.03	+12534	(-)	attttGATTcacccagt	1.0	0.880
	V$MEIS1.03	+13189	(+)	attctGATTtatttgct	1.0	0.975
	V$MEIS1.03	+13292	(-)	aaaatGATTaaagctgt	1.0	0.894
	V$PBX1.01	+11585	(+)	agtttGATTgatgaggt	1.0	0.993
	V$PBX1.01	+12296	(+)	atgtaGATTgagactgg	1.0	0.806
V$HOXF	V$HOX1-3.01	+11546	(-)	atttaTAATgatgaagttt	1.0	0.867
	V$HOX1-3.01	+12319	(+)	aaaacTAATtctggttgaa	1.0	0.863
	V$HOXA3.02	+11635	(-)	agccataATTAgcatgtat	1.0	0.967
	V$HOXA3.02	+11638	(+)	catgctaATTAtggctgtc	1.0	0.969
	V$HOXA3.02	+12862	(-)	cctcataATTAaccagaag	1.0	0.971
	V$HOXA3.02	+12865	(+)	ctggttaATTAtgaggaag	1.0	0.968
	V$HOXA5.01	+12065	(-)	ttttgtAATTtgctatgag	1.0	0.845
	V$HOXB6.01	+11912	(+)	tctgatAATTtcacattca	1.0	0.831
	V$HOXB6.01	+13253	(-)	acatgtAATTttctaaaaa	1.0	0.836
	V$HOXB8.01	+11909	(-)	atgtgaaATTAtcagatca	1.0	0.845
	V$HOXB8.01	+13300	(-)	accagttATTAaaatgatt	1.0	0.842
	V$HOXD8.01	+13237	(+)	ttatgaaATTAtttgcttt	1.0	0.837
	V$NANOG.01	+11917	(-)	gcatttgAATGtgaaatta	1.0	0.956
	V$NANOG.01	+11929	(-)	tggagggAATGagcatttg	1.0	0.947
	V$NANOG.01	+12337	(+)	aatggttAATGgtagtaaa	1.0	0.952
	V$NANOG.01	+12515	(+)	gtggttgAATGtgactgtt	1.0	0.949
	V$NANOG.01	+13063	(+)	acagatgAATGgaaatatg	1.0	0.955
	V$NANOG.01	+13201	(-)	ttttctgAATGgaaagcaa	1.0	0.955
V$IKRS	V$IK2.01	+11297	(-)	atatGGGAatatg	1.0	0.983
	V$IK3.01	+11480	(+)	gcctgGGAAcccg	1.0	0.844
V$IRFF	V$IRF3.01	+11913	(-)	gagcatttgaatgtGAAAttatcag	1.0	0.871
	V$IRF7.01	+12400	(+)	actgtgtaGAAAaaaaaagtgctta	1.0	0.866
V$IRXF	V$IRX5.01	+13262	(-)	agaaCATGtaatt	1.0	0.971
	V$IRX5.01	+13263	(+)	attaCATGttctt	1.0	0.969
V$KLFS	V$EKLF.01	+13025	(+)	aaaacctcgaaGGGTtttc	1.0	0.926
	V$KLF12.01	+11674	(-)	ataaccaGTGGtgacaacc	1.0	0.962
V$LEFF	V$LEF1.01	+12557	(-)	caactaaCAAAgattgt	1.0	0.865
	V$LEF1.02	+11360	(-)	tacatatCAAAgtatag	1.0	0.973
	V$LEF1.02	+12636	(-)	agtttctCAAAgtgcgc	1.0	0.945
	V$LEF1.02	+12662	(+)	ggttagtCAAAgaggac	1.0	0.942
	V$LEF1.04	+11585	(-)	acctcaTCAAtcaaact	1.0	0.966
	V$TCF7.01	+12311	(+)	ggcagatCAAAactaat	1.0	0.884
V$LHXF	V$ISL1.01	+12334	(+)	tgaaatggtTAATggtagtaaac	1.0	0.826
	V$ISL1.01	+12486	(+)	ttacaaattTAATcactggaaga	1.0	0.823
	V$ISL1.01	+13299	(+)	taatcatttTAATaactggtctt	1.0	0.828
	V$ISL2.01	+12169	(+)	actcatgatttATTAaatatctg	1.0	0.884
	V$LHX3.01	+12170	(-)	gcagatatTTAAtaaatcatgag	1.0	0.825
	V$LHX3.02	+11296	(+)	acatattcccataTAATtttgct	1.0	0.849
	V$LHX3.02	+12174	(-)	gagagcagatattTAATaaatca	1.0	0.840
	V$LHX3.02	+12311	(+)	ggcagatcaaaacTAATtctggt	1.0	0.826
	V$LHX3.02	+12482	(+)	ttccttacaaattTAATcactgg	1.0	0.856
	V$LHX3.02	+13257	(-)	tgtaaagaacatgTAATttcta	1.0	0.860

	V$LHX4.01	+12065	(-)	tatattttgtAATTtgctatgag	1.0	0.854
	V$LHX4.01	+12315	(+)	gatcaaaactAATTctggttgaa	1.0	0.845
	V$LHX4.01	+12316	(-)	tttcaaccagAATTagttttgat	1.0	0.847
	V$LHX6.01	+11634	(+)	aatacatgcTAATtatggctgtc	1.0	0.921
	V$LHX6.01	+11635	(-)	ggacagccaTAATtagcatgtat	1.0	0.920
	V$LHX6.01	+12861	(+)	gcttctggtTAATtatgaggaag	1.0	0.910
	V$LHX6.01	+13290	(+)	gtacagcttTAATcattttaata	1.0	0.849
	V$LHX9.01	+12862	(-)	gcttcctcatAATTaaccagaag	1.0	0.895
V$LTSM	V$LTSM.03	+12091	(+)	ATCCtctacctatgt	1.0	0.857
V$MAZF	V$MAZ.01	+12191	(-)	ggagGAGGagagc	1.0	0.940
V$MEF2	V$MEF2.01	+12350	(+)	agtaaactTAAAaaaaactgttg	1.0	0.862
	V$MEF2.06	+11205	(-)	actaaaattcaAAATagcactgt	1.0	0.909
	V$RSRFC4.01	+11138	(-)	aaccaatctacaaATAGagtaga	1.0	0.939
	V$SL1.01	+11137	(+)	gtctactCTATttgtagattggt	1.0	0.960
	V$SL1.01	+11204	(+)	tacagtgCTATtttgaattttag	1.0	0.851
	V$SL1.01	+11424	(-)	gttttgtCTATaaaaaggggaga	1.0	0.845
	V$SL1.01	+12966	(-)	tcccttaCTATagataatggtta	1.0	0.890
V$MEF3	V$SIX2.02	+11450	(+)	ggtTCAGgttcta	1.0	0.957
V$MTF1	V$MTF-1.01	+12156	(+)	ctgtGCACacagcac	1.0	0.929
V$MYBL	V$CMYB.02	+12523	(-)	ttcacccagTAACagtcacat	1.0	0.967
	V$VMYB.04	+12204	(+)	cacaacccctAACGcacactg	1.0	0.892
	V$VMYB.05	+11331	(+)	actcatagtaAACGgtaacac	1.0	0.952
V$MYOD	V$TCFE2A.02	+11457	(+)	gttctacaGATGatcag	1.0	0.940
	V$TCFE2A.02	+11733	(-)	gcatctcaGATGgtttc	1.0	0.972
V$MYT1	V$MYT1.02	+11542	(-)	atgAAGTttttaa	1.0	0.895
	V$MYT1.02	+12349	(-)	tttAAGTttacta	1.0	0.880
	V$MYT1L.01	+11764	(-)	agacAGTTccact	1.0	0.931
	V$MYT1L.01	+12268	(+)	ggaaAGTTtagtt	1.0	0.986
V$MZF1	V$MZF1.02	+11422	(-)	aaGGGGagagc	1.0	0.991
V$NEUR	V$ATOH1.01	+11734	(+)	aaacCATCtgagatg	1.0	0.958
	V$NGN_NEUROD.01	+11458	(-)	tgatCATCtgtagaa	1.0	0.985
	V$NGN_NEUROD.01	+13059	(-)	cattCATCtgtaatg	1.0	0.985
V$NFAT	V$NFAT.01	+11997	(-)	ctgtgtGGAAagatgggtt	1.0	0.984
	V$NFAT.01	+12109	(-)	ttgggaGGAAatacggata	1.0	0.994
	V$NFAT.01	+12473	(-)	ttgtaaGGAAaaaacagca	1.0	0.977
	V$NFAT5.01	+13195	(-)	gaatGGAAagcaaataaat	1.0	0.849
	V$NFAT5.02	+11995	(-)	gtgtGGAAagatgggttgg	1.0	0.885
	V$NFAT5.02	+12264	(+)	cactGGAAagtttagttct	1.0	0.915
	V$NFAT5.02	+13069	(+)	gaatGGAAatatgatcgaa	1.0	0.878
	V$NFAT5.02	+13163	(-)	aactGGAAaatatcctatg	1.0	0.921
	V$NFAT5.02	+13211	(-)	cctaGGAAatttttctgaa	1.0	0.884
V$NFKB	V$HIVEP1.01	+11273	(-)	atGGGAtaatcctat	1.0	0.861
V$NGRE	V$IR2_NGRE.01	+12692	(-)	ttCTCCctggaggtt	1.0	0.890
	V$IR2_NGRE.01	+12693	(+)	acCTCCagggagaag	1.0	0.986
V$NKX1	V$NKX11.01	+11637	(-)	agccatAATTagcatgt	1.0	0.879
	V$NKX12.01	+11638	(+)	catgctAATTatggctg	1.0	0.916
V$NKX6	V$NKX61.01	+11123	(-)	cttgTTAAttttgct	1.0	0.917
	V$NKX61.01	+12174	(-)	atatTTAAtaaatca	1.0	0.915
	V$NKX61.01	+12490	(+)	aaatTTAAtcactgg	1.0	0.938
	V$NKX61.01	+12865	(+)	ctggTTAAttatgag	1.0	0.956
	V$NKX61.01	+13303	(+)	cattTTAAtaactgg	1.0	0.956
	V$NKX63.01	+13294	(+)	agcttTAATcatttt	1.0	0.866
V$NKXH	V$NKX25.02	+11119	(-)	cttgtTAATtttgctctcc	1.0	0.884
	V$NKX25.02	+11635	(-)	agccaTAATtagcatgtat	1.0	0.951
	V$NKX25.02	+12862	(-)	cctcaTAATtaaccagaag	1.0	0.951
	V$NKX25.02	+12865	(+)	ctggtTAATtatgaggaag	1.0	0.899
	V$NKX25.05	+11322	(-)	tactaTGAGtgccatgatg	1.0	0.979
	V$NKX25.05	+12160	(-)	aatcaTGAGtgctgtgtgc	1.0	0.983
	V$NKX31.01	+11689	(-)	atatgtAAGTactacataa	1.0	0.893
	V$NKX31.01	+12346	(-)	tttttAAGTttactacca	1.0	0.878
	V$NKX31.02	+11688	(+)	gttatgtaGTACttacata	1.0	0.848
	V$NKX31.02	+11786	(-)	ccatttagGTACtgtccat	1.0	0.828
V$NOLF	V$OLF1.02	+12687	(-)	tccttcTCCCtggaggttaatgc	1.0	0.896
V$NR2F	V$COUP.01	+12090	(-)	cggataaggacatAGGTagaggatg	1.0	0.827
	V$HNF4G.01	+13309	(-)	tgttttaaaccAAAGaccagttatt	1.0	0.940
	V$PNR.01	+12308	(+)	actggcagaTCAAaactaattctgg	1.0	0.843
V$OCT1	V$OCT1.01	+11296	(-)	taTATGggaatatgt	1.0	0.836
	V$OCT1.02	+11830	(+)	tttATGCaagaatcc	1.0	0.861

	V$OCT1.02	+12679	(-)	ttaATGCaaacacaa	1.0	0.952
	V$OCT1.03	+11548	(+)	acttcatcATTAtaa	1.0	0.853
	V$OCT1.03	+11640	(-)	cagccataATTAgca	1.0	0.917
	V$OCT1.03	+12172	(+)	catgatttATTAaat	1.0	0.856
	V$OCT1.03	+12864	(+)	tctggttaATTAtga	1.0	0.950
	V$OCT1.03	+12867	(-)	tcctcataATTAacc	1.0	0.927
	V$OCT1.05	+13258	(-)	aaCATGtaattttct	1.0	0.954
	V$OCT1.06	+11636	(+)	tacatgctAATTatg	1.0	0.945
	V$OCT1.06	+11910	(+)	gatctgatAATTtca	1.0	0.844
	V$OCT1.06	+12458	(+)	caaatacaAATTcta	1.0	0.816
	V$POU3F3.01	+11827	(-)	ttcttGCATaaacct	1.0	0.855
	V$POU3F3.01	+12682	(+)	tgtttGCATtaacct	1.0	0.814
V$OVOL	V$OVOL1.01	+12525	(+)	gtgactGTTActggg	1.0	0.827
	V$OVOL1.01	+12560	(+)	atctttGTTAgttgg	1.0	0.814
	V$OVOL1.01	+12895	(+)	atggctGTTAactat	1.0	0.843
	V$OVOL1.01	+12961	(-)	ataatgGTTAtctct	1.0	0.830
V$P53F	V$P53.02	+11710	(+)	tacaaggaagctggcCATGtcctga	1.0	0.977
	V$P53.02	+11719	(-)	cagatggtttcaggaCATGgccagc	1.0	0.935
V$PARF	V$DBP.01	+11372	(+)	atgtaTTATgtcagcta	1.0	0.858
	V$DBP.01	+11684	(+)	actggTTATgtagtact	1.0	0.853
	V$DBP.01	+11826	(+)	caggtTTATgcaagaat	1.0	0.929
	V$DBP.01	+13232	(+)	ctgtaTTATgaaattat	1.0	0.928
	V$TEF.01	+12021	(+)	acaatttctGTAAgcac	1.0	0.867
	V$TEF.01	+12072	(-)	atatattttGTAAtttg	1.0	0.919
	V$TEF_HLF.01	+12071	(+)	gcaaaTTACaaaatata	1.0	0.808
	V$VBP.01	+11696	(-)	gtatgatatGTAAgtac	1.0	0.880
	V$VBP.01	+13260	(-)	aaagaacatGTAAtttt	1.0	0.870
V$PAX3	V$PAX3.03	+11389	(+)	atcttCATGgctgaaggca	1.0	0.947
V$PAX6	V$PAX4_PD.01	+12164	(+)	acaGCACtcatgatttatt	1.0	0.911
V$PAX7	V$PAX7.01	+13296	(-)	ttaaaatGATTaaag	1.0	0.826
V$PAXH	V$PAX4.02	+11638	(-)	gccatAATTagcatg	1.0	0.919
	V$PAX4.02	+11639	(+)	atgctAATTatggct	1.0	0.918
	V$PAX4.02	+12865	(-)	ctcatAATTaaccag	1.0	0.911
	V$PAX4.02	+12866	(+)	tggttAATTatgagg	1.0	0.894
V$PDX1	V$IPF1.01	+11634	(+)	aatacatgcTAATtatggc	1.0	0.931
	V$IPF1.01	+11639	(-)	ggacagccaTAATtagcat	1.0	0.943
	V$IPF1.01	+12861	(+)	gcttctggtTAATtatgag	1.0	0.924
	V$IPF1.01	+12866	(-)	gcttcctcaTAATtaacca	1.0	0.914
	V$PDX1.01	+12486	(+)	ttacaaattTAATcactgg	1.0	0.831
V$PERO	V$PPARG.03	+13122	(-)	atcacaaaatctAAAGgtaagca	1.0	0.857
V$PIT1	V$PIT1.02	+12175	(-)	gatatTTAAtaaatc	1.0	0.826
	V$PIT1.02	+12176	(+)	atttaTTAAatatct	1.0	0.824
V$PLZF	V$PLZF.01	+13288	(+)	atgTACAgctttaat	1.0	0.877
	V$PLZF.02	+11201	(+)	tctTACAgtgctatt	1.0	0.890
	V$PLZF.02	+13225	(-)	taaTACAgtacctag	1.0	0.912
V$PRDF	V$PRDM1.01	+12253	(-)	ttccagtGAAAtgactcta	1.0	0.834
V$PROX	V$PROX1.01	+11485	(-)	aaaGACGggttcc	1.0	0.839
V$RBP2	V$PLU1_JARID1B.01	+12034	(+)	GCACagaac	1.0	0.963
V$RBPF	V$RBPJK.01	+11479	(+)	ggccTGGGaaccc	1.0	0.896
	V$RBPJK.02	+11298	(-)	tataTGGGaatat	1.0	0.946
V$RORA	V$REV-ERBA.01	+12705	(+)	aaggaacaggGTCAaaaagctaaac	1.0	0.916
V$RP58	V$RP58.01	+11460	(-)	tgatCATCtgtag	1.0	0.853
V$RREB	V$RREB1.01	+11601	(-)	cCCCAtcctagccca	1.0	0.809
V$RU49	V$RU49.01	+11695	(-)	aAGTAct	1.0	0.989
	V$RU49.01	+11791	(+)	cAGTAcc	1.0	0.994
	V$RU49.01	+13228	(-)	cAGTAcc	1.0	0.994
V$RUSH	V$SMARCA3.01	+11302	(+)	tcCCATataat	1.0	0.984
	V$SMARCA3.01	+11796	(-)	tgCCATttagg	1.0	0.985
	V$SMARCA3.02	+12413	(-)	aagcACTTttt	1.0	0.993
	V$SMARCA3.02	+12673	(+)	gaggACTTgtg	1.0	0.986
V$RXRF	V$RARG.01	+11204	(+)	tacagtgctatTTTGaattttagta	1.0	0.851
	V$RARG.01	+11919	(-)	gggaatgagcaTTTGaatgtgaaat	1.0	0.855
	V$THRA.01	+12915	(+)	tggcttgcctttaccAGGTattgaa	1.0	0.838
	V$VDR_RXR.04	+12706	(+)	aggaacagGGTCaaaaagctaaact	1.0	0.821
V$SATB	V$SATB1.01	+12171	(-)	tttAATAaatcatga	1.0	0.963
	V$SATB1.01	+12280	(+)	tctAATAatgctaga	1.0	0.956
V$SF1F	V$SF1.01	+11862	(-)	ggagCAAGgtgacag	1.0	0.953

V$SMAD	V$SMAD.01	+12551	(-)	attGTCTgtgt	1.0	0.966
	V$SMAD4.01	+12015	(-)	attGTCTaggc	1.0	0.995
V$SORY	V$HBP1.01	+11926	(-)	gtggagggAATGagcatttgaat	1.0	0.922
	V$HBP1.01	+12823	(-)	acagcaagAATGagataaggtag	1.0	0.895
	V$HBP1.02	+13063	(+)	acagatgAATGgaaatatgatcg	1.0	0.886
	V$HBP1.02	+13197	(-)	ttttctgAATGgaaagcaaataa	1.0	0.880
	V$HMGA.01	+12861	(+)	gcttctggttAATTatgaggaag	1.0	0.882
	V$HMGIY.01	+13204	(-)	aggaAATTtttctgaatggaaag	1.0	0.927
	V$SOX1.04	+12538	(+)	ggtGAATcaaaatacacagacaa	1.0	0.885
	V$SOX18.02	+12724	(-)	aaccaatgaaATTGctagtttag	1.0	0.854
	V$SOX21.03	+11215	(+)	tttGAATtttagtaaaattttaa	1.0	0.849
	V$SOX21.03	+13194	(-)	tctGAATggaaagcaaataaatc	1.0	0.828
	V$SOX3.01	+12499	(-)	caaccaCAAAtgatcttccagtg	1.0	0.946
	V$SOX3.03	+12730	(+)	tagCAATttcattggttctggct	1.0	0.815
	V$SOX30.01	+13084	(+)	cgaagACAAtgaggcaaaaaatt	1.0	0.922
	V$SOX5.01	+12431	(-)	aatataCAATgttgtcatctctg	1.0	0.872
	V$SOX5.02	+12046	(+)	cccgtACAAtaatgcccgtctca	1.0	0.877
	V$SOX6.01	+12550	(-)	aactaACAAagattgtctgtgta	1.0	0.976
	V$SOX7.01	+13102	(-)	gcaagACAAtagatgttgaattt	1.0	0.927
	V$SOX7.03	+11847	(+)	aAACAgtctcagtgtctgtcacc	1.0	0.811
V$SP1F	V$SP1.01	+11744	(-)	cttagGGGCaggcatct	1.0	0.880
V$SPZ1	V$SPZ1.01	+11937	(-)	tGGAGggaatg	1.0	0.960
V$SREB	V$SREBP.04	+11607	(-)	gccTCACcccatcct	1.0	0.952
V$STAT	V$STAT.01	+11758	(+)	aaggttagtGGAActgtct	1.0	0.908
	V$STAT.01	+12843	(+)	tgttttagaGGAActaggg	1.0	0.924
	V$STAT6.01	+12257	(-)	aactTTCCagtgaaatgac	1.0	0.919
	V$STAT6.01	+13218	(+)	aaatTTCCtaggtactgta	1.0	0.850
V$STEM	V$OCT3_4.01	+12279	(-)	atctctaGCATtattagaa	1.0	0.822
	V$OCT3_4.02	+12680	(+)	tgtgtttGCATtaacctcc	1.0	0.924
	V$OSNT.01	+11924	(-)	ggaatgaGCATttgaatgt	1.0	0.891
V$TAIP	V$CSRNP1.01	+12578	(+)	AGAGtgc	1.0	1.000
	V$CSRNP1.01	+13001	(+)	AGAGtgg	1.0	1.000
V$TALE	V$PKNOX2.01	+11855	(+)	tcagtgtctGTCAcctt	1.0	0.894
V$TCFF	V$TCF11.01	+12257	(+)	GTCAttt	1.0	1.000
V$TEAF	V$TEAD.01	+11934	(+)	gctCATTccctcc	1.0	0.927
	V$TEAD.01	+12831	(+)	tctCATTcttgct	1.0	0.905
	V$TEF1.01	+11922	(+)	tcaCATTcaaatg	1.0	0.852
	V$TEF1.01	+12516	(-)	tcaCATTcaacca	1.0	0.843
V$XBBF	V$RFX3.03	+11310	(+)	aattTTGCtaagcatcatg	1.0	0.825
	V$RFX5.01	+12366	(-)	tgcaggactaAGCAacagt	1.0	0.976
	V$RFX5.01	+12790	(+)	tttacaacttAGCAacaag	1.0	0.980
V$YBXF	V$YB1.01	+11169	(+)	aagaaTGGCctac	1.0	0.909
V$YBXF	V$YB1.01	+13000	(+)	cagagTGGCtcta	1.0	0.926
V$YY1F	V$REX1.01	+11160	(-)	agtaggCCATtctttattcaaaa	1.0	0.880
	V$YY1.02	+11718	(+)	agctggCCATgtcctgaaaccat	1.0	0.949
	V$YY1.02	+11788	(-)	tttgtgCCATttaggtactgtcc	1.0	0.951
	V$YY1.02	+11882	(-)	tgaagtCCATctcttgattgcag	1.0	0.944
	V$YY2.02	+11962	(-)	tgactaCCATtgcagaagcagag	1.0	0.854
V$ZF03	V$ZNF217.01	+11252	(+)	GAATcttggctat	1.0	0.909
V$ZF05	V$ZFP410.01	+11277	(-)	atttatgGGATaatc	1.0	0.876
V$ZF07	V$ZNF263.01	+11937	(+)	cattccCTCCacctc	1.0	0.924
	V$ZNF263.01	+12191	(+)	gctctcCTCCtccca	1.0	0.951
	V$ZNF263.02	+12194	(+)	ctcctcCTCCcacaa	1.0	0.971
V$ZF10	V$PRDM14.01	+12249	(+)	ttgTTAGagtcattt	1.0	0.875
	V$PRDM14.01	+13002	(-)	ggtTTAGagccactc	1.0	0.874
V$ZF11	V$ZBTB3.01	+11949	(+)	ctcaGCCAgcc	1.0	0.991
V$ZF12	V$ZFP652.01	+12333	(+)	ttgaaatggTTAAtg	1.0	0.908
	V$ZFP652.01	+13296	(-)	ttaaaatgaTTAAag	1.0	0.805
	V$ZNF652.02	+13180	(-)	cagaatGGGTtcaaa	1.0	0.844
V$ZF57	V$ZFP57.01	+13090	(-)	tttTGCCtcattg	1.0	0.847
V$ZFHX	V$AREB6.01	+11823	(-)	cataaACCTgtgt	1.0	0.943
V$ZFXY	V$ZFX.01	+11477	(+)	caGGCCtggga	1.0	0.982

Intron 5

O$INRE	O$DINR.01	15493	(+)	ttTCAGtagag	1.0	0.940
	O$DINR.01	15747	(+)	ttTCAGttgac	1.0	1.000

O$PTBP	O$PTATA.01	15786	(-)	acaaTATAtacatat	1.0	0.884
	O$PTATA.02	13892	(+)	agttTATAaattttt	1.0	0.904
	O$PTATA.02	15764	(-)	gcacTATAgaaacaa	1.0	0.912
O$VTBP	O$ATATA.01	15241	(-)	ggatacaTAAGtgaagc	1.0	0.837
	O$LTATA.01	13760	(+)	gaaTATAatctttaaca	1.0	0.823
	O$MTATA.01	13553	(+)	cactcTAAAaccccaaa	1.0	0.840
	O$MTATA.01	14698	(+)	tgcttTAAAtatacatg	1.0	0.844
	O$MTATA.01	15268	(-)	tttttTAAAgcagcaaa	1.0	0.842
	O$VTATA.01	13893	(+)	gtttaTAAAtttttaaa	1.0	0.915
	O$VTATA.02	13875	(-)	tttcaTAAAactttaat	1.0	0.897
	O$VTATA.02	15198	(-)	ctgcaTAAAaacttaaa	1.0	0.904
	O$VTATA.02	15273	(+)	tgcttTAAAaaaaaaac	1.0	0.909
O$YTBP	O$SPT15.01	13512	(+)	ctactatTATAtattga	1.0	0.858
	O$SPT15.01	13515	(-)	ttatcaaTATAtaatag	1.0	0.916
	O$SPT15.01	13528	(-)	tgagaaaTATAcattat	1.0	0.854
	O$SPT15.01	15784	(+)	aaatatgTATAtattgt	1.0	0.852
	O$SPT15.01	15786	(+)	atatgtaTATAttgtgg	1.0	0.843
	O$SPT15.01	15787	(-)	tccacaaTATAtacata	1.0	0.878
V$AARF	V$AARE.01	14365	(+)	gTTTCacca	1.0	0.953
	V$AARE.01	14521	(-)	tTTTCatca	1.0	0.979
V$ABDB	V$HOXB9.02	13583	(-)	agaggtgaTAAAaactc	1.0	0.899
	V$HOXB9.02	15230	(-)	tgaagccaTAAAggtgg	1.0	0.887
	V$HOXC13.01	15255	(+)	tccttctaaTAAAtttg	1.0	0.976
	V$HOXC9.01	15716	(+)	gtttgaaaTTAAtggac	1.0	0.832
	V$HOXC9.01	15719	(-)	aatgtccaTTAAtttca	1.0	0.843
	V$HOXD10.01	13878	(-)	acttttcaTAAAacttt	1.0	0.920
	V$HOXD13.01	14001	(-)	cttacaaaTAAAaagcg	1.0	0.927
	V$HOXD13.01	14065	(+)	gtggtgaaTAAAacagc	1.0	0.910
V$AHRR	V$AHRARNT.03	14306	(+)	gggattatagGCGTgcgctaccatg	1.0	0.970
	V$NXF_ARNT.01	15163	(+)	gggattacaggCGTGagccaccaca	1.0	0.913
	V$NXF_ARNT.01	15583	(+)	gggattacaggCGTGagccaccccg	1.0	0.904
V$AP1R	V$MAFF.01	13636	(-)	cagcctGCTGagcctggtcccct	1.0	0.856
	V$NRL.02	13640	(+)	gaccaggctcAGCAggctgagaa	1.0	0.991
V$AP4R	V$AP4.01	14073	(+)	taaaaCAGCtggagtgt	1.0	0.862
	V$AP4.03	14072	(-)	cactccaGCTGttttat	1.0	0.994
	V$AP4.03	15810	(+)	aaatccaGCTGacatat	1.0	0.978
V$ARID	V$ARID5A.01	14146	(-)	taacaATATtctgatacgaaa	1.0	0.938
	V$ARID5A.01	14153	(+)	tcagaATATtgttacttaata	1.0	0.953
	V$BRIGHT.01	13870	(+)	ctaaaATTAaagttttatgaa	1.0	0.926
	V$JARID2.01	14558	(-)	cagtatTTTAgtaaaatcttt	1.0	0.894
V$ATBF	V$ATBF1.01	15712	(+)	actggtttgaAATTaat	1.0	0.879
V$BARB	V$BARBIE.01	13996	(-)	ataaAAAGcgtgctg	1.0	0.915
	V$BARBIE.01	15227	(-)	ccatAAAGgtggagg	1.0	0.906
V$BCDF	V$CRX.03	14301	(-)	gcctataATCCcagcta	1.0	0.961
	V$CRX.03	14437	(-)	gcctgtaATCCtcccag	1.0	0.969
	V$OTX1.01	15158	(-)	gcctgtAATCccaacac	1.0	0.882
	V$PCE1.01	14024	(-)	ggatcTAATttgttgaa	1.0	0.895
V$BCL6	V$BCL6.02	13740	(-)	tcatttcTAGAaaggct	1.0	0.894
	V$BCL6.02	13741	(+)	gcctttcTAGAaatgaa	1.0	0.882
	V$BCL6.02	14617	(+)	agtcttcTAGAaatctt	1.0	0.828
	V$BCL6.02	14649	(+)	agtcttcTAGAaatctt	1.0	0.828
	V$BCL6.02	14681	(+)	agtcttcTAGAaatctt	1.0	0.828
	V$BCL6.04	14602	(+)	tgcTTCCtagaagcaag	1.0	0.983
	V$BCL6.04	14634	(+)	tgcTTCCtagaagcaag	1.0	0.983
	V$BCL6.04	14666	(+)	tgcTTCCtagaagcaag	1.0	0.983
V$BEDF	V$ZBED4.01	14406	(-)	ggtGGGCggatcacg	1.0	0.949
	V$ZBED4.01	15130	(-)	ggcGGGCggatcttg	1.0	0.940
V$BEDF	V$ZBED4.01	15599	(-)	gctGGGCggggtggc	1.0	0.951
V$BHLH	V$MESP1_2.01	15818	(+)	ctgaCATAtgcat	1.0	0.916
	V$MESP1_2.01	15819	(-)	aatgCATAtgtca	1.0	0.946
V$BNCF	V$BNC.01	15725	(-)	agggaaaaaaTGTCcatta	1.0	0.892
V$BPTF	V$FAC1.01	15309	(-)	gcaacAACAaa	1.0	0.978
V$BRAC	V$TBX20.02	14966	(-)	ttgagcctgggaggttGACAggctgcaat	1.0	0.898
V$BRN5	V$BRN5.04	14804	(+)	tgccaaaactgATTAtcatcaat	1.0	0.922
V$BRNF	V$BRN2.01	13517	(-)	taCATTatcaatatataat	1.0	0.866
	V$BRN2.02	15824	(+)	tatgcattaccTCATgtaa	1.0	0.832
	V$BRN3.02	15254	(+)	atccttcTAATaaatttgc	1.0	0.907
	V$BRN3.02	15715	(-)	tgtccatTAATttcaaacc	1.0	0.913

	V$BRN3.03	15718	(+)	ttgaaatTAATggacattt	1.0	0.842
V$BTBF	V$KAISO.01	13806	(+)	tataCTGCgac	1.0	0.922
V$CABL	V$CABL.01	14899	(-)	aaAACAaccaa	1.0	0.976
	V$CABL.01	15319	(-)	aaAACAaaaag	1.0	0.997
V$CART	V$ALX3.01	15470	(-)	acaaaaAATTagctgattgtg	1.0	0.852
	V$ISX.01	14020	(-)	ggatctAATTtgttgaagcag	1.0	0.932
	V$ISX.01	14025	(+)	tcaacaAATTagatccacagc	1.0	0.930
	V$PHOX2.01	15256	(+)	ccttcTAATaaatttgctgct	1.0	0.892
	V$PHOX2.01	15720	(+)	gaaatTAATggacatttttc	1.0	0.877
	V$PROP1.01	13897	(-)	tttgttAATTtaaaaatttat	1.0	0.862
	V$PROP1.01	13902	(+)	tttttaAATTaacaaagtttc	1.0	0.857
V$CDXF	V$CDX1.01	13876	(+)	ttaaagtTTTAtgaaaagt	1.0	0.949
	V$CDX1.01	13999	(+)	cacgcttTTTAtttgtaag	1.0	0.941
	V$CDX1.01	14557	(+)	gaaagatTTTActaaaata	1.0	0.953
	V$CDX1.01	14561	(-)	acagtatTTTAgtaaaatc	1.0	0.953
	V$CDX1.02	13581	(+)	gagagttTTTAtcacctct	1.0	0.929
	V$CDX2.02	13890	(-)	ttaaaaatTTATaaacttt	1.0	0.854
	V$CDX2.02	15256	(-)	cagcaaatTTATtagaagg	1.0	0.855
	V$CDX2.02	15736	(+)	ttttccctTTATttcagtt	1.0	0.864
	V$CDX2.03	13786	(+)	aaaagtctTTATggtacag	1.0	0.956
	V$CDX2.03	15228	(+)	ctccacctTTATggcttca	1.0	0.958
V$CEBP	V$CEBPB.01	13880	(+)	agttttatGAAAagt	1.0	0.946
	V$CEBPB.01	15087	(-)	aacatggtGAAAtcc	1.0	0.945
	V$CEBPD.01	15793	(+)	tatatTGTGgaatgc	1.0	0.982
	V$CEBPE.02	14800	(-)	cagttttgGCAAggg	1.0	0.974
V$CHRF	V$CHR.01	15714	(+)	tggtTTGAaatta	1.0	0.952
V$CIZF	V$NMP4.01	13783	(+)	gcAAAAagtct	1.0	0.983
	V$NMP4.01	15731	(-)	ggAAAAaatgt	1.0	0.989
V$CREB	V$CREB1.02	15823	(-)	gttacATGAggtaatgcatat	1.0	0.937
	V$E4BP4.01	14157	(-)	gtgctattaaGTAAcaatatt	1.0	0.937
	V$E4BP4.01	14524	(-)	tttctattctGTAAattttca	1.0	0.827
	V$E4BP4.01	15829	(+)	attacctcatGTAAcagttta	1.0	0.879
V$DLXF	V$DLX3.01	15717	(-)	aatgtccatTAATttcaaa	1.0	0.916
	V$DLX3.02	14026	(-)	ctgtggatcTAATttgttg	1.0	0.931
	V$DLX3.02	15050	(+)	acgtgtggcTAATttttgt	1.0	0.941
V$DMRT	V$DMRT1.01	13805	(+)	ttatactgcgactTTGTtgct	1.0	0.852
	V$DMRT4.01	15830	(-)	ataaactgtTACAtgaggtaa	1.0	0.847
	V$DMRT5.01	14156	(+)	gaatattGTTActtaatagca	1.0	0.834
	V$DMRT5.01	15631	(+)	ctgtaaaGTTActttctctgg	1.0	0.834
	V$DMRT7.01	13504	(+)	agttATTGctactattatata	1.0	0.845
	V$DMRT7.01	13946	(-)	tcgaATTGcaactttgtgagg	1.0	0.837
	V$DMRT7.01	14725	(+)	agatATTGtcaaattgctgct	1.0	0.820
	V$DMRT7.01	15759	(+)	aaaaATTGtttctatagtgca	1.0	0.811
V$DMTF	V$DMP1.01	14378	(+)	ggccaGGATggtctt	1.0	0.825
	V$DMP1.01	15688	(-)	gcccaGGATgtccaa	1.0	0.867
	V$DMP1.02	13938	(-)	tgtgaGGATgggggа	1.0	0.906
V$E2FF	V$E2F.02	15346	(-)	agcctgggcGAAAgagt	1.0	0.849
	V$E2F1.01	15597	(-)	gctgGGCGgggtggctc	1.0	0.974
	V$E2F4.01	15455	(-)	tggtgGCGGgcaccttg	1.0	0.968
V$EBOX	V$ATF6.01	13665	(-)	tgtgCCACtcctggcca	1.0	0.952
	V$ATF6.01	13719	(+)	gctgCCACccctctgag	1.0	0.933
	V$CMYC.02	14399	(+)	ttgacctCGTGatccgc	1.0	0.924
	V$MNT.01	15043	(-)	agccaCACGtgggtggc	1.0	0.986
	V$MYCMAX.01	14398	(-)	cggatCACGaggtcaag	1.0	0.845
	V$NMYC.01	15044	(+)	ccacccaCGTGtggcta	1.0	0.995
V$EGRF	V$EGR2.02	14991	(-)	aagTGGGaggatcgtttga	1.0	0.935
V$ESRR	V$ESRRB.01	15540	(-)	cgggtggatcacaAGGTcaggag	1.0	0.978
V$ETSF	V$ERG.02	13911	(-)	aaaagataGGAAactttgtta	1.0	0.941
	V$PDEF.01	14375	(+)	gttggccaGGATggtcttgat	1.0	0.953
	V$PDEF.01	15519	(+)	gttagccaGGATggtctcgat	1.0	0.953
	V$PDEF.01	15685	(-)	tgagcccaGGATgtccaacat	1.0	0.963
	V$SPI1.02	13928	(-)	aggatgggGGAAggagtaaaa	1.0	0.961
V$EVI1	V$EVI1.02	13614	(-)	gggagAAGAtaaactgg	1.0	0.949
	V$EVI1.04	14140	(-)	ctgatacgaaaaGATAt	1.0	0.934
	V$EVI1.05	14135	(-)	acgaaaaGATAtccacc	1.0	0.890
	V$EVI1.06	13918	(-)	agtaaaAGATaggaaac	1.0	0.836
	V$MEL1.02	13958	(+)	gcaattcGATGagtatc	1.0	0.996
	V$MEL1.03	13703	(-)	cagtgatGATGgagagg	1.0	0.957

	V$MEL1.03	13937	(-)	ttgtgagGATGggggaa	1.0	0.989
V$FAST	V$FAST1.01	13527	(+)	gataatgtaTATTtctc	1.0	0.852
	V$FAST1.01	14701	(-)	aaacatgtaTATTtaaa	1.0	0.848
	V$FAST1.02	15793	(+)	tatatTGTGgaatgcct	1.0	0.835
V$FKHD	V$FHXB.01	13877	(-)	cttttcATAAaacttta	1.0	0.886
	V$FOXP1.02	15305	(-)	aagcaacAACAaaaaaa	1.0	1.000
	V$FOXP1_ES.01	13822	(+)	tgcttaaAACAcagttg	1.0	1.000
	V$FOXP1_ES.01	14069	(+)	tgaataaAACAgctgga	1.0	1.000
	V$FOXP1_ES.01	14855	(-)	aaagaaaAACAcgaaaa	1.0	1.000
	V$FOXP1_ES.01	14870	(-)	ggtctaaAACAcctaaa	1.0	1.000
	V$FOXP1_ES.01	14898	(-)	gtctcaaAACAaccaaa	1.0	1.000
	V$FOXP1_ES.01	15318	(-)	tcaaaaaAACAaaaagc	1.0	1.000
	V$FOXP2.01	14708	(-)	aaattgtaAACAtgtat	1.0	0.986
	V$HFH1.01	15839	(-)	tatacaTAAActgttac	1.0	0.860
V$GATA	V$GATA.01	14146	(-)	ttctGATAcgaaa	1.0	0.949
	V$GATA1.03	13585	(-)	aggtGATAaaaac	1.0	0.951
	V$GATA1.03	13615	(-)	agaaGATAaactg	1.0	0.961
	V$GATA1.03	13919	(-)	aaaaGATAggaaa	1.0	0.957
	V$GATA1.06	14883	(-)	ttctGATAtggtc	1.0	0.970
	V$GATA1.06	15373	(-)	ccgaGATAgtgcc	1.0	0.968
	V$GATA1.06	15669	(+)	aaatGATAaggat	1.0	0.970
	V$GATA2.01	13523	(+)	tattGATAatgta	1.0	0.927
	V$GATA3.02	13761	(-)	taaAGATtatatt	1.0	0.951
	V$GATA3.02	13842	(+)	gagAGATgaaaac	1.0	0.916
	V$GATA3.02	14557	(+)	gaaAGATtttact	1.0	0.969
	V$GATA4.01	14495	(+)	atgtGATAattta	1.0	0.927
	V$GATA4.01	14812	(-)	tgatGATAatcag	1.0	0.921
V$GCMF	V$GCM1.03	13934	(+)	tccttCCCCcatcct	1.0	0.855
V$GFI1	V$GFI1.01	15080	(-)	tgaAATCccatctct	1.0	0.963
	V$GFI1.02	14593	(+)	acaAATCtttgcttc	1.0	0.932
	V$GFI1.02	14625	(+)	agaAATCtttgcttc	1.0	0.923
	V$GFI1.02	14657	(+)	agaAATCtttgcttc	1.0	0.923
	V$GFI1.02	14689	(+)	agaAATCtttgcttt	1.0	0.923
V$GLIF	V$GLI1.01	14977	(+)	tcaacctCCCAggctca	1.0	0.888
	V$GLI1.02	15027	(+)	gagaCCACagatgtatg	1.0	0.821
	V$GLI3.02	15040	(+)	tatgCCACccacgtgtg	1.0	0.888
V$GRHL	V$GRHL1.01	14504	(-)	ggtactGGTTaaa	1.0	0.895
	V$GRHL2.01	13610	(+)	ctAACCagtttat	1.0	0.935
	V$GRHL2.01	15708	(-)	caAACCagtctgg	1.0	0.881
	V$GRHL3.01	13609	(-)	taaactGGTTagg	1.0	0.923
	V$GRHL3.01	15709	(+)	cagactGGTTtga	1.0	0.889
V$HAML	V$AML1.01	14113	(-)	gactGTGGtccttat	1.0	0.946
	V$AML1.01	15024	(-)	atctGTGGtctccgc	1.0	0.972
	V$AML1.02	15176	(-)	gggtGTGGtggctca	1.0	0.960
	V$AML2.01	15462	(-)	gatTGTGgtggcggg	1.0	0.945
V$HAND	V$TAL1_E2A.02	13642	(+)	ccaggctCAGCaggctgagaa	1.0	0.987
	V$TAL1BETAE47.01	15027	(+)	gagaccaCAGAtgtatgccac	1.0	0.872
	V$TWIST.01	15041	(-)	ttagccaCACGtgggtggcat	1.0	0.943
V$HBOX	V$GBX1.01	14024	(+)	ttcaacaAATTagatccac	1.0	0.897
	V$GBX2.01	15053	(+)	tgtggctAATTtttgtatt	1.0	0.830
	V$GSH1.01	15718	(-)	aaatgtccatTAATttcaa	1.0	0.870
	V$GSH2.01	14023	(-)	tggatcTAATttgttgaag	1.0	0.986
	V$MEOX1.01	14494	(-)	ctggttaAATTatcacata	1.0	0.842
	V$MEOX1.01	15474	(+)	atcagctAATTttttgtat	1.0	0.840
V$HEAT	V$HSF1.01	13736	(-)	cttttcatttctAGAAaggcttaaa	1.0	0.859
V$HESF	V$DEC2.01	15044	(-)	gccacaCGTGggtgg	1.0	0.974
	V$DEC2.01	15045	(+)	cacccaCGTGtggct	1.0	0.980
V$HICF	V$HIC1.01	13718	(+)	tgcTGCCacccct	1.0	0.912
	V$HIC1.01	15039	(+)	gtaTGCCacccac	1.0	0.965
V$HIFF	V$HIF1.02	15044	(-)	tagccacaCGTGggtgg	1.0	0.980
	V$HRE.02	15043	(+)	gccacccaCGTGtggct	1.0	0.972
V$HMTB	V$MTBF.01	15300	(+)	tggaATTTt	1.0	0.923
V$HNF1	V$HMBOX.01	13606	(-)	ataaactgGTTAggcac	1.0	0.905
	V$HMBOX.01	14501	(-)	gggtactgGTTAaatta	1.0	0.934
	V$HMBOX.01	14577	(-)	taaggataGTTAttaca	1.0	0.842
	V$HNF1.01	13760	(-)	tGTTAaagattatattc	1.0	0.828
	V$HNF1.01	14162	(+)	tGTTActtaatagcact	1.0	0.856
	V$HNF1.04	13767	(-)	cctttttGTTAaagat	1.0	0.878

	V$HNF1.04	13906	(-)	gaaactttGTTAattta	1.0	0.860
	V$HNF1.04	15382	(-)	agcttgaaGTTAgccga	1.0	0.857
	V$HNF1.04	15651	(-)	tgggaccaGTTAagatc	1.0	0.849
V$HNF6	V$HNF6.02	13520	(-)	atacattatCAATatat	1.0	0.937
	V$HNF6.02	14814	(+)	gattatcatCAATcctt	1.0	0.915
V$HNFP	V$MIZF.01	14243	(-)	ggCGGAggttgtagtgagc	1.0	0.841
V$HOMF	V$BARHL1.01	15473	(+)	aatcagcTAATtttttgta	1.0	0.878
	V$BARX1.01	15474	(-)	atacaaaaAATTagctgat	1.0	0.911
	V$HHEX.01	13871	(-)	tcataaaacttTAATttta	1.0	0.971
	V$HHEX.01	14158	(+)	atattgttactTAATagca	1.0	0.967
	V$HMX1.01	14162	(+)	tgttactTAATagcacttt	1.0	0.824
	V$HMX2.02	13853	(-)	agaataaaAAACctttagt	1.0	0.840
	V$HMX2.02	13886	(-)	aaatttatAAACttttcat	1.0	0.843
	V$HMX2.02	14985	(+)	ccaggctcAAACgatcctc	1.0	0.860
	V$HMX2.03	15649	(+)	tggatcTTAActggtccca	1.0	0.841
	V$HMX3.01	14999	(-)	ggaggctcAAGTgggagga	1.0	0.971
	V$HMX3.02	14163	(-)	aaaagtgctaTTAAgtaac	1.0	0.934
	V$HMX3.02	15645	(+)	tctctggatcTTAActggt	1.0	0.927
	V$HMX3.02	15650	(-)	gtgggaccagTTAAgatcc	1.0	0.927
	V$HMX3.02	15714	(+)	tggtttgaaaTTAAtggac	1.0	0.931
	V$HMX3.02	15719	(-)	aaaatgtccaTTAAtttca	1.0	0.938
	V$LBX2.01	13900	(+)	aattttaAATTaacaaag	1.0	0.895
	V$MSX.01	13867	(-)	aaaacttTAATtttagaat	1.0	0.989
	V$MSX.01	14024	(-)	gtggatcTAATttgttgaa	1.0	0.994
	V$MSX.01	15715	(-)	tgtccatTAATttcaaacc	1.0	0.972
	V$NOBOX.02	14023	(+)	cttcaacaAATTagatcca	1.0	0.909
	V$TLX2.01	13901	(-)	actttgTTAAtttaaaaat	1.0	0.840
V$HOXC	V$HOXB4.02	13701	(-)	gtgaTGATggagaggtg	1.0	0.871
	V$MEIS1.03	15803	(-)	agctgGATTtaggcatt	1.0	0.879
	V$PBX1.01	14816	(-)	gtaagGATTgatgataa	1.0	0.851
V$HOXF	V$HOXA1.01	15472	(-)	acaaaaAATTagctgattg	1.0	0.838
	V$HOXB4.01	13899	(-)	tttgttAATTtaaaaattt	1.0	0.862
	V$HOXB8.01	14161	(-)	aagtgctATTAagtaacaa	1.0	0.859
	V$HOXB8.01	15716	(+)	gtttgaaATTAatggacat	1.0	0.909
	V$HOXC4.01	13902	(+)	tttttaAATTaacaaagtt	1.0	0.864
	V$HOXD3.01	14493	(-)	tggttaAATTatcacatat	1.0	0.859
	V$HOXD3.01	14496	(+)	tgtgatAATTtaaccagta	1.0	0.867
	V$NANOG.01	15719	(+)	tgaaattAATGgacatttt	1.0	0.948
V$HZIP	V$HOMEZ.01	14269	(-)	ggagcATCGcttgaa	1.0	0.835
	V$HOMEZ.01	14990	(-)	ggaggATCGtttgag	1.0	0.877
V$IKRS	V$IK2.01	14422	(-)	ctttGGGAagctg	1.0	0.988
	V$IK2.01	14775	(+)	tattGGGAagagt	1.0	0.990
	V$IK2.01	15159	(+)	tgttGGGAttaca	1.0	0.980
	V$IK3.01	13971	(-)	tactgGGAAgata	1.0	0.843
V$IKZF	V$PEGASUS.01	14061	(+)	tagagtGGTGaat	1.0	0.966
V$IRFF	V$IRF1.01	14474	(-)	acatatctaaaaatGAAAtactact	1.0	0.889
	V$IRF1.01	15503	(-)	ctggctaacacagtGAAAccccatc	1.0	0.878
V$IRXF	V$IRX5.01	14706	(-)	taaaCATGtatat	1.0	0.974
V$IRXF	V$IRX5.01	14707	(+)	tataCATGtttac	1.0	0.974
V$KLFS	V$EKLF.01	14040	(+)	cacagcatttaGGGTtgac	1.0	0.893
	V$GKLF.03	15177	(-)	agcctgggtGTGGtggctc	1.0	0.988
	V$KKLF.01	13678	(-)	aaaggaactGGGGagtgtg	1.0	0.916
	V$KLF2.01	13715	(-)	cagagGGGTggcagcagtg	1.0	0.987
	V$KLF7.01	15597	(-)	atgctgGGCGgggtggctc	1.0	0.966
	V$KLF7.02	14404	(-)	gaggtgGGCGgatcacgag	1.0	0.919
	V$KLF7.02	15128	(-)	aaggcgGGCGgatcttgag	1.0	0.905
	V$KLF7.02	15344	(-)	agcctgGGCGaaagagtga	1.0	0.880
V$LEFF	V$LEF1.01	13811	(-)	taagcaaCAAAgtcgca	1.0	0.871
	V$LEF1.04	13518	(-)	acattaTCAAtatataa	1.0	0.851
	V$LEF1.04	14816	(+)	ttatcaTCAAtccttac	1.0	0.917
V$LHXF	V$ISL1.01	14435	(-)	tcatgcctgTAATcctcccagca	1.0	0.820
	V$ISL1.01	15716	(+)	gtttgaaatTAATggacattttt	1.0	0.848
	V$ISL2.01	14021	(+)	tgcttcaacaaATTAgatccaca	1.0	0.873
	V$ISL2.01	15253	(-)	gcagcaaatttATTAgaaggata	1.0	0.876
	V$ISL2.01	15717	(-)	aaaaaatgtccATTAatttcaaa	1.0	0.879
	V$LHX1.01	13898	(+)	taaatttttaAATTaacaaagtt	1.0	0.861
	V$LHX2.01	14022	(-)	ctgtggatctAATTtgttgaagc	1.0	0.886
	V$LHX3.01	13865	(-)	ataaaactTTAAttttagaataa	1.0	0.818

	V$LHX3.01	15713	(-)	aatgtccaTTAAtttcaaaccag	1.0	0.842
	V$LHX3.02	13869	(-)	tttcataaaacttTAATtttaga	1.0	0.827
	V$LHX3.02	14026	(-)	aatgctgtggatcTAATttgttg	1.0	0.869
	V$LHX6.01	14805	(-)	gattgatgaTAATcagttttggc	1.0	0.841
	V$LMX1A.01	13899	(-)	aaactttgttAATTtaaaaattt	1.0	0.865
V$LTFM	V$LACTOFERRIN.01	13569	(+)	aGCACttga	1.0	0.923
V$LTSM	V$LTSM.02	14383	(-)	agatcaagacCATCc	1.0	0.970
	V$LTSM.02	15527	(-)	agatcgagacCATCc	1.0	0.974
	V$LTSM.03	15812	(+)	ATCCagctgacatat	1.0	0.860
V$MEF3	V$SIX2.02	14418	(+)	accTCAGcttccc	1.0	0.960
	V$SIX2.02	15284	(-)	tatTCAGgttttt	1.0	0.956
V$MOKF	V$MOK2.02	15452	(-)	gtggtggcgggcaCCTTgtag	1.0	0.983
V$MYBL	V$MYBL1.02	13827	(-)	tctctctgccAACTgtgtttt	1.0	0.863
V$MYOD	V$MYOD.02	14073	(-)	acactcCAGCtgtttta	1.0	0.942
	V$MYOGENIN.03	14072	(+)	ataaaaCAGCtggagtg	1.0	0.965
V$MYT1	V$MYT1.02	13876	(+)	ttaAAGTtttatg	1.0	0.990
	V$MYT1.02	13888	(+)	gaaAAGTttataa	1.0	0.992
	V$MYT1.02	13913	(+)	acaAAGTttccta	1.0	0.981
	V$MYT1.02	13950	(+)	acaAAGTtgcaat	1.0	0.882
	V$MYT1.02	14333	(-)	caaAAGTtagctg	1.0	0.883
	V$MYT1.02	14889	(-)	caaAAGTtctgat	1.0	0.901
	V$MYT1.02	15198	(+)	tttAAGTttttat	1.0	0.890
	V$MYT1L.01	13580	(+)	tgagAGTTtttat	1.0	0.958
	V$MYT1L.01	14578	(-)	ggatAGTTattac	1.0	0.925
V$MZF1	V$MZF1.02	13934	(-)	tgGGGGaagga	1.0	0.995
V$NBRE	V$NBRE.01	15540	(-)	tcacAAGGtcaggag	1.0	0.932
V$NEUR	V$NGN_NEUROD.01	15029	(-)	cataCATCtgtggtc	1.0	0.987
	V$OLIG2.01	14073	(-)	actccaGCTGtttta	1.0	0.980
V$NF1F	V$NF1.02	15403	(-)	agaaTGGCatgaacccaggag	1.0	0.818
V$NFAT	V$NFAT5.02	14552	(+)	acctGGAAagattttacta	1.0	0.917
V$NFKB	V$NFKAPPAB.02	15084	(+)	atGGGAtttcaccat	1.0	0.843
V$NKX1	V$NKX11.01	14024	(-)	ggatctAATTtgttgaa	1.0	0.845
V$NKX6	V$NKX61.01	13869	(-)	aactTTAAttttaga	1.0	0.917
	V$NKX61.01	13903	(-)	tttgTTAAtttaaaa	1.0	0.920
	V$NKX61.01	14164	(+)	ttacTTAAtagcact	1.0	0.923
	V$NKX61.01	15717	(-)	tccaTTAAtttcaaa	1.0	0.961
	V$NKX61.02	15720	(+)	gaaaTTAAtggacat	1.0	0.859
V$NKXH	V$BAPX1.01	15237	(-)	atacatAAGTgaagccata	1.0	0.879
	V$NKX25.01	13563	(-)	cacttcAAGTgctttgggg	1.0	1.000
	V$NKX25.01	14997	(-)	aggctcAAGTgggaggatc	1.0	1.000
	V$NKX25.02	13899	(-)	tttgtTAATttaaaaattt	1.0	0.884
	V$NKX25.02	14164	(+)	ttactTAATagcacttttt	1.0	0.927
	V$NKX25.05	14922	(-)	cagccTGAGtgacagtgtg	1.0	0.988
	V$NKX31.01	14157	(-)	gctattAAGTaacaatatt	1.0	0.908
	V$NKX31.01	15195	(+)	tcttttAAGTttttatgca	1.0	0.878
V$NOLF	V$EBF1.01	13625	(-)	gcctggTCCCctgggcagggaga	1.0	0.978
V$NRF1	V$NRF1.02	14311	(-)	ggtagCGCAcgcctata	1.0	0.811
V$OCT1	V$OCT1.01	15852	(+)	taTATGccatttcag	1.0	0.808
	V$OCT1.03	14807	(+)	caaaactgATTAtca	1.0	0.860
	V$OCT1.03	15258	(-)	gcaaatttATTAgaa	1.0	0.857
	V$OCT1.04	13883	(+)	ttTATGaaaagttta	1.0	0.803
	V$OCT1.05	15411	(+)	ttCATGccattctcc	1.0	0.894
	V$OCT1.06	13905	(-)	actttgttAATTtaa	1.0	0.853
	V$OCT1.06	14494	(+)	tatgtgatAATTtaa	1.0	0.850
	V$OCT1.06	14520	(+)	ctgatgaaAATTtac	1.0	0.870
	V$OCT1.06	14525	(-)	attctgtaAATTttc	1.0	0.820
	V$OCT1.06	14717	(-)	aatatctaAATTgta	1.0	0.860
	V$POU3F3.01	15203	(-)	tgcctGCATaaaaac	1.0	0.859
	V$POU3F3.01	15819	(-)	gtaatGCATatgtca	1.0	0.895
	V$POU3F3.01	15822	(+)	catatGCATtacctc	1.0	0.877
V$OVOL	V$OVOL1.01	13906	(-)	aactttGTTAattta	1.0	0.803
	V$OVOL1.01	15835	(-)	taaactGTTAcatga	1.0	0.864
V$P53F	V$P53.02	14312	(+)	ataggcgtgcgctacCATGcccagc	1.0	0.910
	V$P53.08	14447	(-)	ccaggcatggtggctCATGcctgta	1.0	0.867
	V$P53.08	14448	(+)	acaggcatgagccacCATGcctggc	1.0	0.874
V$PARF	V$DBP.01	15202	(+)	agtttTTATgcaggcaa	1.0	0.863
	V$DBP.01	15606	(-)	ccaagTTATgctgggcg	1.0	0.870

	V$HLF.01	14160	(-)	tgctattaaGTAAcaat	1.0	0.940
	V$TEF.01	14527	(-)	ttctattctGTAAattt	1.0	0.888
	V$TEF.01	14825	(-)	gcaaatgttGTAAggat	1.0	0.853
	V$TEF.01	15624	(+)	atttttgctGTAAagtt	1.0	0.854
	V$TEF.01	15826	(-)	ttacatgagGTAAtgca	1.0	0.898
	V$TEF_HLF.01	14159	(+)	tattgTTACttaatagc	1.0	0.820
	V$VBP.01	15830	(+)	ttacctcatGTAAcagt	1.0	0.949
V$PAX2	V$PAX2.01	14525	(+)	gaaaatttacagaatagAAACac	1.0	0.853
	V$PAX2.01	15843	(-)	tgaaatggcatatacatAAACtg	1.0	0.807
V$PAX7	V$PAX7.01	14807	(+)	caaaactGATTatca	1.0	0.829
V$PAXH	V$PAX4.02	14025	(-)	gatctAATTtgttga	1.0	0.869
	V$PAX4.02	14026	(+)	caacaAATTagatcc	1.0	0.868
V$PBXC	V$PBX3.01	14923	(-)	agcctgagTGACagtgt	1.0	1.000
V$PCBE	V$PREB.01	13691	(+)	tccttTCAGgcacct	1.0	0.870
V$PDX1	V$PDX1.01	14570	(+)	aaaatactgTAATaactat	1.0	0.845
V$PEG3	V$PEG3.01	15045	(+)	cacccacgtgTGGCt	1.0	0.849
V$PIT1	V$PIT1.02	15718	(-)	gtccaTTAAtttcaa	1.0	0.813
V$PLZF	V$PLZF.01	14222	(+)	gagTACAgtggcacg	1.0	0.864
	V$PLZF.01	14941	(+)	gagTACAgtggcatg	1.0	0.880
	V$PLZF.02	13797	(+)	tggTACAgttatact	1.0	0.895
V$PRDF	V$PRDM1.02	15635	(-)	atccagaGAAAgtaacttt	1.0	0.923
V$PURA	V$PURALPHA.01	14252	(-)	ggAGGCggaggtt	1.0	0.986
V$RORA	V$RORA1.01	14389	(-)	ggatcacgaGGTCaagagatcaaga	1.0	0.938
	V$RORA1.01	15533	(-)	ggatcacaaGGTCaggagatcgaga	1.0	0.942
V$RP58	V$RP58.01	15031	(-)	cataCATCtgtgg	1.0	0.872
V$RU49	V$RU49.01	14510	(+)	cAGTAcc	1.0	0.994
V$RUSH	V$SMARCA3.02	14172	(+)	tagcACTTttt	1.0	0.993
	V$SMARCA3.02	14891	(+)	cagaACTTttg	1.0	0.986
V$RXRF	V$RAR_RXR.02	14393	(-)	gggcggatcacgaGGTCaagagatc	1.0	0.941
	V$RAR_RXR.02	15537	(-)	gggtggatcacaaGGTCaggagatc	1.0	0.948
	V$RARG.01	14887	(+)	atatcagaactTTTGgttgttttga	1.0	0.853
V$SAL2	V$SALL2.01	15041	(-)	gtggGTGGcat	1.0	0.926
V$SATB	V$SATB1.01	15259	(+)	tctAATAaatttgct	1.0	0.959
V$SF1F	V$FTF.01	15451	(+)	actaCAAGgtgcccg	1.0	0.943
	V$SF1.01	15541	(-)	atcaCAAGgtcagga	1.0	0.996
V$SIX3	V$SIX3.02	15156	(-)	acgcctgTAATcccaacactt	1.0	0.942
V$SMAD	V$SMAD3.01	15706	(-)	ccaGTCTggcc	1.0	0.991
V$SORY	V$HBP1.01	14754	(+)	gatataggAATGaacatccacta	1.0	0.894
	V$HBP1.02	15719	(+)	tgaaattAATGgacattttttcc	1.0	0.835
	V$HMGA.01	15716	(+)	gtttgaaattAATGgacattttt	1.0	0.906
	V$HMGIY.01	14513	(-)	tgtaAATTttcatcagtagggta	1.0	0.948
	V$HMGIY.01	14524	(+)	tgaaAATTtacagaatagaaaca	1.0	0.951
	V$SOX1.04	14758	(+)	tagGAATgaacatccactattgg	1.0	0.808
	V$SOX15.01	14713	(+)	tgtttACAAtttagatattgtca	1.0	0.882
	V$SOX3.01	14329	(-)	aaaataCAAAagttagctgggca	1.0	0.959
	V$SOX3.01	15662	(+)	gtcccaCAAAtgataaggatgaa	1.0	0.946
	V$SOX4.01	13769	(+)	ctttaACAAaaaaggcaaaaagt	1.0	0.911
	V$SOX4.01	15296	(-)	caacaACAAaaaaattccaagtt	1.0	0.912
	V$SOX4.01	15309	(-)	aaaaaACAAaaagcaacaacaaa	1.0	0.914
	V$SOX5.01	14147	(-)	aagtaaCAATattctgatacgaa	1.0	0.992
	V$SOX5.01	14716	(-)	atttgaCAATatctaaattgtaa	1.0	0.877
	V$SOX5.01	15750	(-)	tagaaaCAATtttttgtcaactg	1.0	0.983
	V$SOX6.01	13804	(-)	aagcaACAAagtcgcagtataac	1.0	0.978
	V$SOX6.01	13908	(+)	aattaACAAagtttcctatcttt	1.0	0.972
V$SP1F	V$GC.01	15130	(-)	aaggcgGGCGgatcttg	1.0	0.881
	V$GC.01	15393	(-)	ccaggaGGCGgagcttg	1.0	0.906
	V$SP1.02	15599	(-)	atgctGGGCggggtggc	1.0	0.957
	V$SP4.01	14406	(-)	gaggtgGGCGgatcacg	1.0	0.897
	V$SP4.02	14309	(+)	attataGGCGtgcgcta	1.0	0.893
	V$TIEG.01	13675	(-)	aactGGGGagtgtgcca	1.0	0.916
V$SRFF	V$SRF.05	13770	(+)	tttaacaaaaAAGGcaaaa	1.0	0.817
	V$SRF.05	14105	(+)	agggacaaatAAGGaccac	1.0	0.820
V$STAT	V$STAT.01	14599	(-)	ttgcttctaGGAAgcaaag	1.0	0.948
	V$STAT.01	14631	(-)	ttgcttctaGGAAgcaaag	1.0	0.948
	V$STAT.01	14663	(-)	ttgcttctaGGAAgcaaag	1.0	0.948
	V$STAT.01	14739	(-)	tatctttttGGAAgcagca	1.0	0.876
	V$STAT3.02	14545	(-)	atctTTCCaggttggagtg	1.0	0.948

	V$STAT5A.01	13971	(+)	tatcTTCCcagtattttct	1.0	0.870
	V$STAT5A.01	14601	(+)	ttgcTTCCtagaagcaagt	1.0	0.957
	V$STAT5A.01	14633	(+)	ttgcTTCCtagaagcaagt	1.0	0.957
	V$STAT5A.01	14665	(+)	ttgcTTCCtagaagcaagt	1.0	0.957
V$STEM	V$OCT3_4.01	15820	(+)	gacatatGCATtacctcat	1.0	0.929
V$TAIP	V$CSRNP1.01	13552	(-)	AGAGtga	1.0	1.000
	V$CSRNP1.01	14062	(+)	AGAGtgg	1.0	1.000
	V$CSRNP1.01	15344	(-)	AGAGtga	1.0	1.000
V$TALE	V$MEIS1.01	14919	(+)	tctcacacTGTCactca	1.0	0.957
	V$MEIS1.01	14967	(+)	ttgcagccTGTCaacct	1.0	0.968
	V$TGIF.01	13985	(-)	gtgctgtatGTCAgaaa	1.0	1.000
	V$TGIF.01	14723	(+)	ttagatattGTCAaatt	1.0	1.000
V$TEAF	V$TEAD4.01	14756	(-)	gttcATTCctata	1.0	0.966
	V$TEAD4.01	15797	(-)	aggcATTCcacaa	1.0	0.943
V$THAP	V$THAP1.01	14803	(-)	agttttGGCAa	1.0	0.944
V$WHNF	V$WHN.01	13997	(+)	agcACGCtttt	1.0	0.959
V$XBBF	V$RFX5.01	13817	(-)	ctgtgttttaAGCAacaaa	1.0	0.965
	V$RFX5.01	15312	(-)	aaaaacaaaaAGCAacaac	1.0	0.968
V$YY1F	V$YY1.02	13651	(-)	tcctggCCATgttctcagcctgc	1.0	0.954
	V$YY1.02	14879	(+)	tttagaCCATatcagaacttttg	1.0	0.957
	V$YY2.02	15411	(+)	ttcatgCCATtctcctgcctcag	1.0	0.835
V$ZBED	V$ZBED1.02	14186	(-)	cTGTCtcgaaaaa	1.0	0.812
V$ZF01	V$SZF1.01	14781	(-)	caaGGGTaggagaaatctactcttc	1.0	0.821
V$ZF02	V$ZBTB7.03	15598	(+)	agccaCCCCgcccagcataactt	1.0	0.890
	V$ZNF300.01	13677	(+)	gcacactCCCCagttcctttcag	1.0	0.994
V$ZF04	V$ZID.01	15125	(+)	ggGCTCaagatcc	1.0	0.855
V$ZF05	V$ZFP410.01	15080	(+)	agagatgGGATttca	1.0	0.891
V$ZF06	V$ZBTB7.02	14115	(+)	aagGACCacagtc	1.0	0.899
V$ZF08	V$ZNF354C.01	14034	(+)	tagatCCACag	1.0	0.971
	V$ZNF354C.01	15551	(+)	gtgatCCACcc	1.0	0.976
V$ZF12	V$ZFP652.01	13735	(-)	tagaaaggcTTAAac	1.0	0.901
V$ZF5F	V$ZF5.01	14314	(+)	aggcgtGCGCtacca	1.0	0.959
V$ZFHX	V$AREB6.02	13589	(+)	ttatCACCtctcc	1.0	0.972
V$ZICF	V$ZIC2.02	13644	(+)	aggctCAGCaggctg	1.0	0.979
V$ZTRE	V$ZTRE.03	13680	(+)	caCTCCccagttccttt	1.0	0.971
	V$ZTRE.04	13672	(-)	tggGGAGtgtgccactc	1.0	0.959

Names of matrix families: AARF: AARE-binding factors (amino acid-response element, ATF4-binding site); ABDB: abdominal-B type homeodomain transcription factors (HOX matrix); AHRR: AHR-arnt heterodimers and AHR-related factors (aryl hydrocarbon/dioxin receptor); AIRE: autoimmune regulatory element-binding factor; AP1F: AP1, Activating protein 1; AP1R: MAF and AP1-related factors; AP2F: Activator protein 2; AP4R: AP4 and related proteins; ARID: AT-rich interactive domain factor; ATBF: AT-binding transcription factor; BARB: Barbiturate-inducible element box from pro- and eukaryotic genes; BCDF: bicoid-like homeodomain transcription factors; BCL6: POZ domain zinc finger expressed in B-Cells; BEDF: BED subclass of zinc-finger proteins; BHLH: bHLH transcription factors expressed in muscle, intestine and stomach; BNCF: basonuclein rDNA transcription factor (PolI); BPTF: bromodomain and PHD domain transcription factors; BRAC: Brachyury gene, mesoderm developmental factor; BRN5: Brn-5 POU domain factors; BRNF: Brn POU domain factors; BTBF: BTB/POZ (broad complex, TramTrack, Bric-a-brac/pox viruses and zinc fingers) transcription factor; BZIP: heterodimers between bZIP family members; CAAT: CCAAT-binding factors; CABL: C-abl DNA binding sites; CARE: calcium-response elements; CART: Cart-1 (cartilage homeoprotein 1); CDEF: cell cycle regulators: Cell cycle dependent element; CDXF: vertebrate caudal-related homeodomain protein; CEBP: Ccaat/Enhancer Binding Protein; CHOP: C/EBP homologous protein (CHOP); CHREF: cell cycle regulators: cell cycle homology element; CIZF: CAS interacting zinc finger protein; CLOX: CLOX and CLOX homology (CDP) factors; CP2F: CP2-erythrocyte factor related to drosophila Elf1; CREB: cAMP response-element-binding protein; CSEN: calsenilin, presenilin-binding protein, EF hand transcription factor; CTCF: CTCF and BORIS gene family, transcriptional regulators with 11 highly-conserved zinc finger domains; DEAF: homolog to deformed epidermal autoregulatory factor-1 from *Drosophila melanogaster*; DICE: downstream immunoglobulin control element, critical for B cell activity and specificity; DLXF: distal-less homeodomain transcription factors; DMRT: DM domain-containing transcription factors; DMTF: Cyclin D binding myb-like transcription factor; DUXF: double homeobox factors; E2FF: E2F-myc activator/cell cycle regulator; E4FF: ubiquitous GLI - Krueppel-like zinc finger involved in cell cycle regulation; EBOX: E-box-binding factors; EGRF: EGR/nerve growth factor-induced protein C & related factors; EREF: estrogen-response elements; ESRR: estrogen-related receptors; ETSF: human and murine ETS1 factors; EVI: EVI1-myeloid transforming protein; FAST: FAST-1 SMAD-interacting proteins; FKHD: Fork head domain factors; FXRE: farnesoid X - activated receptor response elements; GABF: GA-boxes; GATA: GATA-box-binding factor; GCF2: transcriptional repressor GC-binding factor 2; GCMF: Chorion-specific transcription factors with a GCM DNA-binding domain; GCNR: germ cell nuclear receptors; GFI1: zinc finger protein Gfi-1 (growth factor independent protein 1 zinc finger protein 163; GLIF: GLI zinc finger family; GREF: glucocorticoid-responsive and related elements; GRHL: grainyhead-like transcription factors; GUCE: GTF2IRDI upstream control element; HAML: acute myeloid leukaemia gene 1a (Runx/runt homology domain); HAND: Twist subfamily of class B bHLH transcription factors; HASF: HIF-1 ancillary

sequence family; HBOX: homeobox transcription factors; HDBP: Huntington's disease gene regulatory region binding proteins; HEAT: heat shock factors; HESF: vertebrate homologues of enhancer of split complex; HICF: Krueppel-like C2H2 zinc finger factors hypermethylated in cancer; HIFF: hypoxia-inducible factor, bHLH/PAS protein family; HMTB: human muscle-specific Mt-binding site; HNF1: hepatic nuclear factor 1; HNF6: onecut homeodomain factor HNF6; HNFP: histone nuclear factor P; HOMF: homeodomain transcription factors; HOXC: HOX - PBX complexes; HOXF: paralog hox genes 1-8 from the four hox clusters A, B, C, D; HOXH: HOX - MEIS1 heterodimers; HUB1: HTLV-I U5 repressive element-binding protein 1; HZIP: homeodomain-leucine zipper transcription factors; IKRS: Ikaros zinc finger family; IKZF: Ikaros family zinc finger 5; INRE: core promoter initiator elements; INSM: insulinoma associated factors; IRFF: interferon regulatory factors; IRXF: iroquois homeobox transcription factors; KLFS: Krueppel-like transcription factors; LEFF: LEF1/TCF; LHXF: Lim homeodomain factors; LHXF: Lim homeodomain factors; LTFM: lactotransferrin motif; LTSM: localized tandem sequence motif; MAZF: Myc-associated zinc fingers; MEF2: MEF2, myocyte-specific enhancer-binding factor; MEF3: MEF3 binding sites; MITF: microphthalmia transcription factor; MIZ1: Myc-interacting Zn finger protein 1; MOKF: mouse Krueppel-like factor; MTEN: core promoter motif ten elements; MTF1: metal-induced transcription factor; MYBL: cellular and viral myb-like transcriptional regulators; MYOD: myoblast determining factors; MYRF: myelin regulatory factors; MYT1: MYT1 C2HC zinc finger protein MZF1: myeloid zinc finger 1 factors; NACA: nascent polypeptide associated complex and coactivator alpha; NBRE: NGFI-B-response elements, subfamily of nuclear receptors; NDPK: nucleoside diphosphate kinase; NEUR: NeuroD, β2, HLH domain; NF1F: nuclear factor 1; NFAT: nuclear factor of activated T-cells; NFKB: nuclear factor κB/c-rel; NGRE: "negative"glucocorticoid-response elements; NKX1: NK1 homeobox transcription factors; NKX6: NK6 homeobox transcription factors; NKXH: NKX homeodomain factors; NOLF: neuron-specific olfactory factor; NR2F: nuclear receptor subfamily 2 factors; NRF1: Nuclear respiratory factor 1; NRSF: Neuron-restrictive silencer factor; OCT1: Octamer binding protein; OSRF: odd-skipped related factors; OVOL: OVO homolog-like transcription factors; P53F: p53 tumor suppressor; PARF: PAR/bZIP family; PAX2: PAX-2 binding sites; PAX3: PAX-3-binding sites; PAX5: PAX-2/5/8-binding sites; PAX6: PAX-4/PAX-6 paired domain-binding sites; PAX7: PAX-7-binding sites; PAXH: PAX homeodomain binding sites; PBXC: PBX - MEIS complexes; PCBE: PREB core-binding element; PDX1: pancreatic and intestinal homeodomain transcription factor; PEG3: imprinted transcription factor PEG3; PERO: peroxisome proliferator-activated receptor; PIT1: GHF-1 pituitary specific POU domain transcription factor; PLAG: pleomorphic adenoma gene; PLZF: C2H2 zinc finger protein PLZF; PRDF: Positive regulatory domain I-binding factor; PROX: prospero-related homeobox; PTBP: Plant TATA binding protein factor; PTF1: pancreas transcription factor 1, heterotrimeric transcription factor; PURA: Pur-α binds both single-stranded and double-stranded DNA in a sequence-specific manner; RBP2: retinoblastoma-binding proteins with demethylase activity; RBPF: RBPJ – κ; RORA: v-ERB and RAR-related orphan receptor α; RP58: RP58 (ZFP238) zinc finger protein; RREB: Ras-responsive element-binding protein; RU49: zinc finger transcription factor RU49, zinc finger proliferation 1 - Zipro1; RUSH: SWI/SNF related nucleophosphoproteins with a RING finger DNA binding motif; RXRF: RXR heterodimer-binding sites; SAL1: spalt-like transcription factor 1; SAL2: spalt-like transcription factor 2; SATB: special AT-rich sequence binding protein; SF1F: vertebrate steroidogenic factor; SIX3: sine oculis homeobox homolog 3; SIXF: sine oculis (SIX) homeodomain factors; SMAD: vertebrate SMAD family of transcription factors; SNAP: snRNA-activating protein complex; SORY: SOX/SRY-sex/testis determining and related HMG box factors; SP1F: GC-Box factors SP1/GC; SPZ1: testis-specific bHLH-Zip transcription factors; SREB: sterol-regulatory element-binding proteins; SRFF: serum-response element-binding factor; STAF: selenocysteine tRNA activating factor; STAT: signal transducer and activator of transcription; STEM: motif composed of binding sites for pluripotency or stem cell factors; TAIP: TGF-β induced apoptosis proteins; TALE: TALE homeodomain class recognizing TG motifs; TCFF: TCF11 transcription factor; TEAF: TEA/ATTS DNA binding domain factors; TELO: protein involved in telomere maintenance; TF2B: RNA polymerase II transcription factor II B; TF3C: general transcription factor IIIC, GTF3C; THAP: THAP domain containing protein; VTBP: vertebrate TATA binding protein factor; WHNF: winged helix binding sites; XBBF: X-box-binding factors; XCPE: activator-, mediator- and TBP-dependent core promoter element for RNA polymerase II transcription from TATA-less promoters; YBXF: Y-box binding transcription factors, multifunctional proteins involved in transcriptional and translational regulation, mRNA splicing, DNA replication and repair; YTBP: yeast TATA binding protein factor; YY1F: activator/repressor-binding to transcription initiation site; ZBED: Zinc finger BED domain-containing protein; ZF01: C2H2 zinc finger transcription factors 1; ZF02: C2H2 zinc finger transcription factors 2; ZF03: C2H2 zinc finger transcription factors 3; ZF04: C2H2 zinc finger transcription factors 4; ZF05: C2H2 zinc finger transcription factors 5; ZF06: C2H2 zinc finger transcription factors 6; ZF07: C2H2 zinc finger transcription factors 7; ZF08: C2H2 zinc finger transcription factors 8; ZF10: C2H2 zinc finger transcription factors 10; ZF11: C2H2 zinc finger transcription factors 11; ZF12: C2H2 zinc finger transcription factors 12; ZF15: C2H2 zinc finger transcription factors 15; ZF35: Zinc finger protein ZNF35; ZF57: KRAB domain zinc finger protein 57; ZF5F: ZF5 POZ domain zinc finger; ZFHX: two-handed zinc finger homeodomain transcription factors; ZFXY: Zfx and Zfy - transcription factors implicated in mammalian sex determination; ZTRE: zinc transcriptional regulatory element
